# *N*‐Alkoxysulfurdiimide Reagents for the One‐Step Modular Synthesis of Primary Sulfonimidamides

**DOI:** 10.1002/anie.7086997

**Published:** 2026-07-07

**Authors:** Suzanne E. Davison, Jonathan A. Andrews, Agamemnon E. Crumpton, Mireia Sidera Portela, Michael A. Clegg, Damien Valette, Michael C. Willis

**Affiliations:** ^1^ Department of Chemistry, Chemistry Research Laboratory University of Oxford Oxford UK; ^2^ Vertex Pharmaceuticals Milton, Abingdon UK; ^3^ Discovery Chemistry MSD (UK) Limited London UK

## Abstract

Sulfonimidamides are an emerging motif in medicinal chemistry; however, current synthetic routes often require multistep reaction sequences, deprotection steps, and lack the modularity needed for rapid and diverse library synthesis. Here, we describe the synthesis and application of previously unexplored *N*‐alkoxysulfurdiimide reagents for the direct, one‐step preparation of primary NH_2_‐sulfonimidamides. By modulating the choice of *N*‐substituent, *N*‐alkoxysulfurdiimide reagents can be designed to deliver different product distributions. This reactivity correlates with the electron density at sulfur, as inferred from ^13^C NMR chemical shifts from the reagents. This tunability can be exploited to design reagents with improved compatibility across organometallic reagents, enabling the preparation of a diverse set of primary NH_2_‐sulfonimidamides.

## Graphical abstract

*N*‐Alkoxysulfurdiimide reagents when combined with organometallic reagents lead directly to primary sulfonimidamide products. The reactions are broad in scope, encompassing aryl, heteroaryl, and alkyl carbon substituents. The reactivity of the *N*‐alkoxysulfurdiimide reagents can be correlated with the electron‐withdrawing ability of the N‐substituent.

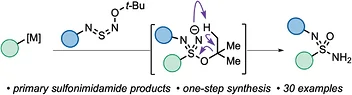

Sulfonamides are a privileged functional group that have been widely employed in medicine [1, 2], agriculture [3], dyes [4], catalysis [5], and polymer chemistry [6]. In medicinal chemistry, they appear in more than 72 FDA‐approved drugs [7], spanning diverse therapeutic areas such as arthritis (e.g., celecoxib) [8], hypertension (e.g., furosemide) [9], and epilepsy (e.g., acetazolamide, Scheme 1A) [10]. Sulfonimidamides, the mono‐aza analogues of sulfonamides, exhibit several properties that make them compelling motifs in drug discovery. The single switch of an oxygen atom with an imidic nitrogen renders the sulfur atom stereogenic, provides an additional functional handle for exploring chemical space, and enables modulation of physicochemical properties (Scheme 1A) [11, 12, 13]. With increasing reports in the patent literature [14, 15, 16], and a small number of sulfonimidamide‐containing compounds having advanced into clinical trials [17], sulfonimidamides are positioned as a scaffold primed with considerable unexplored potential.

**SCHEME 1:**
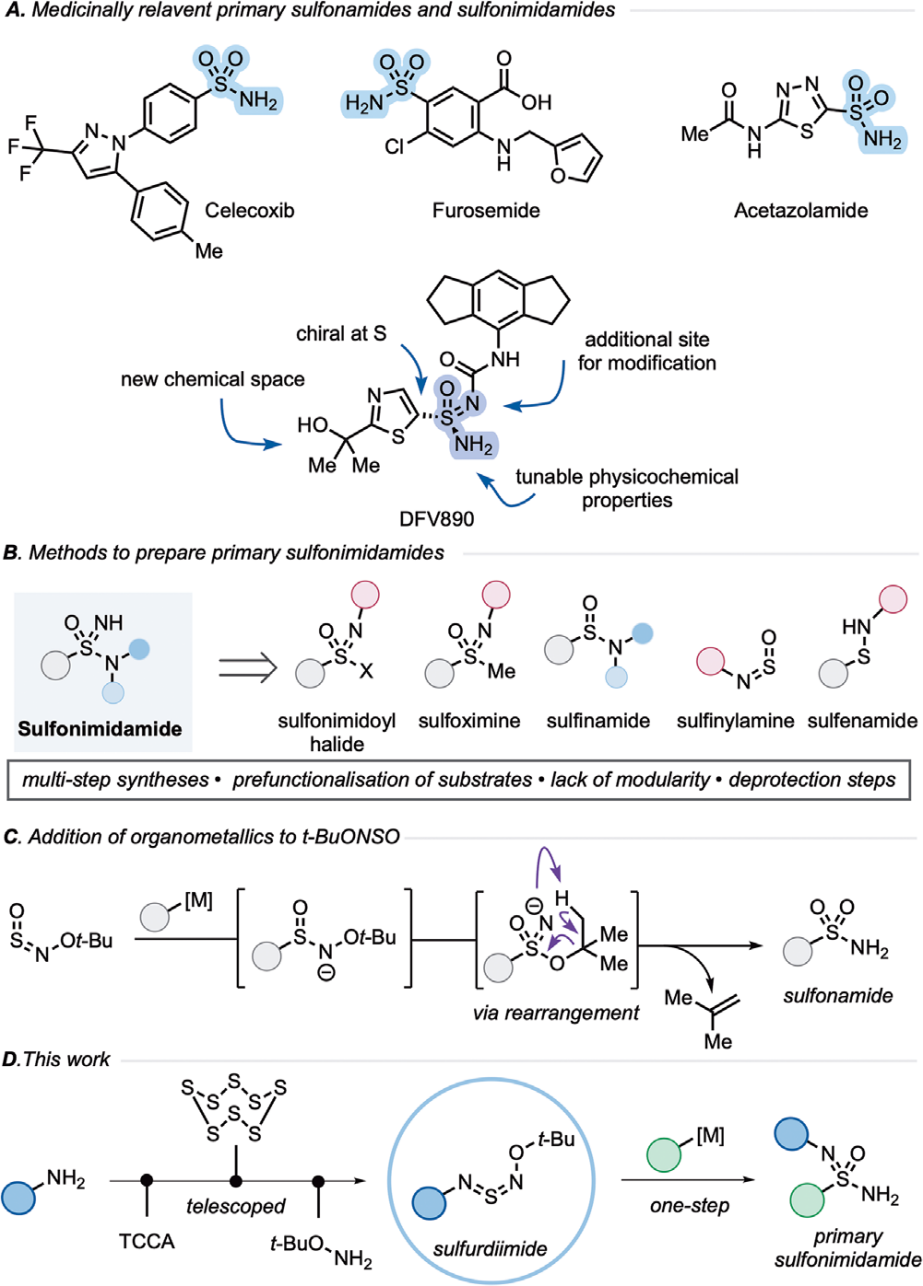
(A) Primary‐sulfonamide containing drugs and primary sulfonimidamide patented compound DFV890. (B) Methods to make primary sulfonimidamides. (C) Addition of organometallics to *t*‐BuONSO. (D) This work: the direct synthesis of primary sulfonimidamides using *N*‐alkoxysulfurdiimide reagents.

Common synthetic approaches to access sulfonimidamides typically begin with S(IV) or S(VI) precursor molecules, with the carbon‐based substituent pre‐installed (Scheme 1B). For example, the oxidation of S(II) or S(IV) compounds such as sulfenamides [18] or sulfinamides [19, 20, 21], as well as visible‐light‐mediated nitrene transfer to sulfinamides [22], have all been exploited to access sulfonimidamides. Additionally, sulfonimidamides can be prepared either directly or indirectly from sulfinylamine reagents through reaction with organometallics [21, 23, 24], alkyl radicals [25, 26, 27, 28], aryl radicals [29, 30], boroxines [31], aryl bromides [32], and diazonium salts [33]. S(VI) compounds such as sulfoximines and sulfonimidoyl halides can also be transformed to sulfonimidamides; for example, Bolm described copper‐catalyzed dealkylation followed by amination of sulfoximines to access sulfonimidamides [34], while sulfonimidoyl halides are widely reacted with amines via halide‐amine exchange [23, 35, 36, 37, 38]. Asymmetric syntheses of these groups are also emerging [39, 40, 41]. A limitation to the widespread use of sulfonimidamides in medicinal chemistry is the requirement for multistep reaction sequences for their synthesis, which often lack modularity and require deprotection steps to access NH_2_‐sulfonimidamides.

The Willis laboratory has previously developed several sulfinylhydroxylamine reagents (now commercially available) that can be used as versatile building blocks to rapidly access S(VI) functionalities [24, 42]. In particular, we have demonstrated that the *t*‐BuO‐NSO reagent can be used for the one‐step synthesis of primary sulfonamides via organometallic reagent addition and subsequent rearrangement (Scheme 1C) [42]. Inspired by this reactivity, we envisaged that the aza‐analogues of these reagents (*t*‐BuO‐NSN‐R), if synthetically accessible, should enable the direct preparation of the primary sulfonimidamide core in a single step. In addition, replacing an oxygen atom with an imidic nitrogen within the reagent should allow tuning of its reactivity through electronic and/or steric control. To date, however, no examples of R'O‐NSN‐R reagents have appeared in the literature. In this report, we describe the synthesis of a series of *t*‐BuO‐NSN‐R reagents and investigate their reactivity towards organometallic reagents, ultimately delivering a one‐step route to primary sulfonimidamides (Scheme 1D).

For the synthesis of *t*‐BuO‐NSN‐R reagents, inspiration was taken from the preparation of *t*‐BuONSO, where thionyl chloride is added to a free‐based solution of *O*‐*tert*‐butylhydroxylamine in the presence of base [42]. The aza‐derivatives of thionyl chloride, imidothionyl chlorides, have previously been used to prepare aza‐sulfur(VI) compounds. This has been demonstrated by De Borggraeve and Demaerel [43] and Bolm [44] to afford thiazynes and imidosulfites, respectively, and builds from the earlier work of Levchenko and co‐workers [45]. Encouraged by this, a telescoped procedure was designed to access *N*‐alkoxysulfurdiimides harnessing an imidothionyl chloride intermediate. First, amines were smoothly converted to the respective *N,N*‐dichloramine via reaction with an excess of trichloroisocyanuric acid (Scheme 2A). Following filtration, the *N,N*‐dichloramine was added to (commercial) sublimed sulfur and sub‐stoichiometric tetrabutylammonium bromide to afford a reactive imidothionyl chloride intermediate. *O*‐*tert‐*Butylhydroxylamine hydrochloride and a threefold excess of pyridine were then added to the imidothionyl chloride solution in situ to afford *N*‐alkoxysulfurdiimides (**1**), which could be purified without the requirement for column chromatography. Full details of the optimization can be found in the Supporting Information (Tables S1–S5). The *N*‐substituent of the *t*‐BuO‐NSN‐R reagents was found to significantly influence the stability and reactivity of the reagents. A series of stable reagents were synthesized with *N*‐substituents including sulfonamide (**1a‐b**), carbamate (**1c‐f**), amide (**1g‐h**), urea (**1i**), and alkyl (**1j**) groups in moderate to excellent yields. In line with similar sulfinylhydroxylamine reagents, these new reagents could be stored for several months at −18°C under an inert atmosphere without degradation. The structure of **1c** was confirmed by single‐crystal x‐ray crystallography [46], could be prepared on a 20 mmol scale affording 3.44 g in 67% yield, and is now commercially available [47].

**SCHEME 2:**
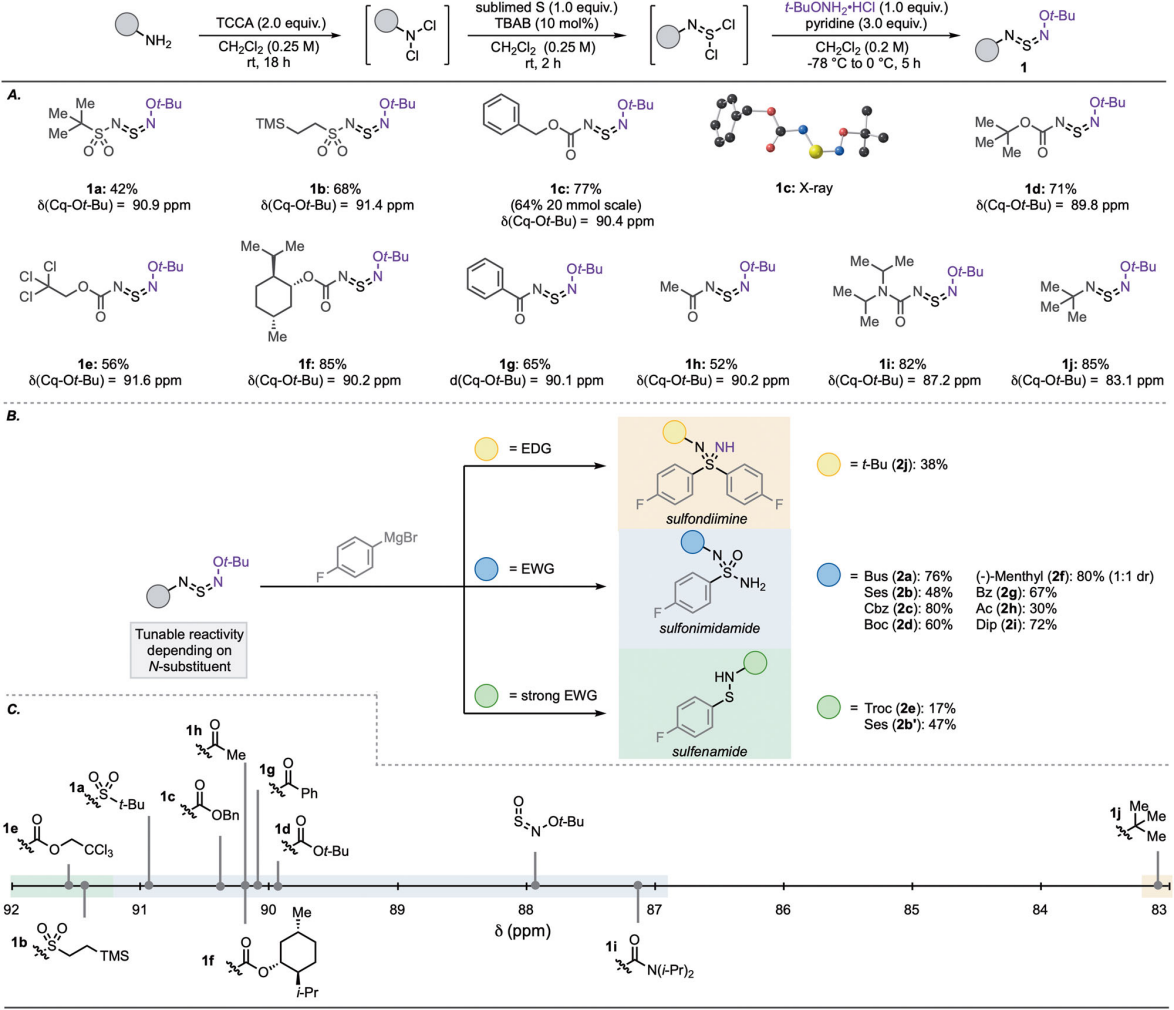
(A) Preparation of *t*‐BuO‐NSN‐R reagents (**1a**‐**1j**); reaction conditions: R‐NH_2_ (1.0 equiv.), TCCA (2.0 equiv.), CH_2_Cl_2_ (0.25 M), rt, 18 h, then sublimed sulfur (1.0 equiv.), TBAB (10 mol%), CH_2_Cl_2_ (0.25 M), rt, 2 h, then *t*‐BuONH_2_·HCl (1.0 equiv.), pyridine (3.0 equiv.), CH_2_Cl_2_ (0.2 M), −78°C to 0°C, 5 h. Isolated yields. 5 mmol scale. (B) Reactions of *t*‐BuO‐NSN‐R reagents with 4‐F‐C_6_H_4_‐MgBr. Reaction conditions: *t*‐BuO‐NSN‐R (1.0 equiv.), 4‐fluorophenylmagnesium bromide (1.1 equiv.), THF (0.1 M), 0°C to rt, 30 min. Isolated yields. (C) ^13^C NMR spectrum showing the quaternary O*t‐*Bu ^13^C shift in *t*‐BuO‐NSN‐R depending on *N*‐substituent.

With a selection of *t*‐BuO‐NSN‐R reagents in hand, we next explored their reactivity towards organometallic addition using commercially available 4‐fluorophenylmagensium bromide as a model (Scheme 2B; full details of the optimization can be found in the Supporting Information (Tables S6–S12). Three distinct modes of reactivity were observed, depending on the nature of the *N*‐substituent (R), affording either a primary sulfonimidamide, sulfondiimine, or sulfenamide. Intuitively, which product dominated seemed to correlate to the electrophilicity of the reagent used, and data to confirm this was available by considering the ^13^C NMR chemical shift of the quaternary carbon of the O*t*‐Bu group within the reagents (Scheme 2C). The observed chemical shifts ranged from 83.1 ppm for electron‐donating substituents (e.g., *t‐*Bu, **1j**) to 91.6 ppm for more electron‐withdrawing substituents (e.g., Troc, **1j**). In terms of which product was obtained from which reagent, reagents bearing moderately electron‐withdrawing *N*‐substituents such as Bus (**1a**), Cbz (**1c**), Boc (**1d**), (‐)‐menthyl carbamate (**1f**), benzyl (**1g**), acetyl (**1h**), and diisopropyl urea (**1i**) provided the corresponding sulfonimidamides (**2a, 2c‐d, 2f‐i**) in good to excellent yields. These reagents have quaternary O*t*‐Bu carbon shifts ranging from 90.9 to 87.2 ppm. Reagents that feature more electron‐withdrawing substituents, such as a 2‐(trimethylsilyl)ethanesulfonyl (Ses) group (**1b**) or a Troc group (**1f**), which have quaternary O*t*‐Bu carbon shifts of 91.4 and 91.6 ppm, respectively, deliver mixtures of sulfonimidamides and sulfenamides, or sulfenamide exclusively. The formation of sulfenamides from the rearrangement of S(IV) intermediates has been reported [48, 49]. In contrast, when a reagent featuring the electron‐donating *tert*‐butyl group was used as the *N*‐substituent (**1j**), with a quaternary O*t*‐Bu carbon shift of 83.1 ppm, the symmetrical sulfondiimine **2j** was formed in 38% yield [24].

To evaluate the scope of sulfonimidamides that could be prepared, and to explore the application of the reaction to other organometallic reagents, *N*‐alkoxysulfurdiimide **1c** (*t‐*BuO‐NSN‐Cbz) was combined with a wide range of Grignard reagents (Scheme 3). Aryl Grignard reagents (**2c**, **3a‐i**), bearing both electron‐donating and electron‐withdrawing groups, were well tolerated under the reaction conditions. Compared to *t*‐BuONSO, *t‐*BuO‐NSN‐Cbz **1c** showed increased sensitivity to steric effects, likely due to its greater size. Accordingly, a small reduction in yield was observed across *para‐, meta‐*, and *ortho*‐methyl aryl substituents (**3b‐d**). Grignard reagents prepared via magnesium‐halogen exchange using *i*‐PrMgCl enabled the incorporation of electrophilic functional groups such as nitriles (**3h**) and amides (**3i**). Alkyl Grignard reagents were also compatible, including methyl (**3j**), primary (**3k‐m**), secondary (**3n‐p**), benzylic (**3q**), vinyl (**3r**), allyl (**3s**), and tertiary (**3t**) species. Benzyl‐protected piperidine (**3o**), containing a basic nitrogen centre, was tolerated. The potentially sensitive vinyl and allyl sulfonimidamides (**3r**, **3s**) were delivered in 51% and 55% yields, respectively. The transformation was equally effective on a preparative scale, with a gram‐scale reaction providing **3a** in 73% yield (5 mmol). A variety of heterocyclic Grignard reagents, including indole (**3u**), benzofuran (**3v**), 2‐thienyl (**3w**), and pyrazole (**3x**) also successfully underwent the transformation to afford the corresponding sulfonimidamide products. In contrast, organolithium reagents, particularly those used to introduce pyridyl substituents, were incompatible with reagent **1c**, leading predominantly to decomposition of *t‐*BuO‐NSN‐Cbz as well as reductive sulfenamide products in low yields. We speculated that this was due to the combination of the moderately electrophilic sulfurdiimide reagent (**1c**) combined with highly nucleophilic organolithium species, favoring the reductive pathway and forming sulfenamide products typically observed for highly electrophilic reagents (**1b** and **1e**).

**SCHEME 3:**
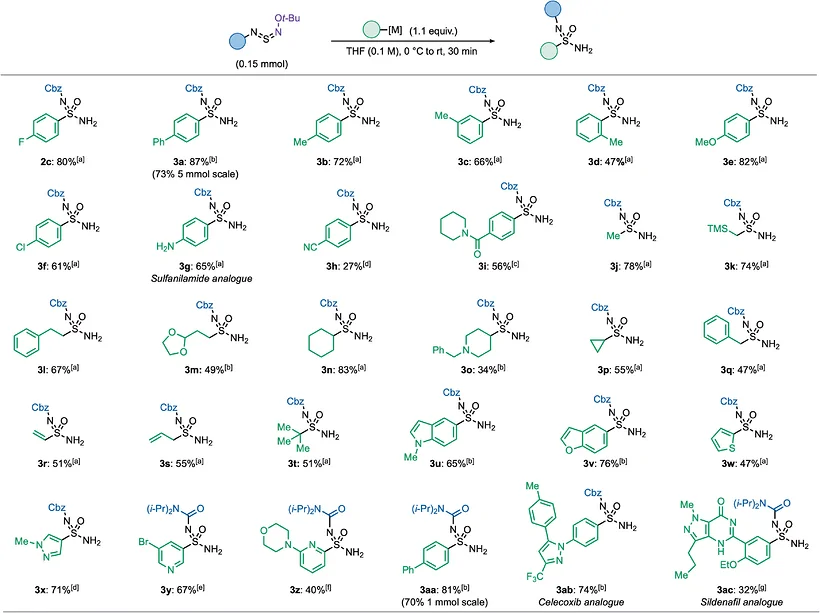
Reaction conditions: *t*‐BuO‐NSN‐Cbz (1.0 equiv.), organometallic reagent (1.1 equiv.), THF (0.1 M), 0°C to rt, 30 min. Isolated yields. [a] Commercial Grignard reagent; [b] generated from aryl bromide and Mg metal; [c] generated from aryl iodide and *i*‐PrMgBr; [d] Generated from aryl iodide and *i*‐PrMgCl; [e] generated from (hetero)aryl bromide and *i*‐PrMgCl**·**LiCl; [f] generated from (hetero)aryl bromide and *n*‐BuLi; [g] generated from aryl bromide, MeLi, and *n*‐BuLi.

To address this limitation, we turned to sulfurdiimide **1i**, which is a less electrophilic variant bearing a diisopropyl urea (Dip) substituent [40]. This enabled the use of more reactive organometallics, including organolithium and “turbo” Grignard reagents, while still yielding the desired sulfonimidamide products. This improved compatibility likely reflects a better electronic match between the nucleophilic and electrophilic components. Using reagent **1i**, both 2‐ and 3‐pyridyl sulfonimidamides (**3y**, **3z**) were accessed successfully. In addition, several more complex primary sulfonimidamides, including analogues of sulfonamide drugs, were obtained in synthetically useful yields. For example, a silyl‐protected aniline Grignard reagent furnished the sulfanilamide analogue **3g** in 65% yield. Celecoxib (**3ab**) and sildenafil (**3ac**) analogues were also successfully prepared in 74% and 32% yields, respectively.

Removal of the Cbz group from sulfonimidamide **3a** could be achieved using hydrogenation conditions to afford NH, NH_2_‐sulfonimidamide **4** in 80% yield (Scheme 4). Likewise, the Dip group present in **3aa** could be removed employing Lopchuk's acidic deprotection conditions [40] using camphorsulfonic acid to afford **4** in 51%.

**SCHEME 4:**
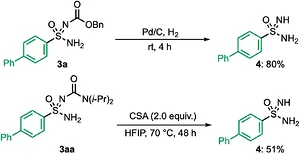
Removal of Cbz and Dip protecting groups from sulfonimidamide **3a** and **3aa** to afford sulfonimidamide **4**.

In conclusion, we have developed a route to access a series of previously unexplored *t*‐BuO‐NSN‐R *N*‐alkoxysulfurdiimide reagents featuring a variety of *N*‐substituents. The reactivity of *t*‐BuO‐NSN‐R reagents towards organometallic addition has been explored, and three modes of reactivity have been identified. ^13^C NMR spectroscopy has been used as a tool to approximate the electrophilicity at sulfur and could be applied to guide reagent design to identify reagents which afford primary sulfonimidamides. This approach also allowed identification of less reactive reagents which could be combined with more nucleophilic organometallic partners, such as organolithiums. By utilizing commercial or readily available organometallic reagents, this methodology offers a rapid and modular route to access the primary sulfonimidamide core.

## Conflicts of Interest

The authors declare no conflicts of interest.

## Supporting information




**Supporting File**: anie73445‐sup‐0001‐SuppMat.pdf.

## Data Availability

The data that support the findings of this study are available in the Supporting Information of this article.
