# Supplementary material for: N‐Alkoxysulfurdiimide Reagents for the One‐Step Modular Synthesis of Primary Sulfonimidamides
Source: Angew Chem Int Ed Engl. 2026 Jul 7;65(35):e7086997. doi: 10.1002/anie.7086997 (PMC13502562; doi:10.1002/anie.7086997)
Supplement: Supplementary file 1 [file ANIE-65-e7086997-s001.pdf]

Supporting Information

N-Alkoxysulfurdiimide Reagents for the One-Step Modular Synthesis of Primary Sulfonimidamides

Suzanne E. Davison,[a] Jonathan A. Andrews,[a] Agamemnon E. Crumpton,[a] Mireia Sidera Portela,[b] Michael A. Clegg,[c] Damien Valette,[c] Michael C. Willis [a,*]

[a] Department of Chemistry, University of Oxford, Chemistry Research Laboratory, Mansfield Road, Oxford, OX1 3TA, UK.

[b] Vertex Pharmaceuticals, 86–88 Jubilee Ave, Milton, Abingdon, OX14 4RW, UK.

[c] Discovery Chemistry, MSD (UK) Limited, 120 Moorgate, London EC2M 6UR, UK.

michael.willis@chem.ox.ac.uk

Table of Contents

1. General Information S3

1.1 General Experimental Considerations S3

1.2 HPLC yields...S4

2. Preparation of Starting Materials S5

2.1 Preparation of Starting Materials for the synthesis of *N*-Alkoxysulfurdiimides S5

2.2 Preparation of Organometallic Reagents S6

3. Optimization of Conditions..S7

3.1 Optimization of *t*-BuO-NSN-R reagent synthesis S7

3.2 Optimization of Grignard reagent into *t*-BuO-NSN-R reagents S10

4. Experimental Procedures and Data S14

4.1 Synthesis of *N*-Alkoxysulfurdiimides (1) S14

4.2 Differential Scanning Calorimetry tests for *N*-Alkoxysulfurdiimides 1c and 1i S22

4.3 Reaction of *N*-alkoxysulfurdiimide reagents 1a-j with Grignard reagents................................S23

4.4 Synthesis of Primary Sulfonimidamides (3) S31

4.5 *N*-Deprotection of sulfonimidamide 3a and 3aa S54

5. Single Crystal X-ray Diffraction S56

6. References S58

7. NMR Spectra S59

1. General Information

1.1 General Experimental Considerations

All reactions were performed under an inert atmosphere of nitrogen or argon using standard Schlenk techniques, unless stated otherwise. All glassware was oven-dried at >100 °C for at least 2 hours and allowed to cool to room temperature under a positive pressure of nitrogen. All chemicals were purchased from commercial suppliers (including Sigma Aldrich, Alfa Aesar, Fisher Scientific, Acros Organics, Fluorochem) and were used as received unless specified. Anhydrous solvents were obtained through the University of Oxford internal solvent drying system (Innovative Technology Inc. PS-400-7), stored over activated 3 Å molecular sieves and degassed by bubbling with N_2 for at least 15 minutes. Anhydrous pentane was obtained through storing distilled HPLC grade pentane over activated 3 Å molecular sieves for at least 24 hours before use. Cooling of reaction mixtures to 0 °C was achieved using an ice-water bath, –15 °C, –20 °C or –78 °C using an acetone-dry ice bath. Heating of reaction mixtures was achieved using an aluminium heating block. Where possible, reactions were monitored using TLC with aluminium backed silica gel 60 F_{254} plates. Plates were visualised with 254 nm UV light and/or staining with potassium permanganate, phosphomolybdic acid, ninhydrin or vanillin solutions. Flash column chromatography was performed using Millipore silica gel 60 with the indicated solvent system.

^{1}H, ^{13}C and ^{19}F NMR spectra were recorded on a Bruker AV III HD 400 spectrometer (at 400 MHz, 101 MHz and 377 MHz respectively), a Bruker AV III HD 500 spectrometer (at 500 MHz, 126 MHz and 470 MHz respectively) or a NEO600 spectrometer (at 600 MHz, 151 MHz and 565 MHz respectively). All spectra were recorded at room temperature and referenced to the residual signal of deuterated solvent ($CDCl_3$: δ_H = 7.26 ppm, δ_C = 77.16 ppm and $(CD_3)_2SO$: δ_H = 2.50 ppm, δ_C = 39.52 ppm). ^{19}F NMR spectra were referenced indirectly using the spectrometer frequency-ratio method relative to the ^{1}H reference standard, corresponding to the $CFCl_3$ scale. All chemical shifts (δ) are reported in parts per million (ppm) to two decimal places for ^{1}H NMR and to one decimal place for ^{13}C NMR and ^{19}F NMR. Multiplicities are reported as s (singlet), d (doublet), t (triplet), q (quartet), m (multiplet), and combinations thereof. Coupling constants (*J*) are given in Hertz (Hz). Infrared spectra were recorded on a Bruker Tensor 27 Fourier Transform spectrometer with an internal range of 600-4000 cm^{-1} and all absorption maxima (ν_{max}) are given in wavenumbers (cm^{-1}). Low resolution mass spectra were recorded on a Waters LCT Premier mass spectrometer. High resolution mass spectra (HRMS) were recorded on a Waters BioAccord TOF spectrometer under electrospray ionisation conditions (ESI) *via* the mass spectrometry service at the Chemistry Research Laboratory, University of Oxford. Samples

for mass spectra were prepared in 1 mg/mL solutions in MeOH or MeCN. m/z values are reported in Daltons (Da) and high-resolution values are calculated to four decimal places from the molecular formula. Melting points were recorded in degrees Celsius (°C) using an Eisco melting point apparatus and are reported uncorrected. Optical rotations were measured on a Perkin Elmer Model 341 polarimeter at 589 nm, 20 °C. $[\alpha]_D^{20}$ is expressed in deg cm^3 g^{-1} dm^{-1} and c is expressed in g 100 cm^{-3}.

DSC experiments were performed on a Waters Thermal Analysers DSC25 coupled with Trios v5.2.0.47555 software for the acquisition of thermal curves and for the analysis of data. Approximately 3−5 mg of each testing sample was placed in a Tzero pan crucible before a Tzero lid was inserted manually. Each crucible was sealed manually with a Tzero press before being heated at 5 °C/min from 25 to 350 °C.

1.2 HPLC yields

HPLC data were obtained using an Agilent Technologies 1200 series HPLC Hypersol ODS, 5 µm column (100 × 4.0 mm) using an extended 20 min method H_2O (0.1 % v/v H_3PO_4): MeCN (10 – 95% (17 min) to 100% (18.5 min) to 10% (20 min)). HPLC yields for reaction optimization were obtained *via* a standard calibration curve using 'authentic' desired product and 1,3,5-triisopropylbenzene as an internal standard. Method validation was achieved by product isolation, giving a result of +/- 2%.

2. Preparation of Starting Materials

2.1 Preparation of Starting Materials for the synthesis of *N*-Alkoxysulfurdiimides

Sulfonamide **S1a**, carbamates **S1c** and **S1d,** amides **S1g** and **S1h** and amine **S1j** were all purchased from commercial suppliers. **S1a** and **S1h** were dried in an 80 °C oven overnight before use.

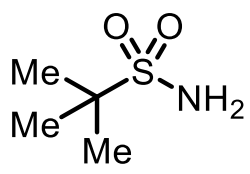

S1a

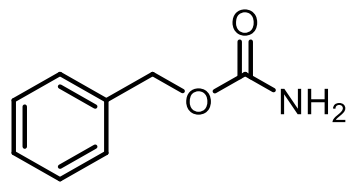

S1c

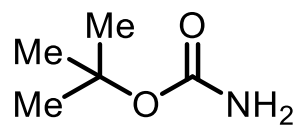

S1d

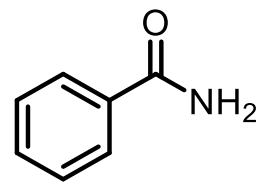

S1g

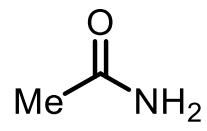

S1h

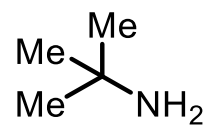

S1j

Sulfonamide **S1b**,[1] carbamates **S1e**[2] and **S1f**,[2] were synthesised according to literature procedures.

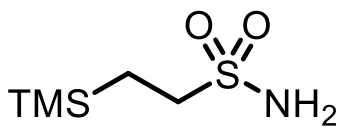

S1b

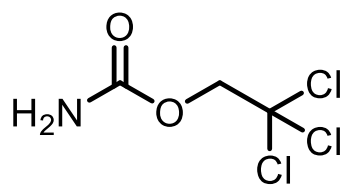

S1e

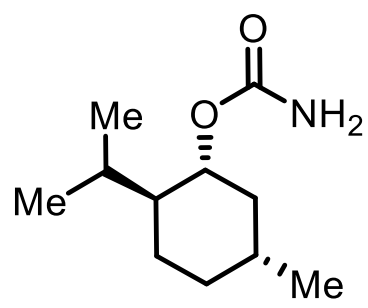

S1f

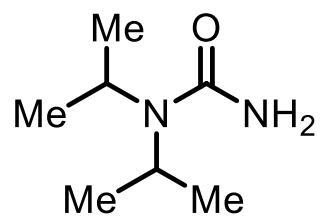

S1i

1,1-Diisopropylurea (S1i):

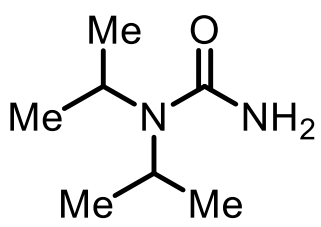

S1i

Diisopropylcarbamoyl chloride (3.27 g, 20.0 mmol, 1.00 equiv.) was dissolved in THF (20.0 mL) and the reaction mixture cooled to 0 °C. Ammonium hydroxide (6.83 mL, 60.0 mmol, 35 wt%) was added slowly and the reaction mixture warmed to rt and stirred for 7 h. The crude reaction mixture was diluted with EtOAc and washed with water. The aqueous phase was extracted with EtOAc (x3). The combined organic extracts were washed with brine, dried over Na_2SO_4, filtered and concentrated *in vacuo* to afford a colourless solid (32%, 0.92 g).

^{1}H NMR (400 MHz, $CDCl_3$) δ_H 4.60 (br. s, 2H), 3.85 (hept, *J* = 7.0 Hz, 2H), 1.23 (d, *J* = 1.9 Hz, 12H).

^{13}C NMR (101 MHz, $CDCl_3$) δ_C 158.0, 45.4, 21.2.

IR (thin film, ν_{max}/cm^{-1}) = 3485, 3335, 3206, 2967, 1651, 1596, 1467, 1453, 1378, 1366.

Data consistent with literature.[3]

2.2 Preparation of Organometallic Reagents

The following commercial Grignard reagents were titrated before use using either salicylaldehyde phenylhydrazone[4] or Knochel method.[5]

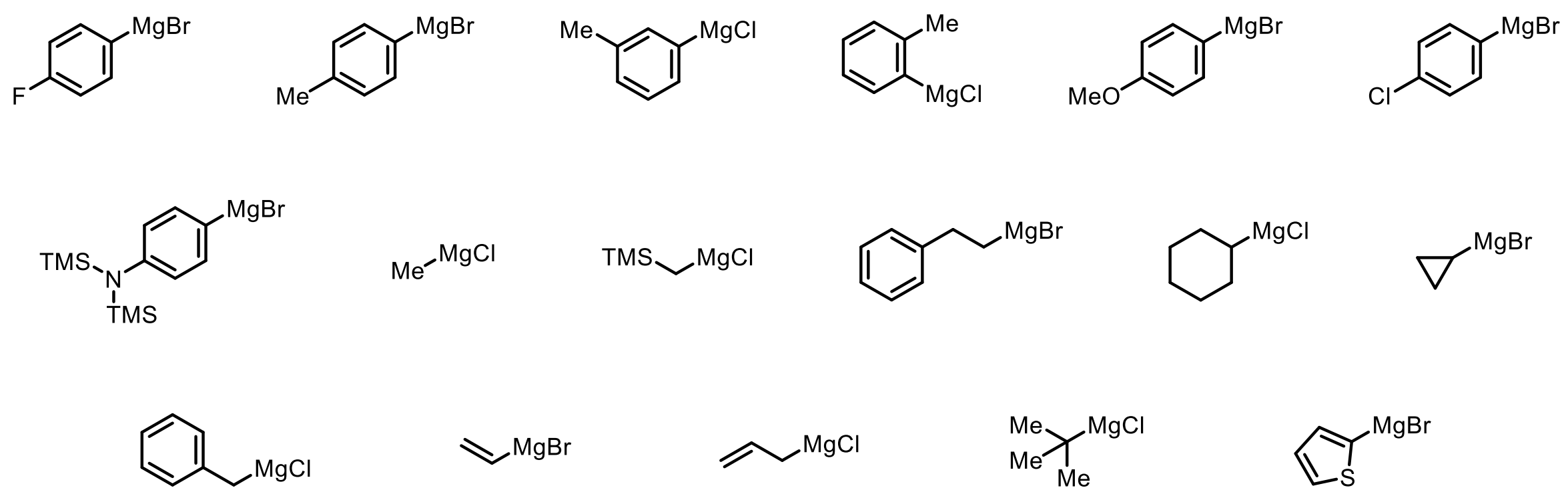

The following Grignard reagents generated from alkyl and (hetero)aryl bromides with magnesium metal were titrated before use using either salicylaldehyde phenylhydrazone[4] or Knochel method.[5]

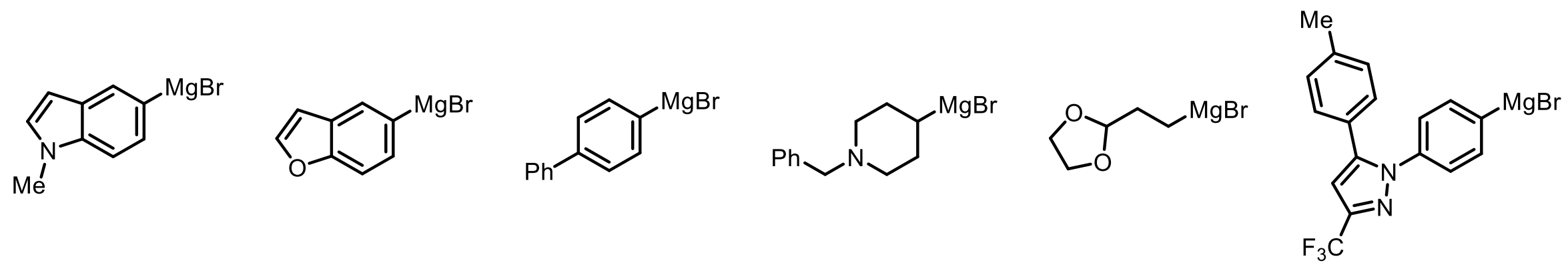

The following organometallic reagents generated from (hetero)aryl halides and *i*-PrMgCl, *i*-PrMgBr, *i*-PrMg·Cl, *n*-BuLi and MeLi were generated in situ and not titrated before use.

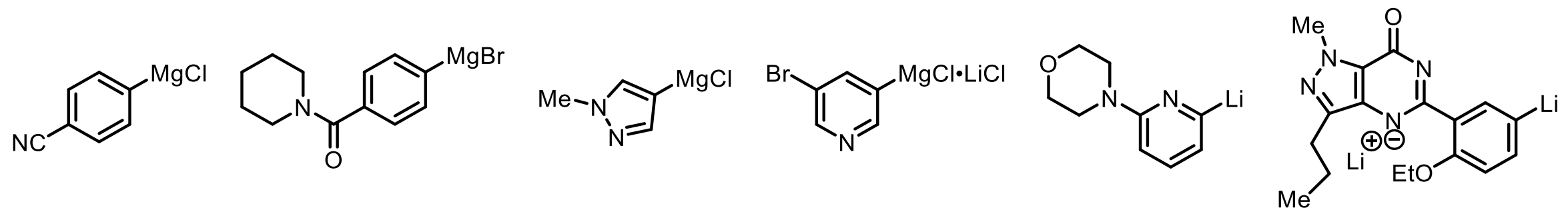

Non-commercial organometallic reagents were synthesised from commercial aryl halides or the relevant aryl halide was prepared via literature procedures.[6]

3. Optimization of Conditions

3.1 Optimization of *t*-BuO-NSN-R reagent synthesis

Table S1: Solvent screening

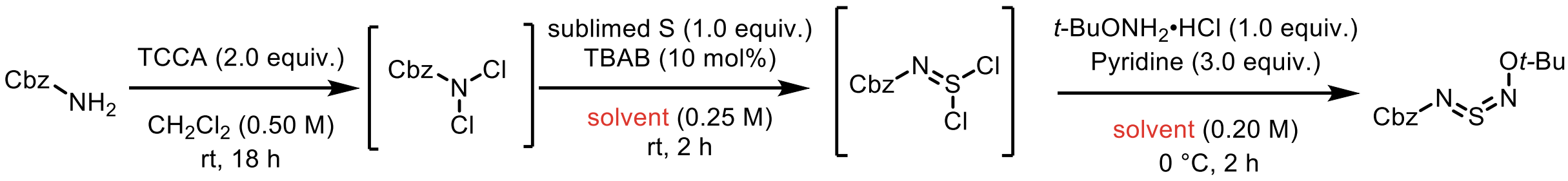

Entry	Solvent	Yield (%)
**1**	**CH_2Cl_2**	**54**
2	$CHCl_3$	3
3	$(CH_2Cl)_2$	58
4	toluene	0
5	THF	0
6	MeCN	46

Reaction conditions: R-NH_2 (1.0 equiv.), TCCA (2.0 equiv.), CH_2Cl_2 (0.50 M), rt, 18 h, *then* sublimed sulfur (1.0 equiv.), TBAB (10 mol%), solvent (0.25 M), rt, 2 h, *then* *t*-BuONH_2·HCl (1.0 equiv.), pyridine (3.0 equiv.), solvent (0.20 M), 0 °C, 2 h. Isolated yields. 5 mmol scale.

Note: Comparable yields were obtained using either dichloromethane, 1,2-dichloroethane or acetonitrile. Dichloromethane selected as the solvent going forward to avoid the requirement of a solvent swap and due to its low-cost availability in its anhydrous form.

Table S2: Solvent concentration for the first step

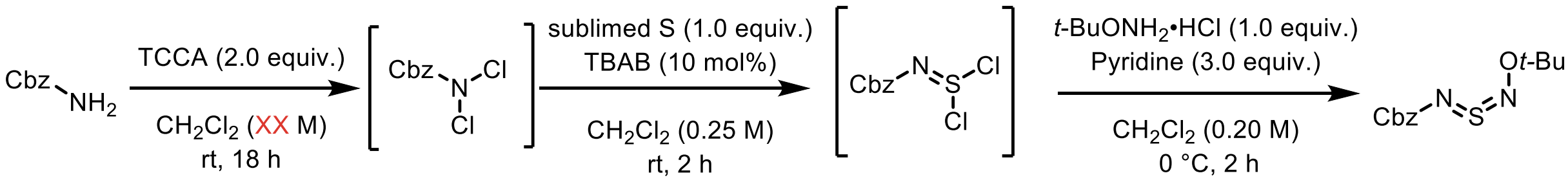

Entry	Solvent Concentation (M)	Yield (%)
**1**	**0.25**	**49**
2	0.50	54

Reaction conditions: R-NH_2 (1.0 equiv.), TCCA (2.0 equiv.), CH_2Cl_2 (XX M), rt, 18 h, *then* sublimed sulfur (1.0 equiv.), TBAB (10 mol%), CH_2Cl_2 (0.25 M), rt, 2 h, *then* *t*-BuONH_2·HCl (1.0 equiv.), pyridine (3.0 equiv.), CH_2Cl_2 (0.20 M), 0 °C, 2 h. Isolated yields. 5 mmol scale.

Note: Whilst 0.25 M concentration gave a slight reduction in yield, it was carried forward due to a reduction in thick slurry formation and increased reaction reproducibility. Additionally, 2.0

equivalents of TCCA were used in order to reproducibly achieve full conversion for the respective *N,N*-dichloramine.

Table S3: Solvent concentration for the second step

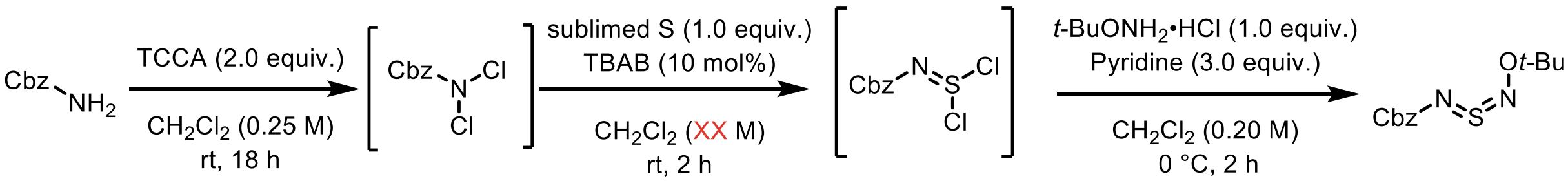

Entry	Solvent Concentation (M)	Yield (%)
1	0.10	47
**2**	**0.25**	**49**
3	0.50	37

Reaction conditions: R-NH_2 (1.0 equiv.), TCCA (2.0 equiv.), CH_2Cl_2 (0.25 M), rt, 18 h, *then* sublimed sulfur (1.0 equiv.), TBAB (10 mol%), CH_2Cl_2 (XX M), rt, 2 h, *then* *t*-BuONH_2·HCl (1.0 equiv.), pyridine (3.0 equiv.), CH_2Cl_2 (0.20 M), 0 °C, 2 h. Isolated yields. 5 mmol scale.

Table S4: Temperature screening for the third step

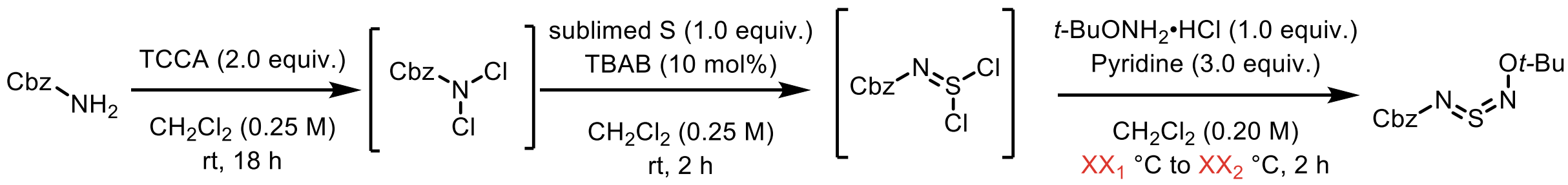

Entry	Temperature 1 (°C)	Temperature 2 (°C)	Yield (%)
1	0	0	49
2	-40	0	58
**3**	**-78**	**0**	**66**
4	-78	rt	63

Reaction conditions: R-NH_2 (1.0 equiv.), TCCA (2.0 equiv.), CH_2Cl_2 (0.50 M), rt, 18 h, *then* sublimed sulfur (1.0 equiv.), TBAB (10 mol%), solvent (0.25 M), rt, 2 h, *then* *t*-BuONH_2·HCl (1.0 equiv.), pyridine (3.0 equiv.), solvent (0.20 M), XX_1 °C to XX_2 °C, 2 h. Isolated yields. 5 mmol scale.

Table S5: Time screening

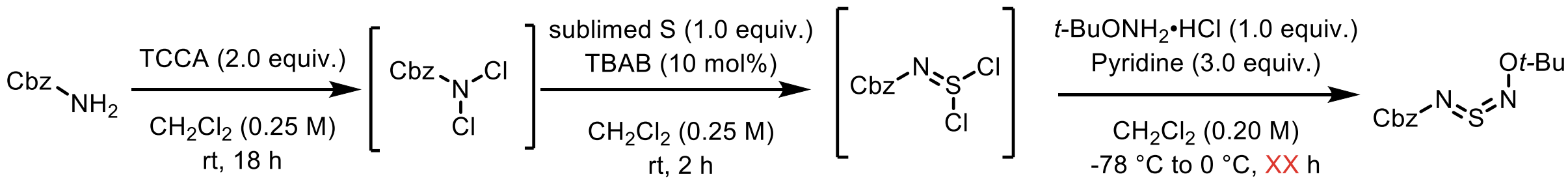

Entry	Time (h)	Yield (%)
1	2	66
2	3	72
3	4	75
**4**	**5**	**77**

Reaction conditions: R-NH_2 (1.0 equiv.), TCCA (2.0 equiv.), CH_2Cl_2 (0.25 M), rt, 18 h, *then* sublimed sulfur (1.0 equiv.), TBAB (10 mol%), solvent (0.25 M), rt, 2 h, *then* *t*-BuONH_2·HCl (1.0 equiv.), pyridine (3.0 equiv.), solvent (0.20 M), -78 °C to 0 °C, XX h. Isolated yields. 5 mmol scale.

3.2 Optimization of Grignard reagent into *t*-BuO-NSN-R reagents

Table S6: Grignard reagent addition temperature screening

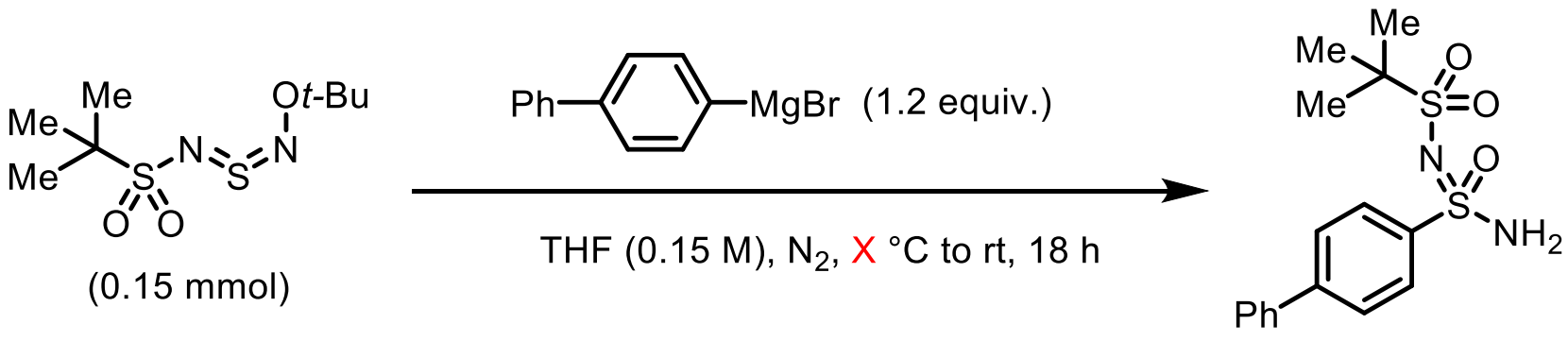

Entry	Addition Temperature (° C)	Yield (%)
1	rt	88
**2**	**0**	**90**
3	-40	90
4	-78	90

Reaction conditions: Bus-NSN-O*t*-Bu (0.15 mmol), 4-fluoromagnesium bromide (0.18 mmol), THF (1 mL), X °C to rt. Yields calculated by HPLC analysis using 1,3,5-triiospropylbenzene as an internal standard.

Note: Whilst comparative yields were obtained when the addition temperature was 0 °C, -40 °C or -78 °C, 0 °C was chosen due to ease of reaction set up.

Table S7: Temperature after Grignard reagent addition screening

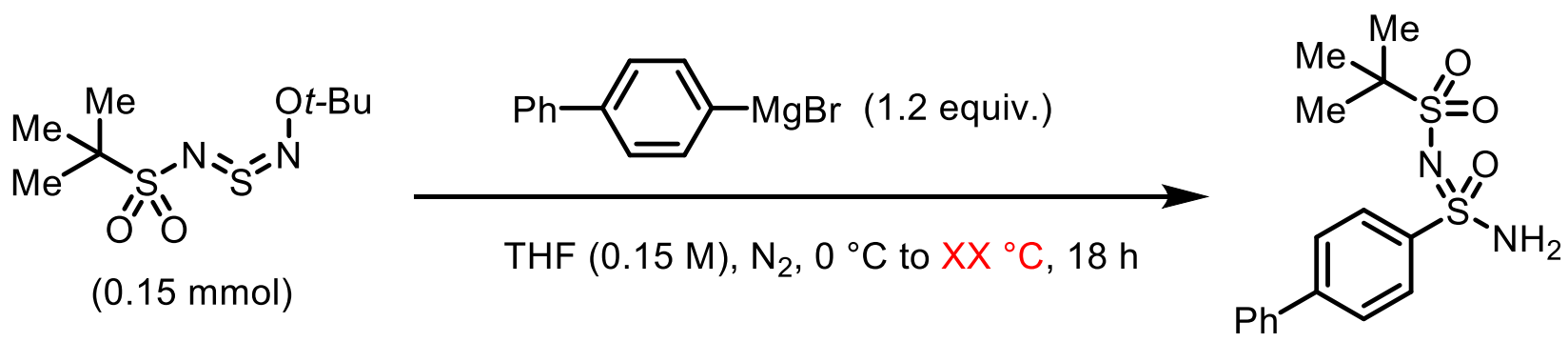

Entry	Temperature After Addition (° C)	Yield (%)
1	rt	89
**2**	**30**	**91 (92)**
3	40	88
4	50	77

Reaction conditions: Bus-NSN-O*t*-Bu (0.15 mmol), 4-fluoromagnesium bromide (0.18 mmol), THF (1 mL), 0 °C to XX °C. Yields calculated by HPLC analysis using 1,3,5-triiospropylbenzene as an internal standard. Isolated yield in brackets.

Table S8: Concentration screening

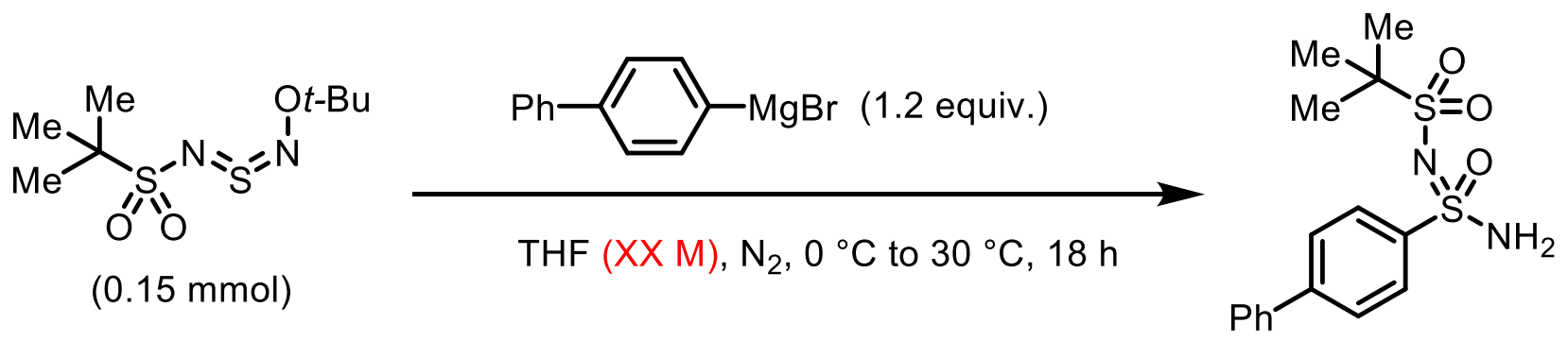

Entry	Solvent Concentration (M)	Yield (%)
1	0.05	94
**2**	**0.10**	**94**
3	0.15	90
4	0.20	85
5	0.30	77

Reaction conditions: Bus-NSN-O*t*-Bu (0.15 mmol), 4-fluoromagnesium bromide (0.18 mmol), THF (XX mL), 0 °C to 30 °C. Yields calculated by HPLC analysis using 1,3,5-triiospropylbenzene as an internal standard.

Table S9: Solvent screening

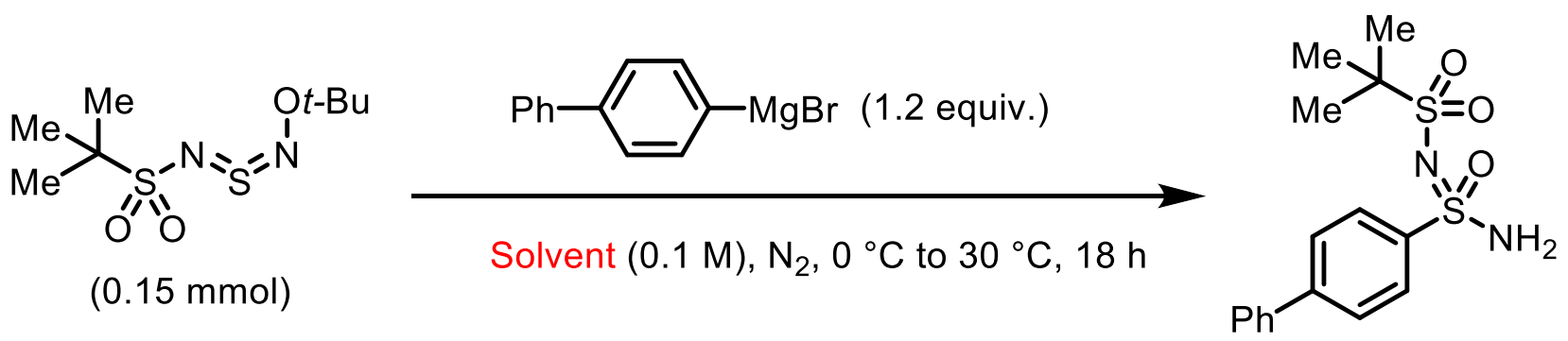

Entry	Solvent	Yield (%)
1	THF	90
2	Et_2O	91
3	2-MeTHF	82
4	DME	23
5	CPME	70

Reaction conditions: Bus-NSN-O*t*-Bu (0.15 mmol), 4-fluoromagnesium bromide (0.18 mmol), Solvent (1.5 mL), 0 °C to 30 °C. Yields calculated by HPLC analysis using 1,3,5-triiospropylbenzene as an internal standard.

Note: Whilst comparable yields were obtained for THF and Et_2O, THF was selected as the choice of solvent going forward due to volatility issues with Et_2O.

Table S10: Grignard reagent equivalents screening

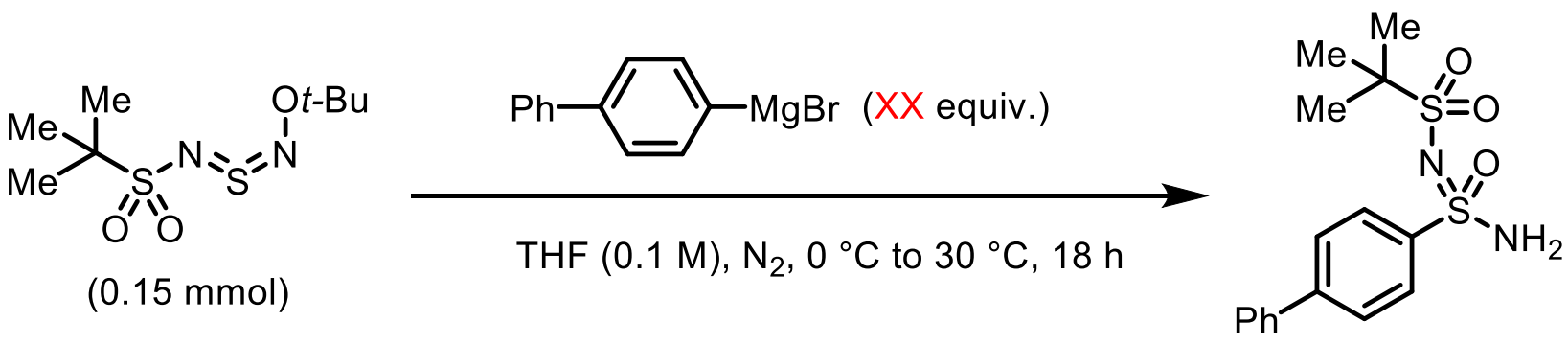

Entry	Equivalents of Grignard Reagent	Yield (%)
1	0.7	57
2	1.0	83
**3**	**1.1**	**96**
4	1.2	96
5	1.5	94
6	2.0	94

Reaction conditions: Bus-NSN-O*t*-Bu (0.15 mmol), 4-fluoromagnesium bromide (XX mmol), THF (1.5 mL), 0 °C to 30 °C. Yields calculated by HPLC analysis using 1,3,5-triiospropylbenzene as an internal standard.

Table S11: Time screening

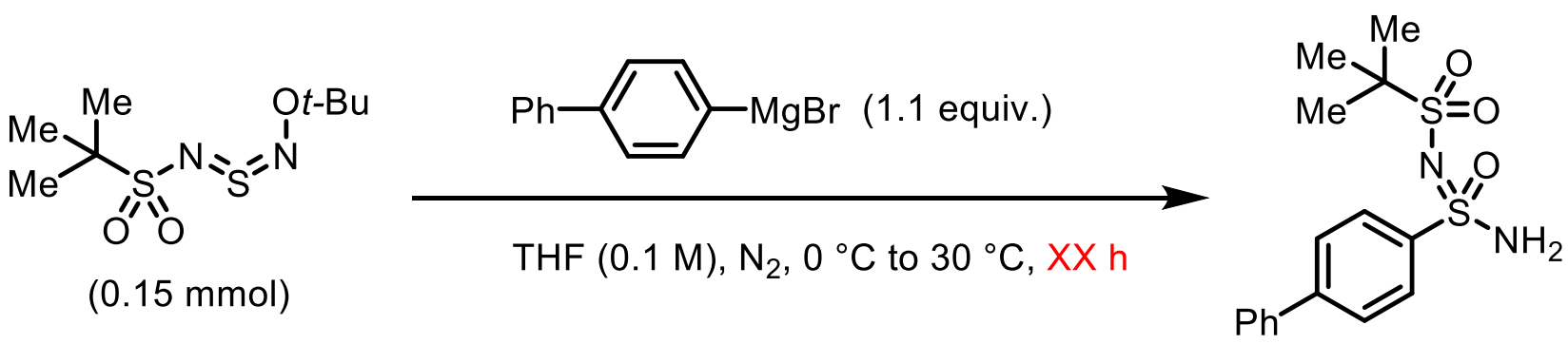

Entry	Time (h)	Yield (%)
1	18	96
2	6	95
3	5	95
4	4	94
5	3	95
6	1	95
7	1	96
**8**	**0.5**	**94 (96)**
9	0.25	94
10	0.167	97
11	0.08	90

Reaction conditions: Bus-NSN-O*t*-Bu (0.15 mmol), 4-fluoromagnesium bromide (0.165 mmol), THF (1.5 mL), 0 °C to 30 °C. Yields calculated by HPLC analysis using 1,3,5-triiospropylbenzene as an internal standard. Isolated yield in brackets.

Note: A reaction time of 30 minutes was selected going forward due to synthetic ease and reaction reproducibility.

Table S12: Temperature after Grignard addition revised

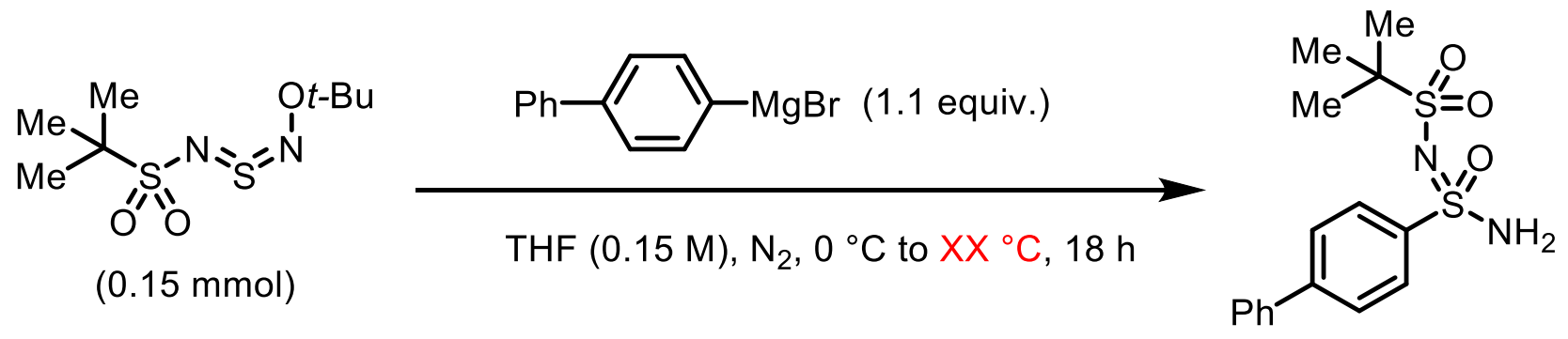

Entry	Temperature After Addition (° C)	Yield (%)
**1**	**rt**	**93 (91)**
2	30	94 (96)

Reaction conditions: Bus-NSN-O*t*-Bu (0.15 mmol), 4-fluoromagnesium bromide (0.165 mmol), THF (1.5 mL), 0 °C to XX ° C. Yields calculated by HPLC analysis using 1,3,5-triiospropylbenzene as an internal standard. Isolated yield in brackets.

Note: Whilst 30 °C gave a slight increase in yield, room temperature was selected going forward due to ease of reaction set up.

4. Experimental Procedures and Data

4.1 Synthesis of *N*-Alkoxysulfurdiimides (1)

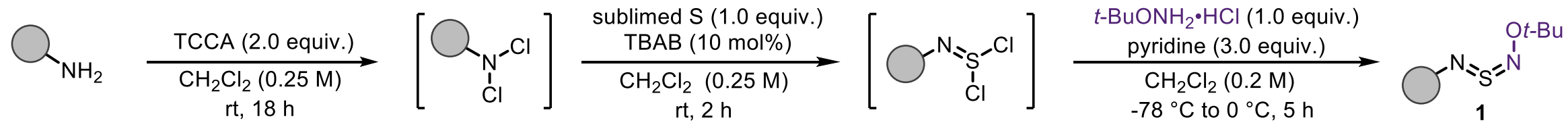

General Procedure A: Amine (1.00 equiv.) and trichloroisocyanuric acid (2.00 equiv.) were added to an oven-dried round-bottom flask, sealed and evacuated and backfilled with N_2 (×3) and dissolved in anhydrous CH_2Cl_2 (0.25 M). The reaction was then vigorously stirred for 18 h (protected from light using aluminium foil).

Upon completion, the suspension was filtered through a sintered funnel into a further oven-dried round-bottom flask, and the solid washed with CH_2Cl_2 (1.00 M). The round-bottom flask containing the filtrate was then sealed, and sparged with nitrogen for 15 minutes.

In a separate oven-dried round-bottom flask, sublimed sulfur (1.00 equiv.) and tetrabutylammonium bromide (10 mol%) were added, and the vessel sealed with a rubber septum, evacuated and backfilled with N_2 (×3). The dichloramine solution was then added and the reaction stirred vigorously for 2 h (protected from light using aluminium foil).

To a separate oven-dried round-bottom flask *O-tert*-butylhydroxylamine hydrochloride (1.00 equiv.) was added, the vessel sealed, evacuated and backfilled with N_2 (×3). Anhydrous CH_2Cl_2 (1.00 M) was added before slow addition of anhydrous pyridine (3.00 equiv.). The hydroxylamine solution was added to the imidothionyl chloride reaction mixture dropwise at –78 °C before warming to 0 °C in an ice-bath and stirring at this temperature for 5 h.

The reaction mixture was concentrated *in vacuo* and the product extracted from the resulting precipitate with ice-cold pentane (~0.05 M). Following filtration, the filtrate was concentrated *in vacuo* to afford the desired product.

Notes:

1) The synthesis of imidothionyl halides have previously been reported by Bolm,[7] Demaerel[8] and Borovikova.[9]
2) Extraction of *N*-alkoxysulfurdiimides from the crude reaction mixture could be aided with sonication, if required.
3) *N*-Alkoxysulfurdiimides can be handled readily for short periods under air for reaction setups without decomposition. Avoidance of contact with air and water or prolonged storage at room temperature is advised.

4) *N*-Alkoxysulfurdiimides were stored under Ar in the freezer (-18 °C) and were stable for > 6 months without decomposition.
5) Sulfur is purchased as sublimed grade from Thermo Scientific Chemicals. Tetrabutylammonium bromide is purchased from Sigma Aldrich and dried under high vacuum for 24 hours before storage in a desiccator.
6) Distilled anhydrous pentane was used to avoid the introduction of grease which was occasionally observed using non-distilled anhydrous pentane. HPLC grade pentane was distilled and stored over 3 Å molecular sieves for 24 h before use.
7) Some *N*-alkoxysulfurdiimides showed lower solubility in pentane after solidification (where relevant).

_N_-((_tert_-Butoxyimino)-λ⁴-sulfaneylidene)-2-methylpropane-2-sulfonamide (1a):

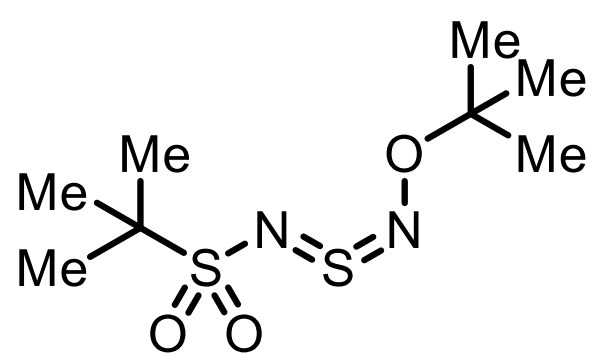

Prepared according to **General Procedure A** using TCCA (2.32 g, 10.0 mmol, 2.00 equiv.), *tert*-butylsulfonamide (0.69 g, 5.00 mmol, 1.00 equiv.), sublimed sulfur (0.16 g, 5.00 mmol, 1.00 equiv.), TBAB (0.16 g, 0.50 mmol, 10 mol%), *O*-*tert*-butylhydroxylamine hydrochloride (0.63 g, 5.00 mmol, 1.00 equiv.) and pyridine (1.20 mL, 15.0 mmol, 3.00 equiv.) in dichloromethane (30 mL total). Purification by extraction from the crude material using ice-cold pentane afforded **1a** as a light-orange solid (0.54 g, 42%).

^{1}H NMR (400 MHz, $CDCl_3$) δ_H 1.48 (s, 9H), 1.46 (s, 9H).

^{13}C NMR (101 MHz, $CDCl_3$) δ_C 90.9, 60.7, 27.3, 24.5.

HRMS (ESI$^+$, m/z) calculated for $[C_8H_{18}N_2O_3S_2Na]^+$ $[M+Na]^+$ 277.0651, found 277.0638.

IR (thin film, ν_{max}/cm^{-1}) = 2987, 1307, 1124, 985, 918, 851, 737.

Melting Point = 69 – 72 °C (*n*-pentane).

N-((tert-Butoxyimino)-λ⁴-sulfaneylidene)-2-(trimethylsilyl)ethane-1-sulfonamide (1b):

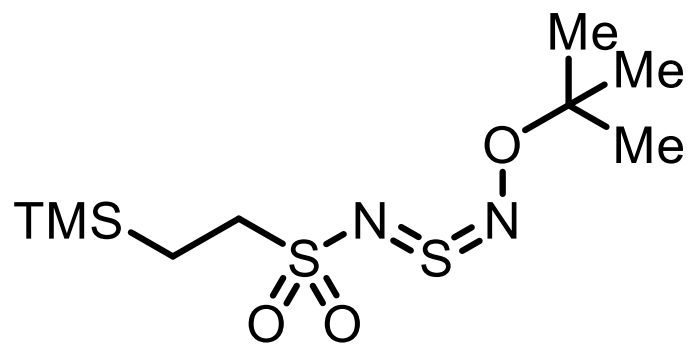

Prepared according to **General Procedure A** using TCCA (2.32 g, 10.0 mmol, 2.00 equiv.), 2-(trimethylsilyl)ethanesulfonamide (0.91 g, 5.00 mmol, 1.00 equiv.), sublimed sulfur (0.16 g, 5.00 mmol, 1.00 equiv.), TBAB (0.16 g, 0.50 mmol, 10 mol%), *O-tert*-butylhydroxylamine hydrochloride (0.63 g, 5.00 mmol, 1.00 equiv.) and pyridine (1.20 mL, 15.0 mmol, 3.00 equiv.) in dichloromethane (30 mL total). Purification by extraction from the crude material using ice-cold pentane afforded **1b** as an orange oil (1.01 g, 68%).

^{1}H NMR (400 MHz, $CDCl_3$) δ_H 3.17 – 3.07 (m, 2H), 1.49 (s, 9H), 1.13 – 1.02 (m, 2H), 0.06 (s, 9H).

^{13}C NMR (101 MHz, $CDCl_3$) δ_C 91.4, 51.9, 27.3, 10.6, -1.8.

HRMS (ESI^+, m/z) calculated for $[C_9H_{22}N_2O_3S_2SiNa]^+$ $[M+Na]^+$ 321.0733, found 321.0727.

IR (thin film, ν_{max}/cm^{-1}) = 2983, 1323, 1140, 988, 860.

Benzyl ((tert-butoxyimino)-λ⁴-sulfaneylidene)carbamate (1c):

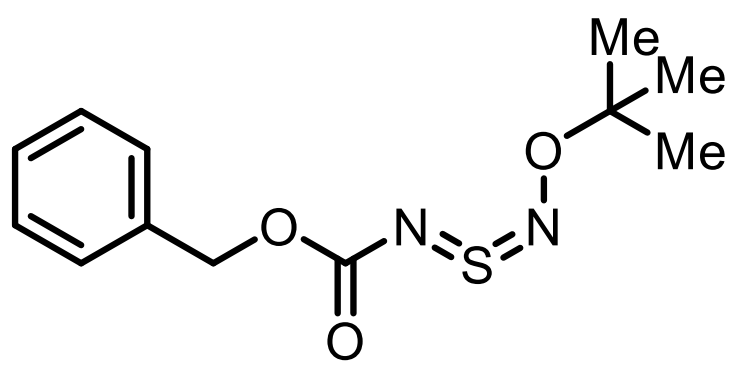

Prepared according to **General Procedure A** using TCCA (2.32 g, 10.0 mmol, 2.00 equiv.), benzyl carbamate (0.75 g, 5.0 mmol, 1.00 equiv.), sublimed sulfur (0.16 g, 5.00 mmol, 1.00 equiv.), TBAB (0.16 g, 0.50 mmol, 10 mol%), *O-tert*-butylhydroxylamine hydrochloride (0.63 g, 5.00 mmol, 1.00 equiv.) and pyridine (1.20 mL, 15.0 mmol, 3.00 equiv.) in dichloromethane (30 mL total). Purification by extraction from the crude material using ice-cold pentane afforded **1c** as a red oil which solidified upon storage (1.03 g, 77%).

A scale up of this reaction was performed according to **General Procedure A** using TCCA (9.30 g, 40.0 mmol, 2.00 equiv.), benzyl carbamate (3.02 g, 20.0 mmol, 1.00 equiv.), sublimed sulfur (0.64 g, 20.0 mmol, 1.00 equiv.), TBAB (0.64 g, 2.00 mmol, 10 mol%), *O-tert*-butylhydroxylamine hydrochloride (2.51 g, 20.0 mmol, 1.00 equiv.) and pyridine (4.83 mL, 60.0 mmol, 3.00 equiv.) in

dichloromethane (120 mL total). Purification by extraction from the crude material using ice-cold pentane afforded **1c** as a red oil which solidified upon storage (3.44 g, 64%).

^{1}H NMR (400 MHz, $CDCl_3$) δ_H 7.44 – 7.28 (m, 5H), 5.20 (s, 2H), 1.45 (s, 9H).

^{13}C NMR (101 MHz, $CDCl_3$) δ_C 158.8, 157.4, 135.8, 128.6, 128.5, 90.4, 68.9, 27.2.

HRMS (ESI$^+$, m/z) calculated for $[C_{12}H_{16}N_2O_3SNa]^+$ $[M+Na]^+$ 291.0774, found 291.0763.

IR (thin film, ν_{max}/cm^{-1}) = 2982, 2930, 1716, 1671, 1371, 1244, 920.

Melting Point = 30 – 31 °C (*n*-pentane).

***tert*-Butyl ((*ter*t-butoxyimino)-λ^4-sulfaneylidene)carbamate (1d):**

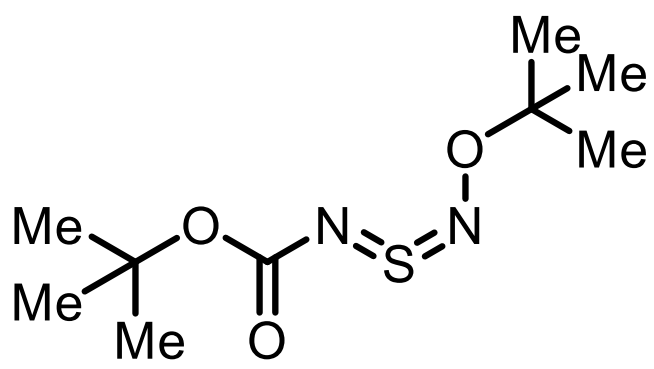

Prepared according to **General Procedure A** using TCCA (2.32 g, 10.0 mmol, 2.00 equiv.), *tert*-butyl carbamate (0.59 g, 5.00 mmol, 1.00 equiv.), sublimed sulfur (0.16 g, 5.00 mmol, 1.00 equiv.), TBAB (0.16 g, 0.50 mmol, 10 mol%), *O*-*tert*-butylhydroxylamine hydrochloride (0.63 g, 5.00 mmol, 1.00 equiv.) and pyridine (1.20 mL, 15.0 mmol, 3.00 equiv.) in dichloromethane (30 mL total). Purification by extraction from the crude material using ice-cold pentane afforded **1d** as a red oil (0.84 g, 71%).

^{1}H NMR (400 MHz, $CDCl_3$) δ_H 1.51 (s, 9H), 1.46 (s, 9H).

^{13}C NMR (101 MHz, $CDCl_3$) δ_C 157.8, 89.8, 82.2, 28.1, 27.3.

HRMS (ESI$^+$, m/z) calculated for $[C_9H_{18}N_2O_3SNa]^+$ $[M+Na]^+$ 257.0930, found 257.0918.

IR (thin film, ν_{max}/cm^{-1}) = 2982, 2936, 1713, 1671, 1370, 1275, 1252, 1157, 934.

2,2,2-Trichloroethyl ((*tert*-Butoxyimino)-λ^4-sulfaneylidene)carbamate (1e):

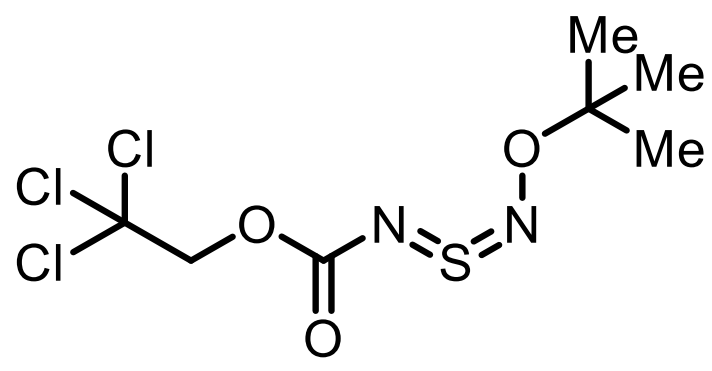

Prepared according to **General Procedure A** using TCCA (2.32 g, 10.0 mmol, 2.00 equiv.), 2,2,2-trichloroethyl carbamate[2] (0.96 g, 5.00 mmol, 1.00 equiv.), sublimed sulfur (0.16 g, 5.0 mmol, 1.00 equiv.), TBAB (0.16 g, 0.5 mmol, 10 mol%), *O*-*tert*-butylhydroxylamine hydrochloride (0.63 g, 5.00 mmol, 1.00 equiv.) and pyridine (1.20 mL, 15.0 mmol, 3.00 equiv.) in dichloromethane (30 mL total). Purification by extraction from the crude material using ice-cold pentane afforded **1e** as an orange crystalline solid (0.87 g, 56%).

^{1}H NMR (400 MHz, $CDCl_3$) δ_H 4.81 (s, 2H), 1.50 (s, 9H).

^{13}C NMR (101 MHz, $CDCl_3$) δ_C 157.1, 95.0, 91.6, 76.2, 27.3.

HRMS (ESI$^+$, m/z) calculated for $[C_7H_{11}Cl_3N_2O_3SNa]^+$ $[M+Na]^+$ 330.9448, found 330.9433.

IR (thin film, ν_{max}/cm^{-1}) = 2983, 1724, 1677, 1370, 1234, 1135, 1054, 918, 843.

Melting Point = 48 – 52 °C (*n*-pentane).

(1*R*,2*S*,5*R*)-2-Isopropyl-5-methylcyclohexyl ((*tert*-butoxyimino)-λ^4-sulfaneylidene)carbamate (1f):

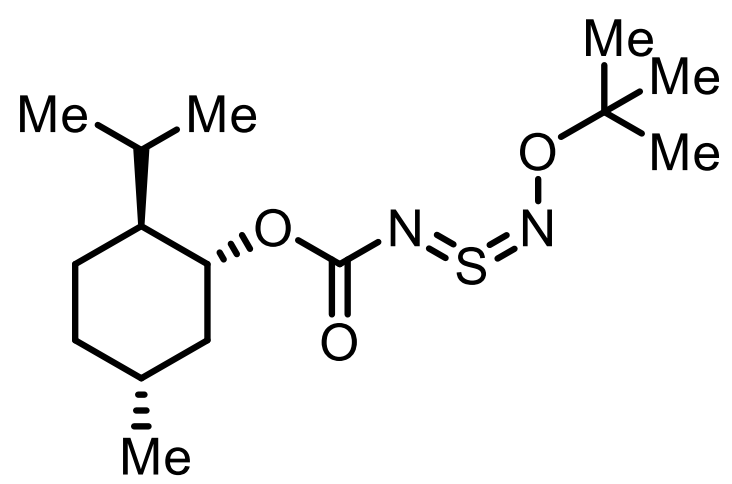

Prepared according to **General Procedure A** using TCCA (2.32 g, 10.0 mmol, 2.00 equiv.), (1*R*,2*S*,5*R*)-2-isopropyl-5-methylcyclohexyl carbamate[2] (0.99 g, 5.00 mmol, 1.00 equiv.), sublimed sulfur (0.16 g, 5.00 mmol, 1.00 equiv.), TBAB (0.16 g, 0.5 mmol, 10 mol%), *O*-*tert*-butylhydroxylamine hydrochloride (0.63 g, 5.00 mmol, 1.00 equiv.) and pyridine (1.20 mL, 15.0 mmol, 3.00 equiv.) in dichloromethane (30 mL total). Purification by extraction from the crude material using ice-cold pentane afforded **1f** as an orange oil (1.36 g, 85%).

^{1}H NMR (400 MHz, $CDCl_3$) δ_H 4.71 – 4.56 (m, 1H), 2.15 – 2.06 (m, 1H), 2.06 – 1.94 (m, 1H), 1.73 – 1.62 (m, 2H), 1.47 (s, 9H), 1.43 – 1.38 (m, 1H), 1.34 – 1.19 (m, 2H), 1.14 – 0.97 (m, 2H), 0.96 – 0.83 (m, 6H), 0.83 – 0.73 (m, 3H).

^{13}C NMR (101 MHz, $CDCl_3$) δ_C 158.8, 90.2, 47.1, 40.9, 34.3, 31.6, 27.3, 26.1, 23.4, 22.2, 21.0, 16.3.

HRMS (ESI$^+$, m/z) calculated for $[C_{15}H_{28}N_2O_3SNa]^+$ $[M+Na]^+$ 339.1713, found 339.1702.

IR (thin film, ν_{max}/cm^{-1}) = 2955, 2930, 2870, 1711, 1665, 1456, 1370, 1243, 1179, 1033, 933, 914.

$[\alpha]_D^{20}$ = – 50.6 (c = 1.0, $CHCl_3$).

***N*-((*tert*-Butoxyimino)-λ^4-sulfaneylidene)benzamide (1g):**

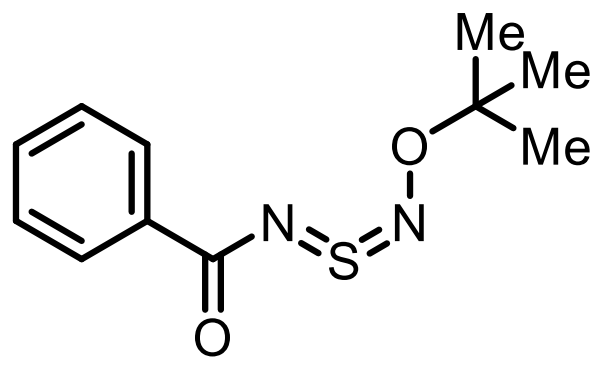

Prepared according to **General Procedure A** using TCCA (2.32 g, 10.0 mmol, 2.00 equiv.), benzamide (0.61 g, 5.00 mmol, 1.00 equiv.), sublimed sulfur (0.16 g, 5.00 mmol, 1.00 equiv.), TBAB (0.16 g, 0.5 mmol, 10 mol%), *O*-*tert*-butylhydroxylamine hydrochloride (0.63 g, 5.00 mmol, 1.00 equiv.) and pyridine (1.20 mL, 15.0 mmol, 3.00 equiv.) in dichloromethane (30 mL total). Purification by extraction from the crude material using ice-cold pentane afforded **1g** as yellow solid (0.78 g, 65%).

^{1}H NMR (400 MHz, $CDCl_3$) δ_H 8.15 – 8.09 (m, 2H), 7.58 – 7.50 (m, 1H), 7.47 – 7.40 (m, 2H), 1.48 (s, 9H).

^{13}C NMR (101 MHz, $CDCl_3$) δ_C 173.5, 134.3, 133.0, 130.2, 128.4, 90.1, 27.3.

IR (thin film, ν_{max}/cm^{-1}) = 2982, 1679, 1630, 1370, 1265, 1172, 1049, 1023, 935, 923, 707.

HRMS (ESI$^+$, m/z) calculated for $[C_{11}H_{14}N_2O_2SNa]^+$ $[M+Na]^+$ 261.0668, found 261.0656.

Melting Point = 58 – 60 °C (*n*-pentane).

N-((*tert*-Butoxyimino)-λ^4-sulfaneylidene)acetamide (1h):

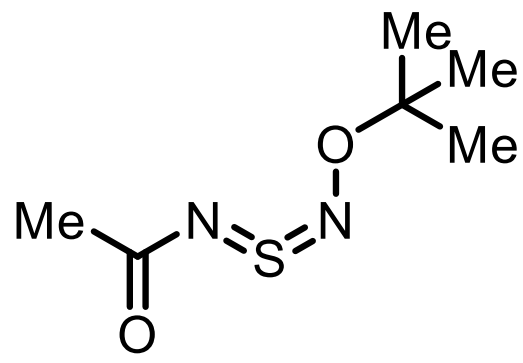

Prepared according to **General Procedure A** using TCCA (2.32 g, 10.0 mmol, 2.00 equiv.), acetamide (0.30 g, 5.00 mmol, 1.00 equiv.), sublimed sulfur (0.16 g, 5.00 mmol, 1.00 equiv.), TBAB (0.16 g, 0.5 mmol, 10 mol%), *O-tert*-butylhydroxylamine hydrochloride (0.63 g, 5.00 mmol, 1.00 equiv.) and pyridine (1.20 mL, 15.0 mmol, 3.00 equiv.) in dichloromethane (30 mL total). Purification by extraction from the crude material using ice-cold pentane afforded **1h** as red oil (0.46 g, 52%).

Note: *N*-alkoxysulfurdiimide **1h** was found to be volatile. For concentration of solvents using a rotary evaporator, the bath temperature was set to 30 °C and the pressure not set below 200 mbar.

^{1}H NMR (400 MHz, $CDCl_3$) δ_H 2.26 (s, 3H), 1.47 (s, 9H).

^{13}C NMR (101 MHz, $CDCl_3$) δ_C 179.6, 90.2, 27.3, 26.5.

IR (thin film, ν_{max}/cm^{-1}) = 2981, 2927, 1682, 1455, 1368, 1229, 1181, 999, 964, 914.

HRMS (ESI$^+$, m/z) calculated for $[C_6H_{12}N_2O_2SNa]^+$ $[M+Na]^+$ 199.0512, found 199.0505.

3-((*tert*-Butoxyimino)-λ^4-sulfaneylidene)-1,1-diisopropylurea (1i):

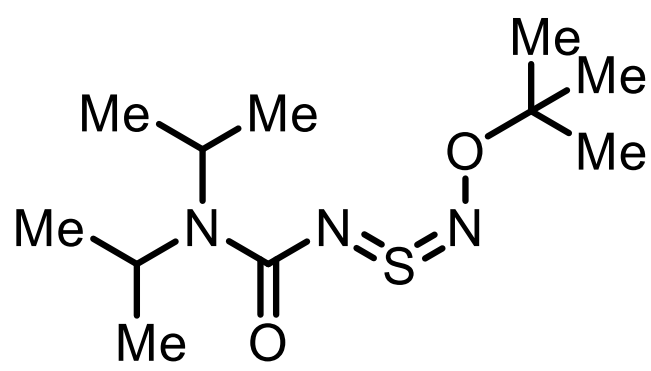

Prepared according to **General Procedure A** using TCCA (2.32 g, 10.0 mmol, 2.00 equiv.), 1,1-diisopropylurea (0.72 g, 5.00 mmol, 1.00 equiv.), sublimed sulfur (0.16 g, 5.00 mmol, 1.00 equiv.), TBAB (0.16 g, 0.5 mmol, 10 mol%), *O-tert*-butylhydroxylamine hydrochloride (0.63 g, 5.00 mmol, 1.00 equiv.) and pyridine (1.20 mL, 15.0 mmol, 3.00 equiv.) in dichloromethane (30 mL total). Purification by extraction from the crude material using ice-cold pentane afforded **1i** as a red oil (1.07 g, 82%).

^{1}H NMR (400 MHz, $CDCl_3$) δ_H 4.08 (s, 1H), 3.97 (s, 1H), 1.42 (s, 9H), 1.34 – 1.26 (m, 6H), 1.25 – 1.20 (m, 6H).

^{13}C NMR (101 MHz, $CDCl_3$) δ_C 158.5, 87.2, 47.9, 45.9, 27.3, 21.4, 20.6.

HRMS (ESI$^+$, m/z) calculated for $[C_{11}H_{24}N_3O_2S]^+$ $[M+H]^+$ 262.1584, found 262.1592.

IR (thin film, ν_{max}/cm^{-1}) = 2974, 2932, 1655, 1619, 1610, 1427, 1368, 1313, 1296, 1013, 939.

***O*-(*tert*-Butyl)-*N*-((*tert*-butylimino)-λ^4-sulfaneylidene)hydroxylamine (1j):**

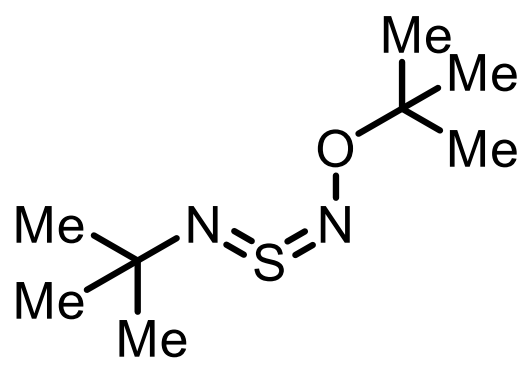

Prepared according to **General Procedure A** using TCCA (2.32 g, 10.0 mmol, 2.00 equiv.), *tert*-butylamine (0.53 mL, 5.00 mmol, 1.00 equiv.), sublimed sulfur (0.16 g, 5.00 mmol, 1.00 equiv.), TBAB (0.16 g, 0.5 mmol, 10 mol%), *O-tert*-butylhydroxylamine hydrochloride (0.63 g, 5.00 mmol, 1.00 equiv.) and pyridine (1.20 mL, 15.0 mmol, 3.00 equiv.) in dichloromethane (30 mL total). Purification by extraction from the crude material using ice-cold pentane afforded **1j** as an orange oil (0.81 g, 85%).

^{1}H NMR (400 MHz, $CDCl_3$) δ_H 1.44 (s, 9H), 1.33 (s, 9H).

^{13}C NMR (101 MHz, $CDCl_3$) δ_C 83.1, 59.0, 33.2, 27.1.

HRMS (ESI$^+$, m/z) calculated for $[C_8H_{18}N_2OSNa]^+$ $[M+Na]^+$ 213.1032, found 213.1040.

IR (thin film, ν_{max}/cm^{-1}) = 2975, 1365, 1223, 1185, 1014, 870, 858.

4.2 Differential Scanning Calorimetry tests for *N*-Alkoxysulfurdiimides 1c and 1i

Differential Scanning Calorimetry (DSC) tests indicate that for *N*-alkoxysulfurdiimide **1c** an exotherm is observed at 79.33 °C (**Figure S1**). Whilst no explosions have been observed over the course of normal use, heating of *N*-alkoxysulfurdiimide **1c** is not advised.

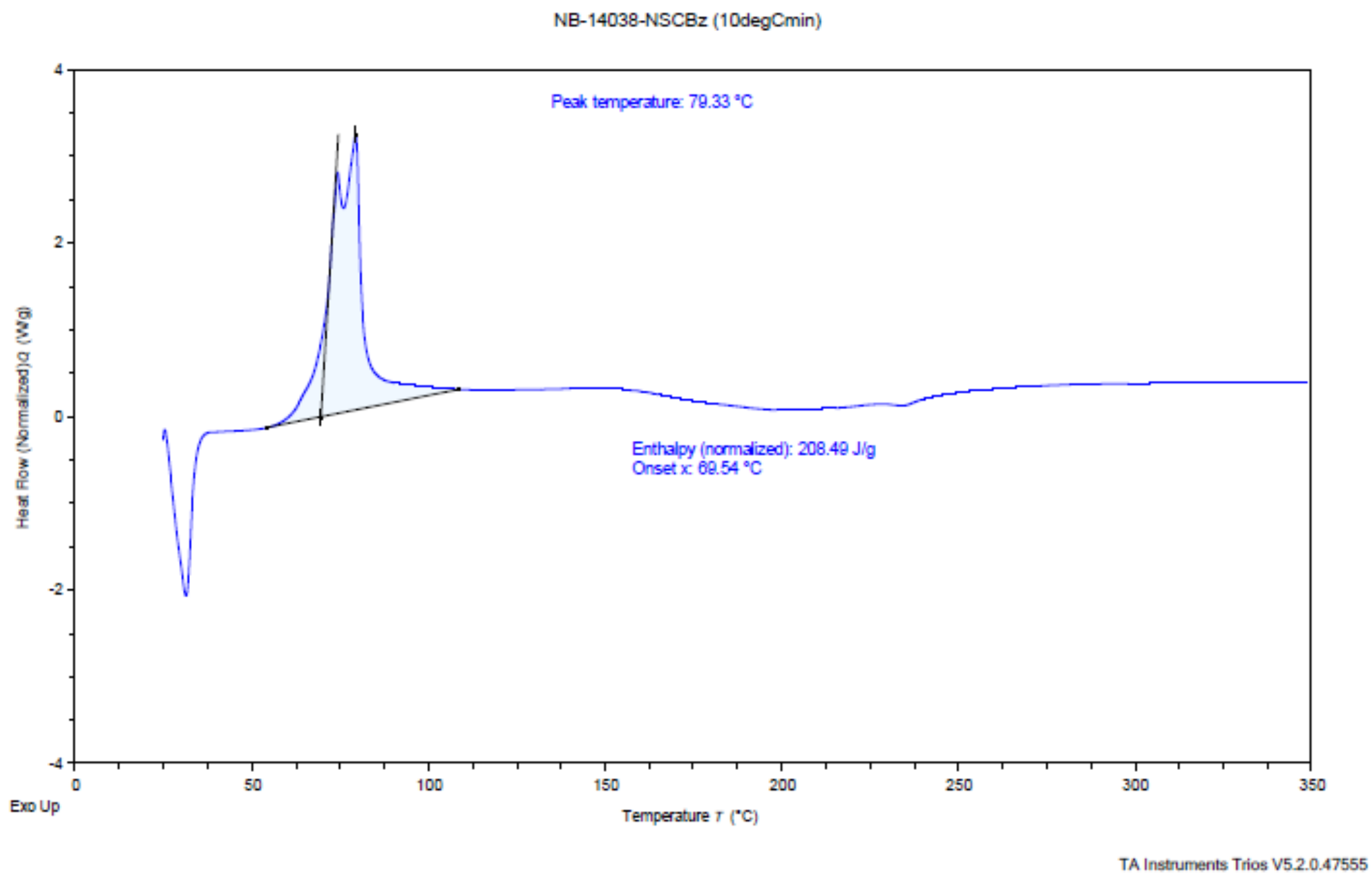

Figure S1: Thermogram for *N*-alkoxysulfurdiimide **1c** when heated between 25 and 350 °C.

For *N*-alkoxysulfurdiimide **1i** no exotherm was observed between 25 and 350 °C (**Figure S2**).

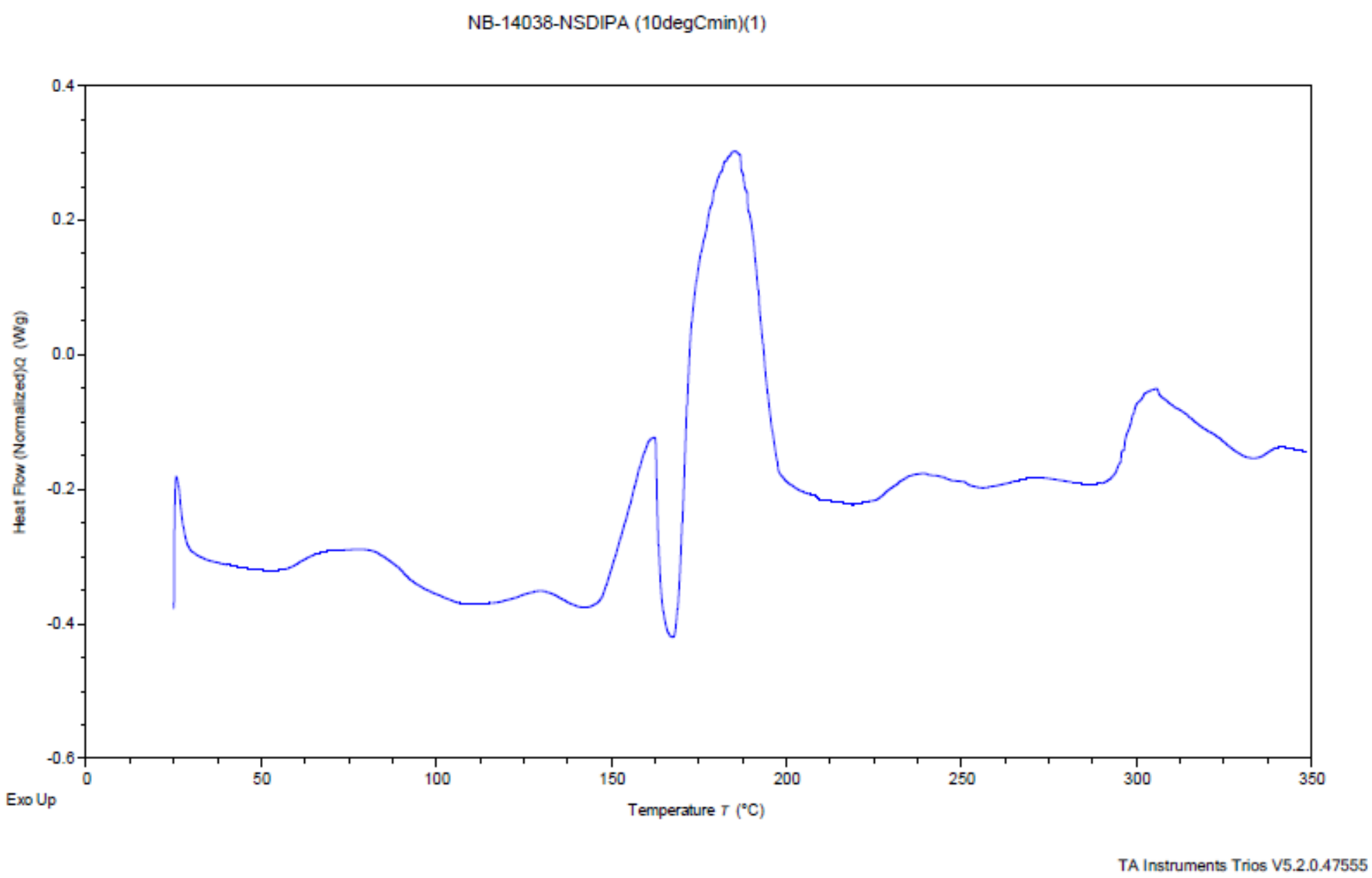

Figure S2: Thermogram for *N*-alkoxysulfurdiimide **1i** when heated between 25 and 350 °C.

4.3 Reaction of *N*-alkoxysulfurdiimide reagents 1a-j with Grignard reagents

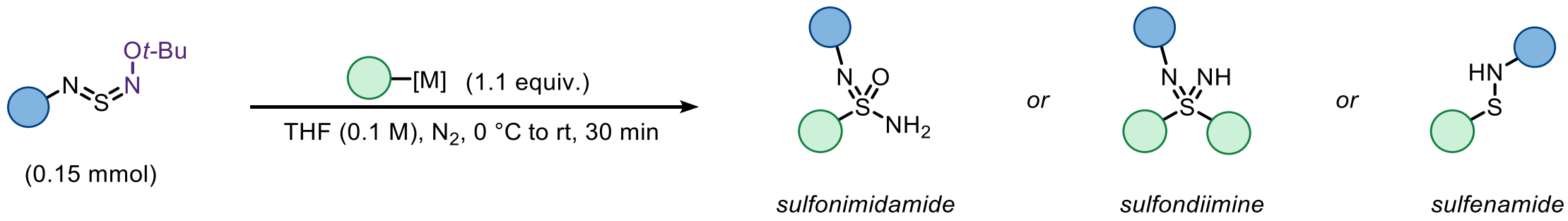

General Procedure B: The corresponding *N*-alkoxysulfurdiimide (1.00 equiv.) was added to an oven-dried reaction vessel. The vessel was then sealed with a rubber septum, evacuated and backfilled with N_2 (×3). Dry THF (0.10 M) was added and the reaction mixture cooled to 0 °C. The corresponding organometallic reagent (1.10 equiv.) was added dropwise and the reaction warmed to rt in a water bath and stirred for 30 mins. The reaction was quenched with silica and the crude product was purified directly by flash column chromatography with the appropriate solvent system.

***N*-(Amino(4-fluorophenyl)(oxo)-λ^6-sulfaneylidene)-2-methylpropane-2-sulfonamide (2a):**

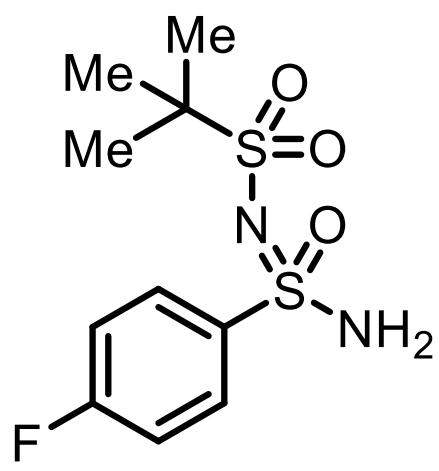

Prepared according to **General Procedure B** using *N*-alkoxysulfurdiimide **1a** (38.2 mg, 0.150 mmol, 1.00 equiv.), THF (1.50 mL) and 4-fluorophenylmagnesium bromide solution (0.17 mL, 0.165 mmol, 0.97 M in THF, 1.10 equiv.). Flash column chromatography (0% – 30% acetone in pentane) afforded sulfonimidamide **2a** as a white solid (33.7 mg, 76%).

R_f = 0.10 (20% acetone in pentane).

^{1}H NMR (400 MHz, $(CD_3)_2CO$) δ_H 8.14 – 8.06 (m, 2H), 7.44 – 7.35 (m, 2H), 7.16 (br. s, 2H), 1.36 (s, 9H).

^{13}C NMR (101 MHz, $(CD_3)_2CO$) δ_C 166.0 (d, *J* = 252.1 Hz), 141.1 (d, *J* = 3.0 Hz), 130.7 (d, *J* = 9.6 Hz), 117.0 (d, *J* = 23.0 Hz), 59.7, 24.5.

^{19}F NMR (377 MHz, $(CD_3)_2CO$) δ_F -107.8 (tt, *J* = 13.2, 5.0 Hz).

HRMS (ESI$^+$, m/z) calculated for $[C_{10}H_{15}FN_2O_3S_2]^+$ $[M+H]^+$ 295.0581, found 295.0563.

IR (thin film, ν_{max}/cm^{-1}) = 3261, 2995, 1700, 1590, 1495, 1277, 1239, 1116, 1080, 839.

Melting Point = 172 – 178 °C (MeOH).

***N*-(Amino(4-fluorophenyl)(oxo)-λ^6-sulfaneylidene)-2-(trimethylsilyl)ethane-1-sulfonamide (2b):**

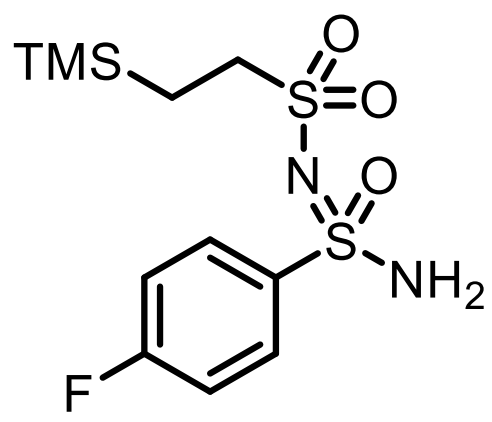

Prepared according to **General Procedure B** using *N*-alkoxysulfurdiimide **1b** (44.8 mg, 0.150 mmol, 1.00 equiv.), THF (1.50 mL) and 4-fluorophenylmagnesium bromide solution (0.17 mL, 0.165 mmol, 1.0 M in THF, 1.10 equiv.). Flash column chromatography (0% – 50% EtOAc in pentane) afforded sulfonimidamide **2b** as a colourless solid (24.4 mg, 48%).

R$_f$ = 0.23 (30% EtOAc in pentane).

^{1}H NMR (400 MHz, $CDCl_3$) δ_H 8.12 – 8.04 (m, 2H), 7.28 – 7.22 (m, 2H), 5.66 (br. s, 2H), 3.24 – 3.10 (m, 2H), 1.24 – 1.08 (m, 2H), 0.06 (s, 9H).

^{13}C NMR (101 MHz, $CDCl_3$) δ_C 165.9 (d, *J* = 251.5 Hz), 136.8 (d, *J* = 3.5 Hz), 130.2 (d, *J* = 9.6 Hz), 116.7 (d, *J* = 23.0 Hz), 53.4, 10.6, -1.8.

^{19}F NMR (377 MHz, $CDCl_3$) δ_F -103.1 (tt, *J* = 8.7, 4.6 Hz).

HRMS (ESI$^+$, m/z) calculated for $[C_{11}H_{19}FN_2O_3S_2SiNa]^+$ $[M+Na]^+$ 361.0483, found 361.0481.

IR (thin film, ν_{max}/cm^{-1}) = 3221, 1311, 1253, 1139, 1114, 842.

Melting Point = 141 – 144 °C ($CHCl_3$).

***N*-((4-Fluorophenyl)thio)-2-(trimethylsilyl)ethane-1-sulfonamide (2b'):**

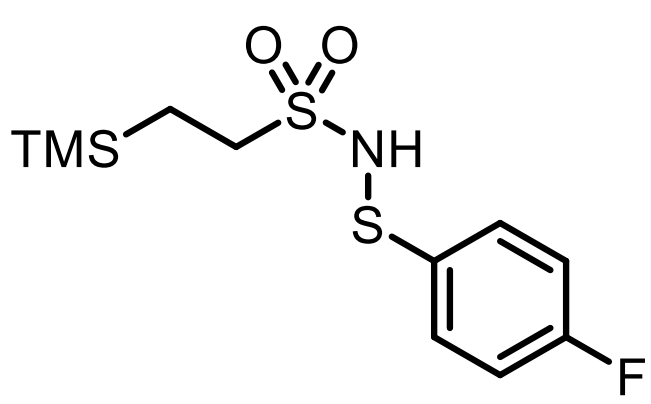

Prepared according to **General Procedure B** using *N*-alkoxysulfurdiimide **1b** (44.8 mg, 0.150 mmol, 1.00 equiv.), THF (1.50 mL) and 4-fluorophenylmagnesium bromide solution (0.17 mL, 0.165 mmol, 1.0 M in THF, 1.10 equiv.). Flash column chromatography (0% – 10% EtOAc in pentane) afforded sulfenamide **2b'** as a colourless solid (21.5 mg, 47%).

R_f = 0.25 (10% EtOAc in pentane).

^{1}H NMR (400 MHz, $CDCl_3$) δ_H 7.59 – 7.52 (m, 2H), 7.12 – 7.04 (m, 2H), 5.81 (br. s, 1H), 3.10 – 2.98 (m, 2H), 1.02 – 0.89 (m, 2H), 0.02 (s, 9H).

^{13}C NMR (101 MHz, $CDCl_3$) δ_C 163.2 (d, *J* = 252.5 Hz), 132.8 (d, *J* = 3.4 Hz), 131.0 (d, *J* = 8.4 Hz), 116.6 (d, *J* = 22.3 Hz), 48.8, 10.6, -1.9.

^{19}F NMR (377 MHz, $CDCl_3$) δ_F -112.0 (tt, *J* = 8.5, 5.1 Hz).

HRMS (ESI$^+$, m/z) calculated for $[C_{11}H_{18}FNO_2S_2SiNa]^+$ $[M+Na]^+$ 330.0425, found 330.0423.

IR (thin film, ν_{max}/cm^{-1}) = 3236, 2953, 1492, 1253, 1146, 862.

Melting Point = 103 – 105 °C ($CHCl_3$).

Benzyl (amino(4-fluorophenyl)(oxo)-λ^6-sulfaneylidene)carbamate (2c):

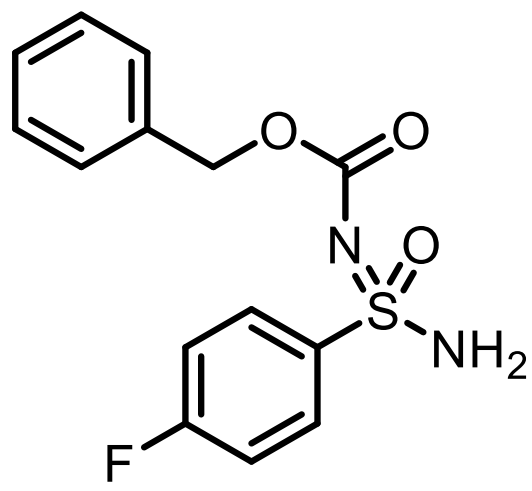

Prepared according to **General Procedure B** using *N*-alkoxysulfurdiimide **1c** (40.3 mg, 0.150 mmol, 1.00 equiv.), THF (1.50 mL) and 4-fluorophenylmagnesium bromide solution (0.18 mL, 0.165 mmol, 0.93 M in THF, 1.10 equiv.). Flash column chromatography (20% – 50% EtOAc in pentane) afforded sulfonimidamide **2c** as a colourless solid (37.1 mg, 80%).

R_f = 0.10 (30% EtOAc in pentane).

^{1}H NMR (400 MHz, $(CD_3)_2SO$) δ_H 7.91 (dd, *J* = 8.9, 3.7 Hz, 2H), 7.84 (br. s, 2H), 7.43 (app. t, *J* = 9.0 Hz, 2H), 7.35 – 7.26 (m, 3H), 7.23 – 7.18 (m, 2H), 4.92 (d, *J* = 12.7 Hz, 1H), 4.87 (d, *J* = 12.6 Hz, 1H).

^{13}C NMR (101 MHz, $(CD_3)_2SO$) δ_C 164.1 (d, *J* = 250.5 Hz), 157.0, 138.7 (d, *J* = 2.9 Hz), 136.9, 129.8 (d, *J* = 9.5 Hz), 128.3, 127.6, 127.5, 116.0 (d, *J* = 22.9 Hz), 66.1.

^{19}F NMR (377 MHz, $(CD_3)_2SO$) δ_F -107.1 (tt, *J* = 9.1, 5.1 Hz).

HRMS (ESI$^+$, m/z) calculated for $[C_{14}H_{13}FN_2O_3SNa]^+$ $[M+Na]^+$ 331.0523, found 331.0538.

IR (thin film, ν_{max}/cm^{-1}) = 1727, 1672, 1494, 1276, 1099, 760.

Melting Point = 121 – 124 °C (acetone).

***tert*-Butyl (amino(4-fluorophenyl)(oxo)-λ⁶-sulfaneylidene)carbamate (2d):**

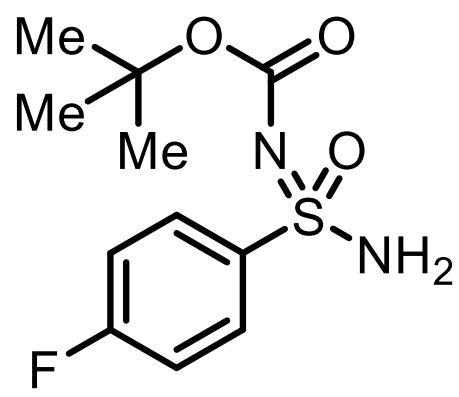

Prepared according to **General Procedure B** using *N*-alkoxysulfurdiimide **1d** (35.1 mg, 0.150 mmol, 1.00 equiv.), THF (1.50 mL) and 4-fluorophenylmagnesium bromide solution (0.17 mL, 0.165 mmol, 0.97 M in THF, 1.10 equiv.). Flash column chromatography (0% – 30% EtOAc in pentane) afforded sulfonimidamide **2d** as a white solid (24.5 mg, 60%).

$\mathbf{R_f}$ = 0.15 (30% EtOAc in pentane).

^{1}H NMR (400 MHz, $CDCl_3$) δ_H 8.02 (m, 2H), 7.19 (ddd, *J* = 9.0, 7.9, 0.9 Hz, 2H), 6.08 (br. s, 2H), 1.35 (s, 9H).

^{13}C NMR (101 MHz, $CDCl_3$) δ_C 165.5 (d, *J* = 255.8 Hz), 157.0, 137.1 (d, *J* = 2.9 Hz), 130.3 (d, *J* = 9.5 Hz), 116.4 (d, *J* = 22.9 Hz), 81.2, 28.1.

^{19}F NMR (377 MHz, $CDCl_3$) δ_F -104.4 (tt, *J* = 8.2, 4.2 Hz).

HRMS (ESI^+, m/z) calculated for $[C_{11}H_{15}FN_2O_3SNa]^+$ $[M+Na]^+$ 297.0680, found 297.0686.

IR (thin film, ν_{max}/cm^{-1}) = 3236, 1670, 1592, 1495, 1369, 1291, 1241, 1156, 841, 737.

Melting Point = 136 – 140 °C ($CHCl_3$) (decomp.).

2,2,2-Trichloroethyl ((4-Fluorophenyl)thio)carbamate (2e):

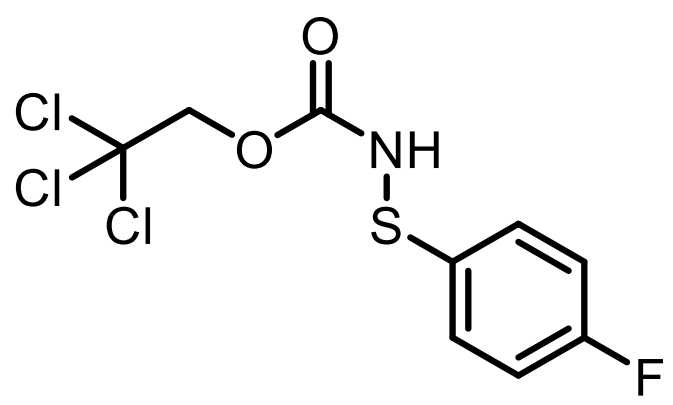

Prepared according to **General Procedure B** using *N*-alkoxysulfurdiimide **1e** (46.4 mg, 0.150 mmol, 1.00 equiv.), THF (1.50 mL) and 4-fluorophenylmagnesium bromide solution (0.17 mL, 0.165 mmol, 1.0 M in THF, 1.10 equiv.). Flash column chromatography (0% – 10% EtOAc in pentane) afforded sulfenamide **2e** as a colourless oil (8.2 mg, 17%).

R_f = 0.28 (10% EtOAc in pentane).

^{1}H NMR (400 MHz, $CDCl_3$) δ_H 7.52 – 7.44 (m, 2H), 7.06 (dd, *J* = 9.6, 7.7 Hz, 2H), 6.19 (br. s, 1H), 4.80 (s, 2H).

^{13}C NMR (101 MHz, $CDCl_3$) δ_C 166.6 (d, *J* = 280.5 Hz), 150.0, 137.7 (d, *J* = 8.5 Hz), 121.9 (d, *J* = 3.6 Hz), 116.7 (d, *J* = 22.3 Hz), 94.2, 75.2.

^{19}F NMR (377 MHz, $CDCl_3$) δ_F -112.4 (tt, *J* = 13.4, 5.1 Hz).

IR (thin film, ν_{max}/cm^{-1}) = 2959, 2932, 1796, 1766, 1733, 1693, 1492, 1224, 1106, 1048, 834.

LRMS (ESI, m/z) $[M+H]^+$ 317.9.

Identification of **2e** using HRMS-ESI and HRMS-CI analyses was not achieved.

1*R*,2*S*,5*R*-2-Isopropyl-5-methylcyclohexyl (Amino(4-fluorophenyl)(oxo)-λ^6-sulfaneylidene)carbamate (2f):

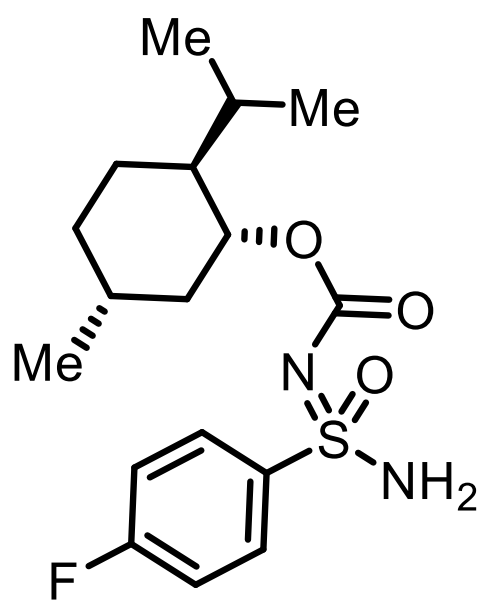

Prepared according to **General Procedure B** using *N*-alkoxysulfurdiimide **1f** (47.5 mg, 0.150 mmol, 1.00 equiv.), THF (1.50 mL) and 4-fluorophenylmagnesium bromide solution (0.17 mL, 0.165 mmol, 1.0 M in THF, 1.1 equiv.). Flash column chromatography (0% – 30% EtOAc in pentane) afforded sulfonimidamide **2f** as a colourless solid in a 1:1 mixture of inseparable diastereomers (42.7 mg, 80%).

R_f = 0.23 (30% EtOAc in pentane).

^{1}H NMR (400 MHz, $CDCl_3$) δ_H 8.02 (ddd, *J* = 9.0, 4.9, 2.3 Hz, 2H), 7.20 (t, *J* = 8.5 Hz, 2H), 6.07 (br. s, 2H), 4.52 – 4.37 (m, 1H), 1.99 – 1.85 (m, 2H), 1.69 – 1.52 (m, 2H), 1.51 – 1.36 (m, 1H), 1.36 – 1.17 (m, 1H), 1.04 – 0.91 (m, 2H), 0.91 – 0.83 (m, 6H), 0.83 – 0.74 (m, 2H), 0.74 – 0.62 (m, 3H).

^{13}C NMR (101 MHz, $CDCl_3$) δ_C 166.9, 164.3, 164.3, 158.0, 157.8, 137.0, 137.0, 136.9, 136.9, 130.4, 130.3, 130.3, 130.2, 116.6, 116.6, 116.4, 116.3, 76.5, 76.4, 47.2, 47.1, 41.1, 41.0, 34.3, 34.3, 31.5, 31.5,

26.1, 26.0, 23.4, 23.3, 22.1, 20.9, 20.9, 16.3, 16.2. Note: extra peaks from diastereomers and coupling to fluorine.

^{19}F NMR (377 MHz, $CDCl_3$) δ_F -104.2 (tt, *J* = 13.2, 5.0 Hz).

HRMS (ESI$^+$, m/z) calculated for $[C_{17}H_{25}FN_2O_3SNa]^+$ $[M+Na]^+$ 379.1462, found 379.1466.

IR (thin film, ν_{max}/cm^{-1}) = 3221, 2960, 2929, 2871, 1661, 1591, 1495, 1266.

Melting Point = 78– 80 °C (*n*-pentane).

$[\alpha]_D^{20}$ = – 38.6 (c = 1.0, $CHCl_3$).

***N*-(Amino(4-fluorophenyl)(oxo)-λ^6-sulfaneylidene)benzamide (2g):**

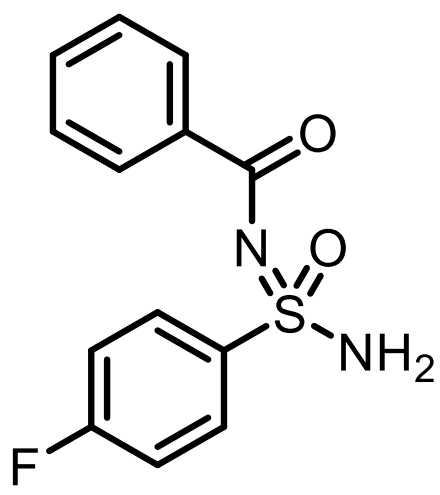

Prepared according to **General Procedure B** using *N*-alkoxysulfurdiimide **1g** (35.7 mg, 0.150 mmol, 1.00 equiv.), THF (1.50 mL) and 4-fluorophenylmagnesium bromide solution (0.17 mL, 0.165 mmol, 1.0 M in THF, 1.10 equiv.). Flash column chromatography (0% – 30% EtOAc in pentane) afforded sulfonimidamide **2g** as a colourless solid (28.0 mg, 67%).

R_f = 0.23 (30% EtOAc in pentane).

^{1}H NMR (400 MHz, $CDCl_3$) δ_H 8.12 (d, *J* = 7.7 Hz, 2H), 8.06 (dd, *J* = 8.8, 4.9 Hz, 2H), 7.52 (t, *J* = 7.4 Hz, 1H), 7.40 (t, *J* = 7.6 Hz, 2H), 7.19 (t, *J* = 8.4 Hz, 2H), 6.38 (br. s, 2H).

^{13}C NMR (101 MHz, $CDCl_3$) δ_C 173.7, 165.6 (d, *J* = 256.7 Hz), 137.4 (d, *J* = 3.2 Hz), 135.2, 132.7, 129.7 (d, *J* = 8.5 Hz), 129.6, 128.3, 116.6 (d, *J* = 23.0 Hz).

^{19}F NMR (377 MHz, $CDCl_3$) δ_F -103.8 (tt, *J* = 8.5, 5.0 Hz).

HRMS (ESI$^+$, m/z) calculated for $[C_{13}H_{11}FN_2O_2SNa]^+$ $[M+Na]^+$ 301.0417, found 301.0423.

IR (thin film, ν_{max}/cm^{-1}) = 3202, 3106, 3070, 2924, 1601, 1573, 1494, 1322, 1293, 1238, 1153, 837, 716.

Melting Point = 130 –134 °C ($CHCl_3$).

***N*-(Amino(4-fluorophenyl)(oxo)-λ^6-sulfaneylidene)acetamide (2h):**

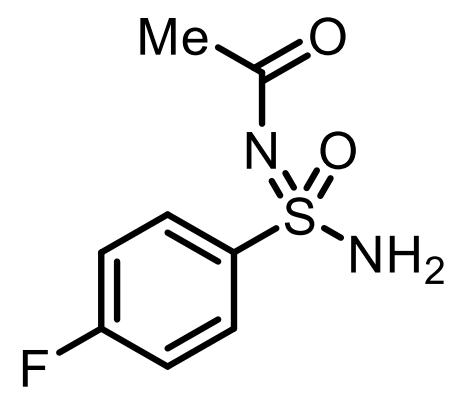

Prepared according to modified **General Procedure B** using *N*-alkoxysulfurdiimide **1h** (28.5 mg, 0.150 mmol, 1.00 equiv.), THF (1.50 mL) and 4-fluorophenylmagnesium bromide solution (0.33 mL, 0.33 mmol, 1.0 M in THF, 2.2 equiv.). Flash column chromatography (0% – 50% EtOAc in pentane) afforded sulfonimidamide **2h** as a colourless oil (9.7 mg, 30%).

R_f = 0.46 (50% EtOAc in pentane).

^{1}H NMR (400 MHz, $CDCl_3$) δ_H 8.04 – 7.95 (m, 2H), 7.24 – 7.13 (m, 2H), 6.31 (br. s, 2H), 2.11 (s, 3H).

^{13}C NMR (101 MHz, $CDCl_3$) δ_C 179.8, 165.6 (d, *J* = 256.7 Hz), 137.2 (d, *J* = 3.1 Hz), 129.7 (d, *J* = 9.5 Hz), 116.6 (d, *J* = 22.3 Hz), 27.2.

^{19}F NMR (377 MHz, $CDCl_3$) δ_F -103.8 (tt, *J* = 8.1, 4.2 Hz).

HRMS (ESI$^+$, m/z) calculated for $[C_8H_9FN_2O_2SNa]^+$ $[M+Na]^+$ 239.0261, found 239.0251.

IR (thin film, ν_{max}/cm^{-1}) = 3190, 3107, 3070, 1615, 1590, 1494, 1368, 1286, 1237, 1098, 1039, 839.

***N*'-(Diisopropylcarbamoyl)-4-fluorobenzenesulfonimidamide (2i):**

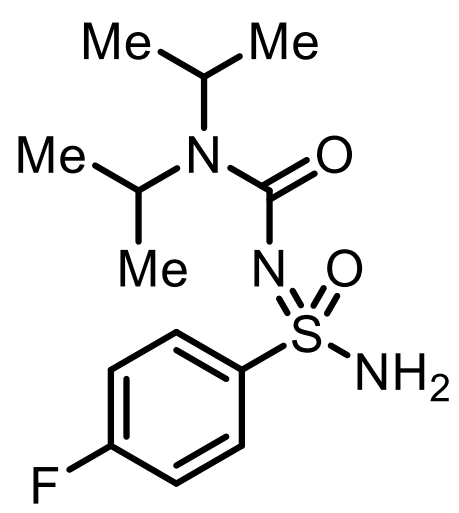

Prepared according to **General Procedure B** using *N*-alkoxysulfurdiimide **1i** (39.2 mg, 0.150 mmol, 1.00 equiv.), THF (1.50 mL) and 4-fluorophenylmagnesium bromide solution (0.17 mL, 0.165 mmol, 1.0 M in THF, 1.10 equiv.). Flash column chromatography (0% – 20% EtOAc in pentane) afforded sulfonimidamide **2i** as a colourless oil (32.4 mg, 72%).

R_f = 0.43 (30% EtOAc in pentane).

^{1}H NMR (400 MHz, $CDCl_3$) δ_H 8.09 – 7.86 (m, 2H), 7.16 (t, *J* = 8.63 Hz, 2H), 6.49 (br. s, 2H), 4.27 (s, 1H), 3.80 (s, 1H), 1.31 – 1.22 (m, 6H), 1.22 – 1.12 (m, 6H).

^{13}C NMR (101 MHz, $CDCl_3$) δ_C 165.1 (d, *J* = 254.3 Hz), 158.3, 139.3 (d, *J* = 3.3 Hz), 129.2 (d, *J* = 8.7 Hz), 116.3 (d, *J* = 22.3 Hz), 47.3, 45.3, 21.3, 21.2, 20.9. (Note: additional peaks due to rotamers caused by the diisopropyl urea group).

^{19}F NMR (377 MHz, $CDCl_3$) δ_F -105.7 (tt, *J* = 13.2, 5.0 Hz).

HRMS (ESI^+, m/z) calculated for $[C_{13}H_{20}FN_3O_2SNa]^+$ $[M+Na]^+$ 324.1152, found 324.1152.

IR (thin film, ν_{max}/cm^{-1}) = 3212, 2972, 2933, 1591, 1494, 1434, 1331, 1235, 1154, 1096, 837.

***N*-*tert*-Butyl-1,1-bis(4-fluorophenyl)-λ^6-sulfanediimine (2j):**

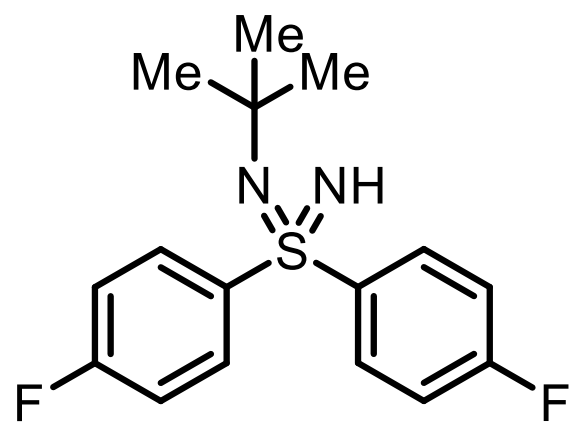

Prepared according to **General Procedure B** using *N*-alkoxysulfurdiimide **1j** (28.5 mg, 0.150 mmol, 1.00 equiv.), THF (1.50 mL) and 4-fluorophenylmagnesium bromide solution (0.17 mL, 0.165 mmol, 1.0 M in THF, 1.10 equiv.). Flash column chromatography (0% – 10% EtOAc in pentane) afforded sulfondiimine **2j** as a colourless solid (17.8 mg, 38%).

R_f = 0.28 (10% EtOAc in pentane).

^{1}H NMR (400 MHz, $CDCl_3$) δ_H 8.19 – 7.98 (m, 4H), 7.06 (m, 4H), 2.01 (br. s, 1H), 1.27 (s, 9H).

^{13}C NMR (101 MHz, $CDCl_3$) δ_C 164.3 (d, *J* = 251.9 Hz), 143.0 (d, *J* = 3.1 Hz), 130.2 (d, *J* = 9.5 Hz), 115.6 (d, *J* = 22.3 Hz), 55.0, 33.3.

^{19}F NMR (377 MHz, $CDCl_3$) δ_F -108.9 (tt, *J* = 8.2, 4.0 Hz).

HRMS (ESI^+, m/z) calculated for $[C_{16}H_{19}F_2N_2S]^+$ $[M+H]^+$ 309.1232, found 309.1247.

IR (thin film, ν_{max}/cm^{-1}) = 2970, 1590, 1489, 1401, 1360, 1219, 1175, 1152, 1083, 982, 834.

Melting Point = 110 –112 °C ($CHCl_3$).

4.4 Synthesis of Primary Sulfonimidamides (3)

Benzyl ([1,1'-biphenyl]-4-yl(amino)(oxo)-λ[6]-sulfaneylidene)carbamate (3a):

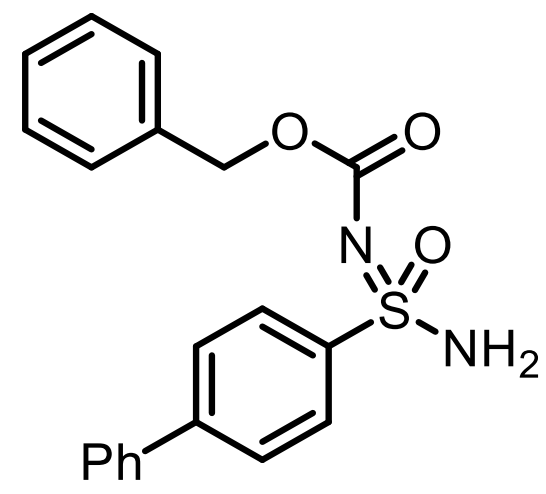

Magnesium turnings (360 mg, 15.0 mmol) were added to an oven-dried round-bottom flask. The vessel was then sealed with a rubber septum, evacuated and backfilled with N_2 (×3). Dry THF (10 mL) was added to the reaction mixture, along with an I_2 crystal. 4-Bromo-1,1'-biphenyl (2.33 g, 10.0 mmol) in THF (1 mL) was added dropwise to the mixture before stirring at rt for 3 h.

Prepared according to **General Procedure B** using *N*-alkoxysulfurdiimide **1c** (40.3 mg, 0.150 mmol, 1.00 equiv.), THF (1.50 mL) and 4-biphenylmagnesium bromide solution (0.20 mL, 0.165 mmol, 0.83 M in THF, 1.10 equiv.). Flash column chromatography (20% – 50% EtOAc in pentane) afforded sulfonimidamide **3a** as a cream solid (47.6 mg, 87%).

A scale up of this reaction was performed according to **General Procedure B** using *N*-alkoxysulfurdiimide **1c** (1.34 g, 5.00 mmol, 1.00 equiv.), THF (50 mL) and 4-biphenylmagnesium bromide solution (6.63 mL, 5.50 mmol, 0.83 M in THF, 1.10 equiv.). Flash column chromatography (0% – 100% EtOAc in pentane) afforded sulfonimidamide **3a** as a cream solid (1.34 g, 73%).

$\mathbf{R_f}$ = 0.33 (40% EtOAc in pentane).

^{1}H NMR (400 MHz, $(CD_3)_2SO$) δ_H 7.95 (d, *J* = 8.3 Hz, 2H), 7.88 (d, *J* = 8.3 Hz, 2H), 7.85 (br. s, 2H), 7.74 (d, *J* = 7.4 Hz, 2H), 7.52 (t, *J* = 7.5 Hz, 2H), 7.45 (t, *J* = 7.4 Hz, 1H), 7.37 – 7.24 (m, 3H), 7.24 – 7.18 (m, 2H), 4.95 (d, *J* = 12.7 Hz, 1H), 4.88 (d, *J* = 12.7 Hz, 1H).

^{13}C NMR (101 MHz, $(CD_3)_2SO$) δ_C 157.1, 144.0, 141.2, 138.6, 137.0, 129.2, 128.6, 128.3, 127.7, 127.6, 127.5, 127.2, 127.1, 66.1.

HRMS (ESI$^+$, m/z) calculated for $[C_{20}H_{19}N_2O_3S]^+$ $[M+H]^+$ 367.1111, found 367.1118.

IR (thin film, ν_{max}/cm^{-1}) = 3235, 3061, 2361, 1673, 1382, 1279, 1100, 975, 763, 698.

Melting Point = 145 –149 °C (EtOAc).

Benzyl (amino(oxo)(*p*-tolyl)-λ⁶-sulfaneylidene)carbamate (3b):

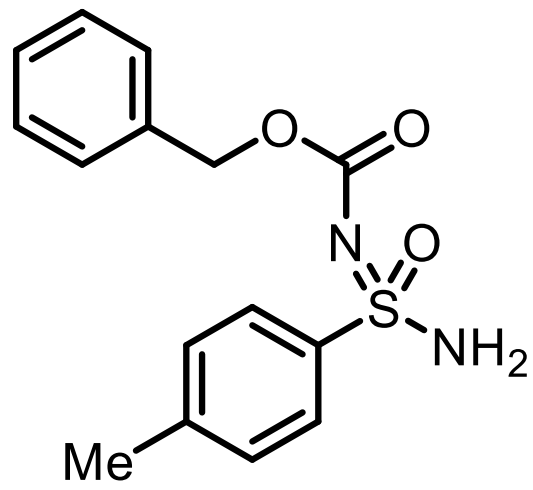

Prepared according to **General Procedure B** using *N*-alkoxysulfurdiimide **1c** (40.3 mg, 0.150 mmol, 1.00 equiv.), THF (1.50 mL) and *p*-tolylmagnesium bromide solution (0.16 mL, 0.165 mmol, 1.05 M in THF, 1.10 equiv.). Flash column chromatography (20% – 50% EtOAc in pentane) afforded sulfonimidamide **3b** as a colourless solid (33.0 mg, 72%).

$\mathbf{R_f}$ = 0.42 (40% EtOAc in pentane).

^{1}H NMR (400 MHz, $(CD_3)_2SO$) δ_H 7.74 (d, *J* = 8.2 Hz, 2H), 7.71 (br. s, 2H), 7.38 (d, *J* = 8.1 Hz, 2H), 7.35 – 7.24 (m, 3H), 7.22 – 7.17 (m, 2H), 4.91 (d, *J* = 12.8 Hz, 1H), 4.86 (d, *J* = 12.7 Hz, 1H), 2.38 (s, 3H).

^{13}C NMR (101 MHz, $(CD_3)_2SO$) δ_C 157.1, 142.8, 139.5, 137.1, 129.4, 128.3, 127.6, 127.4, 126.9, 66.0, 21.0.

HRMS (ESI^+, m/z) calculated for $[C_{15}H_{17}N_2O_3S]^+$ $[M+H]^+$ 305.0954, found 305.0963.

IR (thin film, ν_{max}/cm^{-1}) = 3235, 3063, 1672, 1381, 1277, 1098, 976, 758.

Melting Point = 140 – 143 °C (EtOAc).

Benzyl (amino(oxo)(*m*-tolyl)-λ⁶-sulfaneylidene)carbamate (3c):

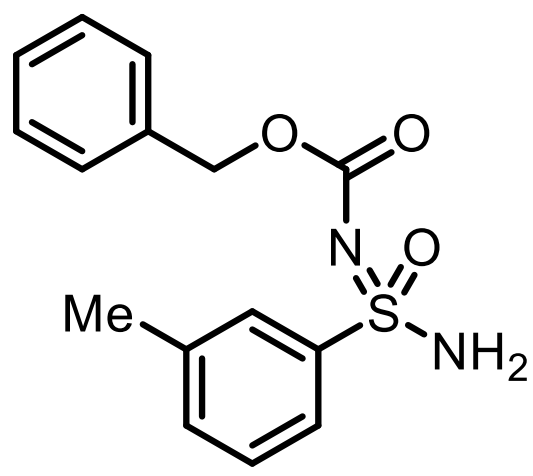

Prepared according to **General Procedure B** using *N*-alkoxysulfurdiimide **1c** (40.3 mg, 0.150 mmol, 1.00 equiv.), THF (1.50 mL) and *m*-tolylmagnesium chloride solution (0.17 mL, 0.165 mmol, 0.97 M in THF, 1.10 equiv.). Flash column chromatography (20% – 50% EtOAc in pentane) afforded sulfonimidamide **3c** as a colourless solid (30.0 mg, 66%).

R_f = 0.30 (40% EtOAc in pentane).

^{1}H NMR (400 MHz, $(CD_3)_2SO$) δ_H 7.74 (br. s, 2H), 7.70 – 7.64 (m, 2H), 7.51 – 7.41 (m, 2H), 7.37 – 7.24 (m, 3H), 7.23 – 7.18 (m, 2H), 4.92 (d, *J* = 12.8 Hz, 1H), 4.88 (d, *J* = 12.8 Hz, 1H), 2.37 (s, 3H).

^{13}C NMR (101 MHz, $(CD_3)_2SO$) δ_C 157.0, 142.4, 138.5, 137.0, 133.0, 128.8, 128.3, 127.6, 127.4, 126.9, 123.9, 66.0, 20.9.

HRMS (ESI$^+$, m/z) calculated for $[C_{15}H_{17}N_2O_3S]^+$ $[M+H]^+$ 305.0954, found 305.0955.

IR (thin film, ν_{max}/cm^{-1}) = 3220, 3064, 2359, 1665, 1382, 1276, 975, 758.

Melting Point: 115 – 117 °C (EtOAc).

Benzyl (amino(oxo)(*o*-tolyl)-λ^6-sulfaneylidene)carbamate (3d):

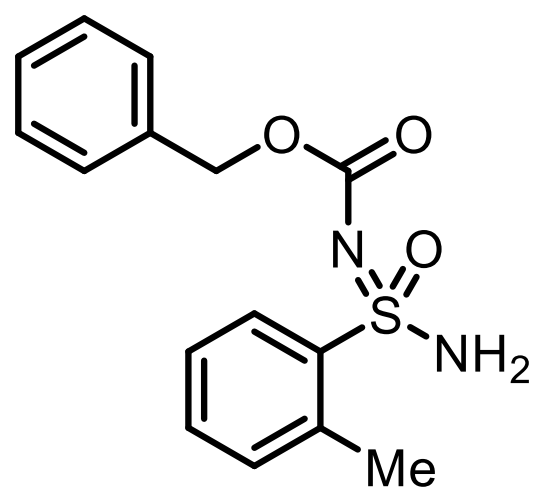

Prepared according to **General Procedure B** using *N*-alkoxysulfurdiimide **1c** (40.3 mg, 0.150 mmol, 1.00 equiv.), THF (1.50 mL) and *o*-tolylmagnesium chloride solution (0.17 mL, 0.165 mmol, 0.95 M in THF, 1.10 equiv.). Flash column chromatography (10% – 50% EtOAc in pentane) afforded sulfonimidamide **3d** as a colourless solid (21.3 mg, 47%).

R_f = 0.38 (40% EtOAc in pentane).

^{1}H NMR (400 MHz, $(CD_3)_2SO$) δ_H 7.93 (dd, *J* = 8.0, 1.4 Hz, 1H), 7.82 (br. s, 2H), 7.52 (td, *J* = 7.5, 1.4 Hz, 1H), 7.42 – 7.36 (m, 2H), 7.34 – 7.24 (m, 3H), 7.21 – 7.15 (m, 2H), 4.92 (d, *J* = 12.9 Hz, 1H), 4.88 (d, *J* = 12.9 Hz, 1H), 2.57 (s, 3H).

^{13}C NMR (101 MHz, $(CD_3)_2SO$) δ_C 157.0, 140.0, 137.1, 136.6, 132.5, 132.5, 129.0, 128.3, 127.6, 127.3, 126.1, 66.0, 19.8.

HRMS (ESI$^+$, m/z) calculated for $[C_{15}H_{17}N_2O_3S]^+$ $[M+H]^+$ 305.0954, found 305.0954.

IR (thin film, ν_{max}/cm^{-1}) = 3237, 2981, 2360, 1667, 1456, 1382, 1275, 760.

Melting Point = 152– 154 °C (EtOAc).

Benzyl (Amino(4-methoxyphenyl)(oxo)-λ⁶-sulfaneylidene)carbamate (3e):

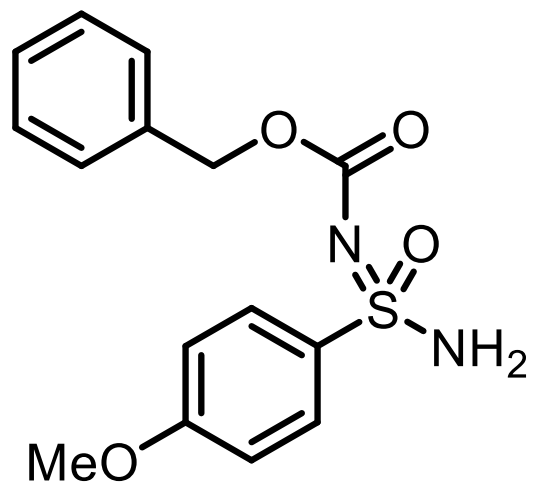

Prepared according to **General Procedure B** using *N*-alkoxysulfurdiimide **1c** (40.3 mg, 0.150 mmol, 1.00 equiv.), THF (1.50 mL) and 4-methoxyphenylmagnesium bromide solution (0.34 mL, 0.165 mmol, 0.48 M in THF, 1.10 equiv.). Flash column chromatography (30% – 60% EtOAc in pentane) afforded sulfonimidamide **3e** as a pink solid (39.6 mg, 82%).

R_f = 0.30 (50% EtOAc in pentane).

^{1}H NMR (400 MHz, $CDCl_3$) δ_H 7.88 (d, *J* = 8.7 Hz, 2H), 7.36 – 7.19 (m, 5H), 6.90 (d, *J* = 8.7 Hz, 2H), 6.05 (br. s, 2H), 5.03 (d, *J* = 12.3 Hz, 1H), 4.96 (d, *J* = 12.4 Hz, 1H), 3.83 (s, 3H).

^{13}C NMR (101 MHz, $CDCl_3$) δ_C 163.6, 158.2, 136.3, 131.5, 129.7, 128.5, 128.1, 128.0, 114.4, 67.8, 55.8.

HRMS (ESI$^+$, m/z) calculated for $[C_{15}H_{17}N_2O_4S]^+$ $[M+H]^+$ 321.0904, found 321.0903.

IR (thin film, ν_{max}/cm^{-1}) = 3235, 1666, 1596, 1498, 1258, 1099, 834, 737.

Melting Point = 94 –95 °C ($CHCl_3$).

Benzyl (amino(4-chlorophenyl)(oxo)-λ⁶-sulfaneylidene)carbamate (3f):

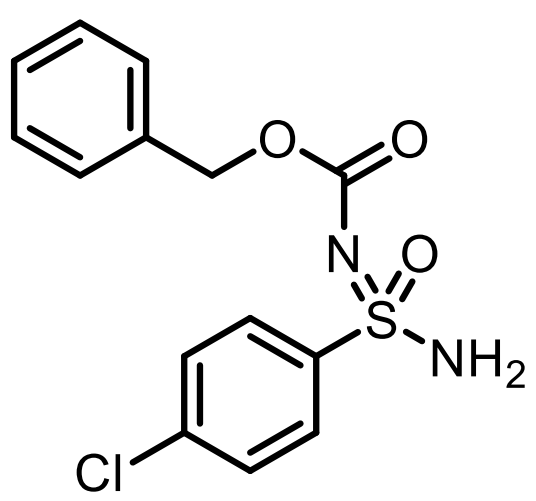

Prepared according to **General Procedure B** using *N*-alkoxysulfurdiimide **1c** (40.3 mg, 0.150 mmol, 1.00 equiv.), THF (1.50 mL) and 4-chlorophenylmagnesium bromide solution (0.16 mL, 0.165 mmol, 1.04 M in Et_2O, 1.10 equiv.). Flash column chromatography (20% – 50% EtOAc in pentane) afforded sulfonimidamide **3f** as a pale-pink solid (29.7 mg, 61%).

R_f = 0.40 (40% EtOAc in pentane).

^{1}H NMR (400 MHz, $(CD_3)_2SO$) δ_H 7.89 (br. s, 2H), 7.88 – 7.83 (m, 2H), 7.70 – 7.63 (m, 2H), 7.35 – 7.27 (m, 3H), 7.22 – 7.17 (m, 2H), 4.93 (d, *J* = 12.7 Hz, 1H), 4.86 (d, *J* = 12.7 Hz, 1H).

^{13}C NMR (101 MHz $(CD_3)_2SO$) δ_C 156.9, 141.4, 137.3, 136.9, 129.1, 128.8, 128.3, 127.7, 127.5, 66.1.

HRMS (ESI$^+$, m/z) calculated for $[C_{14}H_{14}{}^{35}ClN_2O_3S]^+$ $[M+H]^+$ 325.0408, found 325.0408; $[C_{14}H_{13}{}^{37}ClN_2O_3SNa]^+$ $[M+Na]^+$ 349.0198, found 349.0206.

IR (thin film, ν_{max}/cm^{-1}) = 3283, 3164, 1712, 1273, 1086, 992, 873.

Melting Point = 134 –137 °C (EtOAc).

Benzyl (amino(4-aminophenyl)(oxo)-λ^6-sulfaneylidene)carbamate (3g):

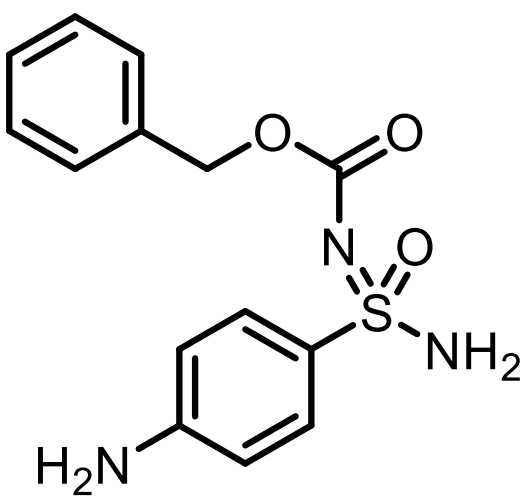

Prepared according to modified **General Procedure B** using *N*-alkoxysulfurdiimide **1c** (40.3 mg, 0.150 mmol, 1.00 equiv.), THF (1.50 mL) and 4-[bis(trimethylsilyl)amino]phenylmagnesium bromide solution (0.37 mL, 0.165 mmol, 0.45 M in THF, 1.10 equiv.). The reaction mixture was diluted with EtOAc and quenched with aq. 1 M HCl. The aqueous layer was extracted with EtOAc and the combined organic extracts were dried over $MgSO_4$, filtered and concentrated *in vacuo*. Flash column chromatography (20% – 60% EtOAc in pentane) afforded sulfonimidamide **3g** as a beige solid (30.0 mg, 65%).

R_f = 0.19 (60% EtOAc in pentane).

^{1}H NMR (400 MHz, $(CD_3)_2SO$) δ_H 7.49 (d, *J* = 8.4 Hz, 2H), 7.38 (br. s, 2H), 7.35 – 7.26 (m, 3H), 7.22 (d, *J* = 7.3 Hz, 2H), 6.60 (d, *J* = 8.4 Hz, 2H), 5.98 (br. s, 2H), 4.91 (d, *J* = 12.9 Hz, 1H), 4.86 (d, *J* = 12.8 Hz, 1H).

^{13}C NMR (101 MHz $(CD_3)_2SO$) δ_C 157.2, 152.8, 137.3, 128.9, 128.3, 127.6, 127.4, 126.5, 112.5, 65.8.

HRMS (ESI$^+$, m/z) calculated for $[C_{14}H_{15}N_3O_3SNa]^+$ $[M+Na]^+$ 328.0726, found 328.0729.

IR (thin film, ν_{max}/cm^{-1}) = 3471, 3368, 3233, 3062, 2926, 1657, 1631, 1266, 1095.

Melting Point = 107 – 110 °C (EtOAc).

Benzyl (amino(4-cyanophenyl)(oxo)-λ^6-sulfaneylidene)carbamate (3h):

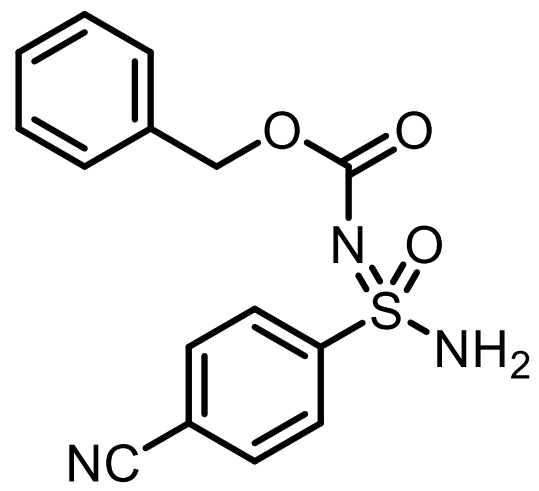

4-Iodobenzonitrile (37.8 mg, 0.165 mmol, 1.10 equiv.) was weighed into an oven-dried reaction vessel. The vessel was then sealed with a rubber septum, evacuated and backfilled with N_2 (×3). Dry THF (0.34 mL) was added and the reaction mixture cooled to -20 °C. Isopropylmagnesium chloride solution (0.08 mL, 2.08 M, 0.165 mmol, 1.10 equiv.) was added dropwise and the reaction mixture stirred at -20 °C for 30 mins, until the magnesium-halogen exchange was complete.

N-Alkoxysulfurdiimide **1c** (40.3 mg, 0.150 mmol, 1.00 equiv.) was weighed into an oven-dried reaction vessel. The vessel was then sealed with a rubber septum, evacuated and backfilled with N_2 (×3). Dry THF (1.50 mL) was added and the solution was added dropwise to the organometallic-containing vessel at -20 °C. The reaction mixture was warmed to rt in a water bath and stirred for 30 mins. The reaction was quenched with silica and the crude product was purified directly by flash column chromatography (10% – 40% EtOAc in pentane) to afford sulfonimidamide **3h** as a colourless solid (13.0 mg, 27%).

R_f = 0.41 (60% EtOAc in pentane).

^{1}H NMR (400 MHz, $(CD_3)_2SO$) δ_H 8.06 (d, *J* = 7.9 Hz, 4H), 7.99 (d, *J* = 8.2 Hz, 2H), 7.30 (d, *J* = 7.2 Hz, 3H), 7.22 – 7.15 (m, 2H), 4.92 (d, *J* = 12.7 Hz, 1H), 4.85 (d, *J* = 12.6 Hz, 1H).

^{13}C NMR (101 MHz $(CD_3)_2SO$) δ_C 156.9, 147.0, 136.8, 133.2, 128.3, 127.8, 127.6, 127.6, 117.8, 114.8, 66.3.

HRMS (ESI$^+$, m/z) calculated for $[C_{15}H_{14}N_3O_3S]^+$ $[M+H]^+$ 316.0750, found 316.0738.

IR (thin film, ν_{max}/cm^{-1}) = 3263, 3173, 2924, 2853, 2234, 1640, 1394, 1307, 1267, 1119, 953, 696.

Melting Point = 126 – 128 °C (EtOAc).

Benzyl (amino(oxo)(4-(piperidine-1-carbonyl)phenyl)-λ⁶-sulfaneylidene)carbamate (3i):

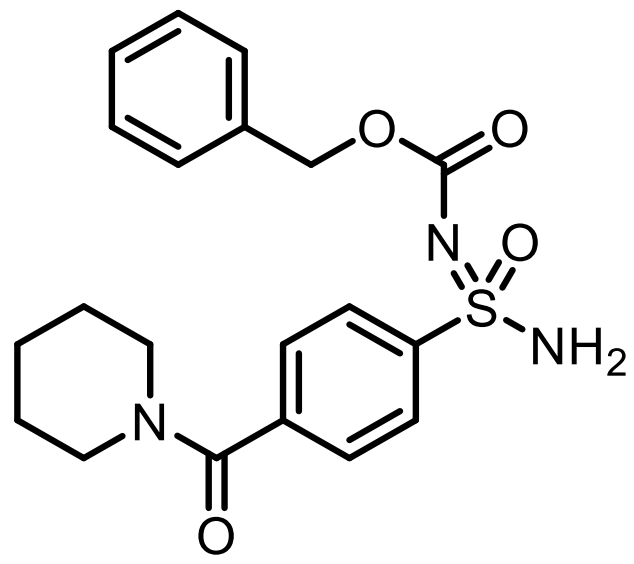

(4-iodophenyl)(piperidin-1-yl)methanone (52.0 mg, 0.165 mmol, 1.10 equiv.) was weighed into an oven-dried reaction vessel. The vessel was then sealed with a rubber septum, evacuated and backfilled with N_2 (×3). Dry THF (0.17 mL) was added and the reaction mixture cooled to -20 °C. Isopropylmagnesium bromide solution (0.20 mL, 0.81 M, 0.165 mmol, 1.10 equiv.) was added dropwise and the reaction mixture stirred at -20 °C for 1 h, until the magnesium-halogen exchange was complete.

N-Alkoxysulfurdiimide **1c** (40.3 mg, 0.150 mmol, 1.00 equiv.) was weighed into an oven-dried reaction vessel. The vessel was then sealed with a rubber septum, evacuated and backfilled with N_2 (×3). Dry THF (1.50 mL) was added and the solution was added dropwise to the organometallic-containing vessel at -20 °C. The reaction mixture was warmed to rt in a water bath and stirred for 30 mins. The reaction was quenched with silica and the crude product was purified directly by flash column chromatography (40% – 70% EtOAc in pentane) to afford sulfonimidamide **3i** as a colourless solid (33.9 mg, 56%).

R_f = 0.10 (60% EtOAc in pentane).

^{1}H NMR (400 MHz, $CDCl_3$) δ_H 7.93 (d, *J* = 8.1 Hz, 2H), 7.41 (d, *J* = 8.0 Hz, 2H), 7.33 – 7.27 (m, 5H), 6.31 (br. s, 2H), 5.05 (d, *J* = 1.3 Hz, 2H), 3.69 (s, 2H), 3.23 (t, *J* = 5.5 Hz, 2H), 1.68 (s, 4H), 1.49 (s, 2H).

^{13}C NMR (101 MHz, $CDCl_3$) δ_C 168.5, 158.1, 141.7, 141.1, 136.2, 128.6, 128.2, 128.2, 127.5, 127.5, 68.1, 48.8, 43.4, 26.6, 25.6, 24.5. (note: for piperidine ring, 5 peaks were found instead of 3).

HRMS (ESI$^+$, m/z) calculated for $[C_{20}H_{24}N_3O_4S]^+$ $[M+H]^+$ 402.1482, found 402.1496.

IR (thin film, ν_{max}/cm^{-1}) = 3213, 3063, 2941, 1674, 1613, 1379, 1263.

Melting Point = 50 – 53 °C ($CHCl_3$).

Benzyl (amino(methyl)(oxo)-λ^6-sulfaneylidene)carbamate (3j):

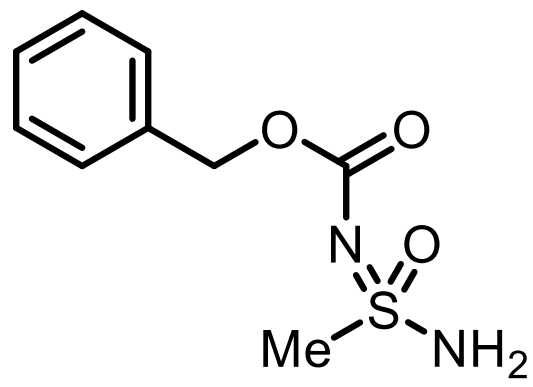

Prepared according to **General Procedure B** using *N*-alkoxysulfurdiimide **1c** (40.3 mg, 0.150 mmol, 1.00 equiv.), THF (1.50 mL) and methylmagnesium chloride solution (0.06 mL, 0.165 mmol, 2.78 M in THF, 1.10 equiv.). Flash column chromatography (30% – 80% EtOAc in pentane) afforded sulfonimidamide **3j** as a colourless oil (26.6 mg, 78%).

R$_f$ = 0.13 (50% EtOAc in pentane).

^{1}H NMR (400 MHz, $CDCl_3$) δ_H 7.40 – 7.25 (m, 5H), 5.88 (br. s, 2H), 5.10 (s, 2H), 3.25 (s, 3H).

^{13}C NMR (101 MHz, $CDCl_3$) δ_C 158.8, 136.1, 128.7, 128.3, 128.2, 68.0, 43.8.

HRMS (ESI$^+$, m/z) calculated for $[C_9H_{12}N_2O_3S]^+$ $[M+H]^+$ 229.0641, found 229.0633.

IR (thin film, ν_{max}/cm^{-1}) = 3234, 3035, 1667, 1383, 1286, 1000, 911, 738.

Benzyl (amino(oxo)((trimethylsilyl)methyl)-λ^6-sulfaneylidene)carbamate (3k):

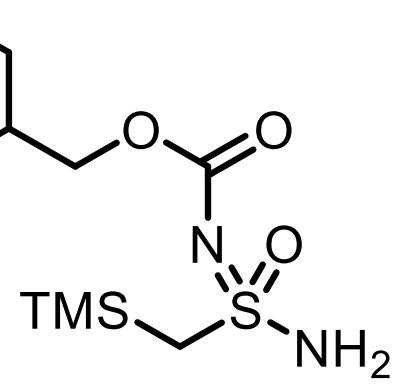

Prepared according to **General Procedure B** using *N*-alkoxysulfurdiimide **1c** (40.3 mg, 0.150 mmol, 1.00 equiv.), THF (1.50 mL) and (trimethylsilyl)methylmagnesium chloride solution (0.16 mL, 0.165 mmol, 1.03 M in Et_2O, 1.10 equiv.). Flash column chromatography (10% – 40% EtOAc in pentane) afforded sulfonimidamide **3k** as a colourless solid (33.3 mg, 74%).

R$_f$ = 0.47 (40% EtOAc in pentane).

^{1}H NMR (400 MHz, $CDCl_3$) δ_H 7.41 – 7.27 (m, 5H), 5.49 (br. s, 2H), 5.15 – 5.03 (m, 2H), 3.23 (d, *J* = 14.3 Hz, 1H), 3.02 (d, *J* = 14.4 Hz, 1H), 0.23 (s, 9H).

^{13}C NMR (101 MHz, $CDCl_3$) δ_C 159.6, 136.5, 128.6, 128.1, 128.1, 67.9, 46.6, -1.0.

HRMS (ESI$^+$, m/z) calculated for $[C_{12}H_{21}N_2O_3SSi]^+$ $[M+H]^+$ 301.1037, found 301.1028.

IR (thin film, ν_{max}/cm^{-1}) = 3240, 2958, 1656, 1382, 1284, 914, 856.

Melting Point = 60 – 62 °C ($CHCl_3$).

Benzyl (amino(oxo)(phenethyl)-λ^6-sulfaneylidene)carbamate (3l):

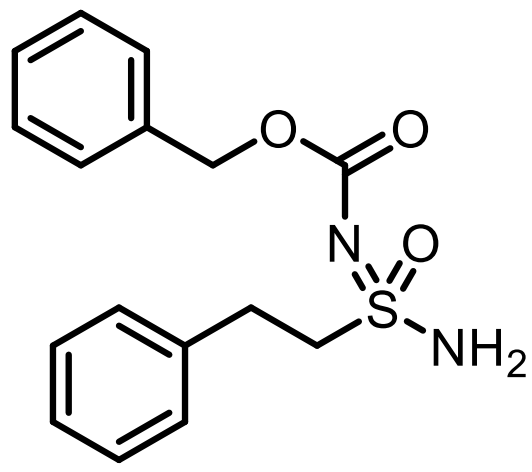

Prepared according to **General Procedure B** using *N*-alkoxysulfurdiimide **1c** (40.3 mg, 0.150 mmol, 1.00 equiv.), THF (1.50 mL) and phenylethylmagnesium bromide solution (0.19 mL, 0.165 mmol, 0.87 M in THF, 1.10 equiv.). Flash column chromatography (20% – 40% EtOAc in pentane) afforded sulfonimidamide **3l** as a colourless solid (31.9 mg, 67%).

R_f = 0.38 (40% EtOAc in pentane).

^{1}H NMR (400 MHz, $CDCl_3$) δ_H 7.40 – 7.21 (m, 8H), 7.20 – 7.14 (m, 2H), 5.54 (s, 2H), 5.10 (br. s, 2H), 3.62 (ddd, *J* = 10.4, 6.1, 2.3 Hz, 2H), 3.21 – 3.08 (m, 2H).

^{13}C NMR (101 MHz, $CDCl_3$) δ_C 158.8, 137.1, 136.2, 129.1, 128.7, 128.6, 128.3, 128.2, 127.3, 68.1, 56.8, 29.6.

HRMS (ESI$^+$, m/z) calculated for $[C_{16}H_{19}N_2O_3S]^+$ $[M+H]^+$ 319.1111, found 319.1096.

IR (thin film, ν_{max}/cm^{-1}) = 3234, 3064, 3032, 1658, 1380, 1266, 985, 644.

Melting Point = 60 – 62 °C ($CHCl_3$).

Benzyl ((2-(1,3-dioxolan-2-yl)ethyl)(amino)(oxo)-λ^6-sulfaneylidene)carbamate (3m):

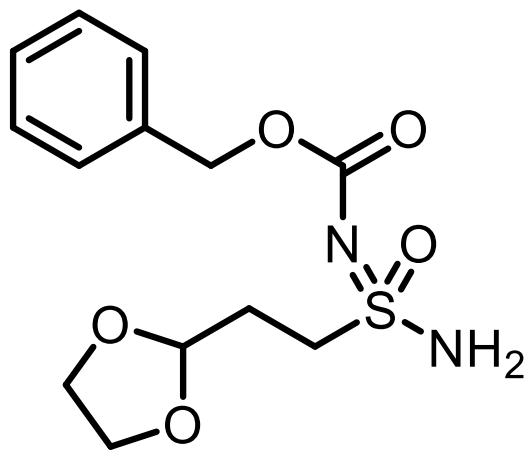

Magnesium turnings (292 mg, 12.0 mmol, 1.2 equiv.) were added to an oven-dried round-bottom flask. The vessel was then sealed with a rubber septum, evacuated and backfilled with N_2 (×3). Dry THF (10 mL) was added to the reaction mixture, along with an I_2 crystal. 2-(2-Bromoethyl)-1,3-dioxolane (1.17 mL, 10.0 mmol) in THF (1 mL) was added dropwise to the mixture before stirring at rt for 2 h.

Prepared according to **General Procedure B** using *N*-alkoxysulfurdiimide **1c** (40.3 mg, 0.150 mmol, 1.00 equiv.), THF (1.50 mL) and (1,3-dioxan-2-ylethyl)magnesium bromide solution (0.29 mL, 0.165 mmol, 0.57 M in THF, 1.10 equiv.). Flash column chromatography (40% – 60% EtOAc in pentane) afforded sulfonimidamide **3m** as a colourless oil (23.0 mg, 49%).

R_f = 0.30 (40% EtOAc in pentane).

^{1}H NMR (400 MHz, $CDCl_3$) δ_H 7.41 – 7.28 (m, 5H), 5.75 (s, 2H), 5.10 (br.s, 2H), 4.99 (t, *J* = 3.9 Hz, 1H), 3.99 – 3.89 (m, 2H), 3.89 – 3.79 (m, 2H) 3.54 – 3.44 (m, 2H), 2.28 – 2.17 (m, 2H).

^{13}C NMR (101 MHz, $CDCl_3$) 158.8, 136.3, 128.6, 128.2, 128.2, 101.8, 68.0, 65.3, 50.3, 27.6.

HRMS (ESI$^+$, m/z) calculated for $[C_{13}H_{19}N_2O_5S]^+$ $[M+H]^+$ 315.1009, found 315.1000.

IR (thin film, ν_{max}/cm^{-1}) = 3228, 3066, 2916, 1658, 1381, 1265, 1135, 894, 699.

Benzyl (amino(cyclohexyl)(oxo)-λ^6-sulfaneylidene)carbamate (3n):

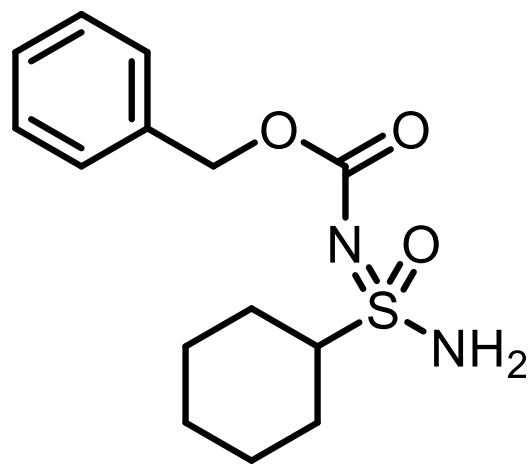

Prepared according to **General Procedure B** using *N*-alkoxysulfurdiimide **1c** (40.3 mg, 0.150 mmol, 1.00 equiv.), THF (1.50 mL) and cyclohexylmagnesium chloride solution (0.08 mL, 0.165 mmol, 2.14 M in Et_2O, 1.10 equiv.). Flash column chromatography (20% – 60% EtOAc in pentane) afforded sulfonimidamide **3n** as an off-white solid (37.1 mg, 83%).

R_f = 0.39 (40% EtOAc in pentane).

^{1}H NMR (400 MHz, $CDCl_3$) δ_H 7.41 – 7.26 (m, 5H), 5.56 (br. s, 2H), 5.10 (s, 2H), 3.20 (m, 1H), 2.20 (m, 2H), 1.95 – 1.85 (m, 2H), 1.69 (m, 1H), 1.50 (m, 2H), 1.35 – 1.06 (m, 3H).

^{13}C NMR (101 MHz, $CDCl_3$) δ_C 159.0, 136.4, 128.6, 128.2, 128.2, 67.9, 63.7, 26.0, 25.6, 25.1, 25.0. (note: for cyclohexane ring, 6 peaks were found instead of 4).

HRMS (ESI$^+$, m/z) calculated for $[C_{14}H_{21}N_2O_3S]^+$ $[M+H]^+$ 297.1267, found 297.1268.

IR (thin film, ν_{max}/cm^{-1}) = 3250, 3067, 2937, 2858, 1659, 1454, 1381, 1286, 1261, 894, 760.

Melting Point = 89 – 93 °C ($CHCl_3$).

Benzyl (amino(1-benzylpiperidin-4-yl)(oxo)-λ^6-sulfaneylidene)carbamate (3o):

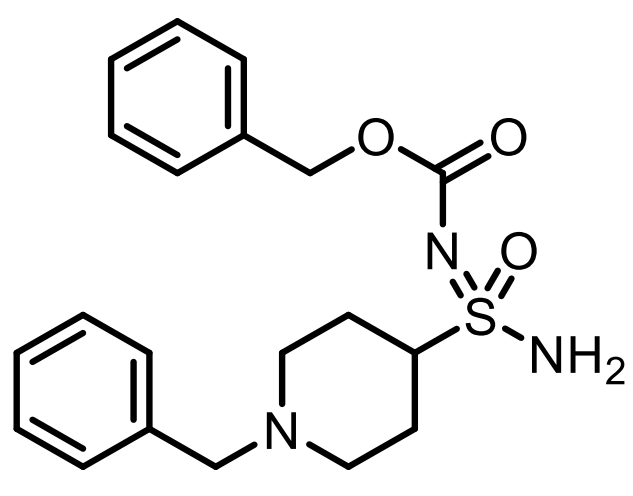

Magnesium turnings (146 mg, 6.00 mmol) were added to an oven-dried round-bottom flask. The vessel was then sealed with a rubber septum, evacuated and backfilled with N_2 (×3). Dry THF (5 mL) was added to the reaction mixture, along with an I_2 crystal. 1-Benzyl-4-bromopiperidine (1.27 g, 5 mmol) in THF (1 mL) was added dropwise to the mixture before heating to 60 °C for 2 h.

Prepared according to **General Procedure B** using *N*-alkoxysulfurdiimide **1c** (40.3 mg, 0.150 mmol, 1.00 equiv.), THF (1.50 mL) and (1-benzylpiperidin-4-yl)magnesium bromide solution (0.53 mL, 0.165 mmol, 0.31 M in THF, 1.10 equiv.). Flash column chromatography (0% – 80% EtOAc in pentane) afforded sulfonimidamide **3o** as a pink solid (20.0 mg, 34%).

R_f = 0.17 (80% EtOAc in pentane).

^{1}H NMR (400 MHz, $CDCl_3$) δ_H 7.46 – 7.17 (m, 10H), 5.10 (br. s, 2H), 3.51 (s, 2H), 3.21 (tt, *J* = 11.6, 3.7 Hz, 1H), 3.07 – 2.97 (m, 2H), 2.11 (t, *J* = 15.4 Hz, 2H), 1.99 (td, *J* = 12.0, 3.6 Hz, 2H), 1.90 (td, *J* = 11.4, 5.9 Hz, 2H).

^{13}C NMR (101 MHz, $CDCl_3$) δ_C 158.9, 137.9, 136.3, 129.1, 128.6, 128.5, 128.3, 128.2, 127.4, 68.1, 62.6, 62.0, 52.1, 25.9, 25.2. (note: for piperidine ring, 5 peaks were found instead of 3).

HRMS (ESI$^+$, m/z) calculated for $[C_{20}H_{26}N_3O_3S]^+$ $[M+H]^+$ 388.1689, found 388.1682.

IR (thin film, ν_{max}/cm^{-1}) = 3221, 3087, 3061, 1658, 1585, 1129, 679, 651.

Melting Point = 42 – 44 °C ($CHCl_3$).

Benzyl (amino(cyclopropyl)(oxo)-λ^6-sulfaneylidene)carbamate (3p):

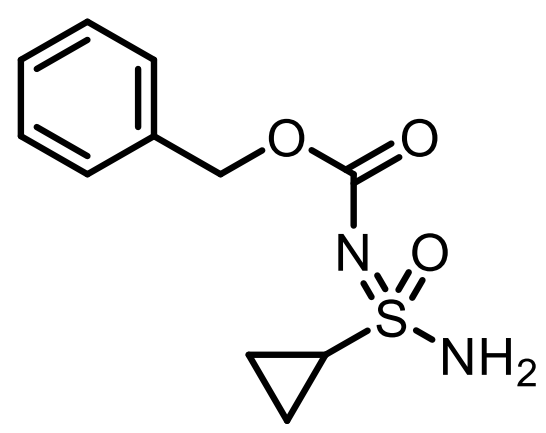

Prepared according to **General Procedure B** using *N*-alkoxysulfurdiimide **1c** (40.3 mg, 0.150 mmol, 1.00 equiv.), THF (1.50 mL) and cyclopropylmagnesium bromide solution (0.31 mL, 0.165 mmol, 0.54 M in 2-MeTHF, 1.10 equiv.). Flash column chromatography (30% – 50% EtOAc in pentane) afforded sulfonimidamide **3p** as a yellow solid (20.8 mg, 55%).

R_f = 0.10 (40% EtOAc in pentane).

^{1}H NMR (400 MHz, $CDCl_3$) δ_H 7.42 – 7.27 (m, 5H), 5.64 (br. s, 2H), 5.11 (s, 2H), 2.81 (m, 1H), 1.41 – 1.29 (m, 1H), 1.25 (m, 1H), 1.17 – 0.96 (m, 2H).

^{13}C NMR (101 MHz, $CDCl_3$) δ_C 158.9, 136.3, 128.6, 128.3, 128.2, 68.0, 33.5, 6.7, 5.7. (Note: for cyclopropyl ring, 3 peaks were found instead of 2).

HRMS (ESI^+, m/z) calculated for $[C_{11}H_{15}N_2O_3S]^+$ $[M+H]^+$ 255.0798, found 255.0798.

IR (thin film, ν_{max}/cm^{-1}) = 3219, 3000, 1661, 1382, 1278, 913, 892.

Melting Point = 47 – 48 °C ($CHCl_3$).

Benzyl (amino(benzyl)(oxo)-λ^6-sulfaneylidene)carbamate (3q):

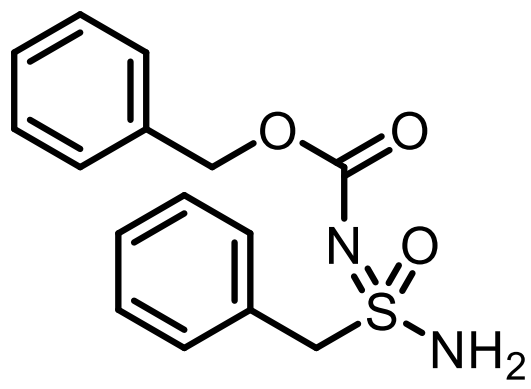

Prepared according to **General Procedure B** using *N*-alkoxysulfurdiimide **1c** (40.3 mg, 0.150 mmol, 1.00 equiv.), THF (1.50 mL) and benzylmagnesium chloride solution (0.19 mL, 0.165 mmol, 0.87 M in Et_2O, 1.10 equiv.). Flash column chromatography (20% – 30% EtOAc in pentane) afforded sulfonimidamide **3q** as a colourless solid (21.4 mg, 47%).

R_f = 0.27 (40% EtOAc in pentane).

^{1}H NMR (400 MHz, $CDCl_3$) δ_H 7.44 – 7.28 (m, 10H), 5.42 (br. s, 2H), 5.11 (s, 2H), 4.58 (d, *J* = 14.0 Hz, 1H), 4.54 (d, *J* = 13.7 Hz, 1H).

^{13}C NMR (101 MHz, $CDCl_3$) δ_C 158.8, 136.3, 131.4, 129.5, 129.1, 128.6, 128.3, 128.3, 127.7, 68.1, 61.2.

HRMS (ESI^+, m/z) calculated for $[C_{15}H_{16}N_2O_3SNa]^+$ $[M+Na]^+$ 327.0774, found 327.0782.

IR (thin film, ν_{max}/cm^{-1}) = 3236, 3065, 1665, 1381, 1286, 970, 758, 699.

Melting Point = 119 – 121 °C ($CHCl_3$).

Benzyl (amino(oxo)(vinyl)-λ^6-sulfaneylidene)carbamate (3r):

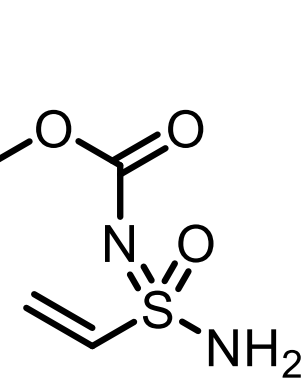

Prepared according to **General Procedure B** using *N*-alkoxysulfurdiimide **1c** (40.3 mg, 0.150 mmol, 1.00 equiv.), THF (1.50 mL) and vinylmagnesium bromide solution (0.18 mL, 0.165 mmol, 0.94 M in THF, 1.10 equiv.). Flash column chromatography (30% – 60% EtOAc in pentane) afforded sulfonimidamide **3r** as an off-white solid (18.4 mg, 51%).

R_f = 0.29 (60% EtOAc in pentane).

^{1}H NMR (400 MHz, $CDCl_3$) δ_H 7.44 – 7.28 (m, 5H), 6.78 (dd, *J* = 16.4, 9.8 Hz, 1H), 6.38 (d, *J* = 16.3 Hz, 1H), 6.02 (d, *J* = 9.8 Hz, 1H), 5.64 (br. s, 2H), 5.11 (s, 2H).

^{13}C NMR (101 MHz, $CDCl_3$) δ_C 158.4, 136.8, 136.1, 128.6, 128.3, 128.2, 127.6, 68.2.

HRMS (ESI$^+$, m/z) calculated for $[C_{10}H_{13}N_2O_3SNa]^+$ $[M+Na]^+$ 263.0461, found 263.0464.

IR (thin film, ν_{max}/cm^{-1}) = 3212, 3116, 2980, 1659, 1382, 1270, 968.

Melting Point = 60 – 62 °C ($CHCl_3$).

Benzyl (allyl(amino)(oxo)-λ^6-sulfaneylidene)carbamate (3s):

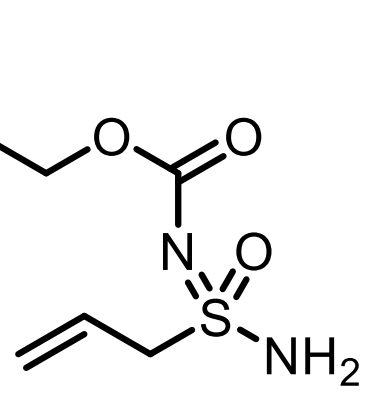

Prepared according to **General Procedure B** using *N*-alkoxysulfurdiimide **1c** (40.3 mg, 0.150 mmol, 1.00 equiv.), THF (1.50 mL) and allylmagnesium chloride solution (0.10 mL, 0.165 mmol, 1.66 M in THF, 1.10 equiv.). Flash column chromatography (20% – 50% EtOAc in pentane) afforded sulfonimidamide **3s** as a colourless solid (21.0 mg, 55%).

R_f = 0.19 (40% EtOAc in pentane).

^{1}H NMR (400 MHz, $CDCl_3$) δ_H 7.40 – 7.27 (m, 5H), 5.92 – 5.77 (m, 1H), 5.50 – 5.39 (m, 2H), 5.10 (s, 2H), 4.08 (d, *J* = 7.3 Hz, 1H), 4.06 (d, *J* = 7.3 Hz, 1H). (Note: NH_2 protons missing).

^{13}**C NMR** (101 MHz, $CDCl_3$) δ_C 158.7, 136.2, 128.6, 128.3, 128.2, 126.1, 124.3, 68.1, 59.6.

HRMS (ESI$^+$, m/z) calculated for $[C_{11}H_{14}N_2O_3SNa]^+$ $[M+Na]^+$ 277.0617, found 277.0625.

IR (thin film, ν_{max}/cm^{-1}) = 3237, 3071, 2358, 1664, 1382, 1288, 975, 758, 700.

Melting Point = 60 – 63 °C ($CHCl_3$).

Benzyl (amino(*tert*-butyl)(oxo)-λ^6-sulfaneylidene)carbamate (3t):

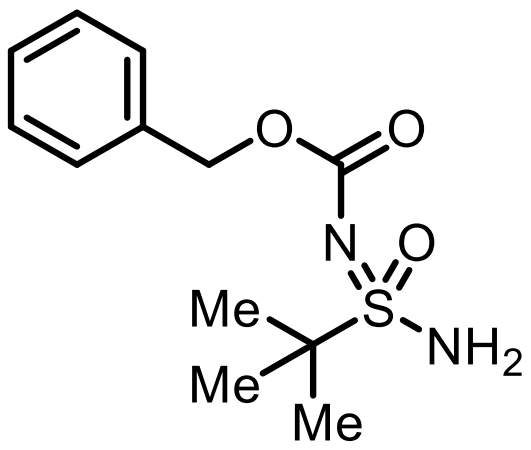

Prepared according to **General Procedure B** using *N*-alkoxysulfurdiimide **1c** (40.3 mg, 0.150 mmol, 1.00 equiv.), THF (1.50 mL) and *tert*-butylmagnesium chloride solution (0.17 mL, 0.165 mmol, 1.00 M in THF, 1.10 equiv.). Flash column chromatography (10% – 30% EtOAc in pentane) afforded sulfonimidamide **3t** as a colourless solid (20.8 mg, 51%).

R_f = 0.32 (40% EtOAc in pentane).

^{1}**H NMR** (400 MHz, $CDCl_3$) δ_H 7.47 – 7.20 (m, 5H), 5.67 (br. s, 2H), 5.14 (d, *J* = 12.5 Hz, 1H), 5.09 (d, *J* = 12.4 Hz, 1H), 1.45 (s, 9H).

^{13}**C NMR** (101 MHz, $CDCl_3$) δ_C 159.4, 136.5, 128.7, 128.6, 128.3, 128.2, 68.0, 62.0, 23.4.

HRMS (ESI$^+$, m/z) calculated for $[C_{12}H_{18}N_2O_3SNa]^+$ $[M+Na]^+$ 293.0930, found 293.0941.

IR (thin film, ν_{max}/cm^{-1}) = 3226, 2987, 2359, 1655, 1383, 1287, 920, 761.

Melting Point = 136 – 138 °C ($CHCl_3$).

Benzyl (amino(1-methyl-1*H*-indol-5-yl)(oxo)-λ^6-sulfaneylidene)carbamate (3u):

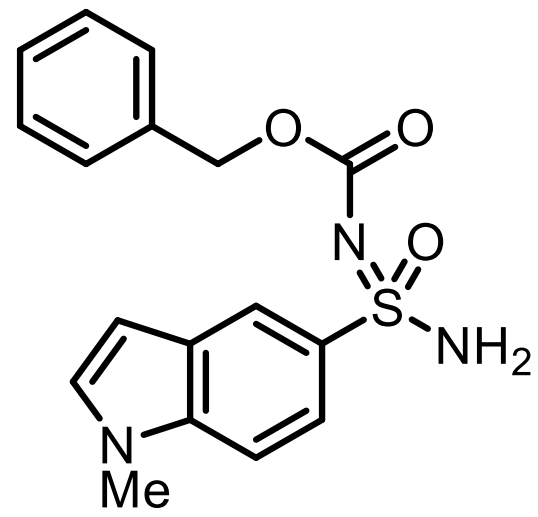

Magnesium turnings (146 mg, 6 mmol) were added to an oven-dried round-bottom flask. The vessel was then sealed with a rubber septum, evacuated and backfilled with N_2 (×3). Dry THF (5 mL) was added to the reaction mixture, along with an I_2 crystal. 5-Bromo-1-methyl-1*H*-indole (1.05 g, 5 mmol) in THF (1 mL) was added dropwise to the mixture before heating to reflux for 2 h.

Prepared according to **General Procedure B** using *N*-alkoxysulfurdiimide **1c** (40.3 mg, 0.150 mmol, 1.00 equiv.), THF (1.50 mL) and (1-methyl-1*H*-indol-5-yl)magnesium bromide solution (0.39 mL, 0.165 mmol, 0.42 M in THF, 1.10 equiv.). Flash column chromatography (30% – 50% EtOAc in pentane) afforded sulfonimidamide **3u** as an off-white solid (33.3 mg, 65%).

R$_f$ = 0.30 (60% EtOAc in pentane).

^{1}H NMR (400 MHz, $(CD_3)_2SO$) δ_H 8.14 (d, *J* = 2.0 Hz, 1H), 7.70 – 7.57 (m, 4H), 7.52 (d, *J* = 3.2 Hz, 1H), 7.32 – 7.23 (m, 3H), 7.18 (dd, *J* = 7.2, 2.5 Hz, 2H), 6.64 (d, *J* = 3.2 Hz, 1H), 4.90 (d, *J* = 12.9 Hz, 1H), 4.85 (d, *J* = 12.8 Hz, 1H), 3.85 (s, 3H).

^{13}C NMR (101 MHz $(CD_3)_2SO$) δ_C 157.2, 137.8, 137.2, 132.5, 132.2, 128.2, 127.5, 127.3, 127.0, 120.6, 119.4, 110.0, 102.0, 65.8, 32.8.

HRMS (ESI$^+$, m/z) calculated for $[C_{17}H_{17}N_3O_3SNa]^+$ $[M+Na]^+$ 366.0883, found 366.0879.

IR (thin film, ν_{max}/cm^{-1}) = 3211, 3063, 3033, 1662, 1262, 1061, 918, 759.

Melting Point = 122 – 126 °C (EtOAc).

Benzyl (amino(benzofuran-5-yl)(oxo)-λ^6-sulfaneylidene)carbamate (3v):

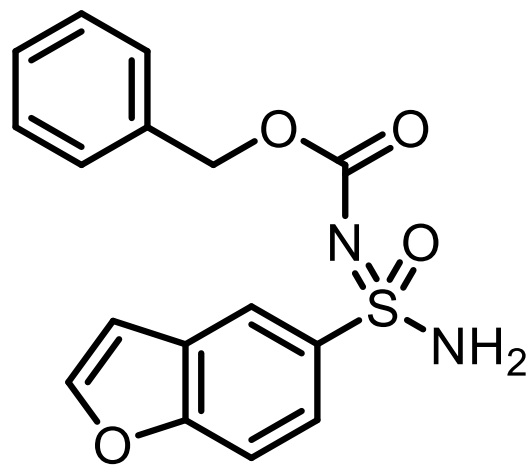

Magnesium turnings (146 mg, 6 mmol) were added to an oven-dried round-bottom flask. The vessel was then sealed with a rubber septum, evacuated and backfilled with N_2 (×3). Dry THF (0.4 mL) was added to the reaction mixture, along with 1,4-dibromoethane (6 drops). The mixture was stirred at rt for 10 mins. 5-Bromobenzofuran (0.63 mL, 5 mmol) in THF (4.6 mL) was added dropwise to the mixture before heating to 60 °C for 2 h.

Prepared according to **General Procedure B** using *N*-alkoxysulfurdiimide **1c** (40.3 mg, 0.150 mmol, 1.00 equiv.), THF (1.50 mL) and benzofuran-5-ylmagnesium bromide solution (0.49 mL, 0.165 mmol, 0.34 M in THF, 1.10 equiv.). Flash column chromatography (20% – 40% EtOAc in pentane) afforded sulfonimidamide **3v** as an off-white solid (37.8 mg, 76%).

R$_f$ = 0.51 (60% EtOAc in pentane).

^{1}H NMR (400 MHz, $CDCl_3$) δ_H 8.28 (d, *J* = 2.1 Hz, 1H), 7.91 (dd, *J* = 8.7, 2.0 Hz, 1H), 7.74 (d, *J* = 2.3 Hz, 1H), 7.54 (d, *J* = 8.8 Hz, 1H), 7.30 – 7.16 (m, 5H), 6.82 (d, *J* = 2.3 Hz, 1H), 6.03 (br.s, 2H), 5.04 (d, *J* = 12.3 Hz, 1H), 4.97 (d, *J* = 12.4 Hz, 1H).

^{13}C NMR (101 MHz, $CDCl_3$) δ_C 158.2, 157.2, 147.5, 136.2, 135.0, 128.5, 128.1, 128.1, 127.9, 123.6, 122.1, 112.3, 107.4, 67.9.

HRMS (ESI$^+$, m/z) calculated for $[C_{16}H_{15}N_2O_4S]^+$ $[M+H]^+$ 331.0747, found 331.0739.

IR (thin film, ν_{max}/cm^{-1}) = 3226, 3067, 2981, 1663, 1454, 1380, 1259, 1028, 920.

Melting Point = 60 – 64 °C ($CHCl_3$).

Benzyl (amino(oxo)(thiophen-2-yl)-λ^6-sulfaneylidene)carbamate (3w):

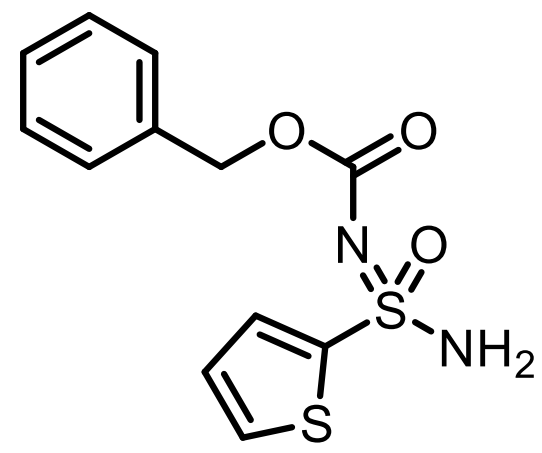

Prepared according to **General Procedure B** using *N*-alkoxysulfurdiimide **1c** (40.3 mg, 0.150 mmol, 1.00 equiv.), THF (1.50 mL) and 2-thienylmagnesium bromide solution (0.17 mL, 0.165 mmol, 0.97 M in THF, 1.10 equiv.). Flash column chromatography (20% – 50% EtOAc in pentane) afforded sulfonimidamide **3w** as a beige solid (21.0 mg, 47%).

R_f = 0.35 (40% EtOAc in pentane).

^{1}H NMR (400 MHz, $(CD_3)_2SO$) δ_H 8.08 (br. s, 2H), 7.93 (dd, *J* = 4.9, 1.4 Hz, 1H), 7.63 (dd, *J* = 3.7, 1.4 Hz, 1H), 7.37 – 7.22 (m, 5H), 7.16 (dd, *J* = 5.0, 3.8 Hz, 1H), 4.97 (d, *J* = 12.9 Hz, 1H), 4.93 (d, *J* = 12.7 Hz, 1H).

^{13}C NMR (101 MHz $(CD_3)_2SO$) δ_C 156.8, 143.5, 137.0, 133.1, 132.1, 128.3, 127.7, 127.5, 127.5, 66.2.

HRMS (ESI$^+$, m/z) calculated for $[C_{12}H_{12}N_2O_3S_2]^+$ $[M+H]^+$ 297.0362, found 297.0362.

IR (thin film, ν_{max}/cm^{-1}) = 3234, 3069, 2925, 2361, 1676, 1381, 1281, 1100, 977, 764.

Melting Point = 130 –133 °C (EtOAc).

Benzyl (amino(1-methyl-1*H*-pyrazol-4-yl)(oxo)-λ^6-sulfaneylidene)carbamate (3x):

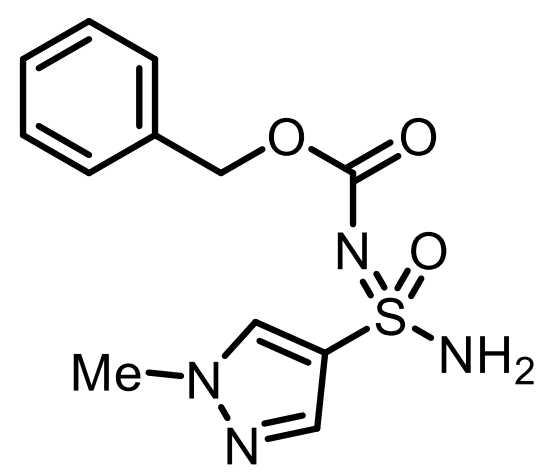

4-Iodo-1-methyl-1*H*-pyrazole (34.3 mg, 0.165 mmol, 1.10 equiv.) was weighed into an oven-dried reaction vessel. The vessel was then sealed with a rubber septum, evacuated and backfilled with N_2 (×3). Dry THF (0.22 mL) was added and the reaction mixture cooled to -20 °C. Isopropylmagnesium chloride solution (0.08 mL, 0.165 mmol, 2.08 M in THF, 1.10 equiv.) was added dropwise and the reaction mixture stirred at -20 °C for 1 h.

N-Alkoxysulfurdiimide **1c** (40.3 mg, 0.150 mmol, 1.00 equiv.) was weighed into an oven-dried reaction vessel. The vessel was then sealed with a rubber septum, evacuated and backfilled with N_2 (×3). Dry THF (1.50 mL) was added and the solution was added to the organometallic-containing vessel at -20 °C dropwise. The reaction mixture was warmed to rt in a water bath and stirred for 30 mins. The reaction was quenched with silica and the crude product was purified directly by flash column chromatography (20% – 80% EtOAc in pentane) to afford sulfonimidamide **3x** as an off-white solid (31.4 mg, 71%).

R_f = 0.07 (60% EtOAc in pentane).

^{1}H NMR (400 MHz, $(CD_3)_2SO$) δ_H 8.26 (s, 1H), 7.74 (s, 3H), 7.31 (dt, *J* = 24.2, 7.3 Hz, 5H), 4.96 (d, *J* = 12.7 Hz, 1H), 4.91 (d, *J* = 12.7 Hz, 1H), 3.87 (s, 3H).

^{13}C NMR (101 MHz $(CD_3)_2SO$) δ_C 157.2, 138.2, 137.2, 133.0, 128.4, 127.7, 127.6, 123.5, 66.0, 39.1.

HRMS (ESI$^+$, m/z) calculated for $[C_{12}H_{14}N_4O_3SNa]^+$ $[M+Na]^+$ 317.0679, found 317.0685.

IR (thin film, ν_{max}/cm^{-1}) = 3203, 3125, 3034, 2953, 1662, 1523, 1381, 1265, 976, 702.

Melting Point = 94 – 97 °C (EtOAc).

6-Bromo-*N*'-(diisopropylcarbamoyl)pyridine-2-sulfonimidamide (3y):

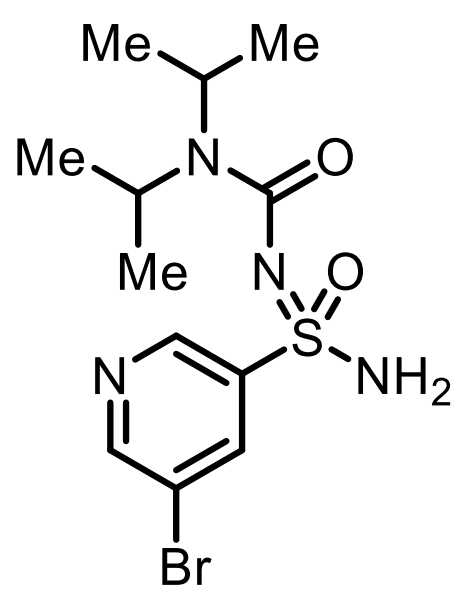

An oven-dried reaction vessel was sealed with a rubber septum, evacuated and backfilled with N_2 (×3). Isopropylmagnesium chloride lithium chloride complex (0.13 mL, 0.165 mmol, 1.28 M in THF, 1.10 equiv.) was added and cooled to -15 °C. 3,5-Dibromopyridine (39.1 mg, 0.165 mmol, 1.10 equiv.) was added and the reaction mixture stirred for 40 min at the same temperature. A solution of *N*-alkoxysulfurdiimide **1i** (39.3 mg, 0.150 mmol, 1.00 equiv.) in THF (1.5 mL) was added at -15 °C and then warmed to rt and stirred for 30 mins. The crude reaction mixture was purified directly by flash column chromatography (0% – 40% EtOAc in pentane) to afford sulfonimidamide **3y** as a colourless solid (36.7 mg, 67%).

R$_f$ = 0.64 (50% EtOAc in pentane).

^{1}H NMR (400 MHz, $(CD_3)_2SO$) δ_H 8.91 (d, *J* = 2.1 Hz, 1H), 8.88 (d, *J* = 1.9 Hz, 1H), 8.30 (dd, *J* = 2.1 Hz, 2.1 Hz, 1H), 7.56 (br. s, 2H), 4.22 (s, 1H), 3.64 (s, 1H), 1.28 – 0.98 (m, 12H).

^{13}C NMR (101 MHz, $(CD_3)_2SO$) δ_C 157.1, 152.7, 145.5, 142.3, 136.4, 119.7, 46.6, 43.9, 21.0, 20.6. (Note: additional peaks due to rotamers caused by the diisopropyl urea group).

HRMS (ESI$^+$, m/z) calculated for $[C_{12}H_{20}{}^{79}BrN_4O_2S]^+$ $[M+H]^+$ 363.0485, found 363.0485; $[C_{12}H_{20}{}^{81}BrN_4O_2S]^+$ $[M+H]^+$ 365.0464, found 365.0441.

IR (thin film, ν_{max}/cm^{-1}) = 3396, 2960, 2925, 2854, 1648, 1597, 1436, 1312, 1264, 1023, 773, 689, 660, 614.

Melting Point = 164 – 167 °C (EtOAc).

***N*'-(Diisopropylcarbamoyl)-5-morpholinopyridine-3-sulfonimidamide (3z):**

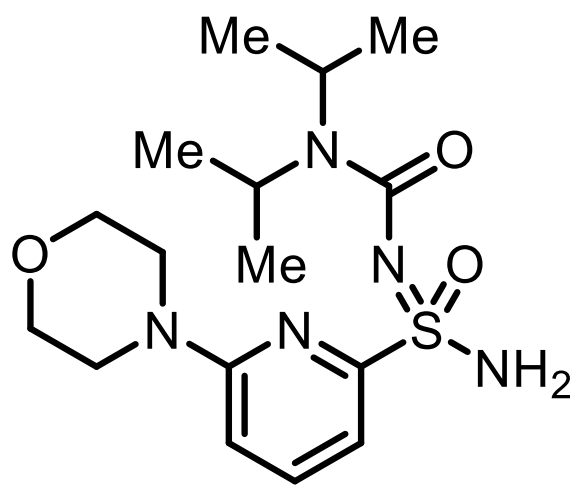

2-Bromo-6-morpholinopyridine (40.1 mg, 0.165 mmol, 1.10 equiv.) was added to an oven-dried round bottom flask. The vessel was then sealed with a rubber septum, evacuated and backfilled with N_2 (×3). Dry THF (0.75 mL) was added and the reaction mixture cooled to -78 °C. *n*-BuLi solution (0.07 mL, 0.165 mmol, 2.30 M in hexanes, 1.10 equiv.) was added dropwise and the reaction mixture stirred at -78 °C for 1 h. Then a solution of *N*-alkoxysulfurdiimide **1i** (39.3 mg, 0.150 mmol, 1.00 equiv.) in THF (0.75 mL) was added at -78 °C and then warmed to rt and stirred for 30 mins. The crude reaction mixture was purified directly by flash column chromatography (0% – 60% EtOAc in pentane) to afford sulfonimidamide **3z** as a colourless solid (22.2 mg, 40%).

R$_f$ = 0.28 (60% EtOAc in pentane).

^{1}H NMR (400 MHz, $(CD_3)_2SO$) δ_H 7.74 (dd, *J* = 8.0 Hz, 8.0 Hz, 1H), 7.23 (br. s, 2H), 7.14 (d, *J* = 7.3 Hz, 1H), 6.98 (d, *J* = 8.6 Hz, 1H), 4.22 (s, 1H), 3.67 (m, 5H), 3.57 (m, 4H), 1.13 (m, 12H).

^{13}C NMR (101 MHz, $(CD_3)_2SO$) δ_C 158.2, 157.5, 139.0, 109.7, 109.3, 65.8, 46.2, 44.8, 43.8, 21.0, 20.8. (Note: additional peaks due to rotamers caused by the diisopropyl urea group).

HRMS (ESI$^+$, m/z) calculated for $[C_{16}H_{28}N_5O_3S]^+$ $[M+H]^+$ 370.1907, found 370.1899.

IR (thin film, ν_{max}/cm^{-1}) = 3212, 2967, 2930, 2860, 1595, 1476, 1447, 1333, 1255, 1156, 1118.

Melting Point = 104 – 106 °C (EtOAc).

***N*'-(Diisopropylcarbamoyl)-[1,1'-biphenyl]-4-sulfonimidamide (3aa):**

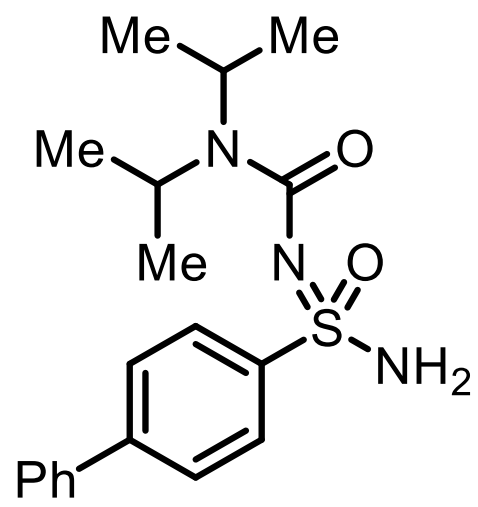

Magnesium turnings (360 mg, 15 mmol) were added to an oven-dried round-bottom flask. The vessel was then sealed with a rubber septum, evacuated and backfilled with N_2 (×3). Dry THF (10 mL) was added to the reaction mixture, along with an I_2 crystal. 4-Bromo-1,1'-biphenyl (2.33 g, 10.0 mmol) in THF (1 mL) was added dropwise to the mixture before stirring at rt for 3 h.

Prepared according to **General Procedure B** using *N*-alkoxysulfurdiimide **1i** (39.2 mg, 0.150 mmol, 1.00 equiv.), THF (1.50 mL) and 4-biphenylmagnesium bromide solution (0.20 mL, 0.165 mmol, 0.83 M in THF, 1.10 equiv.). Flash column chromatography (0% – 20% EtOAc in pentane) afforded sulfonimidamide **3aa** as a colourless solid (43.5 mg, 81%).

A scale up of this reaction was performed according to **General Procedure B** using *N*-alkoxysulfurdiimide **1i** (261.4 mg, 1.00 mmol, 1.00 equiv.), THF (10 mL) and 4-biphenylmagnesium bromide solution (1.33 mL, 1.10 mmol, 0.83 M in THF, 1.10 equiv.). Flash column chromatography (0% – 20% EtOAc in pentane) afforded **3aa** as a colourless solid (0.25 g, 70%).

$\mathbf{R_f}$ = 0.35 (20% EtOAc in pentane).

^{1}H NMR (400 MHz, $(CD_3)_2SO$) δ_H 7.98 – 7.80 (m, 4H), 7.74 (d, *J* = 7.6 Hz, 2H), 7.51 (t, *J* = 7.6 Hz, 2H), 7.43 (t, *J* = 7.3 Hz, 1H), 7.37 (br. s, 2H), 4.24 (s, 1H), 3.69 (s, 1H), 1.15 (m, 12H).

^{13}C NMR (101 MHz, $(CD_3)_2SO$) δ_C 157.4, 143.3, 143.2, 138.7, 129.1, 128.3, 127.0, 126.9, 126.8, 46.3, 43.8, 21.1, 20.8.

HRMS (ESI$^+$, m/z) calculated for $[C_{19}H_{25}N_3O_2SNa]^+$ $[M+Na]^+$ 382.1560, found 382.1573.

IR (thin film, ν_{max}/cm^{-1}) = 3318, 3074, 2970, 2932, 1563, 1481, 1442, 1323, 1263, 1135, 1034.

Melting Point = 142 – 146 °C (EtOAc).

Benzyl (amino(oxo)(4-(5-(*p*-tolyl)-3-(trifluoromethyl)-1*H*-pyrazol-1-yl)phenyl)-λ⁶-sulfaneylidene)carbamate (3ab):

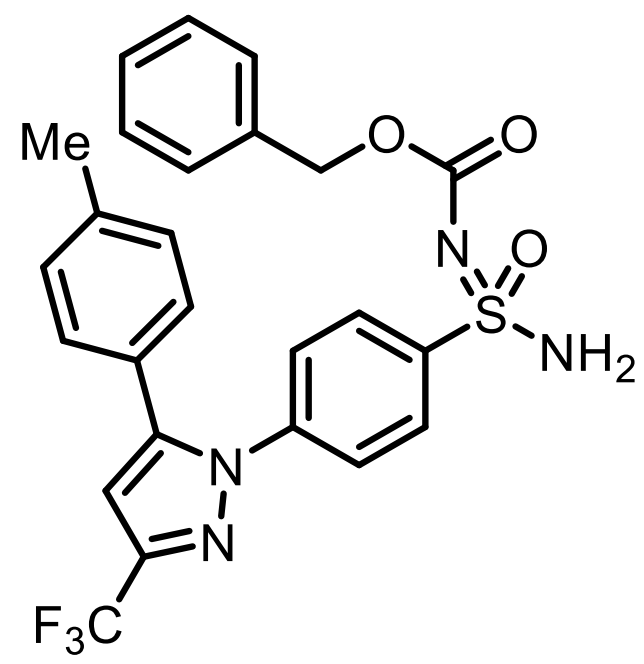

Magnesium turnings (87.6 mg, 3.6 mmol) were added to an oven-dried round-bottom flask. The vessel was then sealed with a rubber septum, evacuated and backfilled with N_2 (×3). Dry THF (3 mL) was added to the reaction mixture, along with an I_2 crystal. 1-(4-Bromophenyl)-5-(*p*-tolyl)-3-(trifluoromethyl)-1*H*-pyrazole (1.14 g, 3 mmol) in THF (1 mL) was added dropwise to the mixture before heating to reflux for 2 h.

Prepared according to **General Procedure B** using *N*-alkoxysulfurdiimide **1c** (40.3 mg, 0.150 mmol, 1.00 equiv.), THF (1.50 mL) and (4-(5-(*p*-tolyl)-3-(trifluoromethyl)-1*H*-pyrazol-1-yl)phenyl)magnesium bromide solution (0.61 mL, 0.165 mmol, 0.27 M in THF, 1.10 equiv.). Flash column chromatography (0% – 40% EtOAc in pentane) afforded sulfonimidamide **3ab** as an off-white solid (56.8 mg, 74%).

R_f = 0.46 (40% EtOAc in pentane).

^{1}H NMR (400 MHz, $CDCl_3$) δ_H 7.92 (d, *J* = 8.6 Hz, 2H), 7.41 (d, *J* = 8.5 Hz, 2H), 7.33 – 7.22 (m, 5H), 7.17 (d, *J* = 7.9 Hz, 2H), 7.10 (d, *J* = 7.9 Hz, 2H), 6.74 (s, 1H), 6.10 (br. s, 2H), 5.02 (s, 2H), 2.37 (s, 3H).

^{13}C NMR (101 MHz, $CDCl_3$) δ_C 157.9, 145.4, 144.3 (q, *J* = 38.6 Hz), 143.2, 140.0, 139.9, 136.0, 129.9, 128.9, 128.6, 128.4, 128.3, 128.2, 125.8, 125.4, 121.1 (q, *J* = 270.8 Hz), 106.6 (q, *J* = 1.8 Hz), 68.2, 21.5.

^{19}F NMR (377 MHz, $CDCl_3$) δ_F -62.4 (s).

HRMS (ESI$^+$, m/z) calculated for $[C_{25}H_{22}F_3N_4O_3S]^+$ $[M+H]^+$ 515.1359, found 515.1366.

IR (thin film, ν_{max}/cm^{-1}) = 3225, 3069, 3034, 1666, 1472, 1270, 1237, 1163, 1134, 1099, 976.

Melting Point = 149 – 151 °C ($CHCl_3$).

***N*'-(Diisopropylcarbamoyl)-4-ethoxy-3-(1-methyl-7-oxo-3-propyl-4,7-dihydro-1*H*-pyrazolo[4,3-*d*]pyrimidin-5-yl)benzenesulfonimidamide (3ac):**

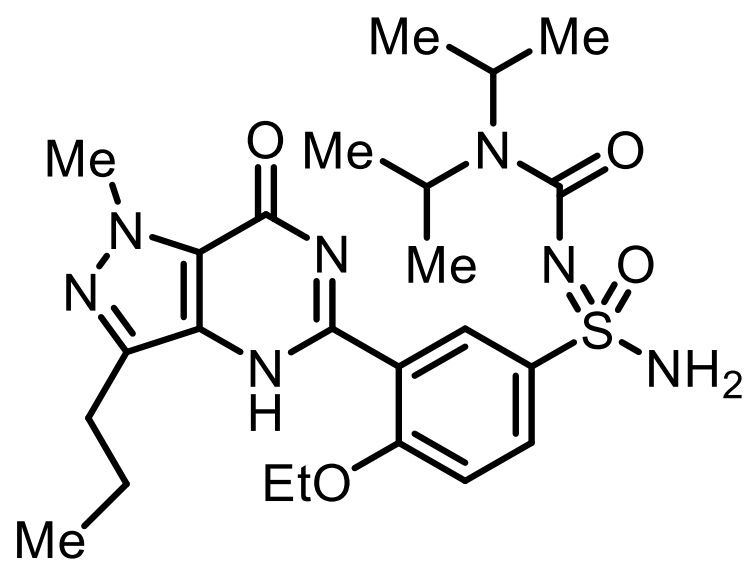

5-(5-Bromo-2-ethoxyphenyl)-1-methyl-3-propyl-1,6-dihydro-7*H*-pyrazolo[4,3-*d*]pyrimidin-7-one (64.9 mg, 0.165 mmol, 1.10 equiv. was added to an oven-dried round bottom flask. The vessel was then sealed with a rubber septum, evacuated and backfilled with N_2 (×3). Dry THF (0.83 mL) was added and the reaction mixture cooled to -78 °C. MeLi solution (0.12 mL, 0.18 mmol, 1.49 M in Et_2O, 1.2 equiv.) was added dropwise and stirred at -78 °C for 10 mins. *n*-BuLi solution (0.10 mL, 0.23 mmol, 2.30 M in hexanes, 1.5 equiv.) was added dropwise and the reaction mixture stirred at -78 °C for a further 50 mins. A solution of *N*-alkoxysulfurdiimide **1i** (60.1 mg, 0.23 mmol, 1.5 equiv.) in THF (1.50 mL) was added at -78 °C and then warmed to rt and stirred for 30 mins. The crude reaction mixture was purified directly by flash column chromatography (10% – 80% EtOAc in pentane) to afford sulfonimidamide **3ac** as a colourless solid (24.7 mg, 32%).

$\mathbf{R_f}$ = 0.21 (80% EtOAc in pentane).

^{1}H NMR (400 MHz, $CDCl_3$) δ_H 10.82 (s, 1H), 8.98 (d, *J* = 2.6 Hz, 1H), 8.03 (dd, *J* = 8.8, 2.6 Hz, 1H), 7.09 (d, *J* = 8.9 Hz, 1H), 6.50 (br. s, 2H), 4.34 (q, *J* = 7.0 Hz, 2H), 4.26 (s, 4H), 3.85 (br. s, 1H), 2.99 – 2.80 (m, 2H), 1.83 (h, *J* = 7.4 Hz, 2H), 1.62 (t, *J* = 7.0 Hz, 3H), 1.35 – 1.16 (m, 12H), 1.01 (t, *J* = 7.4 Hz, 3H).

^{13}C NMR (101 MHz, $CDCl_3$) δ_C 159.2, 158.4, 153.8, 147.1, 146.8, 138.5, 136.5, 130.6, 130.1, 124.6, 121.0, 113.2, 66.2, 47.2, 45.3, 38.4, 27.7, 22.5, 21.3, 20.9, 14.7, 14.1. (Note: additional peaks due to rotamers caused by the diisopropyl urea group).

HRMS (ESI$^+$, m/z) calculated for $[C_{24}H_{36}N_7O_4S]^+$ $[M+H]^+$ 518.2544, found 518.2537.

IR (thin film, ν_{max}/cm^{-1}) = 3298, 2964, 2932, 2873, 1689, 1586, 1489, 1469, 1436, 1322, 1273, 1246, 1153.

Melting Point = 142 – 146 °C (EtOAc).

4.5 *N*-Deprotection of sulfonimidamide 3a and 3aa

[1,1'-Biphenyl]-4-sulfonimidamide (4):

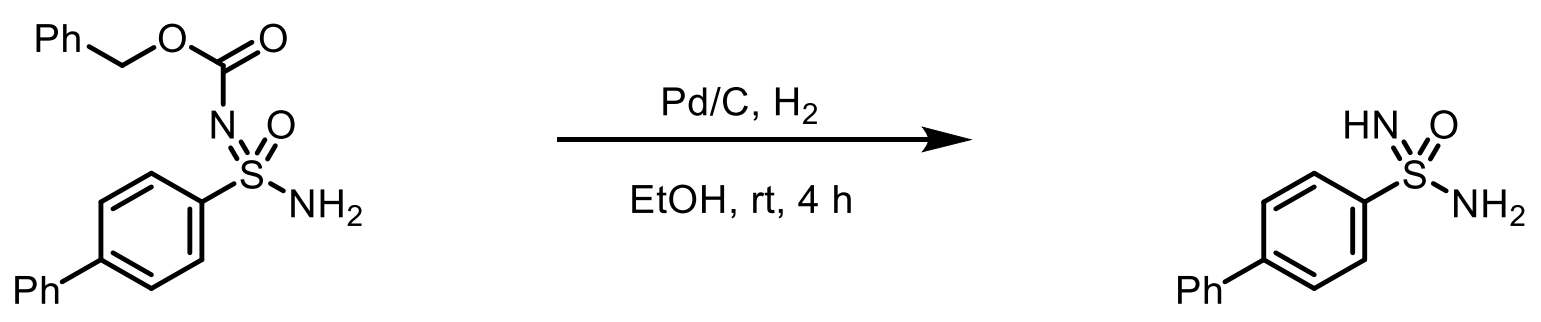

Sulfonimidamide **3a** (45.0 mg, 0.120 mmol, 1.00 equiv.) was dissolved in EtOH (2.50 mL) at rt. To the solution was added 10 wt% palladium on activated charcoal, moistened with water (65.0 mg, 61.0 µmol, 10% Wt, 0.50 equiv.). The vial was transferred to a Cat96 reactor and evacuated and purged with nitrogen (×3) before being exposed to an atmosphere of hydrogen (5 bar) generated by H-Genie equipment. The reaction was stirred at rt under an atmosphere of hydrogen (5 bar) for 2 h. The reaction vial was evacuated and purged with nitrogen (×3) before a further portion of 10 wt% palladium on activated charcoal, moistened with water (65.0 mg, 61.0 µmol, 10% Wt, 0.50 equiv.) was added at rt. The vial was returned to the Cat96 reactor and evacuated and purged with nitrogen (×3) before being exposed to an atmosphere of hydrogen (5 bar) generated by H-Genie equipment. The reaction was stirred at rt under an atmosphere of hydrogen (5 bar) for 2 h. The reaction vial was evacuated and purged with nitrogen (×3) before being filtered over celite. The celite was washed with EtOH (2 × 3 mL) and the filtrates combined and concentrated in vacuo to afford sulfonimidamide **4** (24.0 mg, 80 %) as an off-white solid.

^{1}H NMR (400 MHz, $(CD_3)_2SO$) δ_H 8.00 – 7.95 (m, 2H), 7.83 – 7.78 (m, 2H), 7.74 – 7.70 (m, 2H), 7.53 – 7.47 (m, 2H), 7.45 – 7.39 (m, 1H), 5.92 (br. s, 3H).

^{13}C NMR (151 MHz, $(CD_3)_2SO$) δ_C 145.6, 142.5, 139.0, 129.1, 128.2, 127.0, 126.7, 126.6.

LRMS (ESI, m/z) $[M+H]^+$ 233.0.

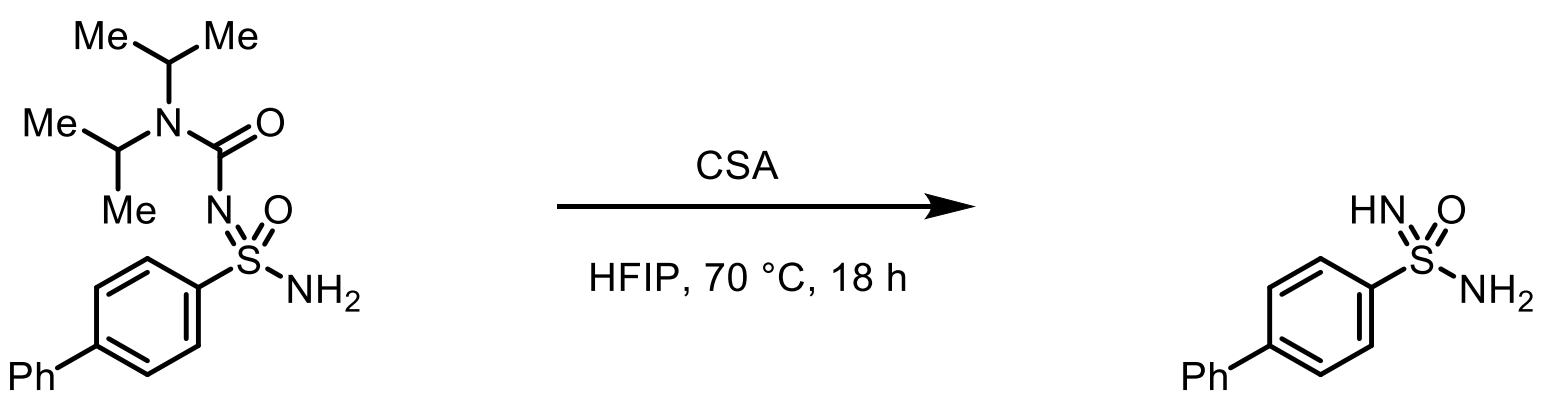

Sulfonimidamide **3aa** (61 mg, 0.17 mmol, 1.00 equiv.) was dissolved in HFIP (1.7 mL). Camphorsulfonic acid (79 mg, 0.34 mmol, 2.00 equiv.) was added and the reaction vessel was sealed and heated to 70 °C in an oil bath for 48 h. The reaction was then cooled to rt and adjusted to pH 7 with saturated

aqueous $NaHCO_3$ solution. The reaction mixture was extracted with EtOAc (x4), washed with brine, dried over Na_2SO_4 and concentrated in vacuo. The crude product was purified by flash column chromatography (50% EtOAc in pentane to 100% EtOAc) to afford sulfonimidamide **4** (20 mg, 51 %) as a colourless solid.

Characterisation data consistent with above.

5. Single Crystal X-ray Diffraction

Single crystals of **1c** suitable for X-ray diffraction were obtained from a pentane solution. The single-crystal X-ray structure of **1c** was determined by the Chemical Crystallography Service at the Chemistry Research Laboratory, Department of Chemistry, University of Oxford. Data were collected on a Rigaku Oxford Diffraction Synergy DW diffractometer equipped with a MicroMax-007 HF microfocus rotating-anode X-ray generator (Cu Kα radiation, λ = 1.5418 Å) and a HyPix-Arc 150° pixel detector. A suitable crystal was selected under Fomblin® YR 1800 oil, mounted on a MiTeGen loop, and cooled to 150 K using an Oxford Cryosystems nitrogen-flow device.[10] Data collection, indexing, integration, and multi-scan absorption correction were carried out using CrysAlisPro. Unit-cell parameters were refined against all suitable reflections.

The structure was solved using SHELXT,[11] and refined by full-matrix least-squares on F^2 using the CRYSTALS refinement suite.[12] The asymmetric unit contains two independent molecules. The refined Flack parameter is consistent with the presence of a single enantiomer in the measured crystal, and the absolute configuration (hand) shown corresponds to that determined crystallographically. Crystal data and structure refinement details for **1c** are provided in Supporting Table S12.

Crystallographic data for compound **1c** have been deposited with the Cambridge Crystallographic Data Centre (CCDC) under deposition number **CCDC-2544780**. These data can be obtained free of charge via www.ccdc.cam.ac.uk/data_request/cif.

Table S12: Crystal data and structure refinement for **1c**.

Identification code	**1c**	
Empirical formula	C12 H16 N2 O3 S	
Formula weight	268.34	
Temperature	100 K	
Wavelength	1.54184 Å	
Crystal system	Triclinic	
Space group	P -1	
Unit cell dimensions	a = 6.02960(10) Å	a= 89.3500(10)°.
	b = 9.6313(2) Å	b= 79.1340(10)°.
	c = 11.5112(2) Å	g = 85.0890(10)°.
Volume	654.09(2) $Å^3$	
Z	2	
Density (calculated)	1.362 Mg/m^3	
Absorption coefficient	2.237 mm^{-1}	
F(000)	284	
Crystal size	0.293 x 0.267 x 0.080 mm^3	
Theta range for data collection	3.910 to 75.049°.	
Index ranges	-7<=h<=7, -11<=k<=12, -14<=l<=14	
Reflections collected	17807	
Independent reflections	2669 [R(int) = 0.026]	
Completeness to theta = 73.548°	99.5 %	
Absorption correction	Semi-empirical from equivalents	
Max. and min. transmission	0.84 and 0.20	
Refinement method	Full-matrix least-squares on F^2	
Data / restraints / parameters	2669 / 0 / 163	
Goodness-of-fit on F^2	1.0052	
Final R indices [I>2sigma(I)]	R1 = 0.0277, wR2 = 0.0726	
R indices (all data)	R1 = 0.0280, wR2 = 0.0729	
Largest diff. peak and hole	0.19 and -0.38 e.$Å^{-3}$	

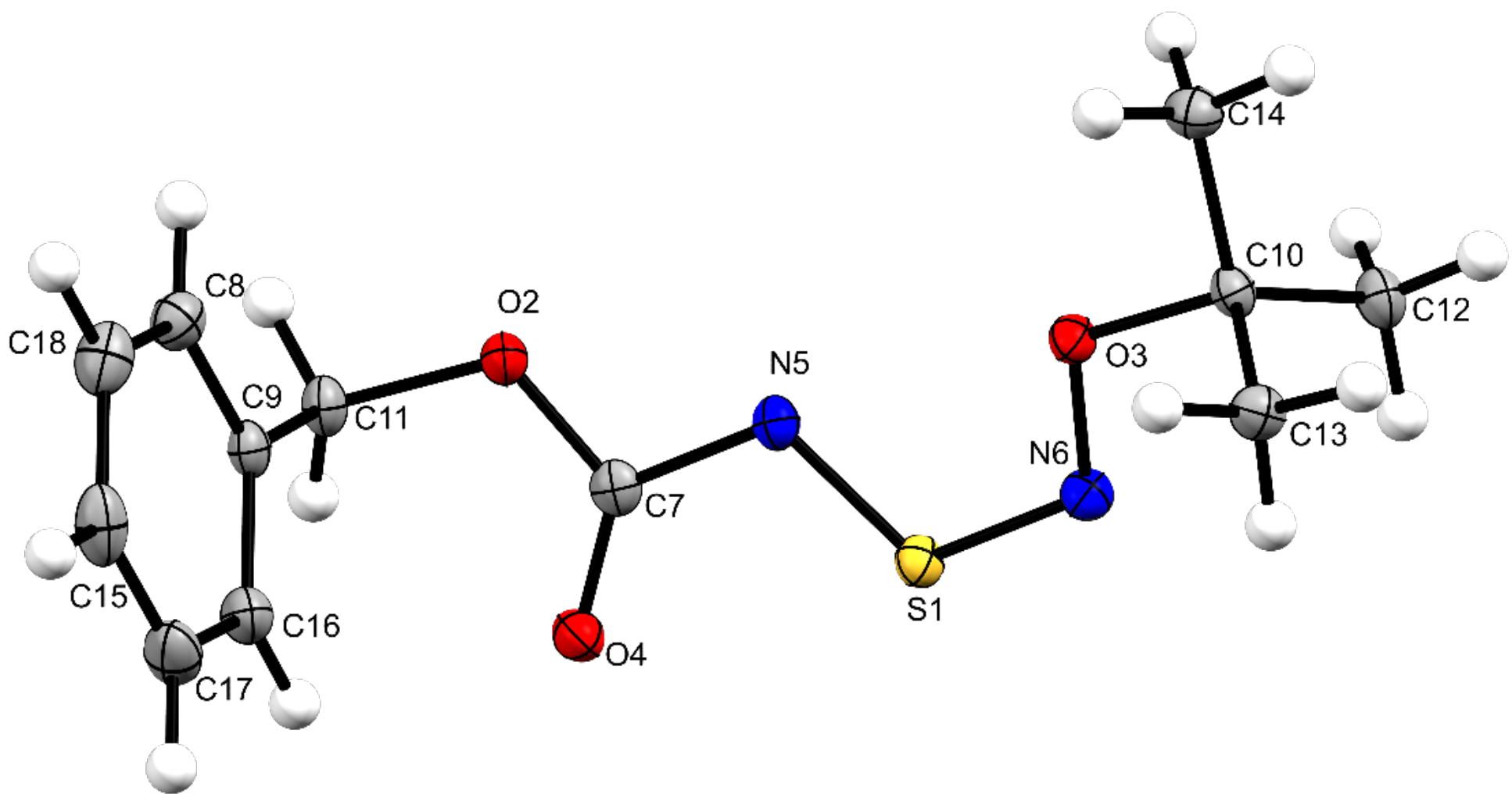

6. References

[1] M. Benohoud, L. Leman, S. H. Cardoso, P. Retailleau, P. Dauban, J. Thierry, R. H. Dodd, *J. Org. Chem.* **2009**, *74*, 5331–5336.

[2] S. R. Pollack, A. Dion, *J. Org. Chem.* **2021**, *86*, 11748–11762.

[3] I. D. Inaloo, S. Majnooni, M. Esmaeilpour, *Eur. J. Org. Chem.* **2018**, *2018*, 3481–3488.

[4] B. E. Love, E. G. Jones, *J. Org. Chem.* **1999**, *64*, 3755–3756.

[5] A. L. Krasovskiy, P. Knochel, *Synthesis* **2006**, *2006*, 0890–0891.

[6] a) Q.-W. Zhao, Z.-F. Yang, X.-P. Fu, X. Zhang, *Synlett* **2020**, *32*, 1565–1569; b) F. Zhao, X.-W. Gu, R. Franke, X.-F. Wu, *Angew. Chem. Int. Ed.* **2022**, *61*, e202214812; c) T. Q. Davies, A. Hall, M. C. Willis, *Angew. Chem. Int. Ed. Engl.* **2017**, *56*, 14937–14941; d) O. Rabal, J. A. Sánchez-Arias, M. Cuadrado-Tejedor, I. de Miguel, M. Pérez-González, C. García-Barroso, A. Ugarte, A. Estella-Hermoso de Mendoza, E. Sáez, M. Espelosin, S. Ursua, T. Haizhong, W. Wei, X. Musheng, A. Garcia-Osta, J. Oyarzabal, *J. Med. Chem.* **2016**, *59*, 8967–9004.

[7] P. Wu, J. Demaerel, B. J. Statham, C. Bolm, *Chem. Sci.* **2024**, *15*, 5333–5339.

[8] B. Y. Li, K. Su, L. Van Meervelt, S. H. L. Verhelst, E. Ismalaj, W. M. De Borggraeve, J. Demaerel, *Angew. Chem. Int. Ed. Engl.* **2023**, *62*, e202305093.

[9] G. S. Borovikova, E. S. Levchenko, E. I. Borovik, *Zh. Org. Khim.* **1979**, *15*, 2485−2490.

[10] J. Cosier, A. M. Glazer, *J. Appl. Crystallogr.* **1986**, *19*, 105–107.

[11] G. Sheldrick, *Acta Crystallographica Section A* **2015**, *71*, 3–8.

[12] a) P. Parois, R. I. Cooper, A. L. Thompson, *Chem. Cent. J.* **2015**, *9*, 30; b) P. W. Betteridge, J. R. Carruthers, R. I. Cooper, K. Prout, D. J. Watkin, *J. Appl. Crystallogr.* **2003**, *36*, 1487.

7. NMR Spectra

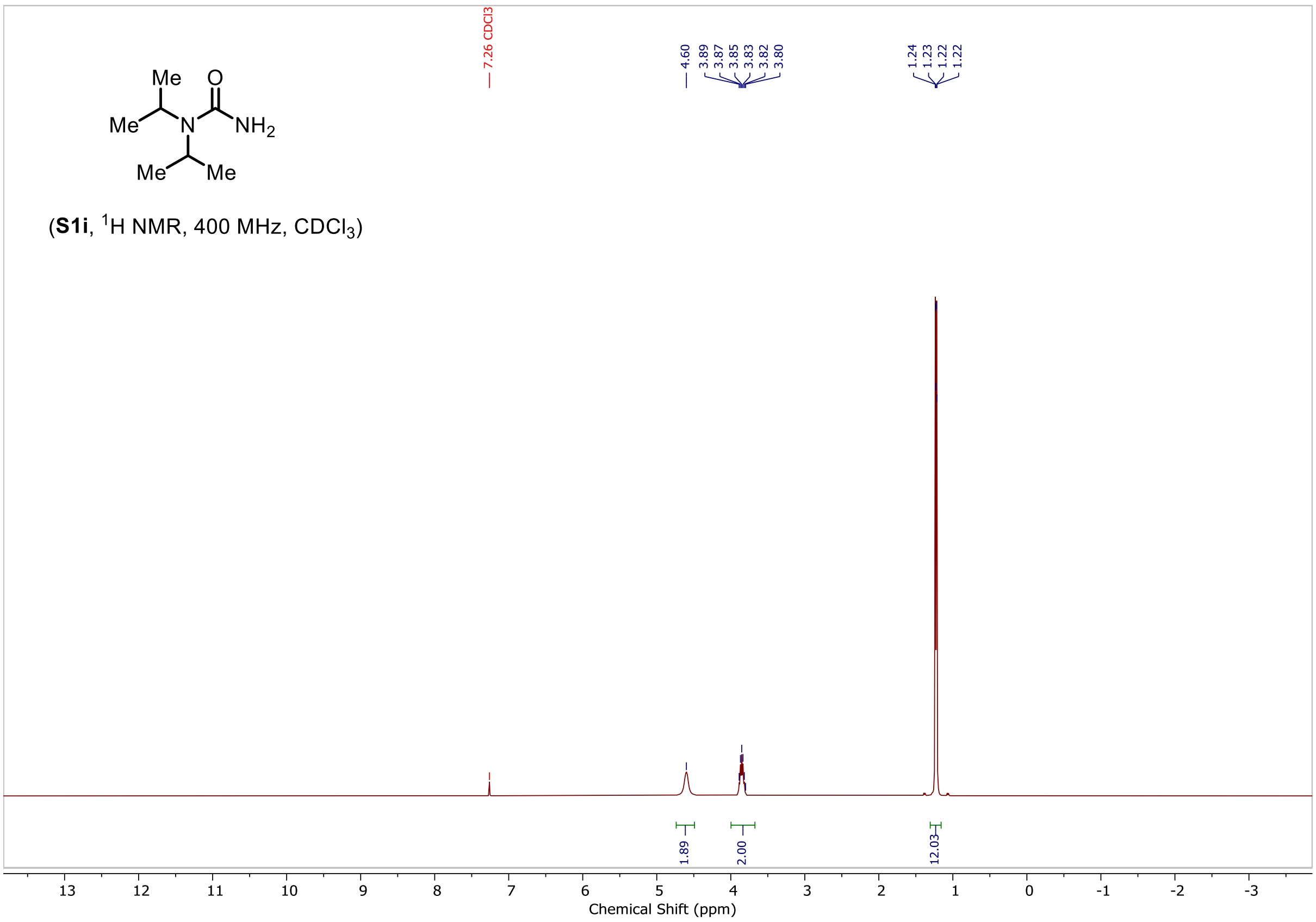

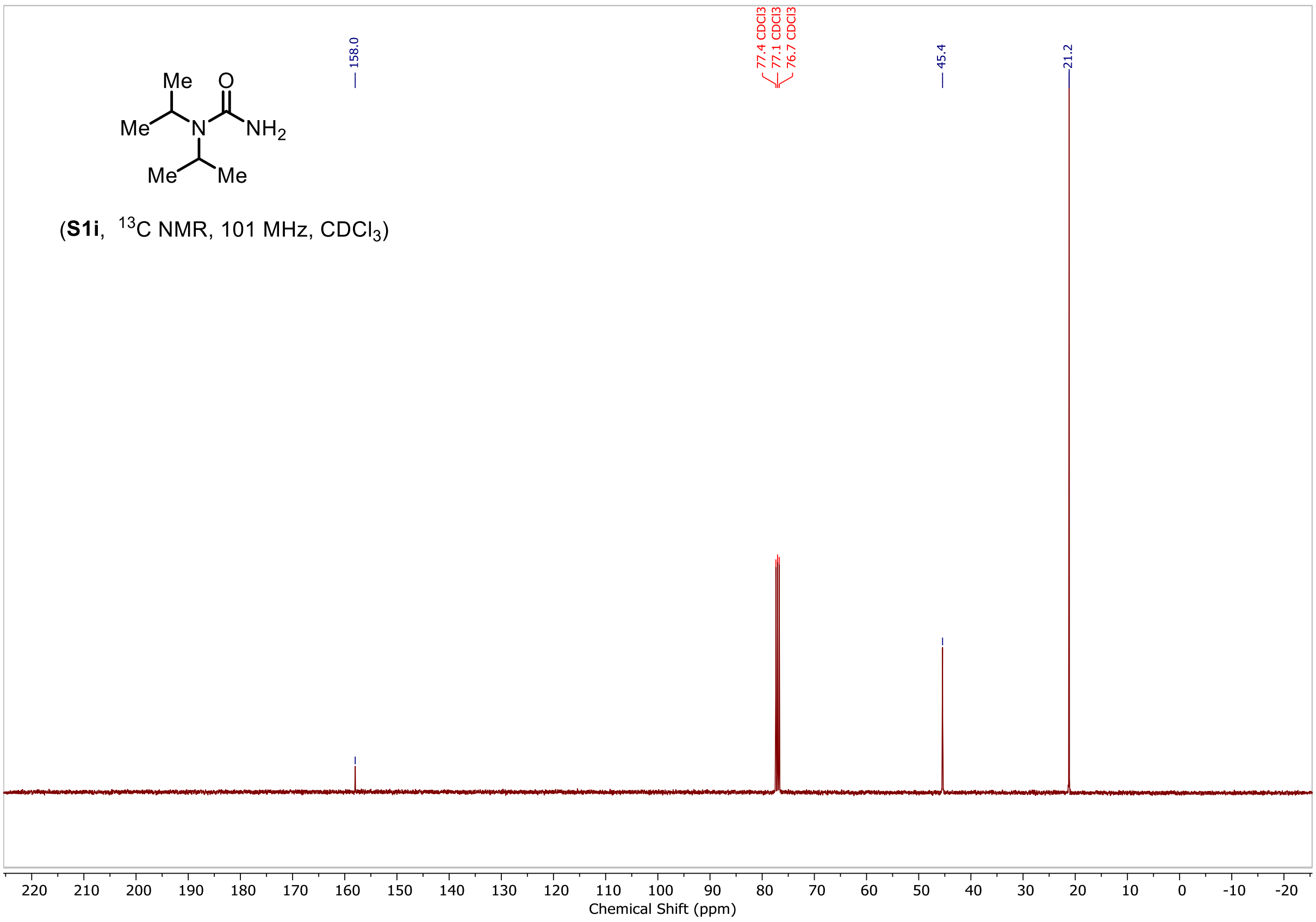

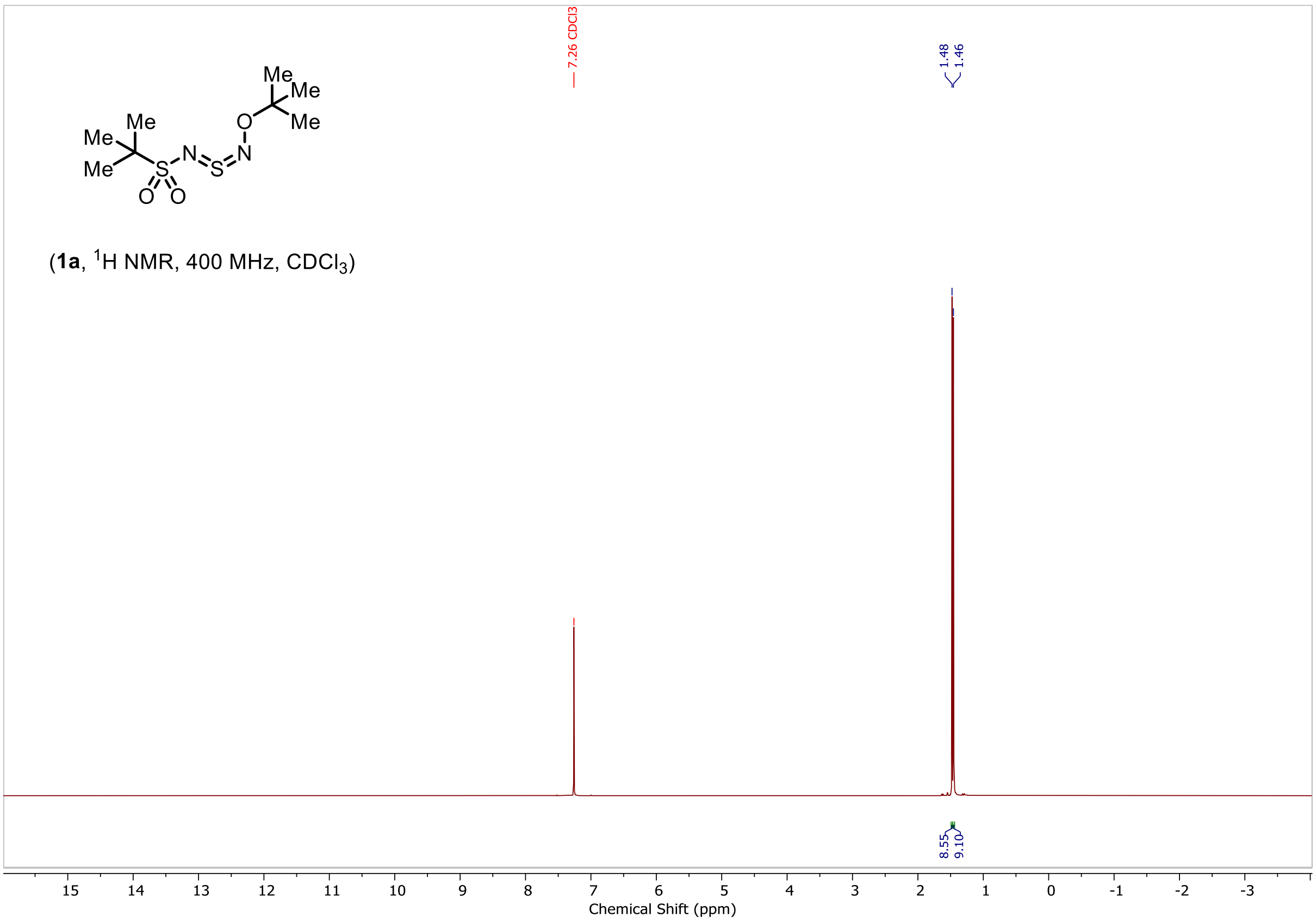

(**1a**, ^{1}H NMR, 400 MHz, $CDCl_3$)

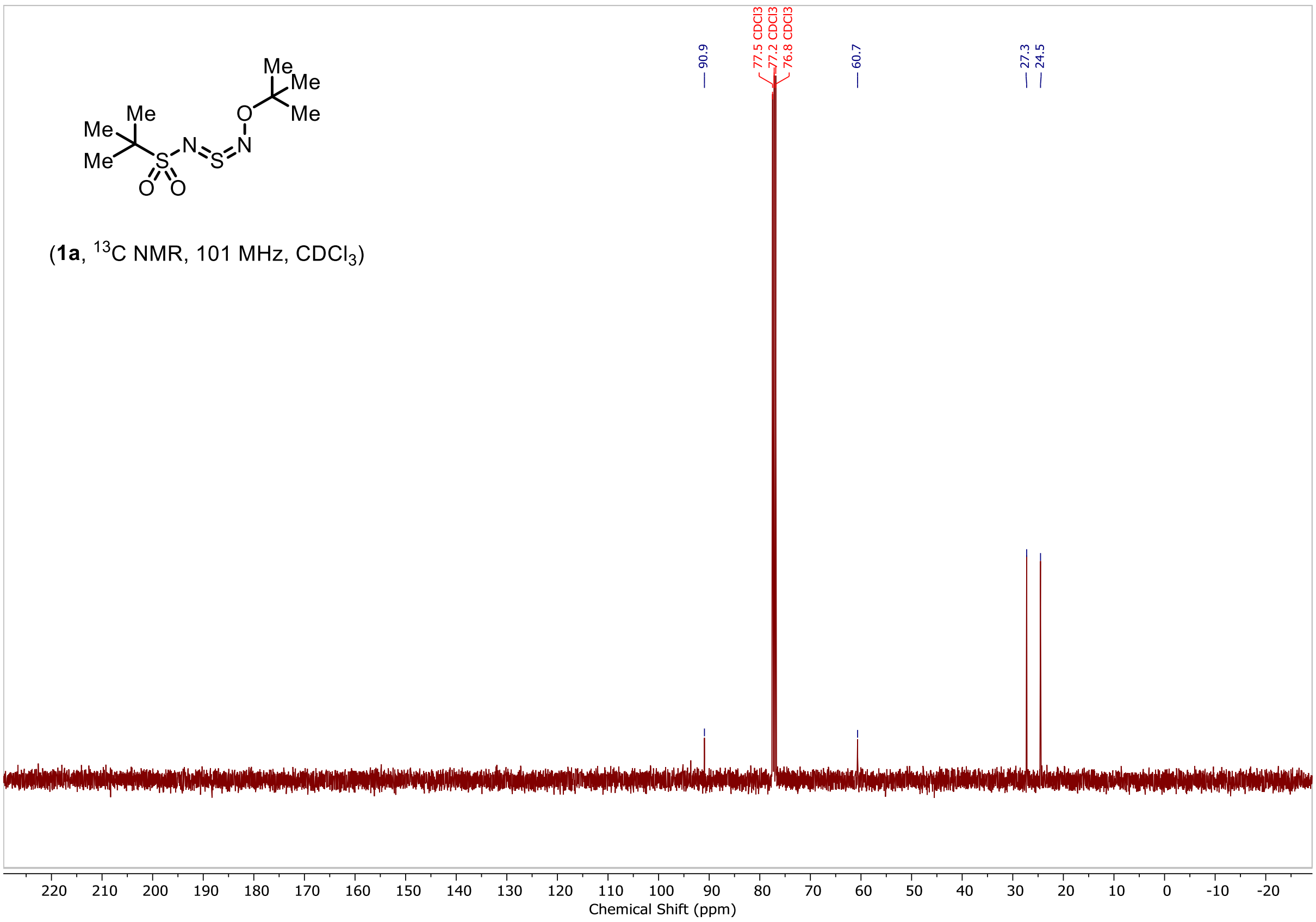

(**1a**, ^{13}C NMR, 101 MHz, $CDCl_3$)

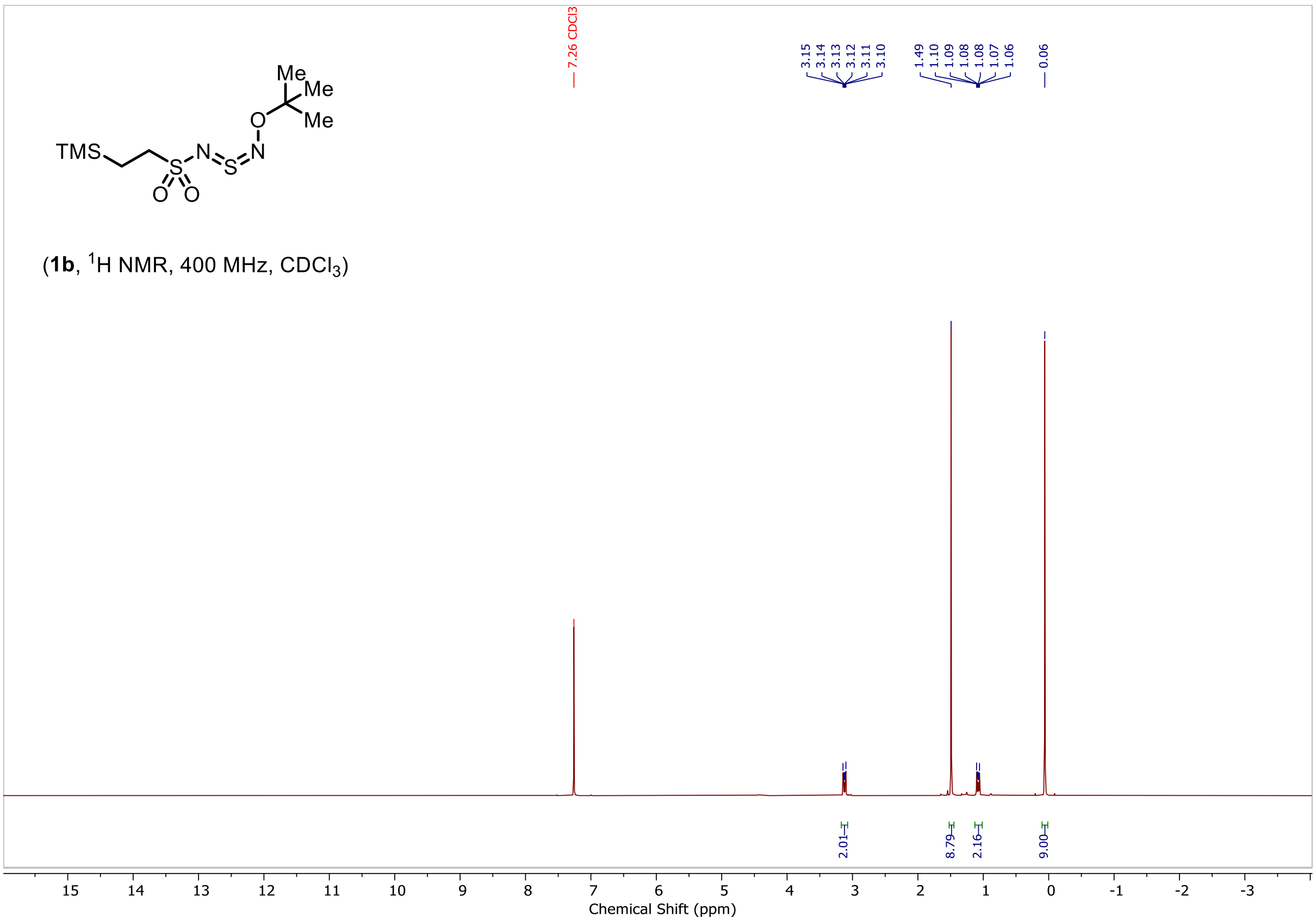

(**1b**, ^{1}H NMR, 400 MHz, $CDCl_3$)

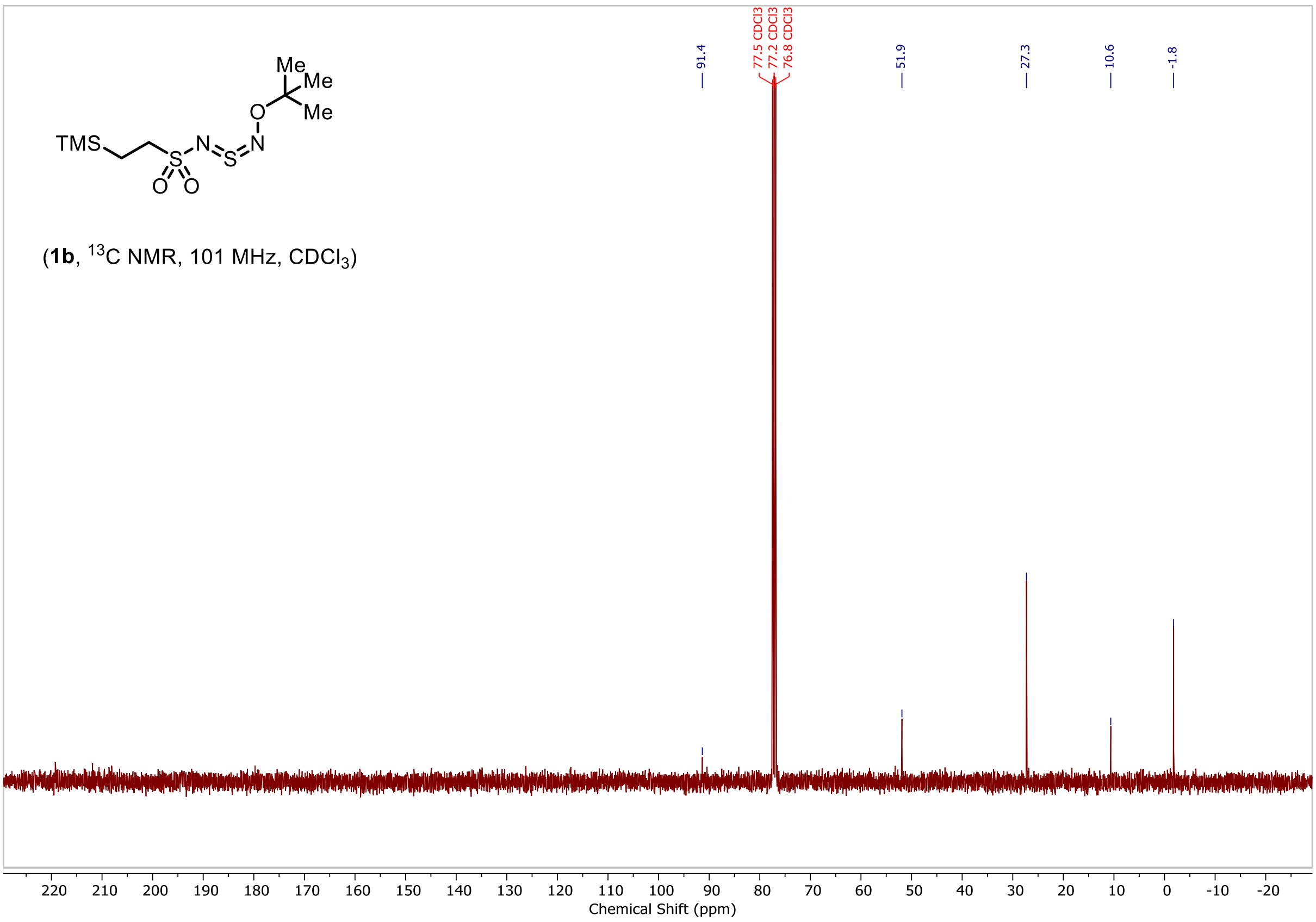

(**1b**, ^{13}C NMR, 101 MHz, $CDCl_3$)

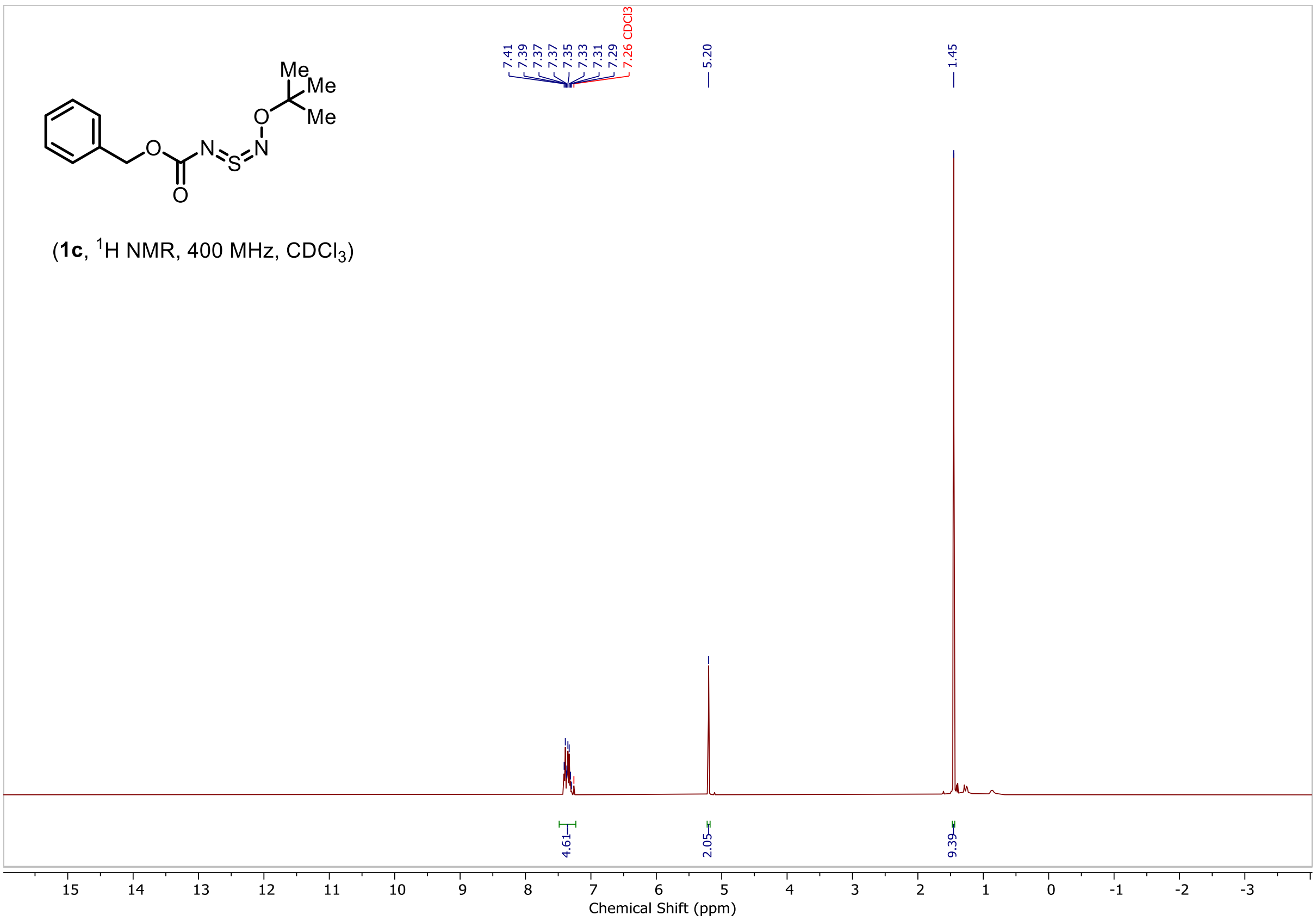

Me
Me
Me
O
O
N
S
N
O
(1c, 1H NMR, 400 MHz, CDCl3)
7.41
7.39
7.37
7.37
7.35
7.33
7.31
7.29
7.26 CDCl3
5.20
1.45
4.61
2.05
9.39
15 14 13 12 11 10 9 8 7 6 5 4 3 2 1 0 -1 -2 -3
Chemical Shift (ppm)

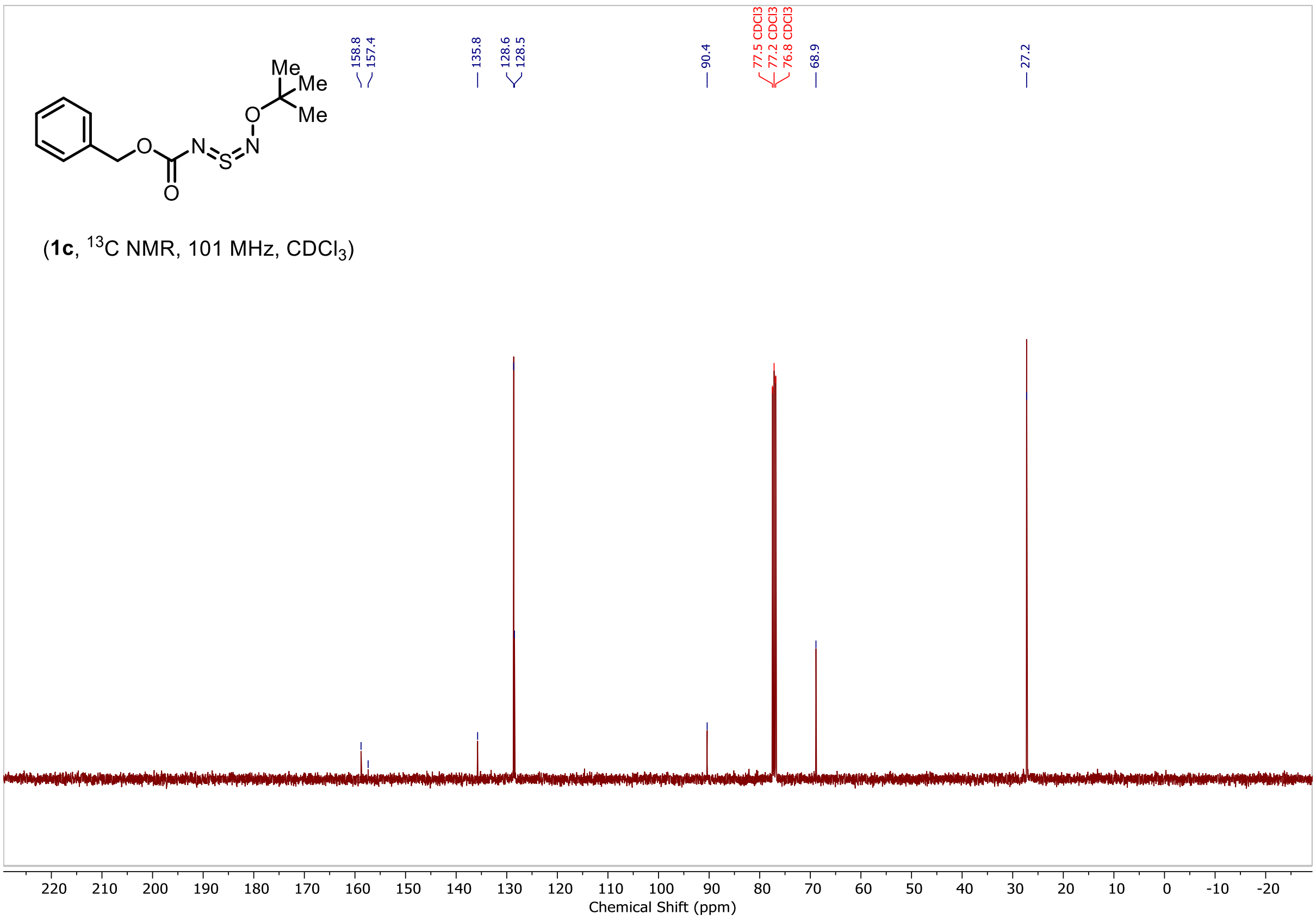

Me
Me
Me
O
O
N
S
N
O
(1c, 13C NMR, 101 MHz, CDCl3)
158.8
157.4
135.8
128.6
128.5
90.4
77.5 CDCl3
77.2 CDCl3
76.8 CDCl3
68.9
27.2
220 210 200 190 180 170 160 150 140 130 120 110 100 90 80 70 60 50 40 30 20 10 0 -10 -20
Chemical Shift (ppm)

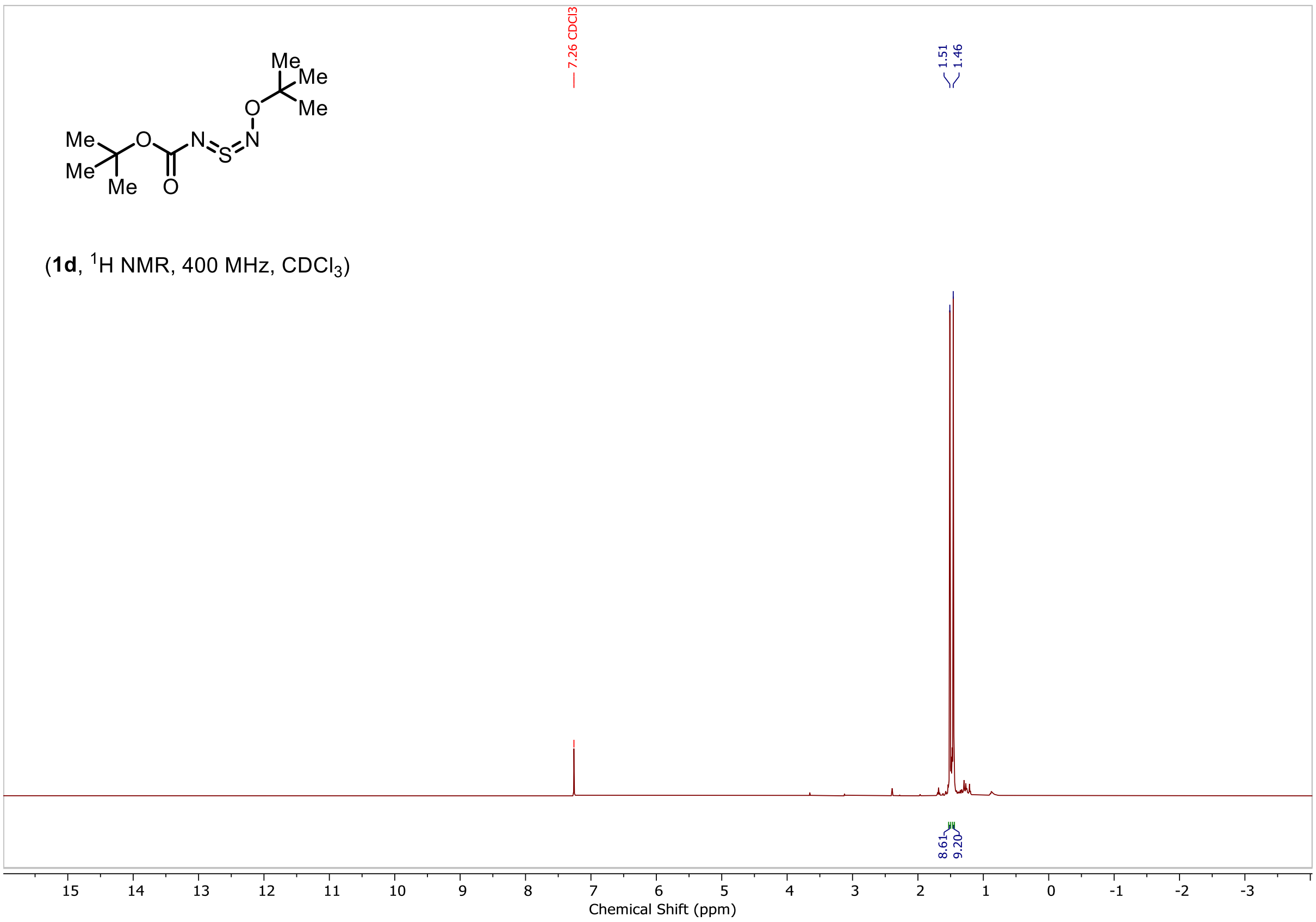
7.26 CDCl3
1.51
1.46
(1d, 1H NMR, 400 MHz, CDCl3)
8.61
9.20
Chemical Shift (ppm)

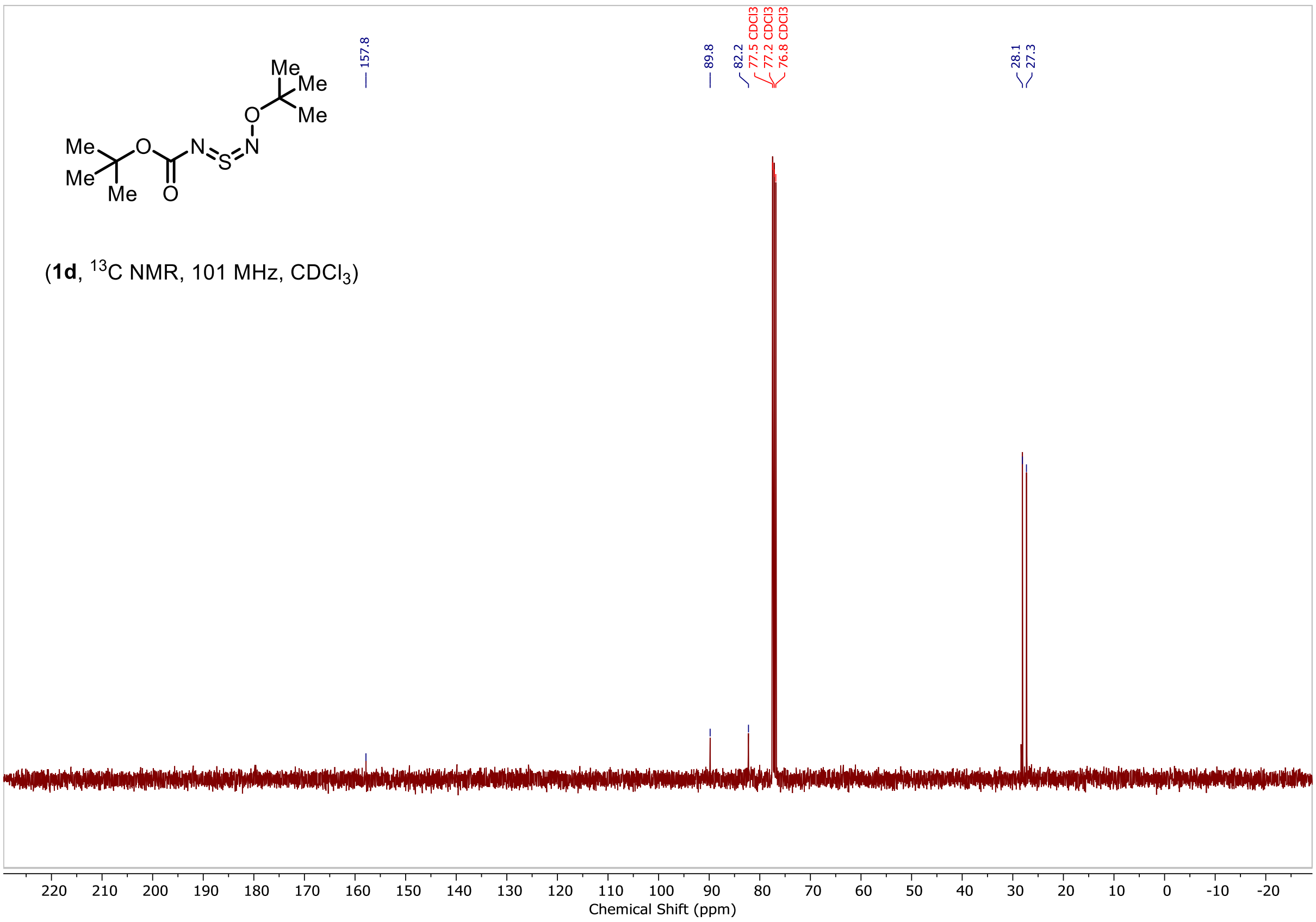
157.8
89.8
82.2
77.5 CDCl3
77.2 CDCl3
76.8 CDCl3
28.1
27.3
(1d, 13C NMR, 101 MHz, CDCl3)
Chemical Shift (ppm)

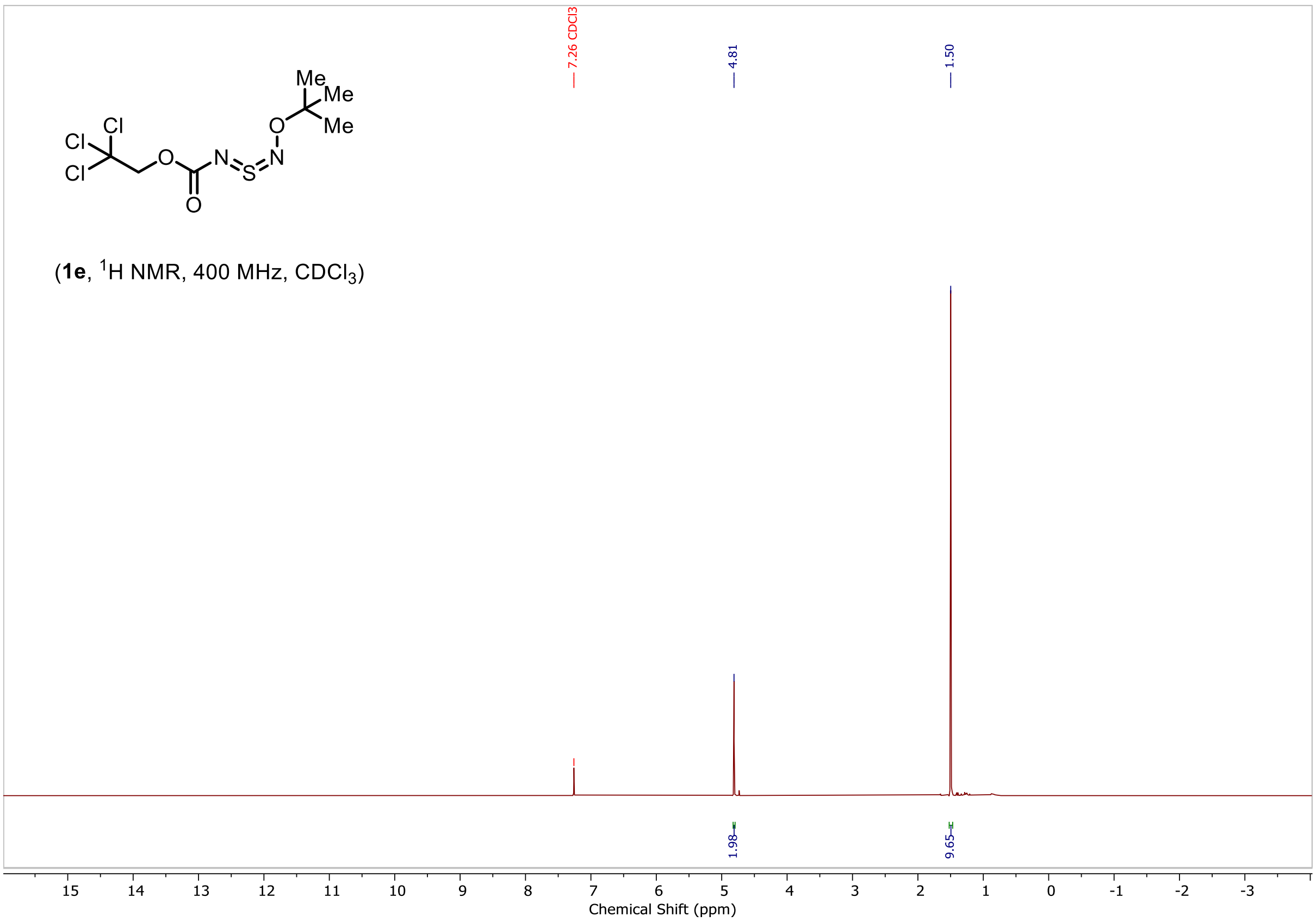

Me
Me
Me
Cl
Cl
Cl
O
N
S
N
O
O
(1e, ^{1}H NMR, 400 MHz, $CDCl_3$)
7.26 CDCl3
4.81
1.50
1.98
9.65
15 14 13 12 11 10 9 8 7 6 5 4 3 2 1 0 -1 -2 -3
Chemical Shift (ppm)

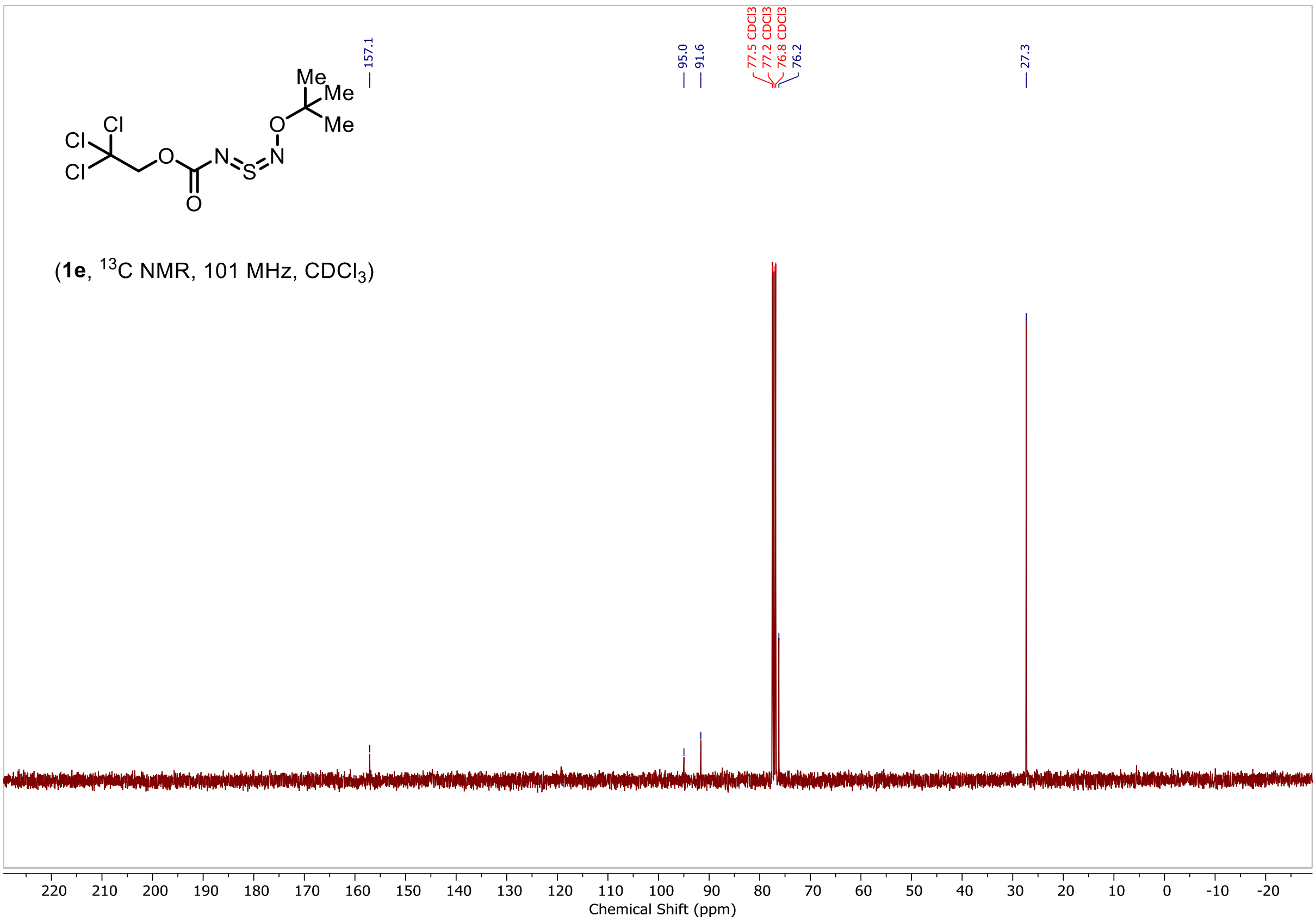

Me
Me
Me
Cl
Cl
Cl
O
N
S
N
O
O
(1e, ^{13}C NMR, 101 MHz, $CDCl_3$)
157.1
95.0
91.6
77.5 CDCl3
77.2 CDCl3
76.8 CDCl3
76.2
27.3
220 210 200 190 180 170 160 150 140 130 120 110 100 90 80 70 60 50 40 30 20 10 0 -10 -20
Chemical Shift (ppm)

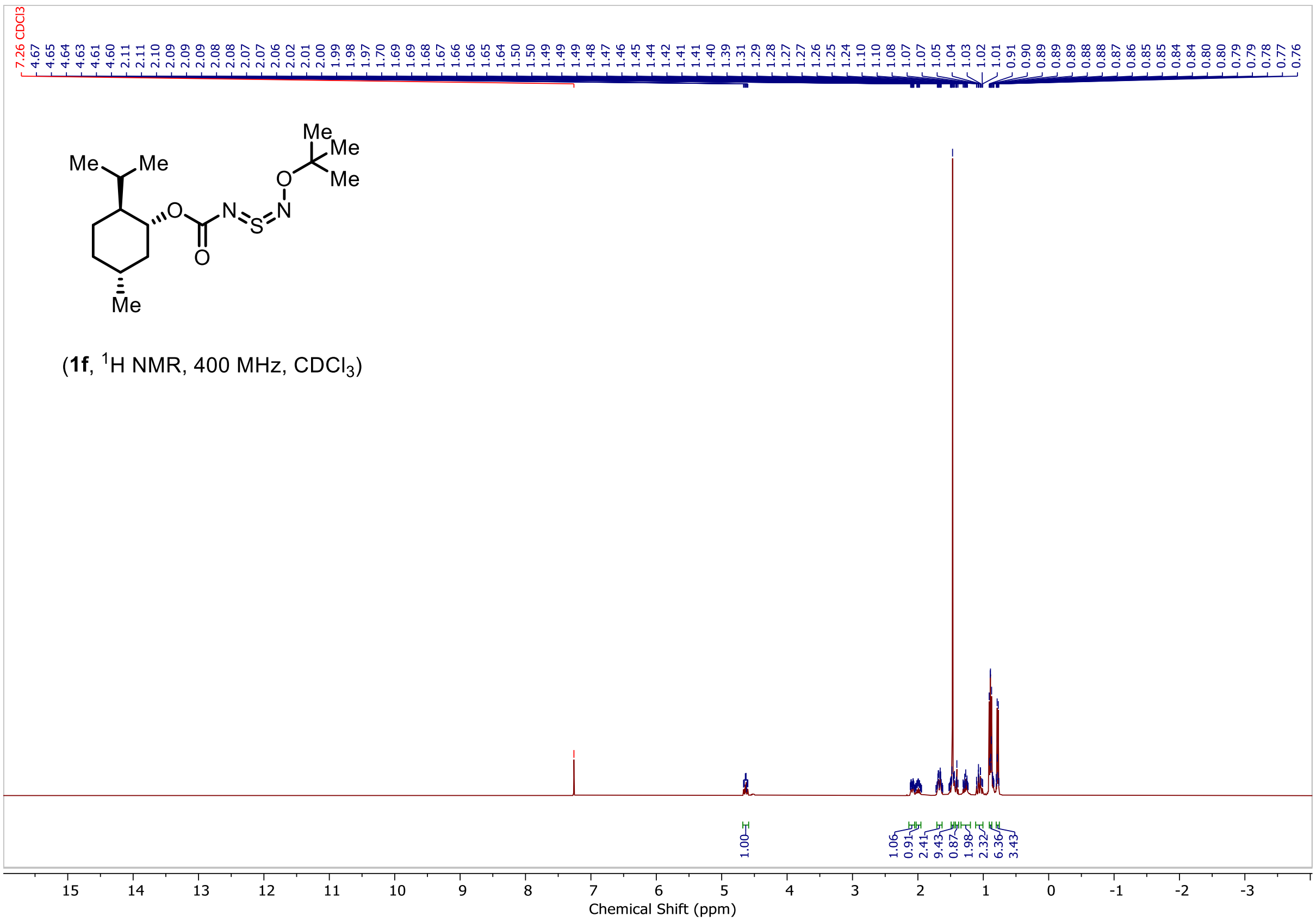
Me
Me
Me
Me
Me
O
N
S
N
O
O
Me
(1f, 1H NMR, 400 MHz, CDCl3)
7.26 CDCl3
1.00
1.06
0.91
2.41
9.43
0.87
1.98
2.32
6.36
3.43
15 14 13 12 11 10 9 8 7 6 5 4 3 2 1 0 -1 -2 -3
Chemical Shift (ppm)

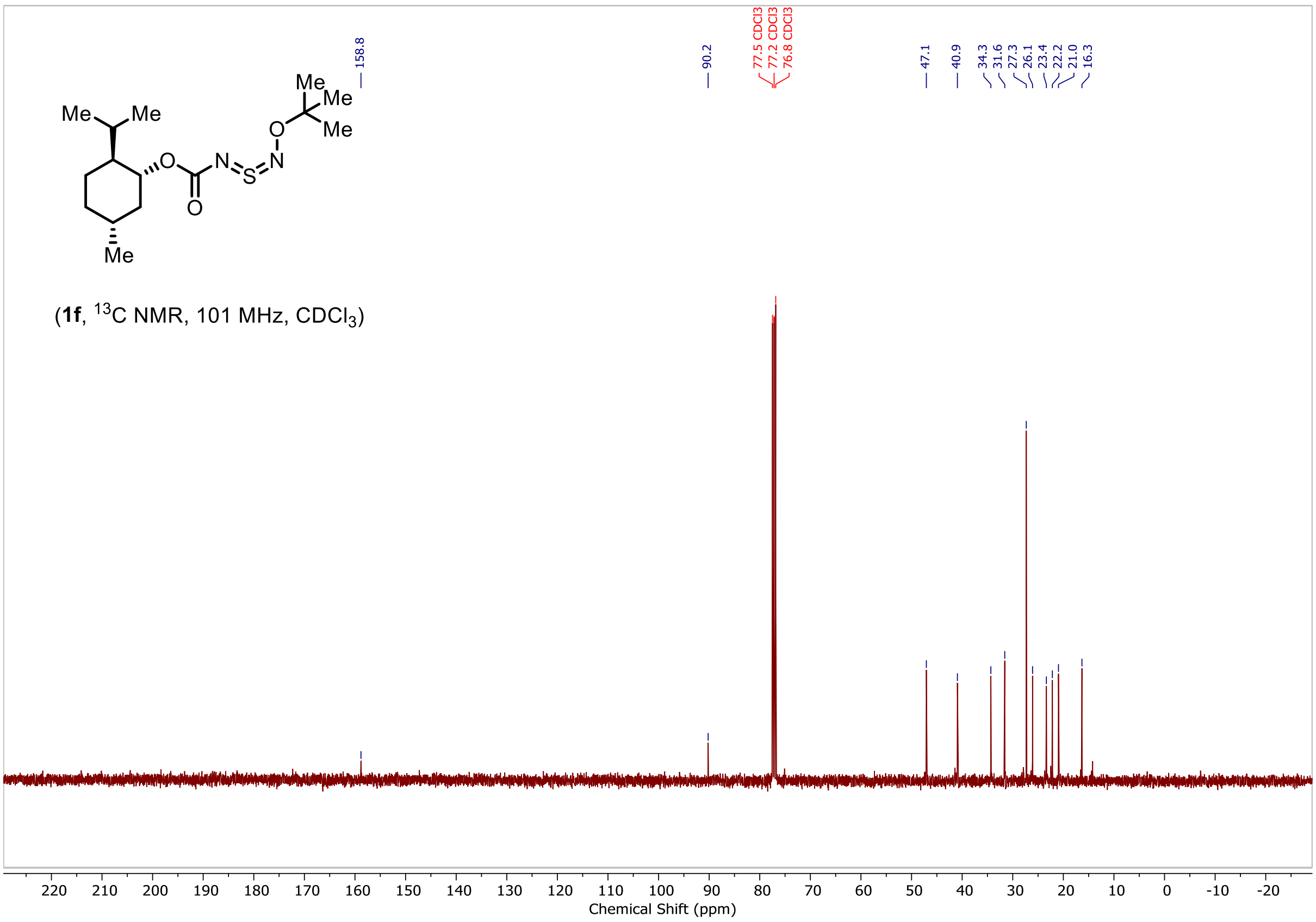
158.8
90.2
77.5 CDCl3
77.2 CDCl3
76.8 CDCl3
47.1
40.9
34.3
31.6
27.3
26.1
23.4
22.2
21.0
16.3
(1f, 13C NMR, 101 MHz, CDCl3)
220 210 200 190 180 170 160 150 140 130 120 110 100 90 80 70 60 50 40 30 20 10 0 -10 -20
Chemical Shift (ppm)

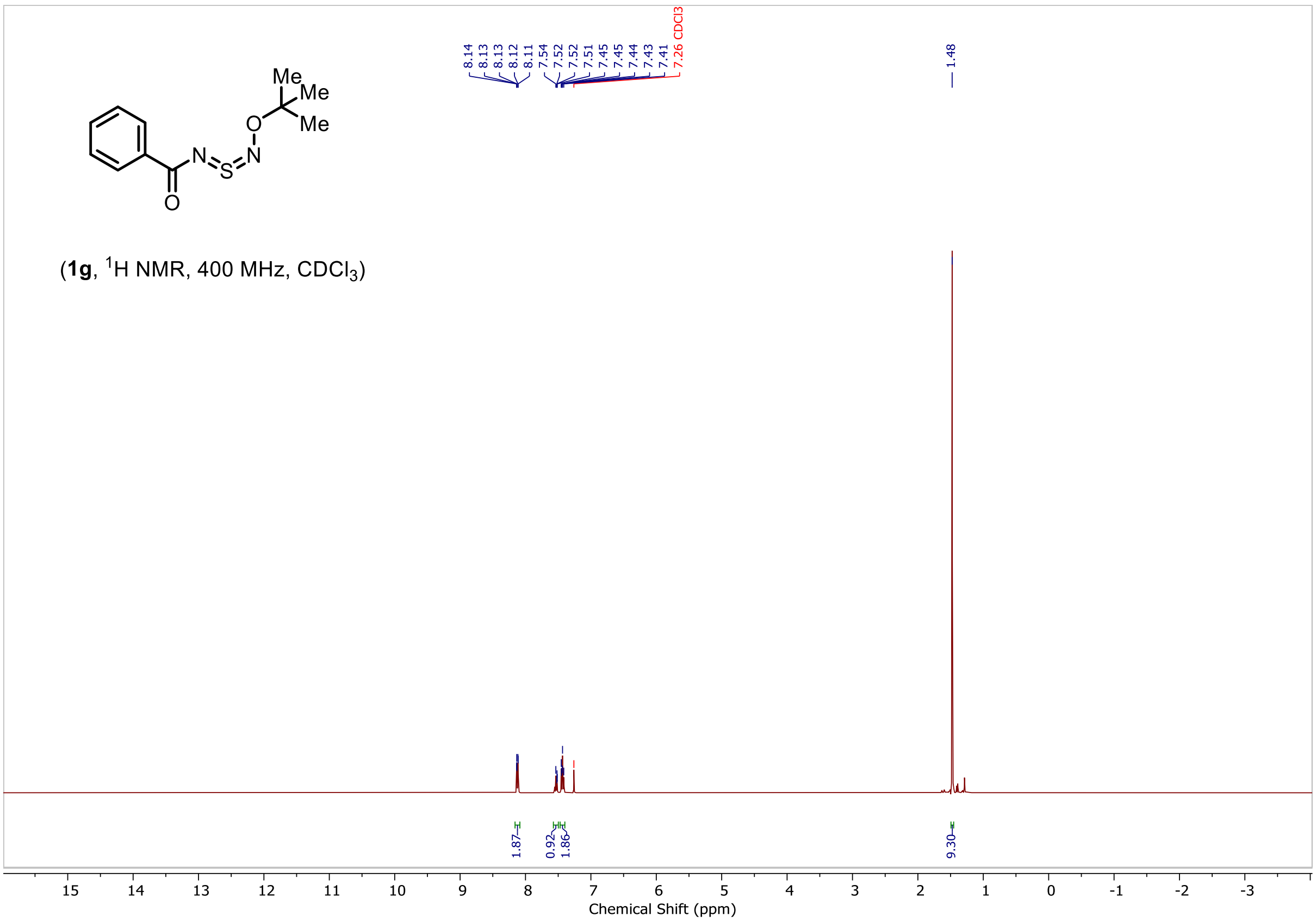

(**1g**, ^{1}H NMR, 400 MHz, $CDCl_3$)

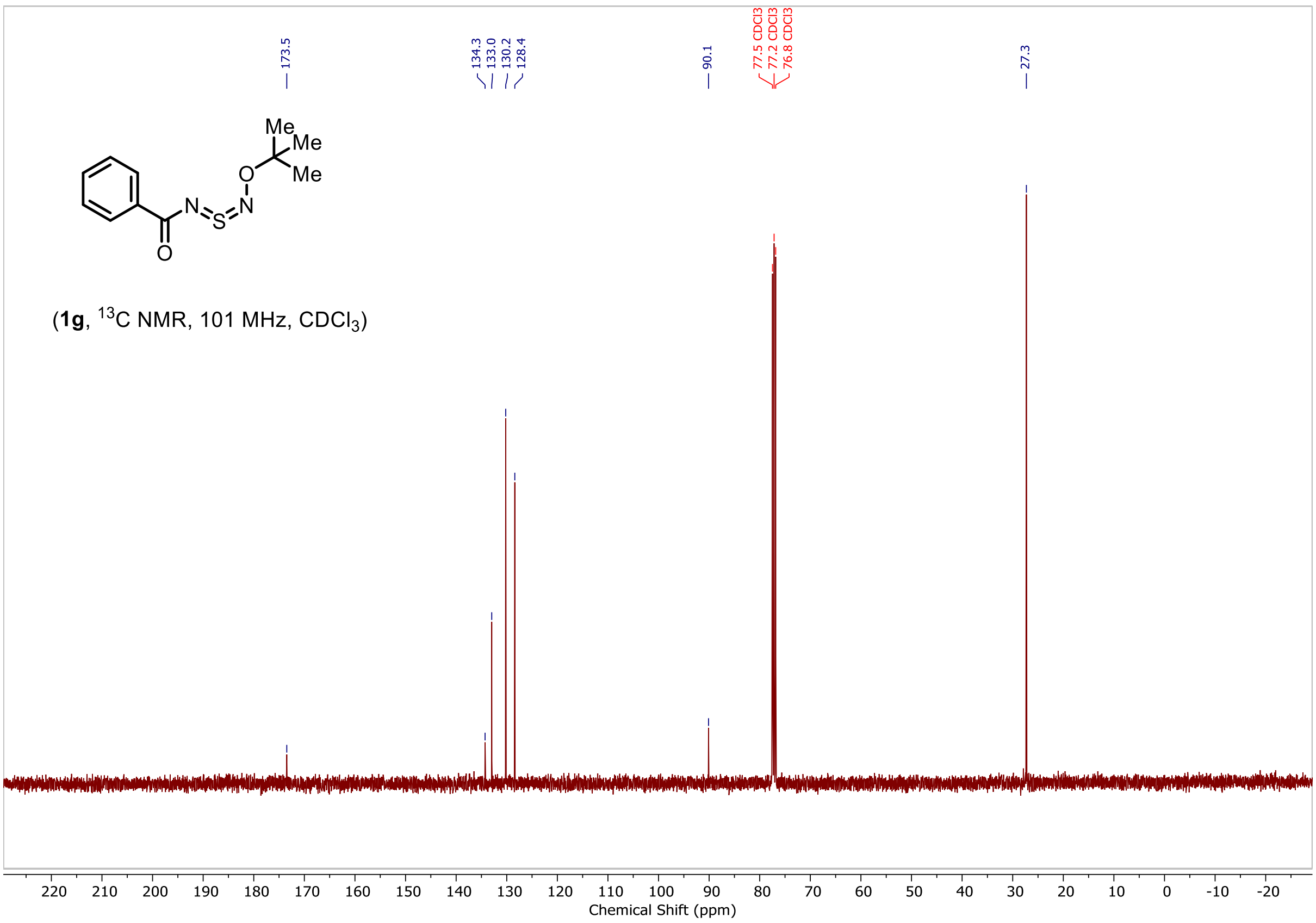

(**1g**, ^{13}C NMR, 101 MHz, $CDCl_3$)

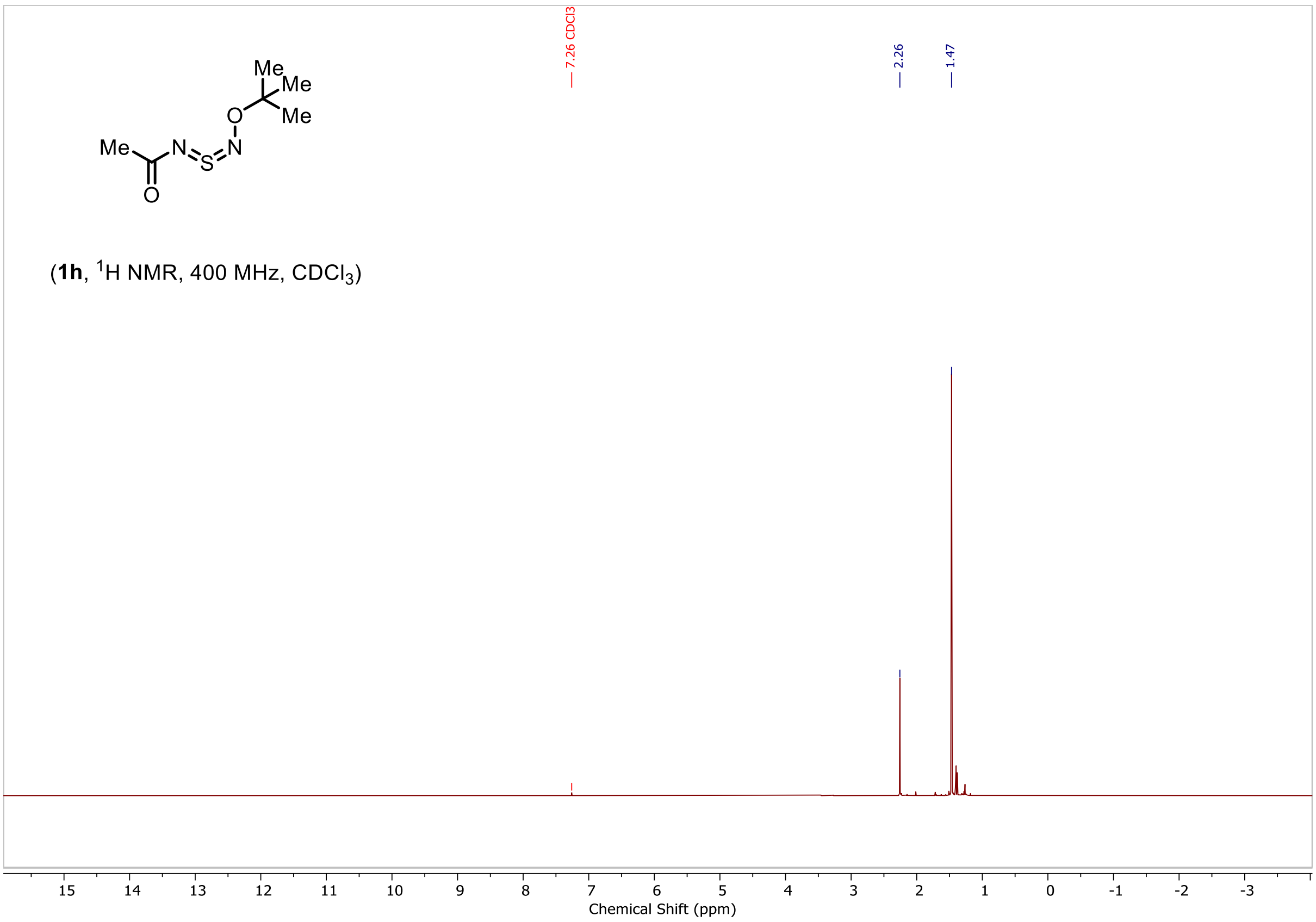

7.26 CDCl3
2.26
1.47
Me
Me
Me
O
Me
N
S
N
O
(1h, 1H NMR, 400 MHz, CDCl3)
15 14 13 12 11 10 9 8 7 6 5 4 3 2 1 0 -1 -2 -3
Chemical Shift (ppm)

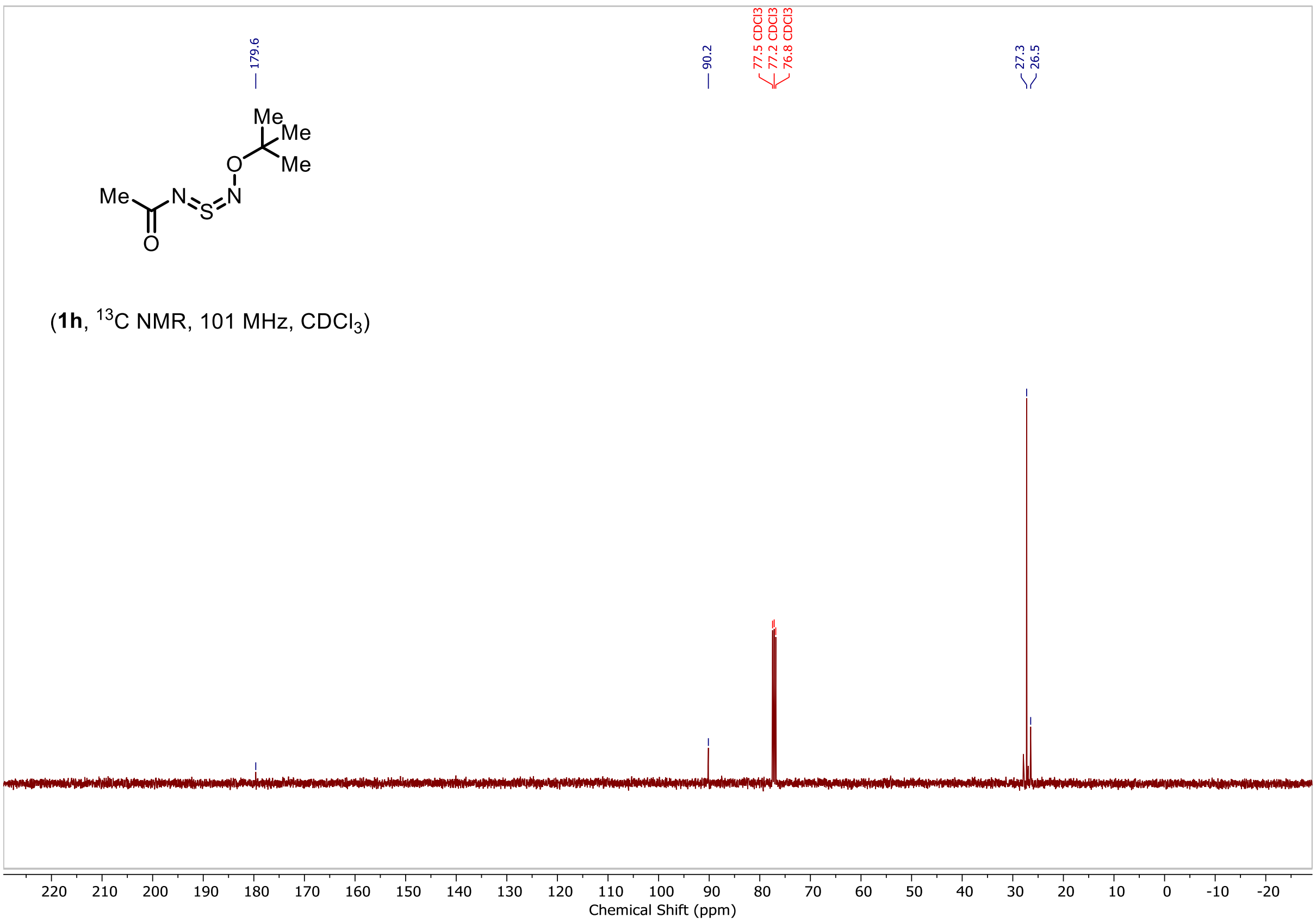

179.6
90.2
77.5 CDCl3
77.2 CDCl3
76.8 CDCl3
27.3
26.5
Me
Me
Me
O
Me
N
S
N
O
(1h, 13C NMR, 101 MHz, CDCl3)
220 210 200 190 180 170 160 150 140 130 120 110 100 90 80 70 60 50 40 30 20 10 0 -10 -20
Chemical Shift (ppm)

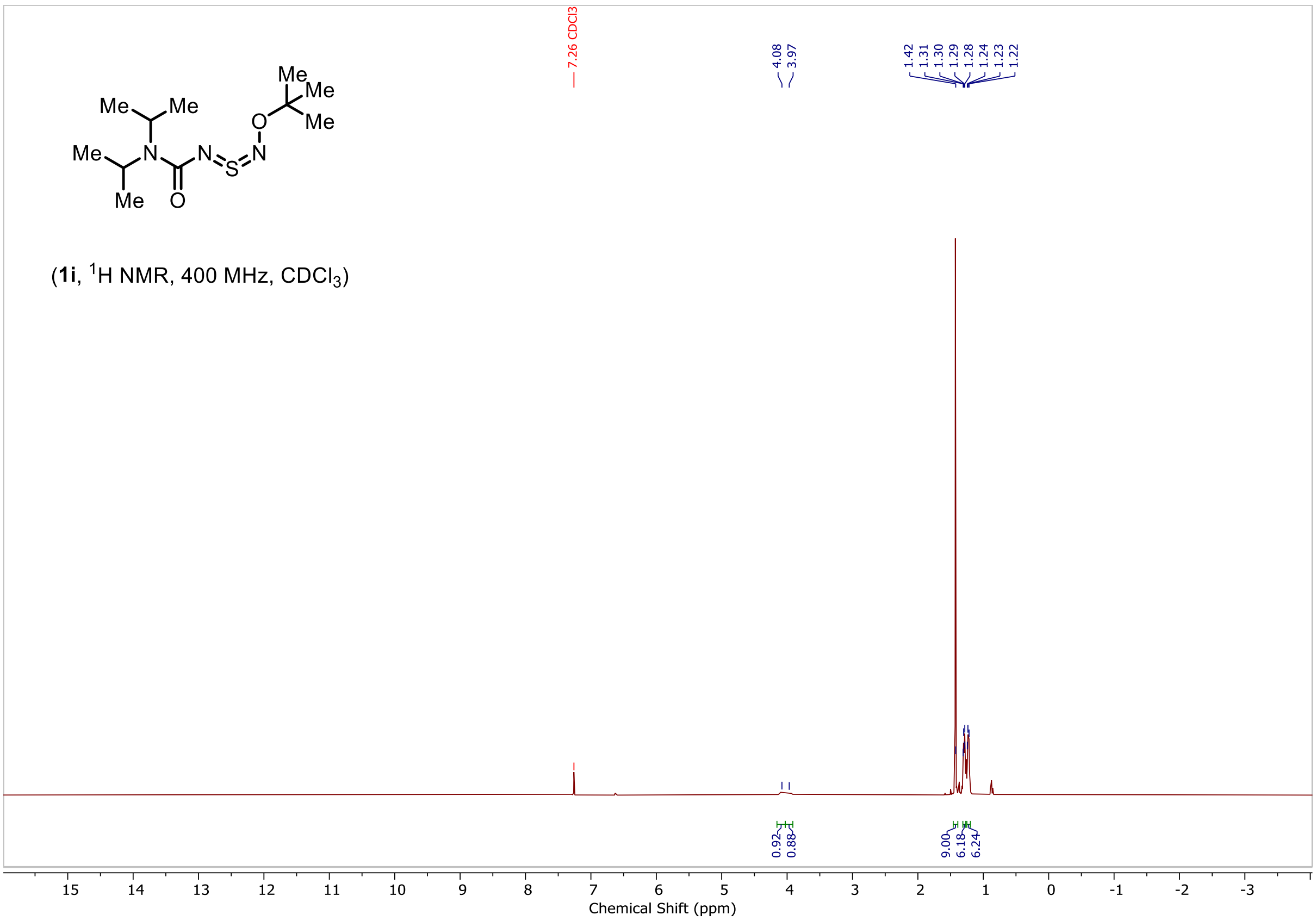
(1i, 1H NMR, 400 MHz, CDCl3)
7.26 CDCl3
4.08
3.97
1.42
1.31
1.30
1.29
1.28
1.24
1.23
1.22
0.92
0.88
9.00
6.18
6.24
Chemical Shift (ppm)

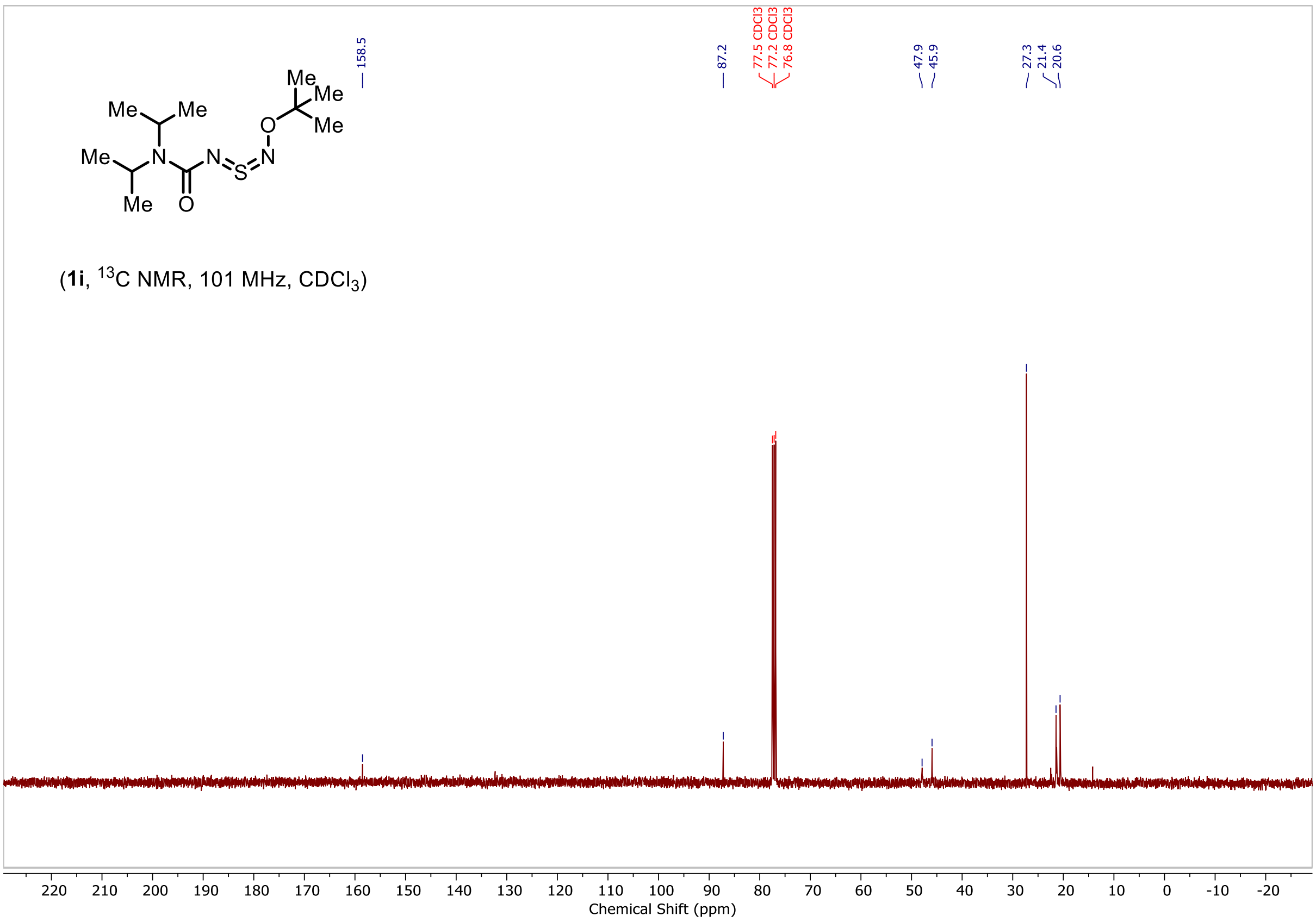
(1i, 13C NMR, 101 MHz, CDCl3)
158.5
87.2
77.5 CDCl3
77.2 CDCl3
76.8 CDCl3
47.9
45.9
27.3
21.4
20.6
Chemical Shift (ppm)

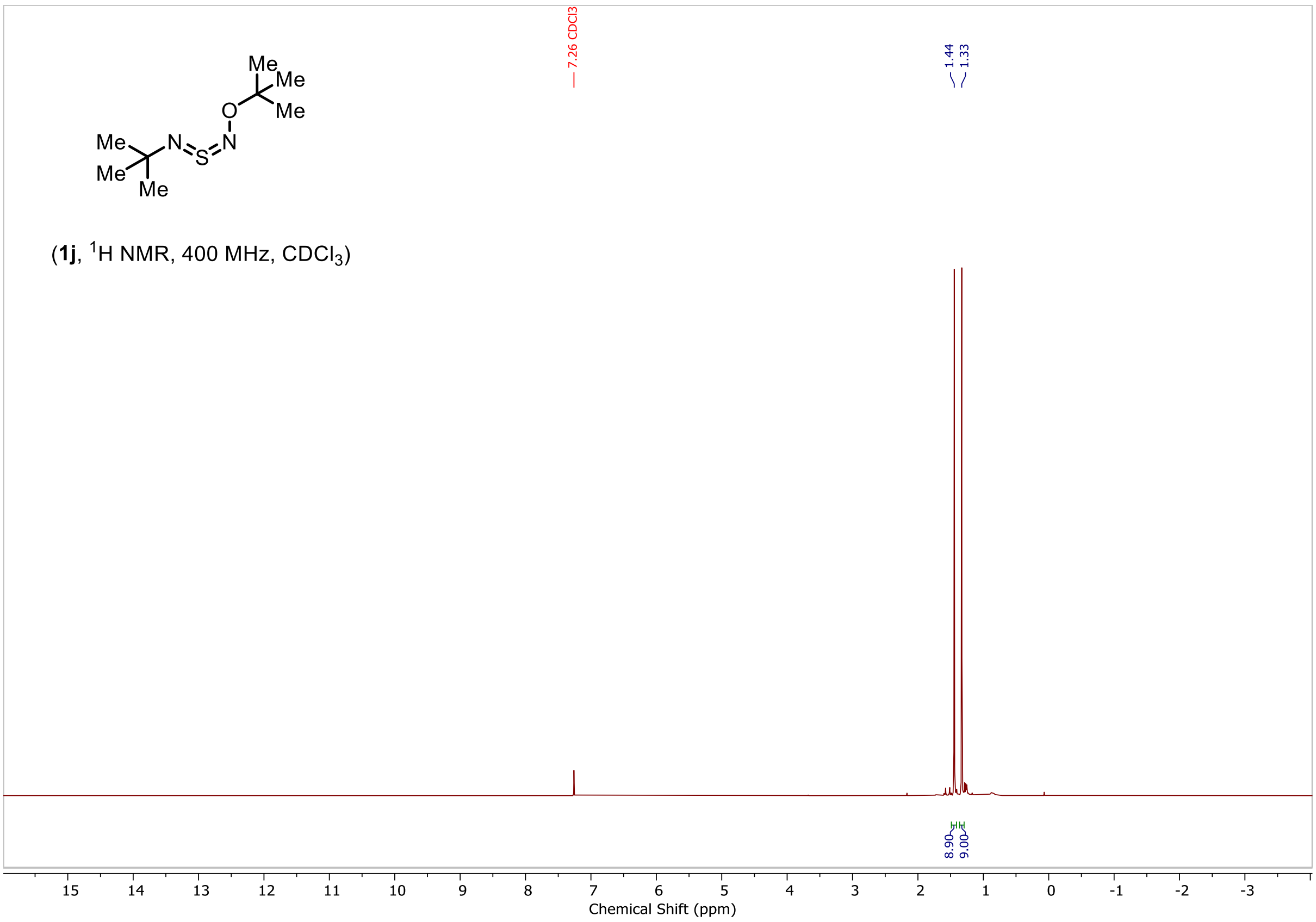
7.26 CDCl3
1.44
1.33
(1j, 1H NMR, 400 MHz, CDCl3)
Me
Me
Me
O
N
S
N
Me
Me
Me
8.90
9.00
Chemical Shift (ppm)

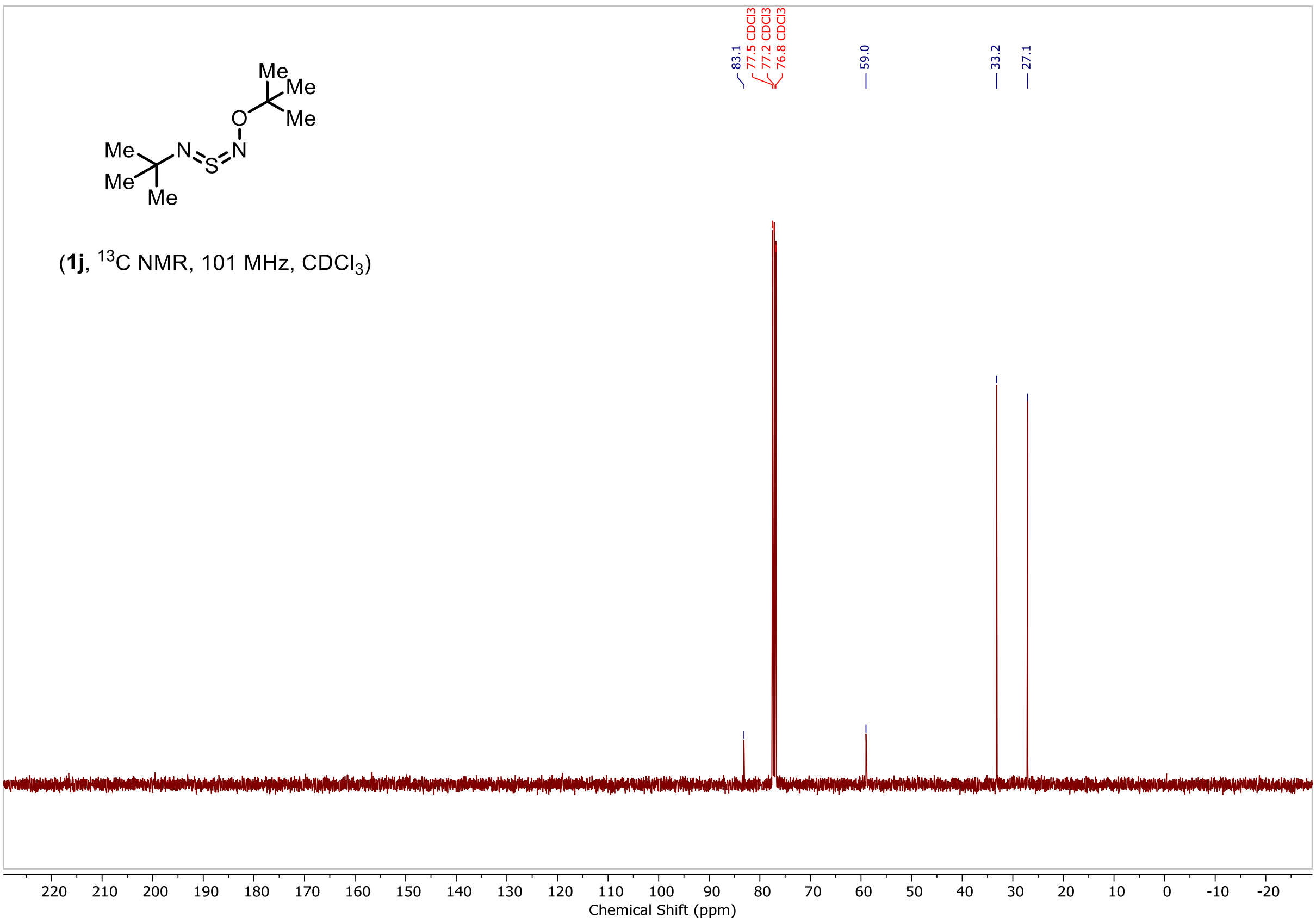
83.1
77.5 CDCl3
77.2 CDCl3
76.8 CDCl3
59.0
33.2
27.1
(1j, 13C NMR, 101 MHz, CDCl3)
Chemical Shift (ppm)

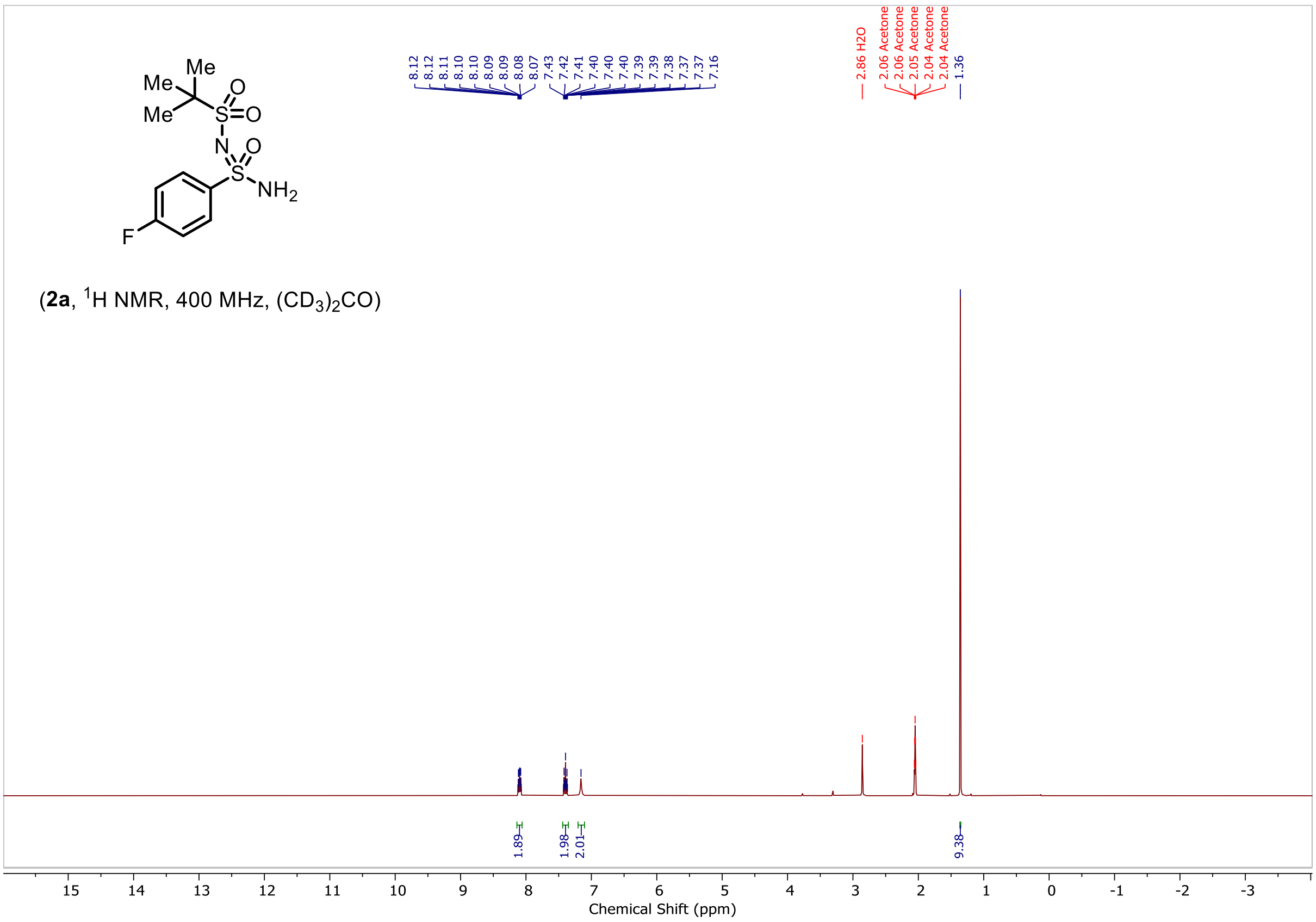

(**2a**, ^{1}H NMR, 400 MHz, $(CD_3)_2CO$)

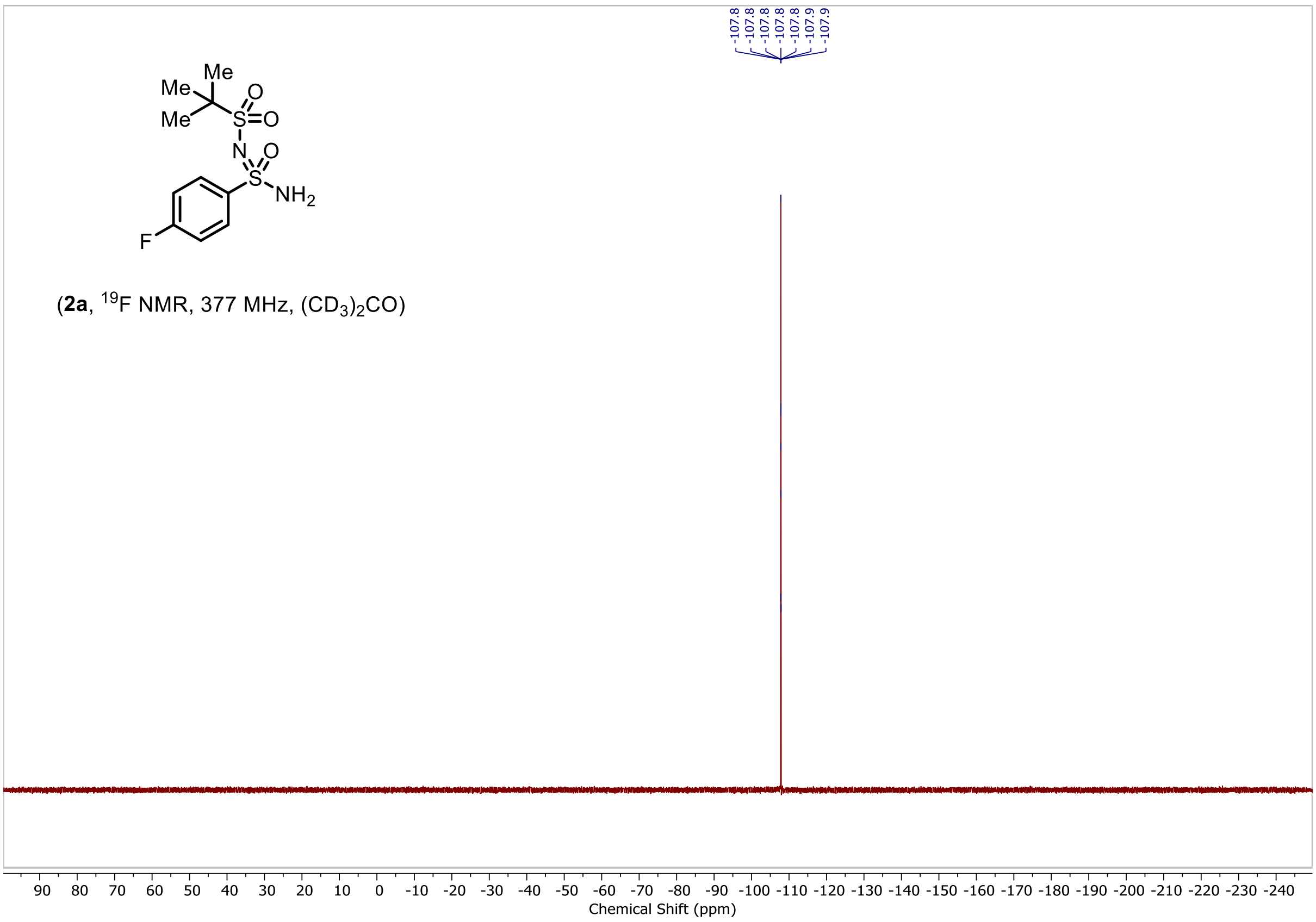

(**2a**, ^{19}F NMR, 377 MHz, $(CD_3)_2CO$)

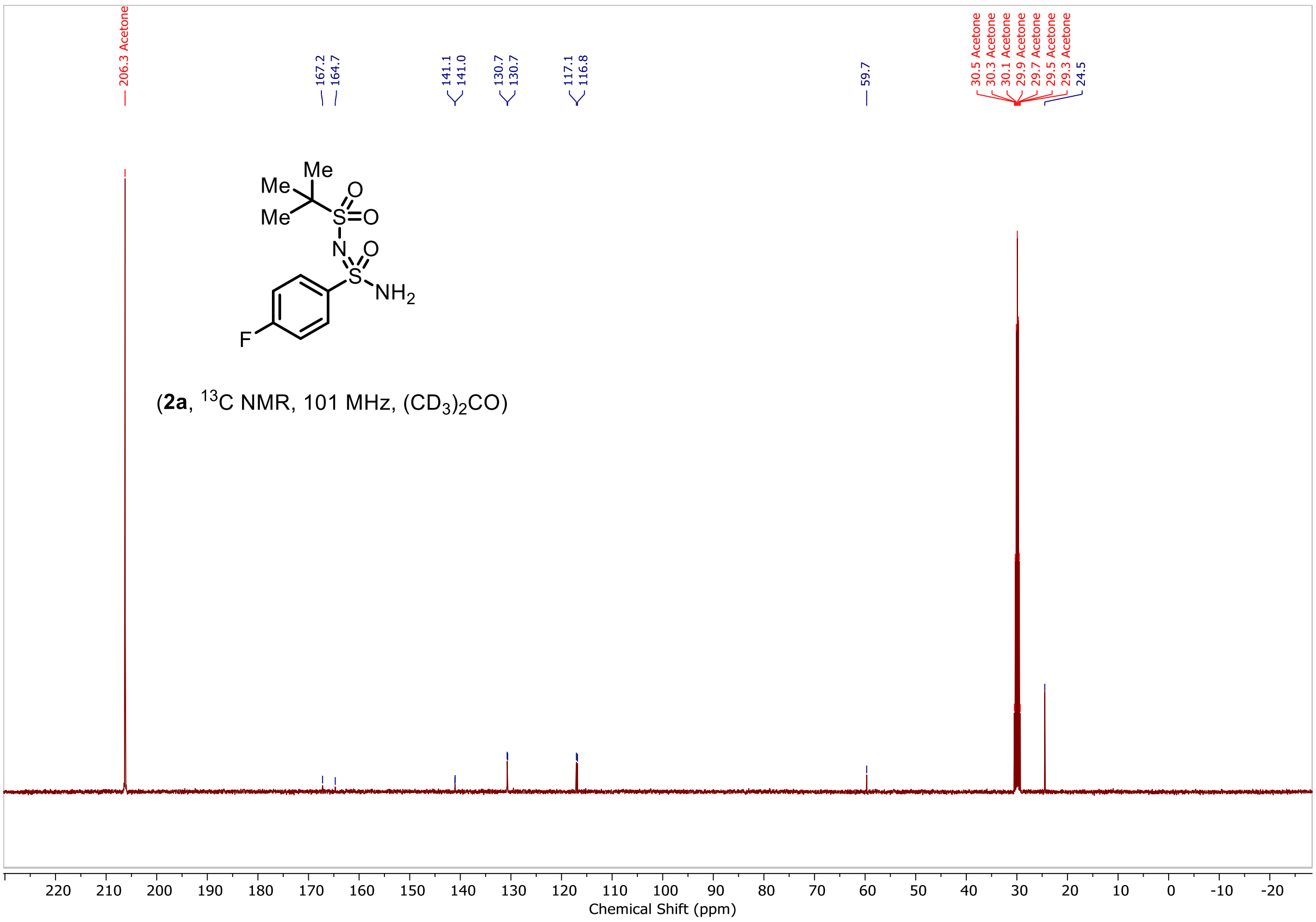

(**2a**, ^{13}C NMR, 101 MHz, $(CD_3)_2CO$)

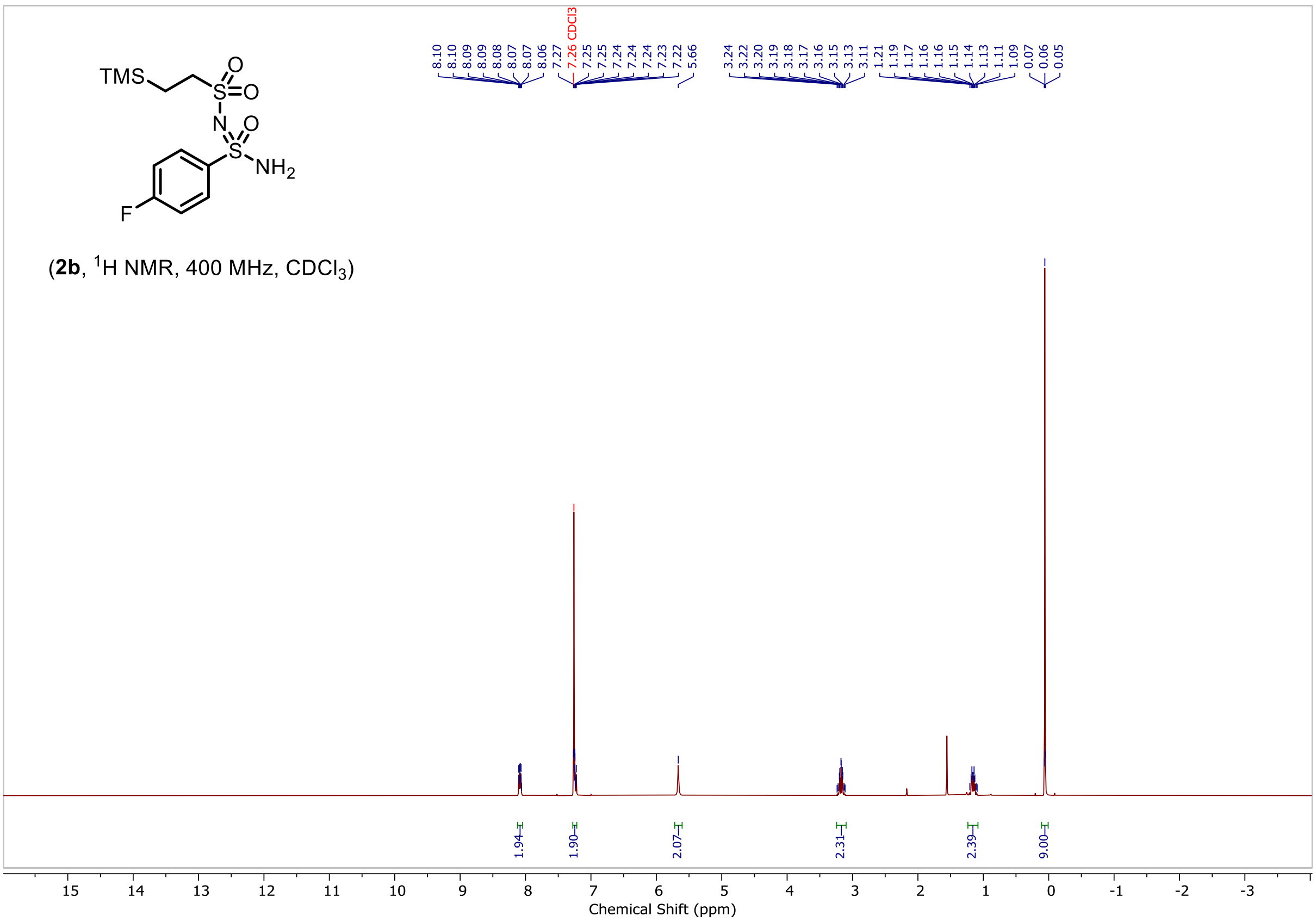

(**2b**, ^{1}H NMR, 400 MHz, $CDCl_3$)

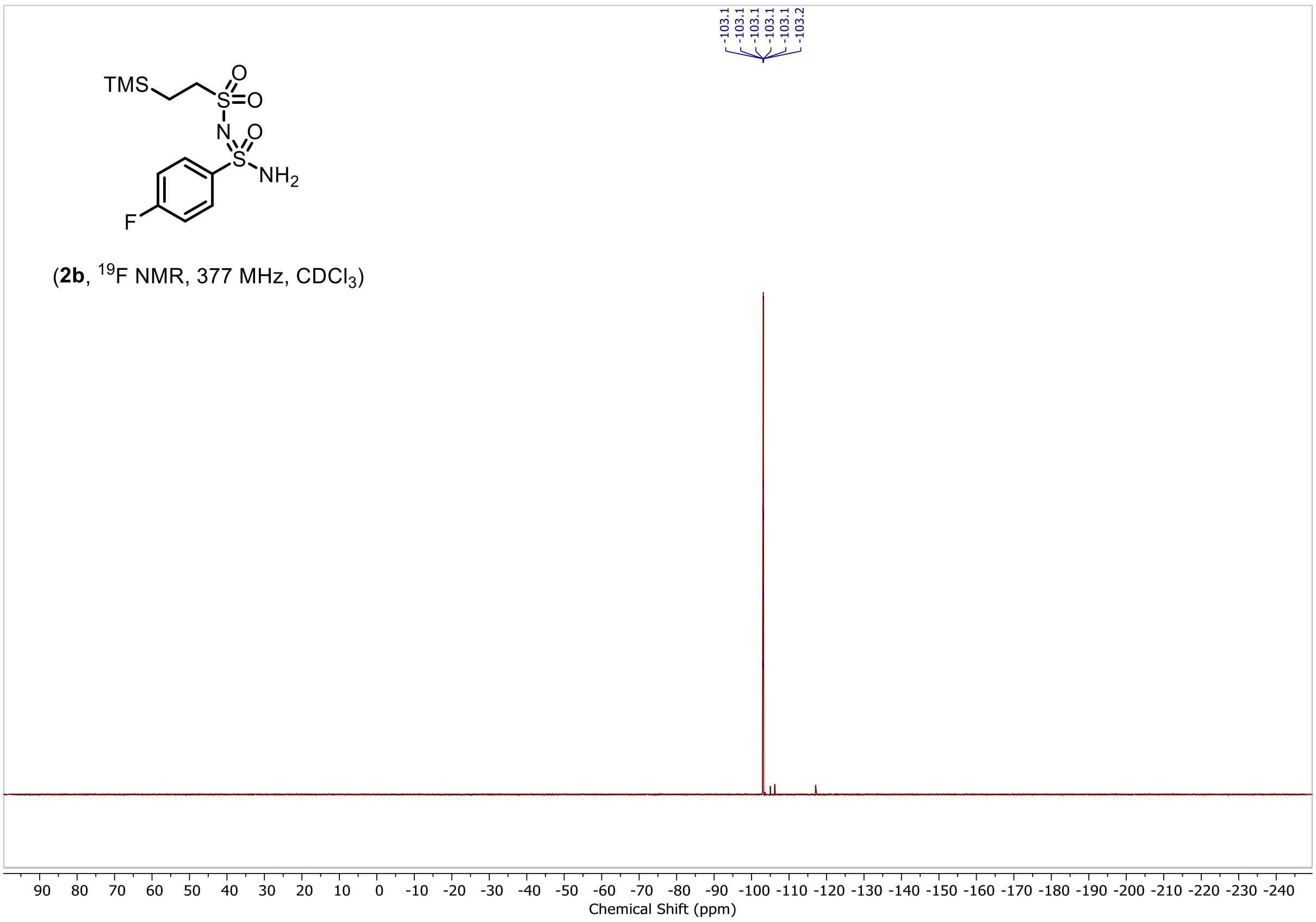

(**2b**, ^{19}F NMR, 377 MHz, $CDCl_3$)

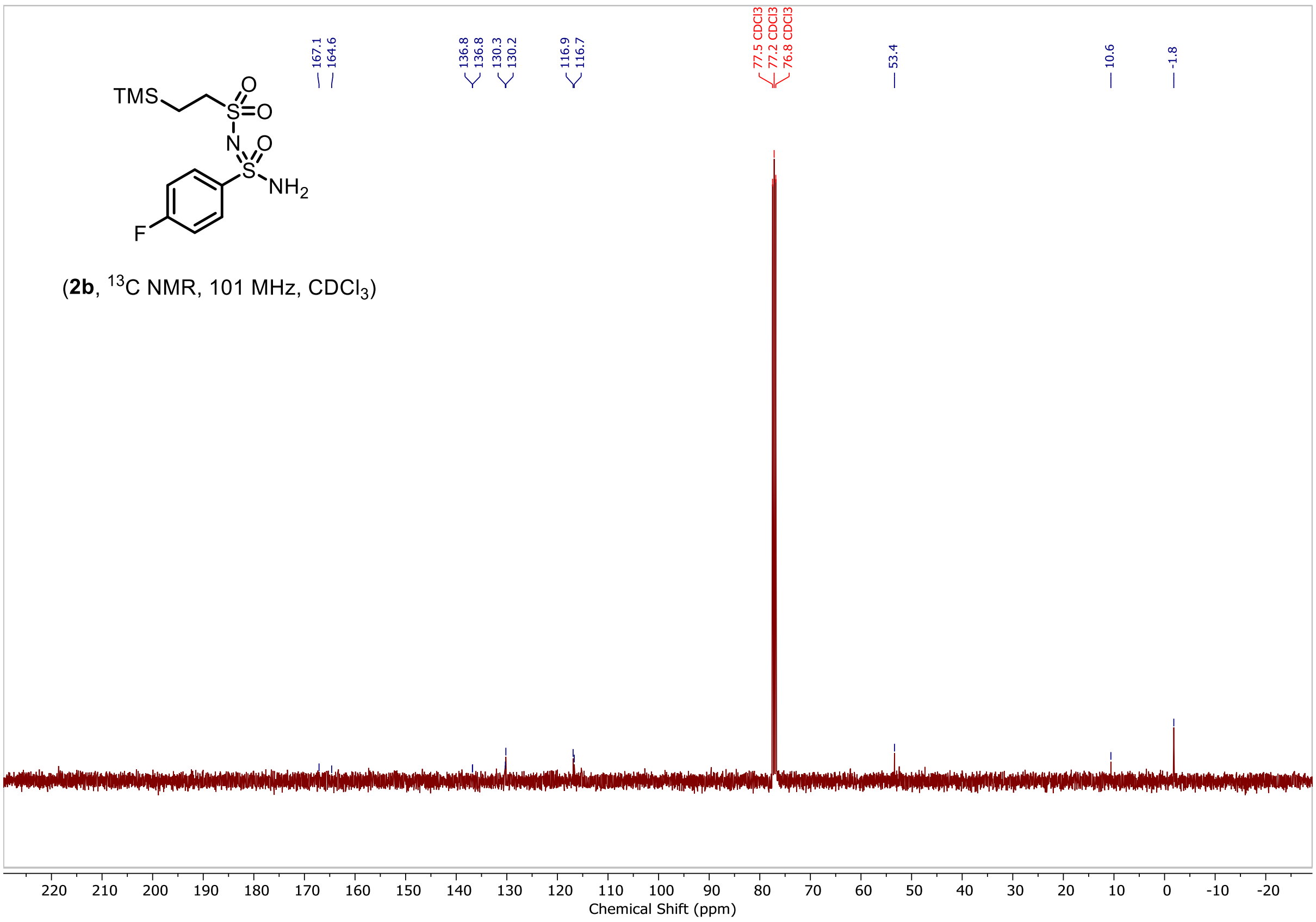

(**2b**, ^{13}C NMR, 101 MHz, $CDCl_3$)

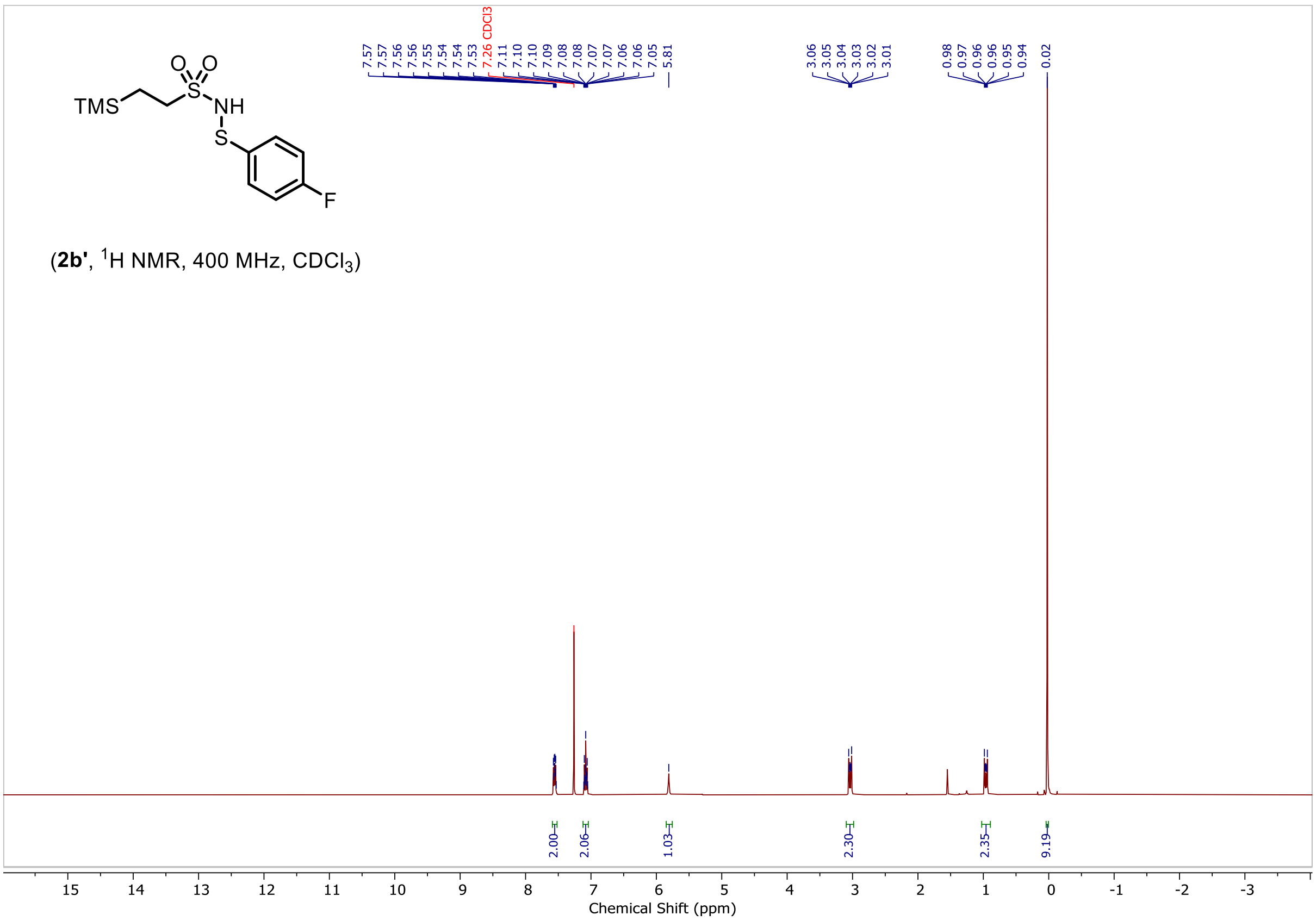
TMS
NH
S
F
(2b', 1H NMR, 400 MHz, CDCl3)
7.57 7.57 7.56 7.56 7.55 7.54 7.54 7.53 7.26 CDCl3 7.11 7.10 7.10 7.09 7.08 7.08 7.07 7.07 7.06 7.06 7.05 5.81
3.06 3.05 3.04 3.03 3.02 3.01
0.98 0.97 0.96 0.96 0.95 0.94 0.02
2.00 2.06 1.03 2.30 2.35 9.19
15 14 13 12 11 10 9 8 7 6 5 4 3 2 1 0 -1 -2 -3
Chemical Shift (ppm)

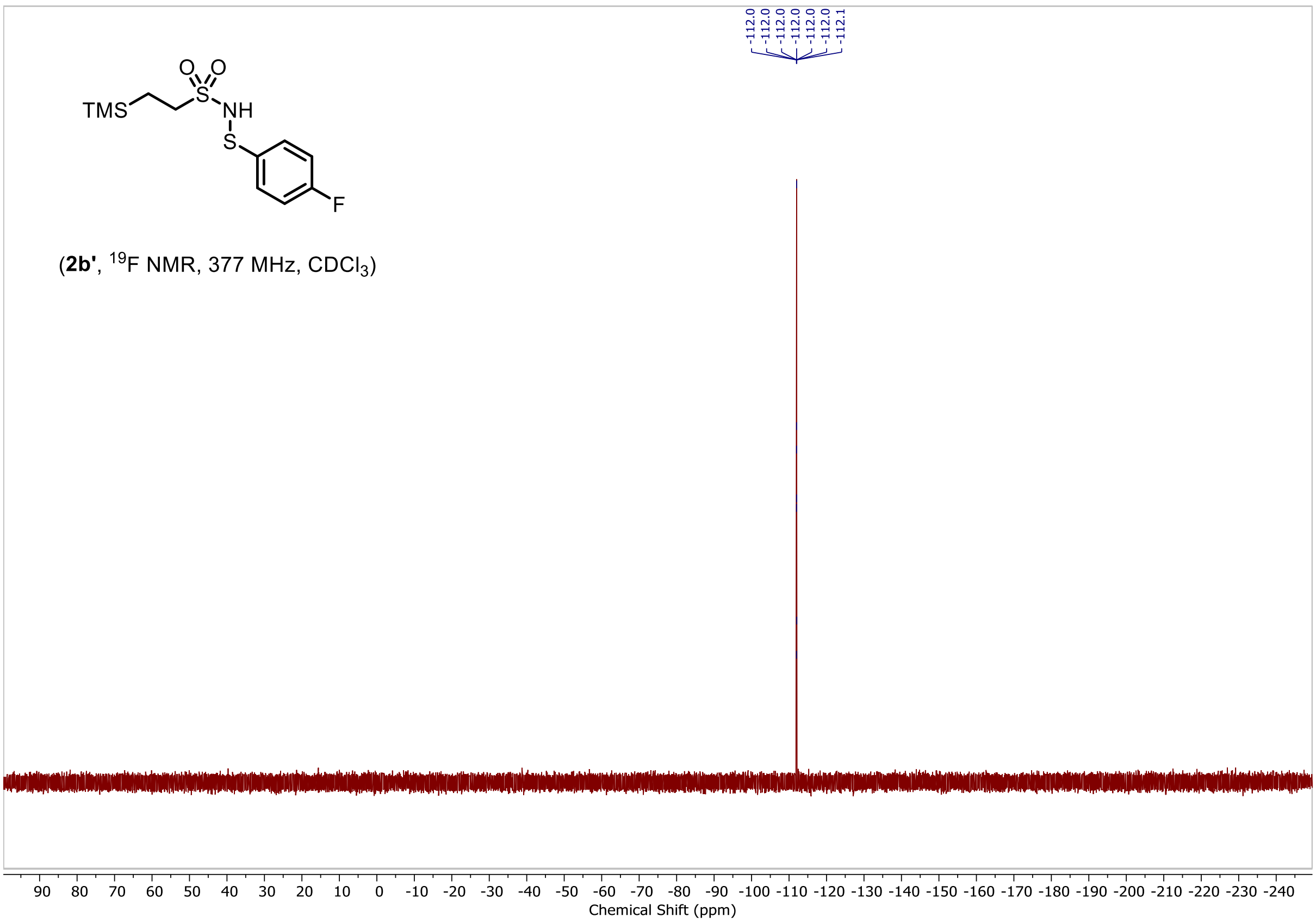
TMS
NH
S
F
(2b', 19F NMR, 377 MHz, CDCl3)
-112.0 -112.0 -112.0 -112.0 -112.0 -112.0 -112.1
90 80 70 60 50 40 30 20 10 0 -10 -20 -30 -40 -50 -60 -70 -80 -90 -100 -110 -120 -130 -140 -150 -160 -170 -180 -190 -200 -210 -220 -230 -240
Chemical Shift (ppm)

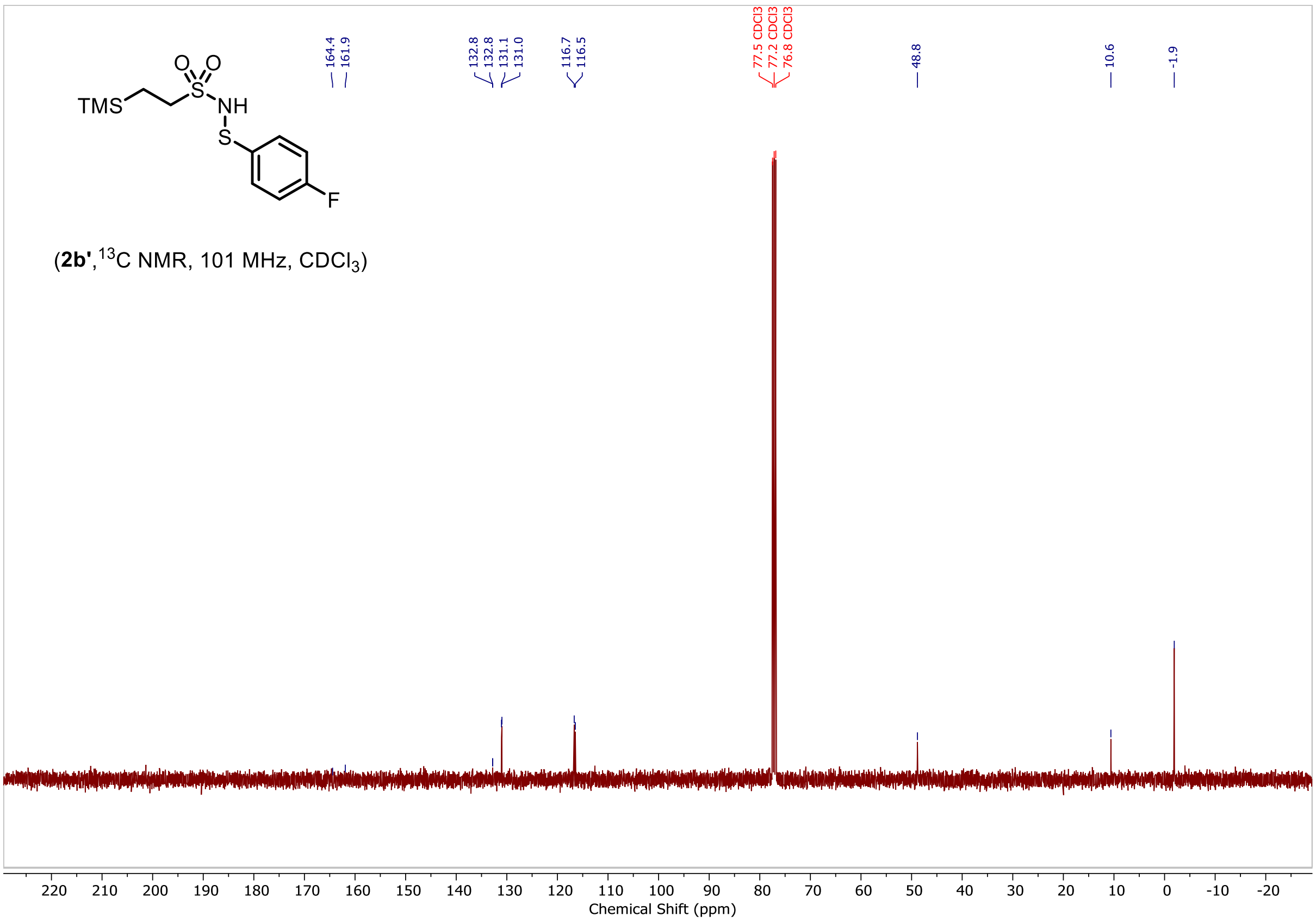
TMS
O
O
S
NH
S
F
(2b', 13C NMR, 101 MHz, CDCl3)
164.4
161.9
132.8
132.8
131.1
131.0
116.7
116.5
77.5 CDCl3
77.2 CDCl3
76.8 CDCl3
48.8
10.6
-1.9
Chemical Shift (ppm)

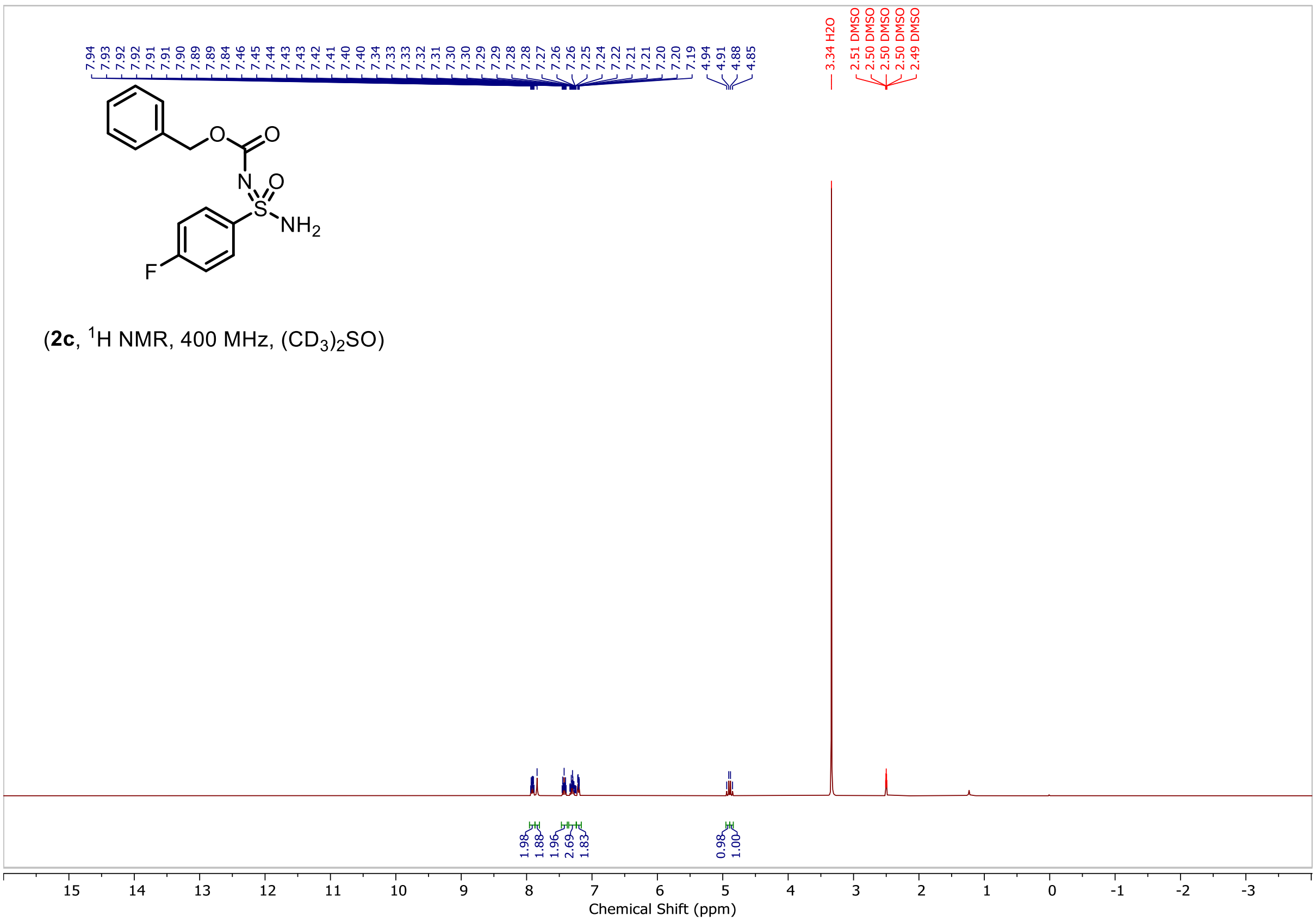
O
O
N
O
S
NH2
F
(2c, 1H NMR, 400 MHz, (CD3)2SO)
3.34 H2O
2.51 DMSO
2.50 DMSO
2.50 DMSO
2.50 DMSO
2.49 DMSO
4.94
4.91
4.88
4.85
1.98
1.88
1.96
2.69
1.83
0.98
1.00
Chemical Shift (ppm)

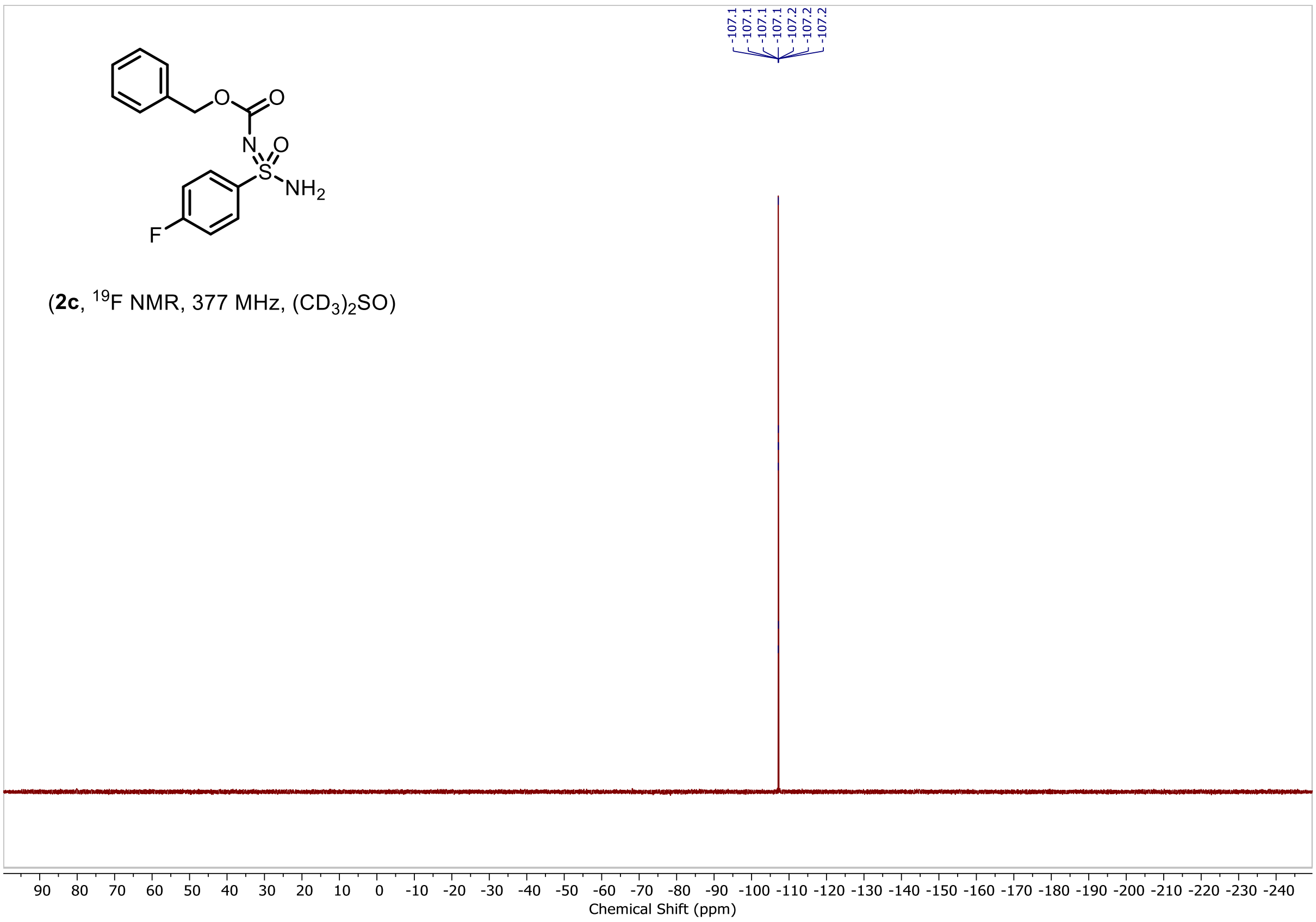

(**2c**, ^{19}F NMR, 377 MHz, $(CD_3)_2SO$)

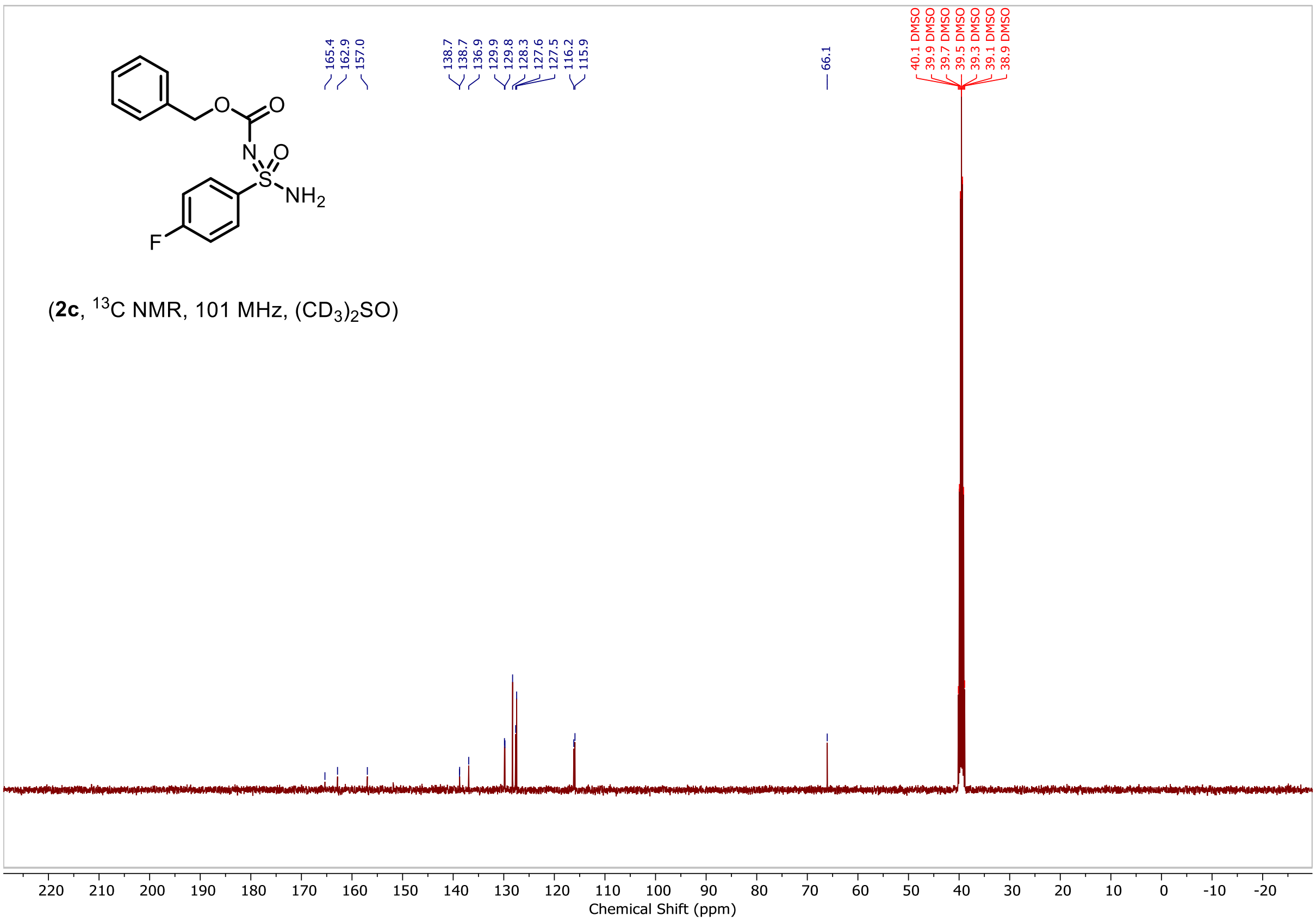

(**2c**, ^{13}C NMR, 101 MHz, $(CD_3)_2SO$)

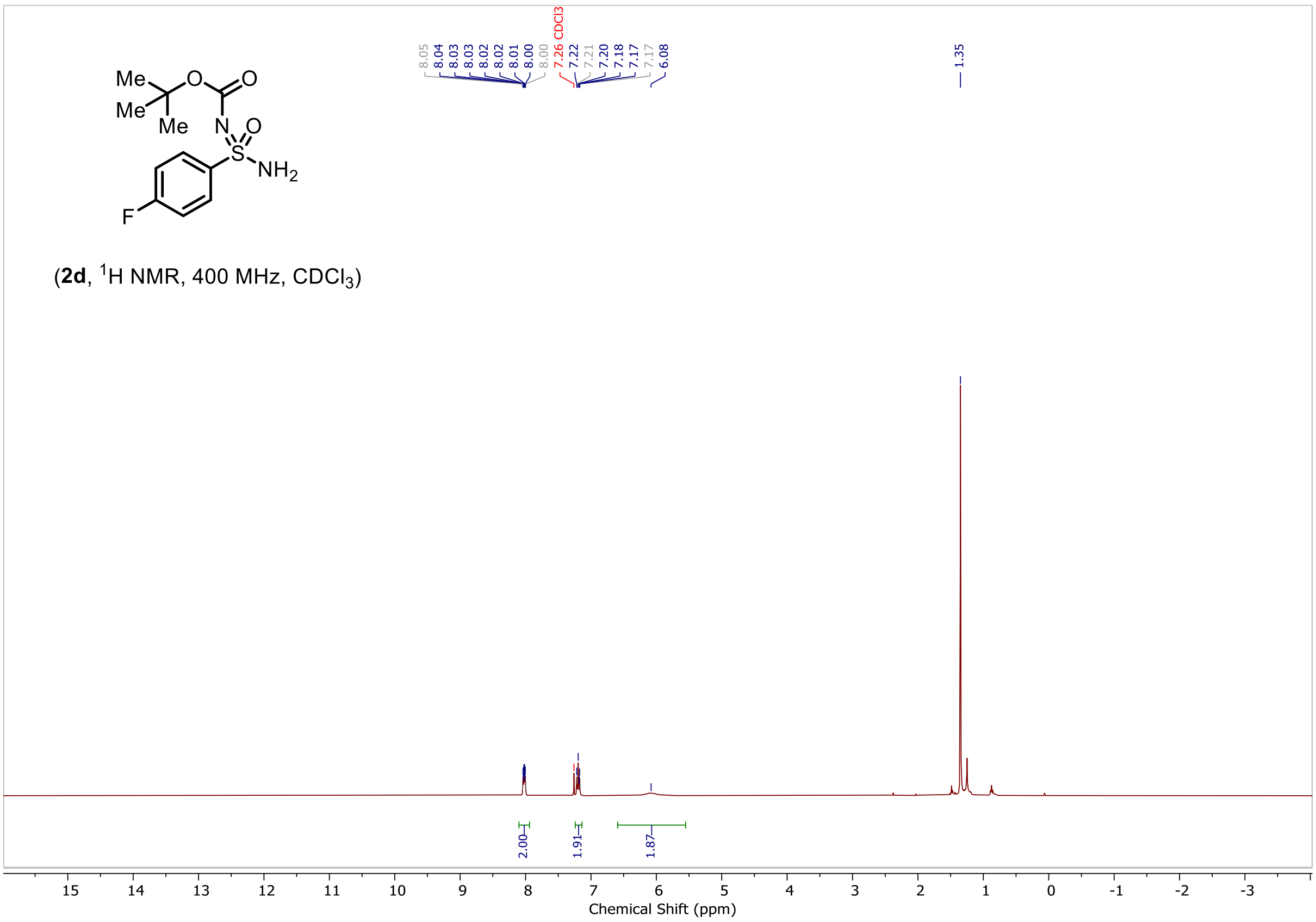

(**2d**, ^{1}H NMR, 400 MHz, $CDCl_3$)

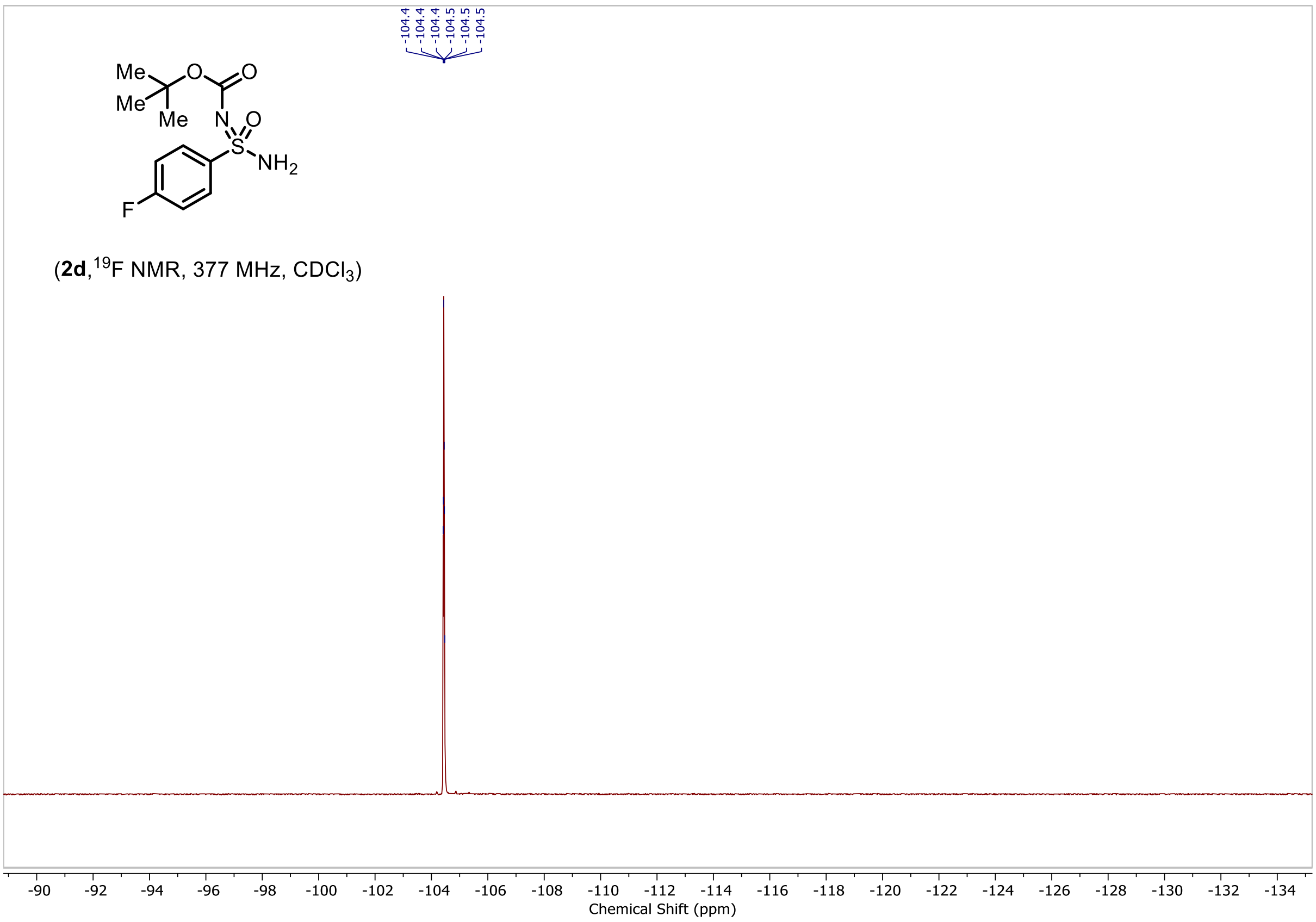

(**2d**, ^{19}F NMR, 377 MHz, $CDCl_3$)

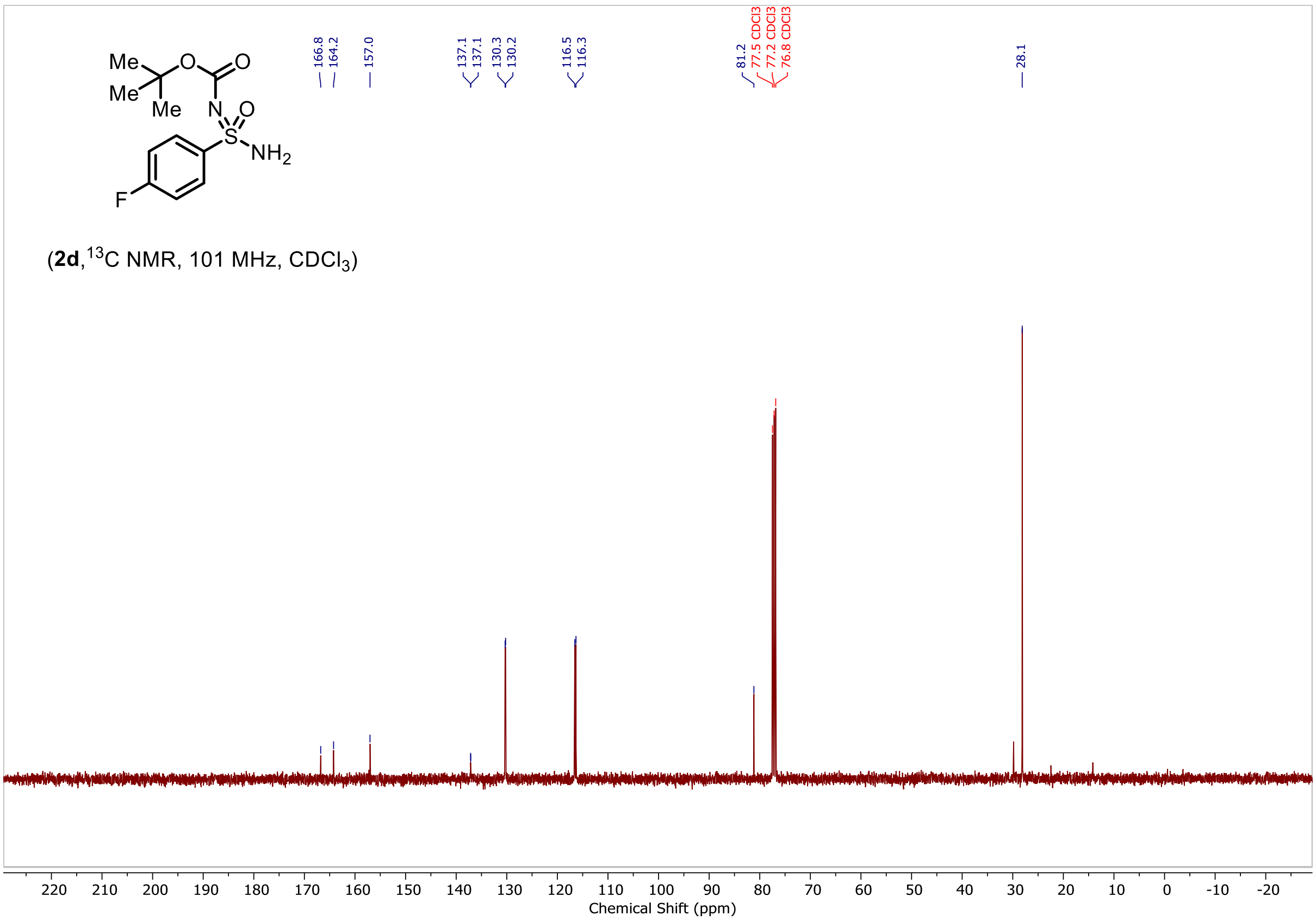

Me
Me
Me
O
O
N
O
S
NH2
F
(2d, 13C NMR, 101 MHz, CDCl3)
166.8
164.2
157.0
137.1
137.1
130.3
130.2
116.5
116.3
81.2
77.5 CDCl3
77.2 CDCl3
76.8 CDCl3
28.1
220 210 200 190 180 170 160 150 140 130 120 110 100 90 80 70 60 50 40 30 20 10 0 -10 -20
Chemical Shift (ppm)

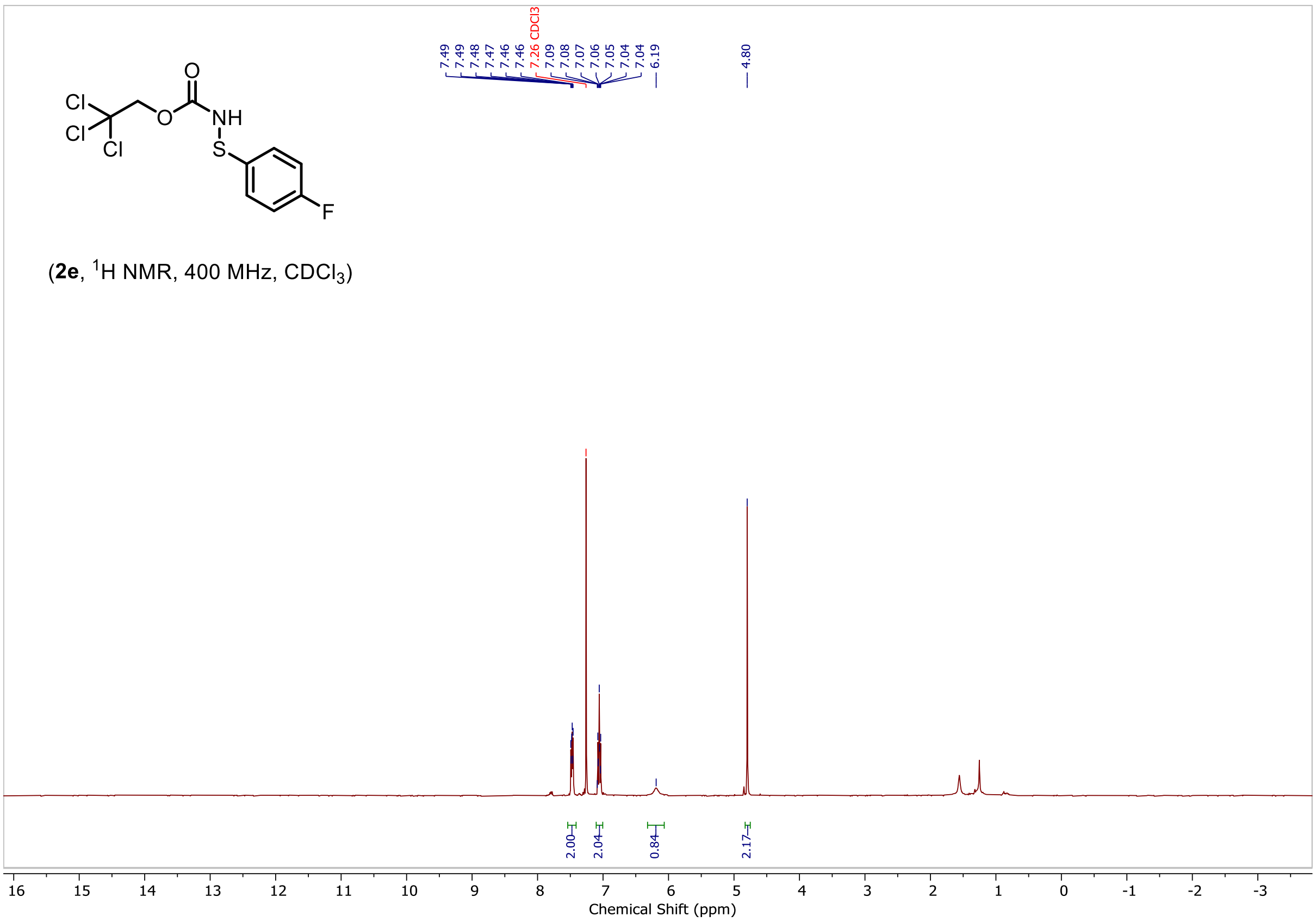

Cl
Cl
Cl
O
O
NH
S
F
(2e, 1H NMR, 400 MHz, CDCl3)
7.49
7.49
7.48
7.47
7.46
7.46
7.26 CDCl3
7.09
7.08
7.07
7.06
7.05
7.04
7.04
6.19
4.80
2.00
2.04
0.84
2.17
16 15 14 13 12 11 10 9 8 7 6 5 4 3 2 1 0 -1 -2 -3
Chemical Shift (ppm)

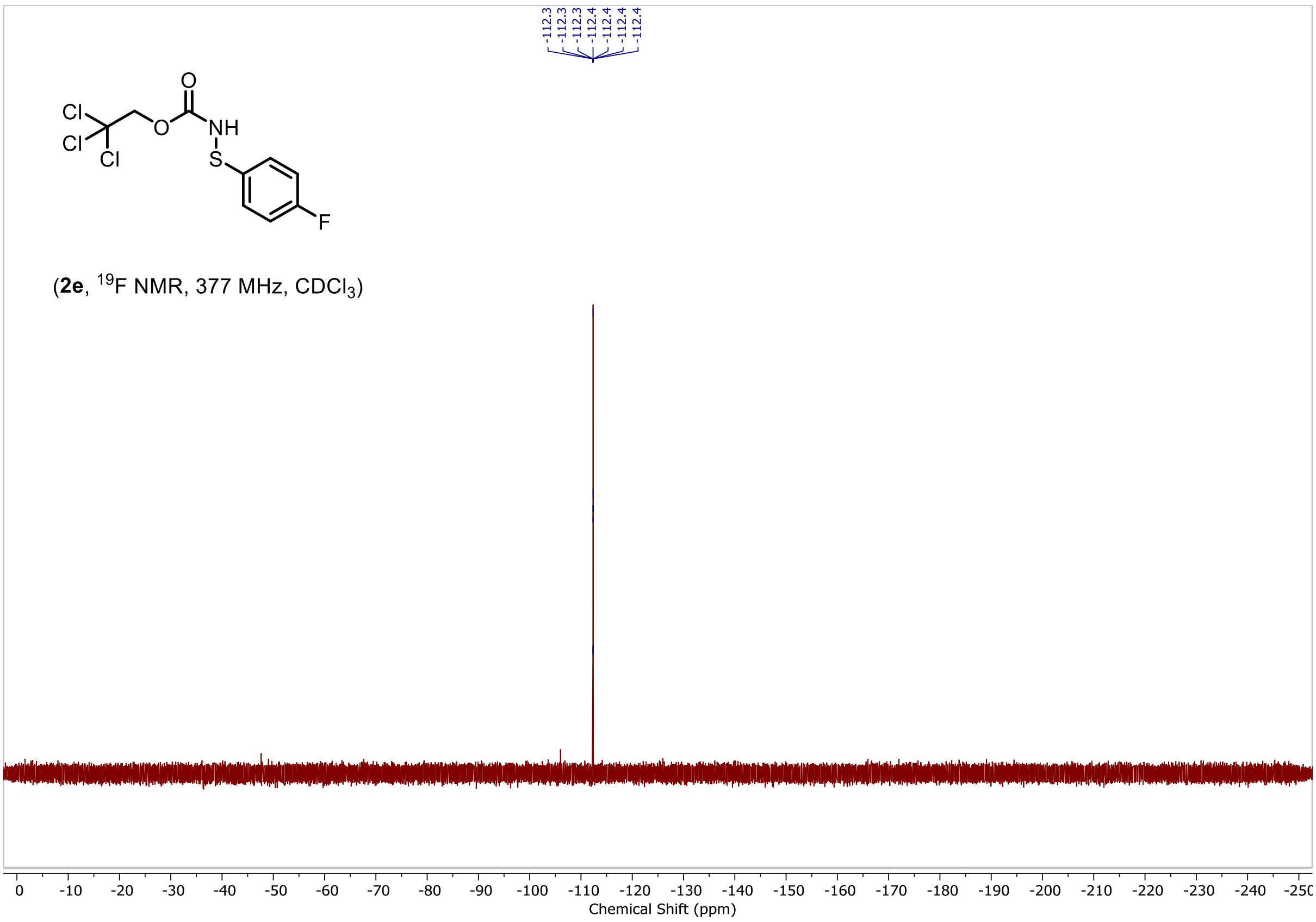

(**2e**, ^{19}F NMR, 377 MHz, $CDCl_3$)

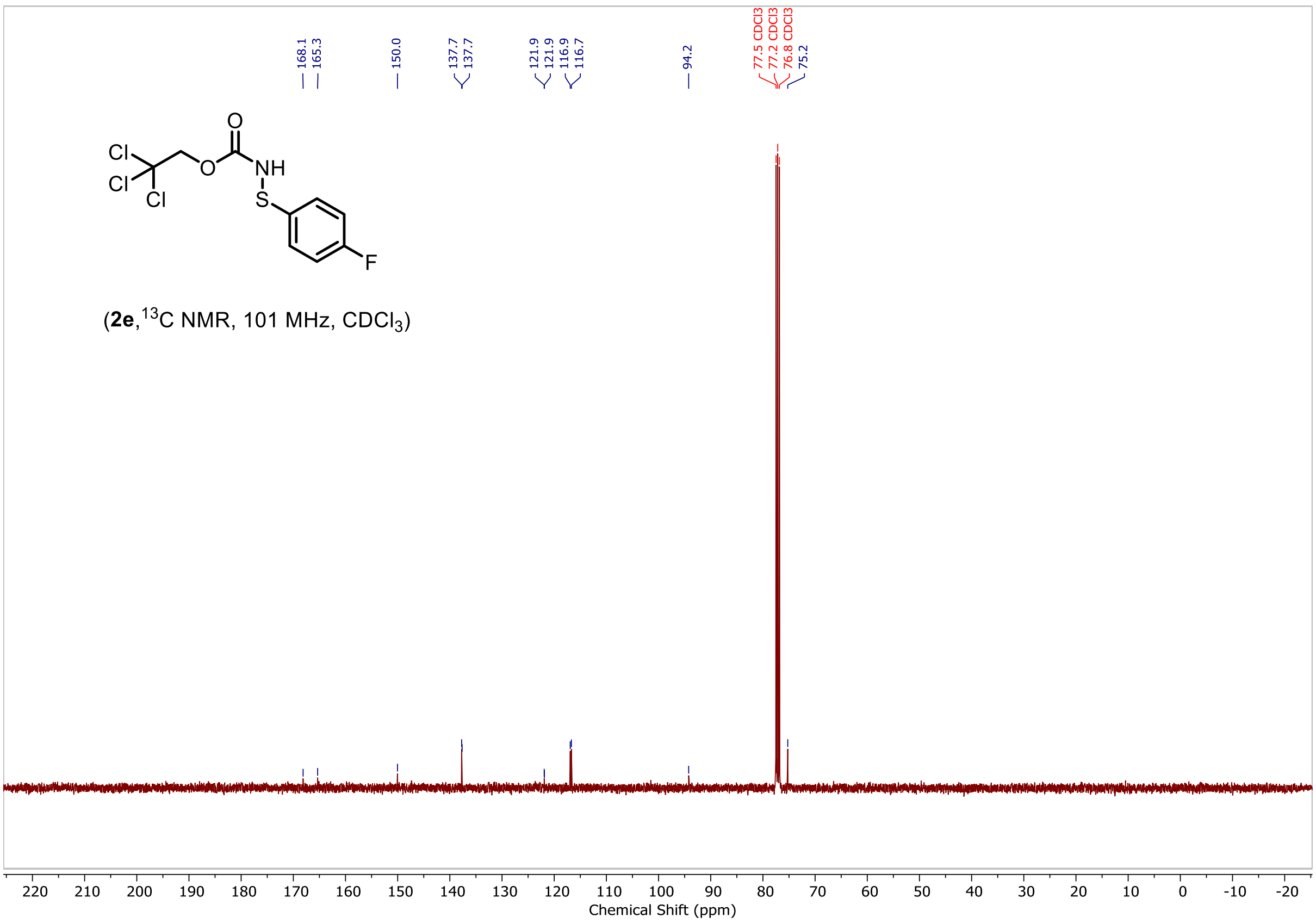

(**2e**, ^{13}C NMR, 101 MHz, $CDCl_3$)

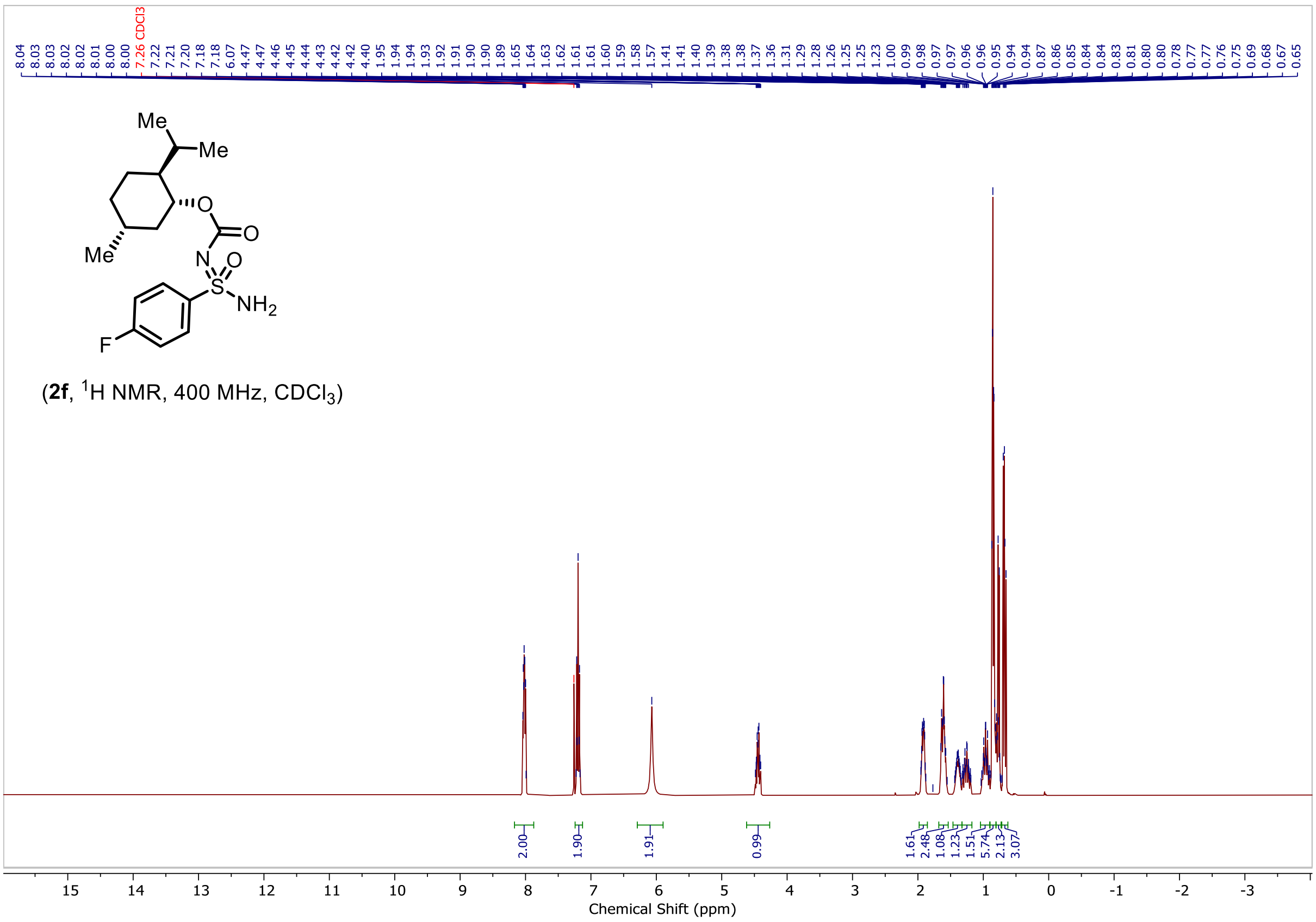

(**2f**, ^{1}H NMR, 400 MHz, $CDCl_3$)

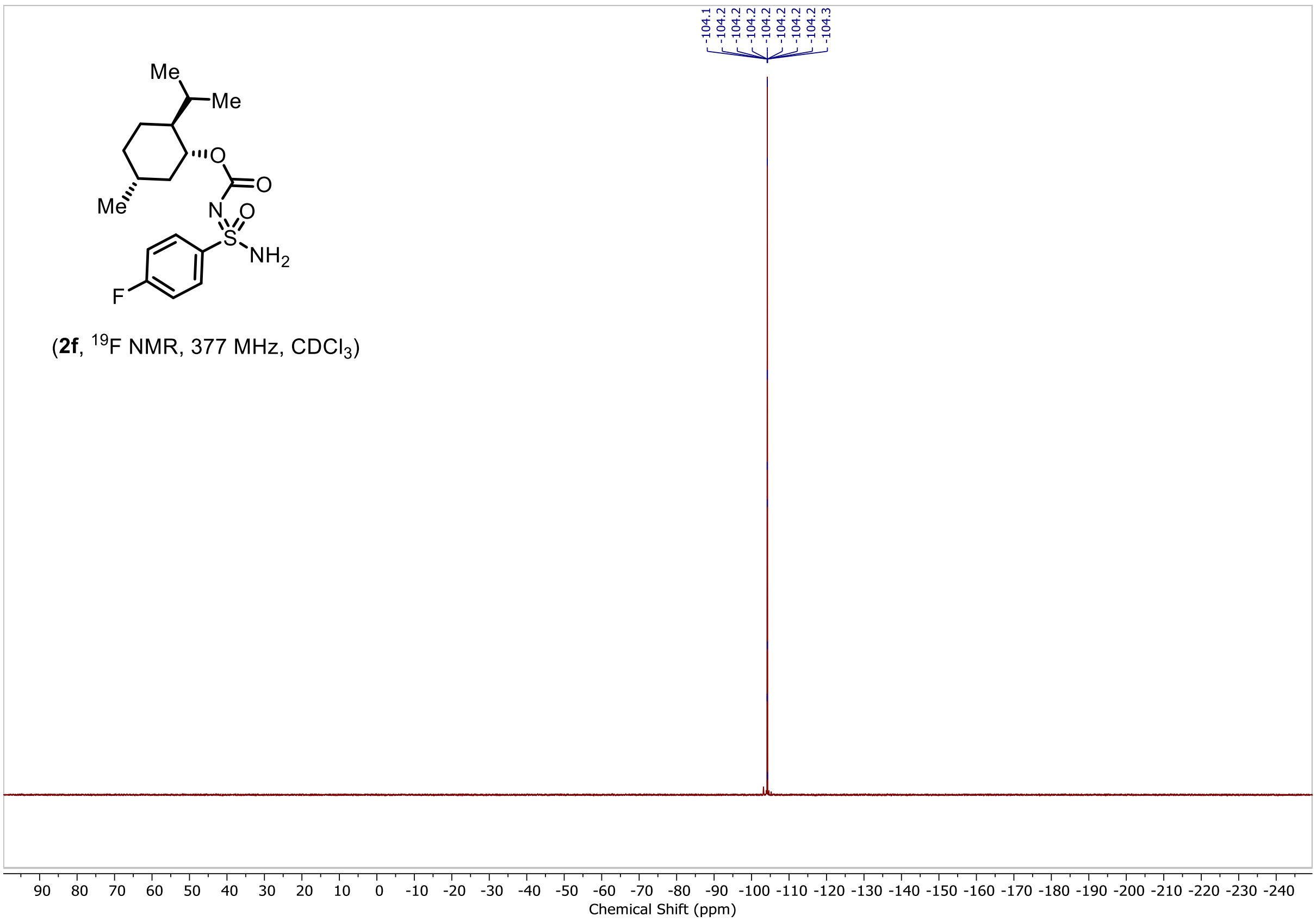

(**2f**, ^{19}F NMR, 377 MHz, $CDCl_3$)

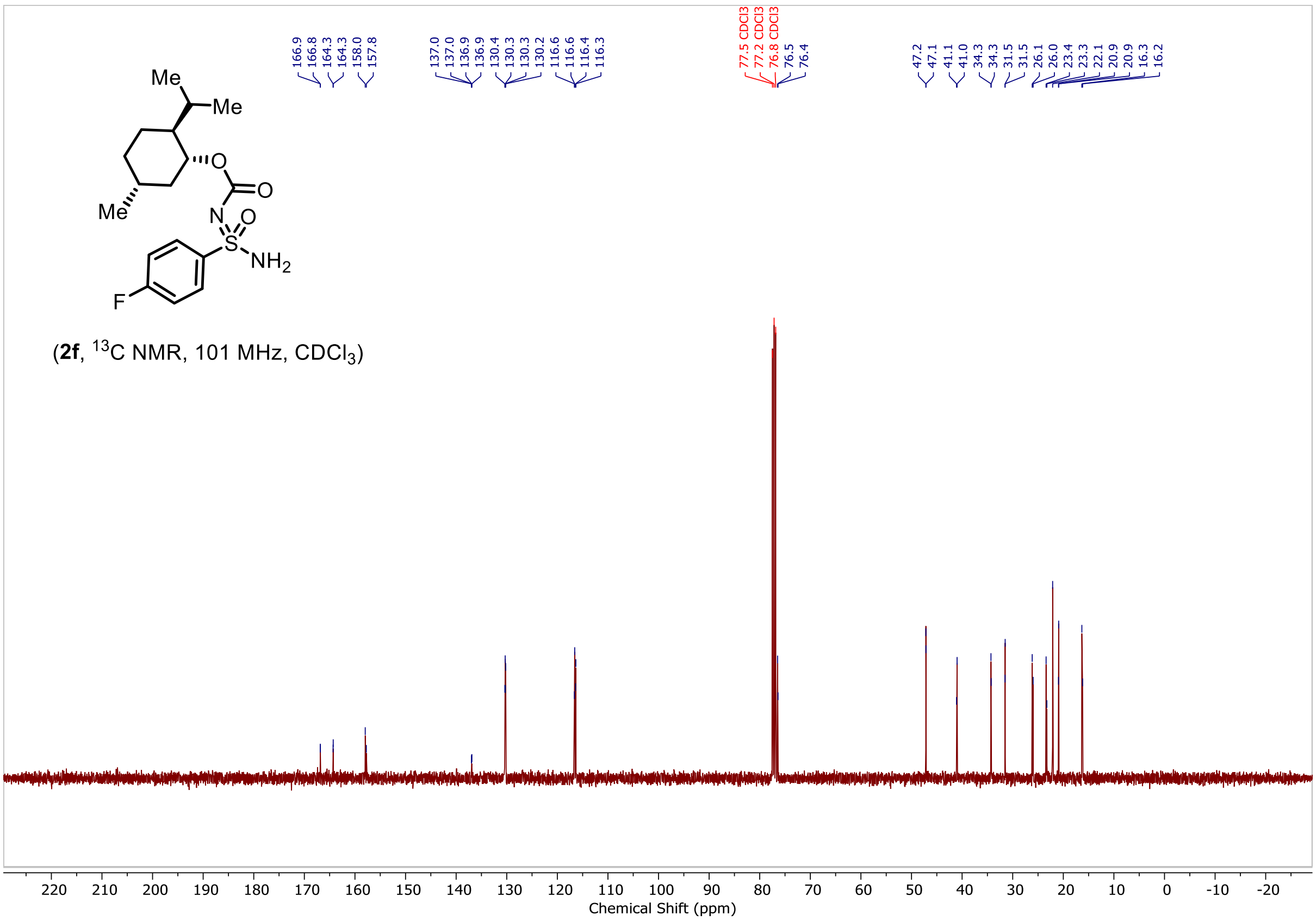

(**2f**, ^{13}C NMR, 101 MHz, $CDCl_3$)

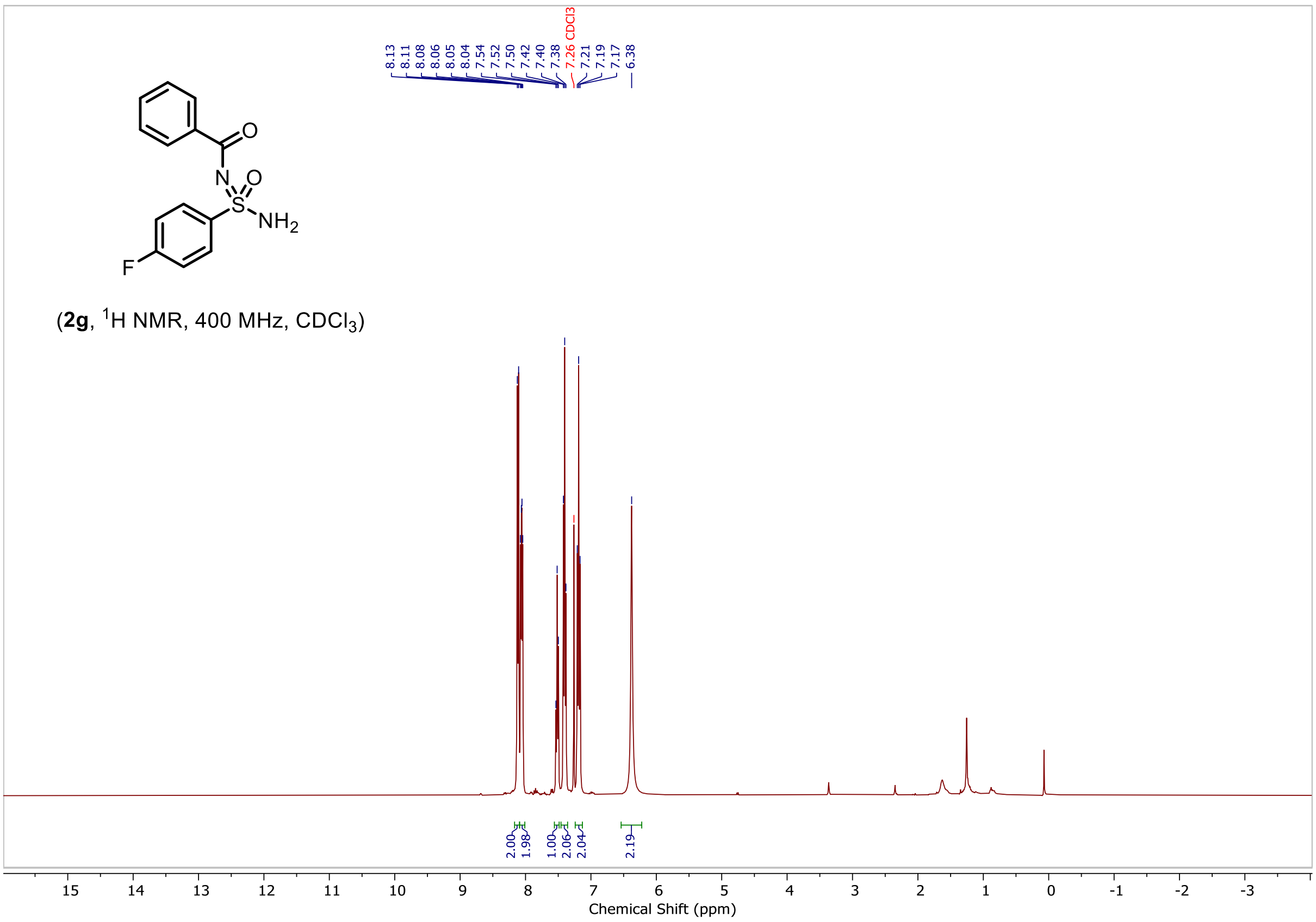

(**2g**, ^{1}H NMR, 400 MHz, $CDCl_3$)

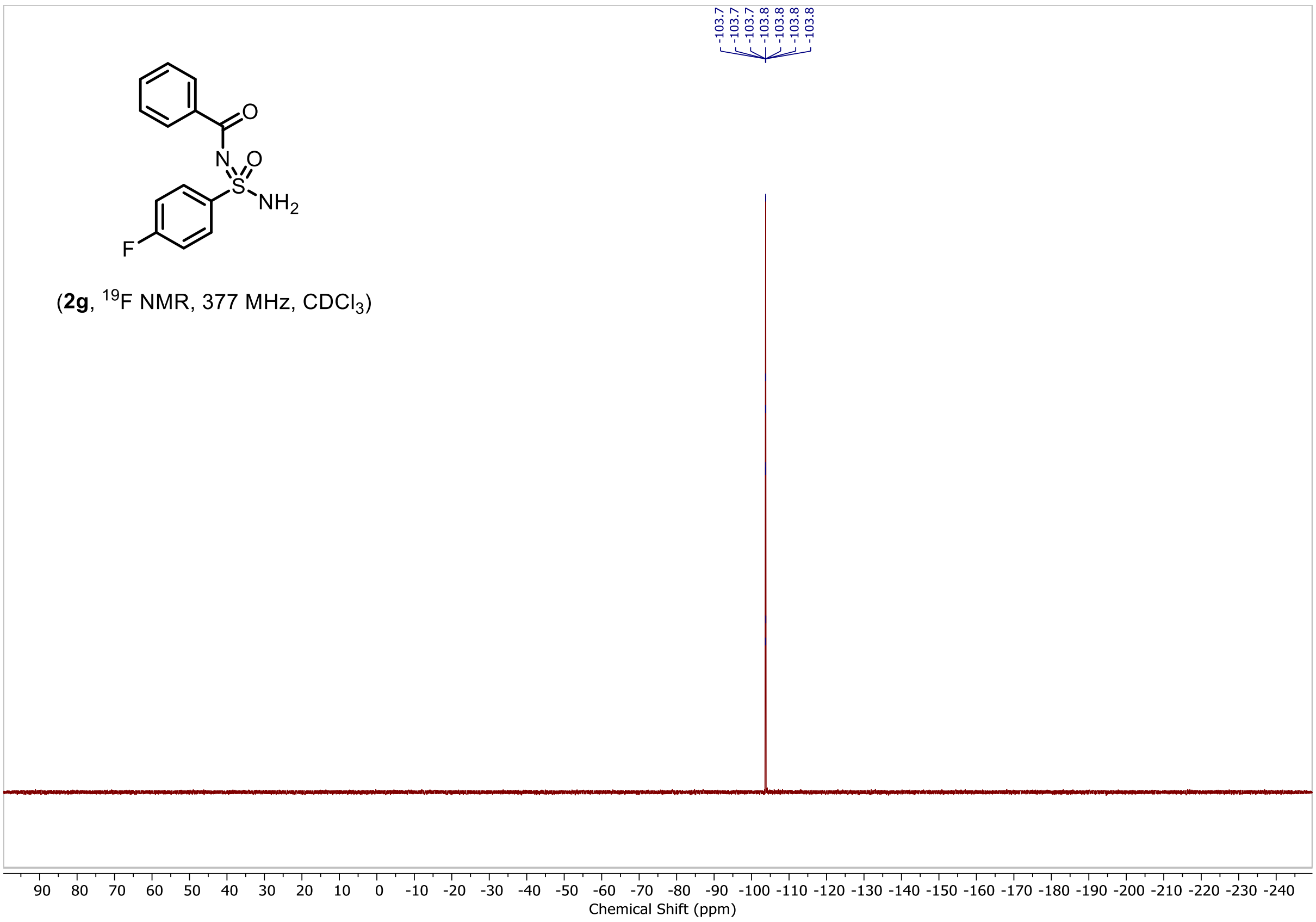
-103.7
-103.7
-103.7
-103.8
-103.8
-103.8
-103.8
O
N
S
O
NH2
F
(2g, 19F NMR, 377 MHz, CDCl3)
90 80 70 60 50 40 30 20 10 0 -10 -20 -30 -40 -50 -60 -70 -80 -90 -100 -110 -120 -130 -140 -150 -160 -170 -180 -190 -200 -210 -220 -230 -240
Chemical Shift (ppm)

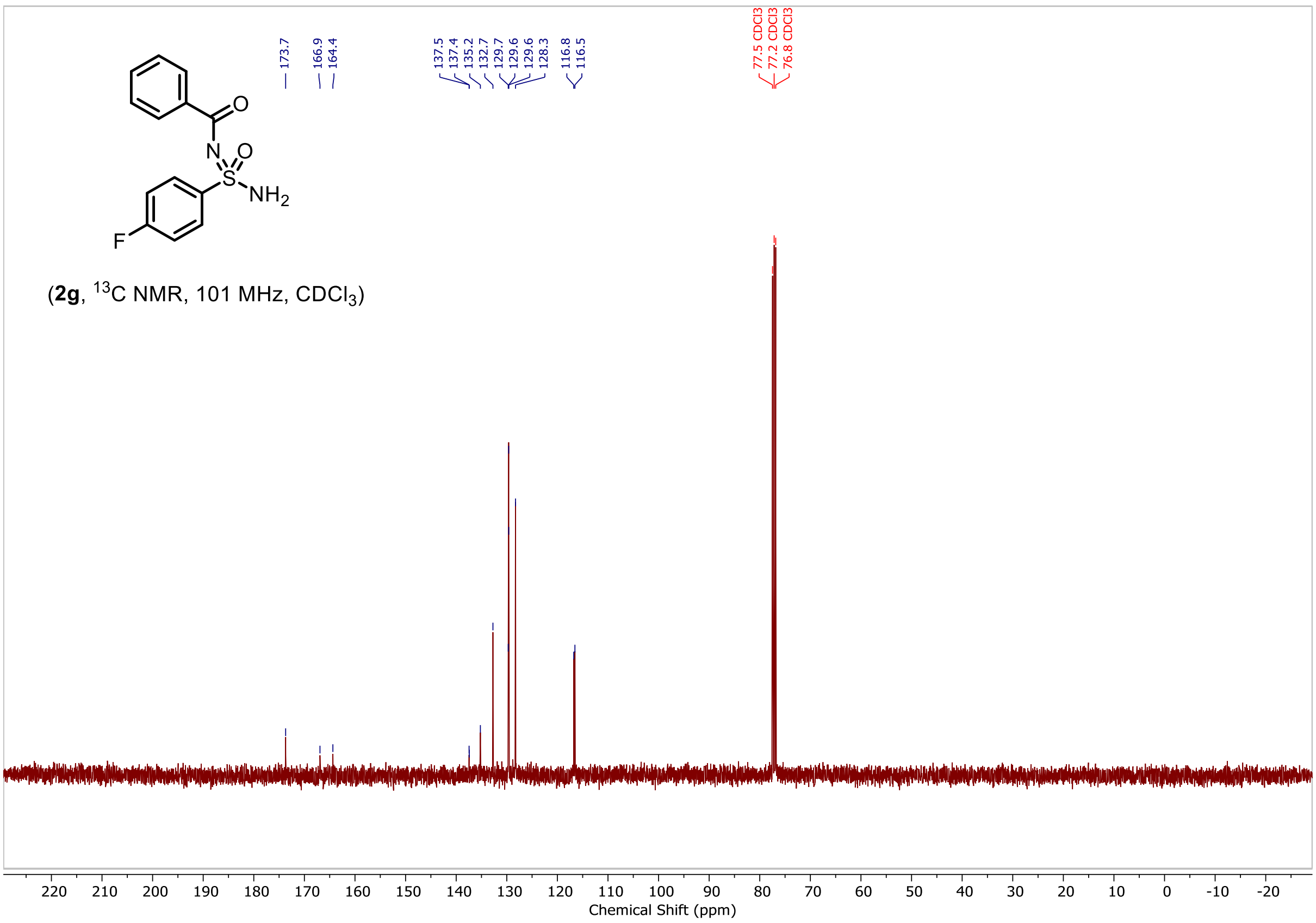
173.7
166.9
164.4
137.5
137.4
135.2
132.7
129.7
129.6
129.6
128.3
116.8
116.5
77.5 CDCl3
77.2 CDCl3
76.8 CDCl3
O
N
S
O
NH2
F
(2g, 13C NMR, 101 MHz, CDCl3)
220 210 200 190 180 170 160 150 140 130 120 110 100 90 80 70 60 50 40 30 20 10 0 -10 -20
Chemical Shift (ppm)

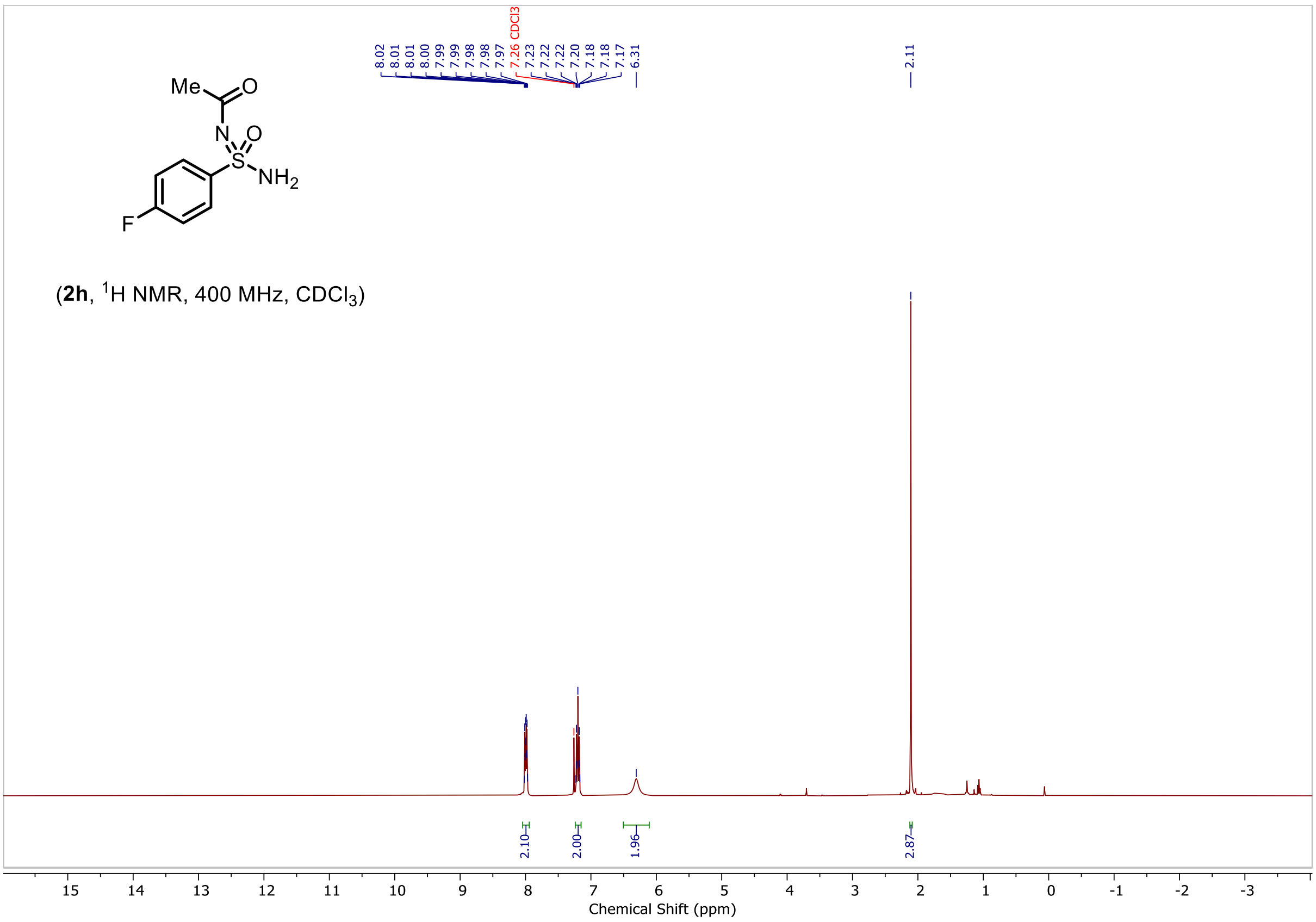

(**2h**, ^{1}H NMR, 400 MHz, $CDCl_3$)

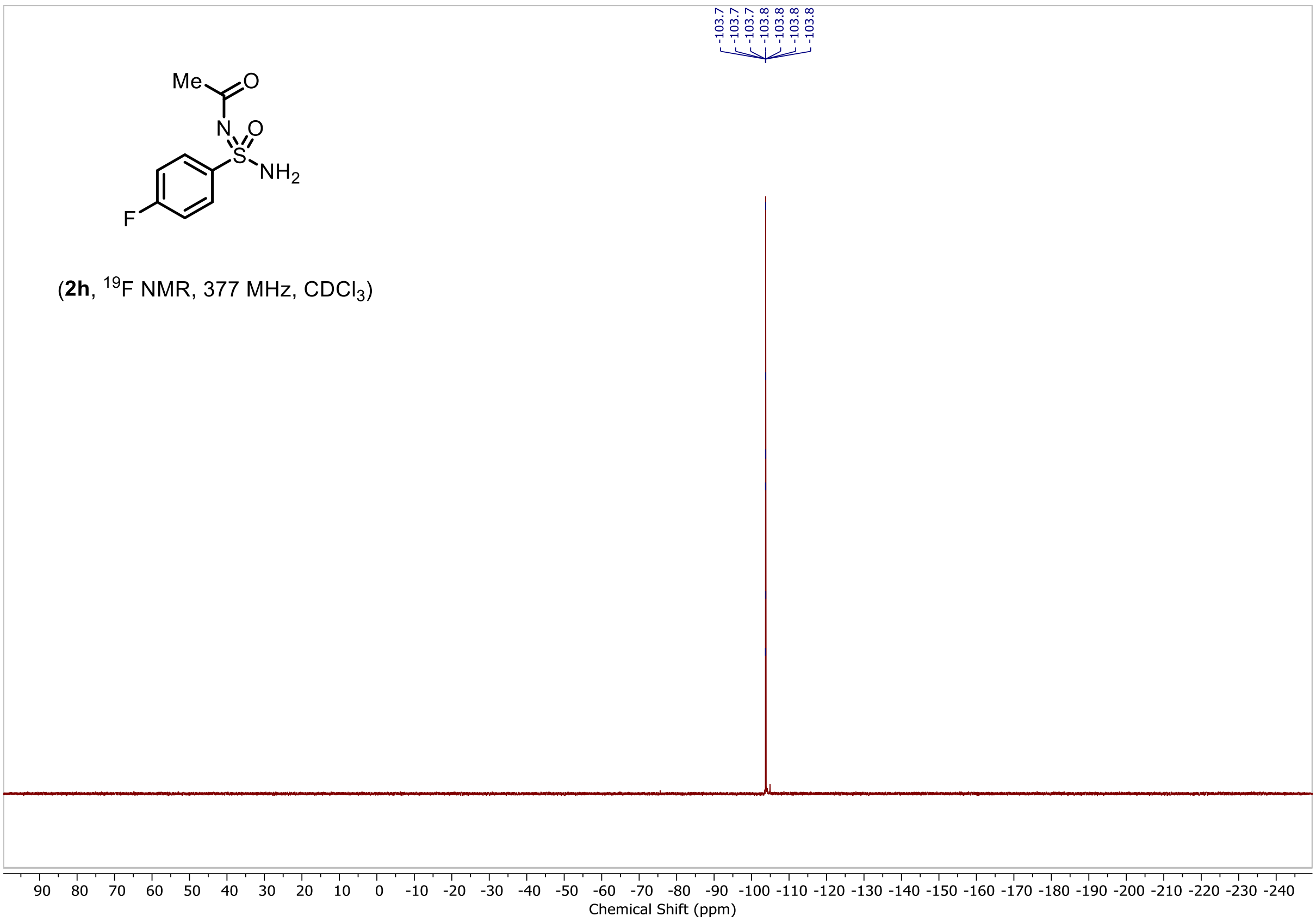

(**2h**, ^{19}F NMR, 377 MHz, $CDCl_3$)

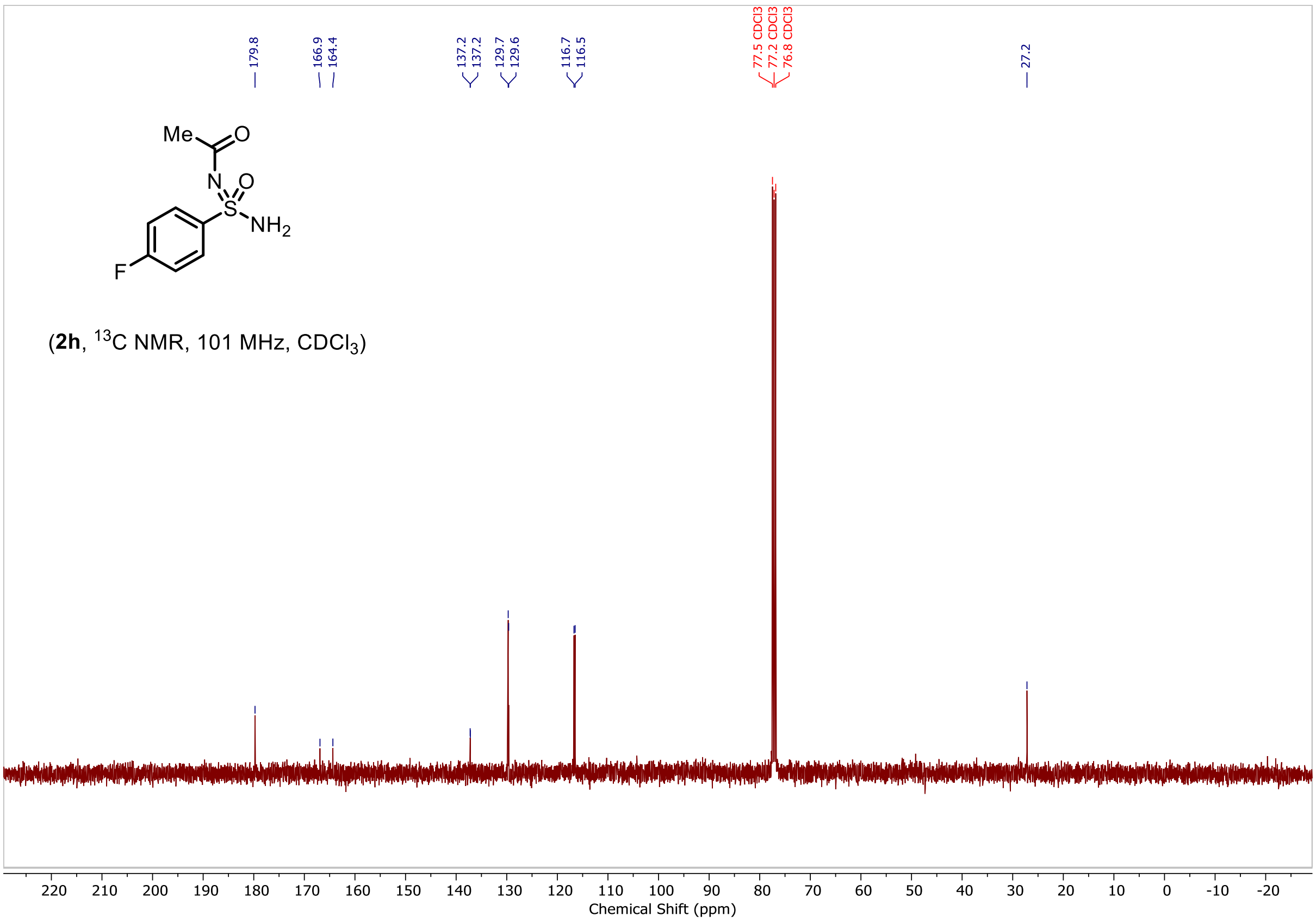

(**2h**, ^{13}C NMR, 101 MHz, $CDCl_3$)

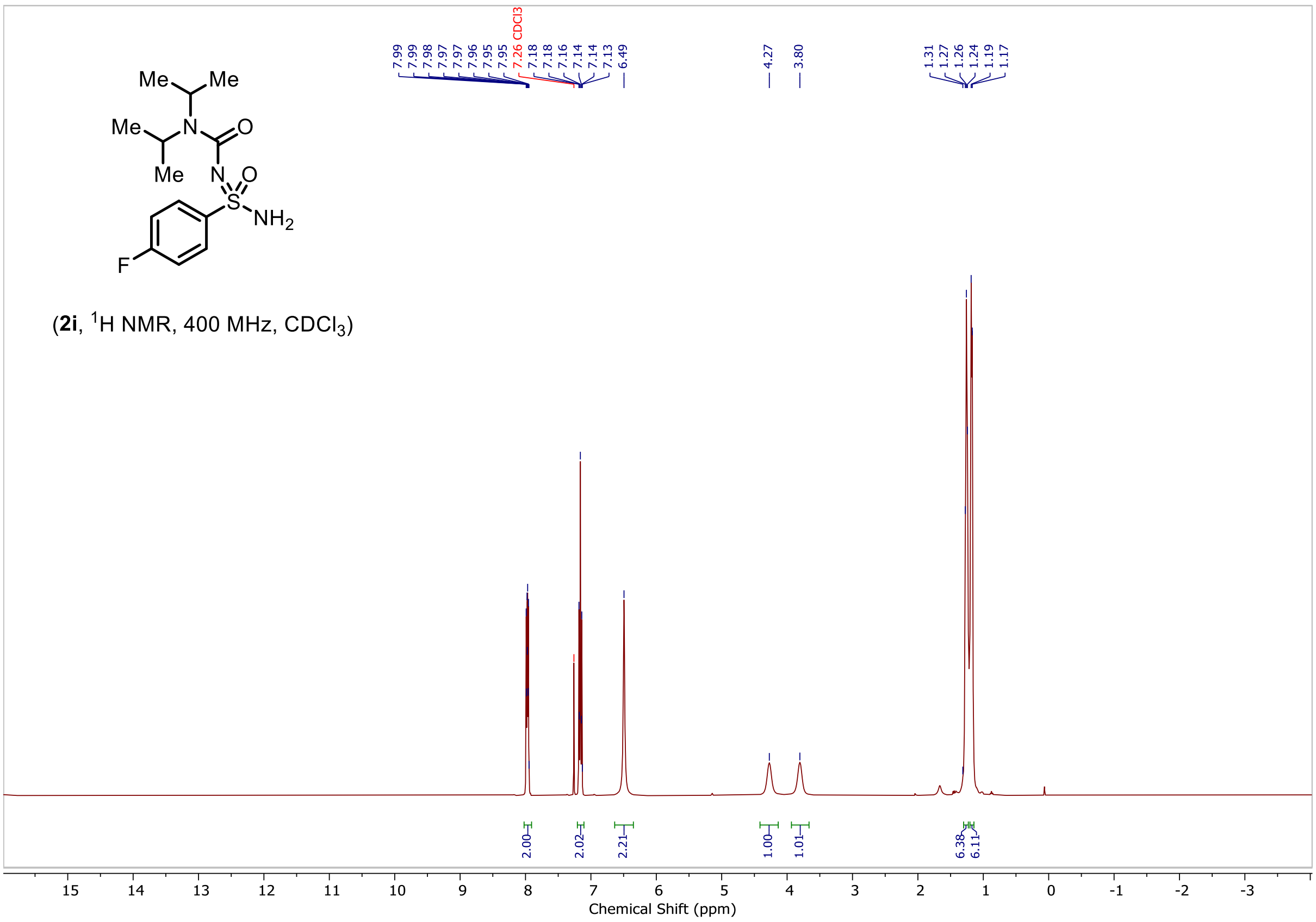

(**2i**, ^{1}H NMR, 400 MHz, $CDCl_3$)

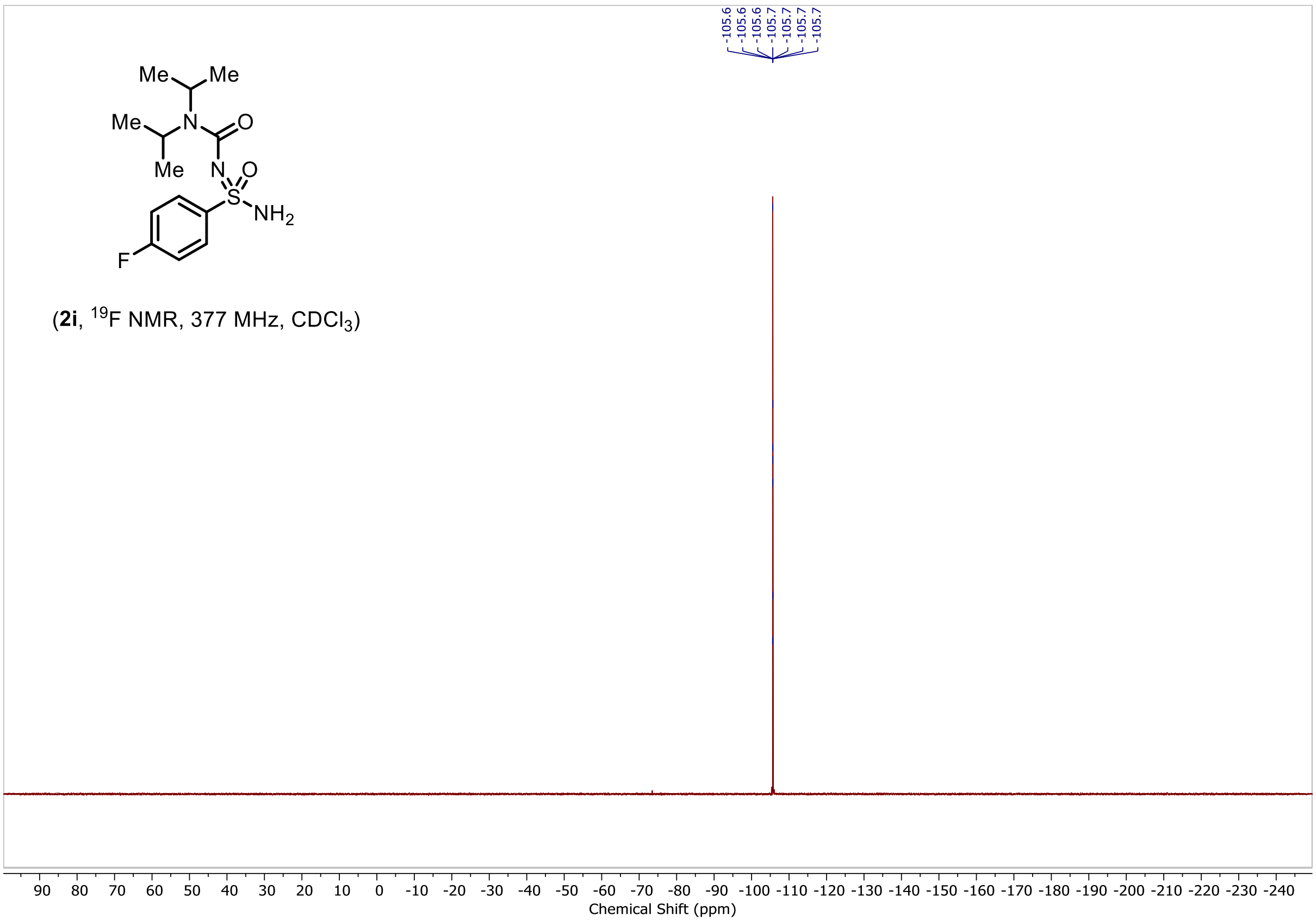
-105.6
-105.6
-105.6
-105.7
-105.7
-105.7
-105.7
Me
Me
Me
N
O
Me
N
O
S
NH2
F
(2i, 19F NMR, 377 MHz, CDCl3)
90 80 70 60 50 40 30 20 10 0 -10 -20 -30 -40 -50 -60 -70 -80 -90 -100 -110 -120 -130 -140 -150 -160 -170 -180 -190 -200 -210 -220 -230 -240
Chemical Shift (ppm)

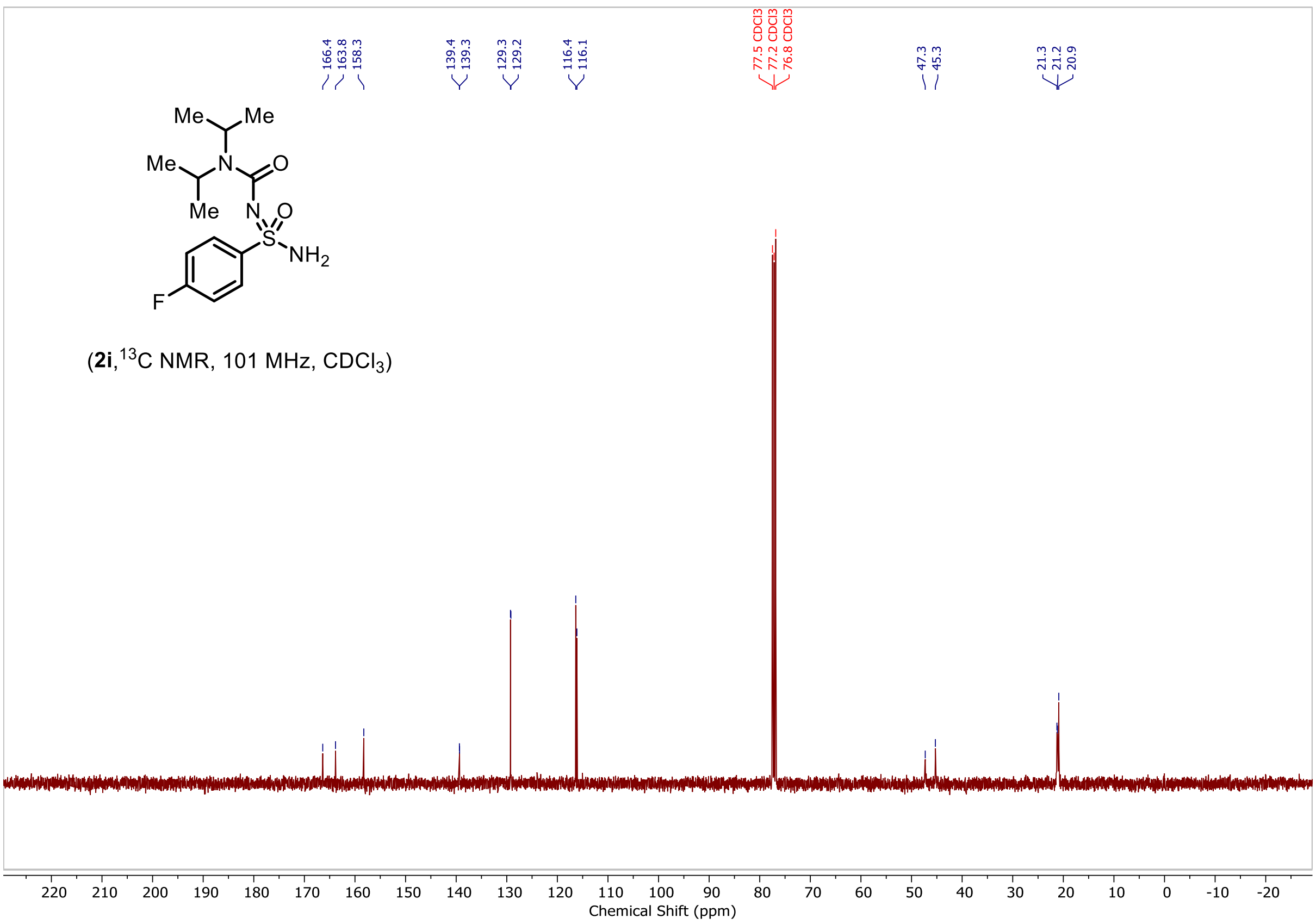
166.4
163.8
158.3
139.4
139.3
129.3
129.2
116.4
116.1
77.5 CDCl3
77.2 CDCl3
76.8 CDCl3
47.3
45.3
21.3
21.2
20.9
Me
Me
Me
N
O
Me
N
O
S
NH2
F
(2i,13C NMR, 101 MHz, CDCl3)
220 210 200 190 180 170 160 150 140 130 120 110 100 90 80 70 60 50 40 30 20 10 0 -10 -20
Chemical Shift (ppm)

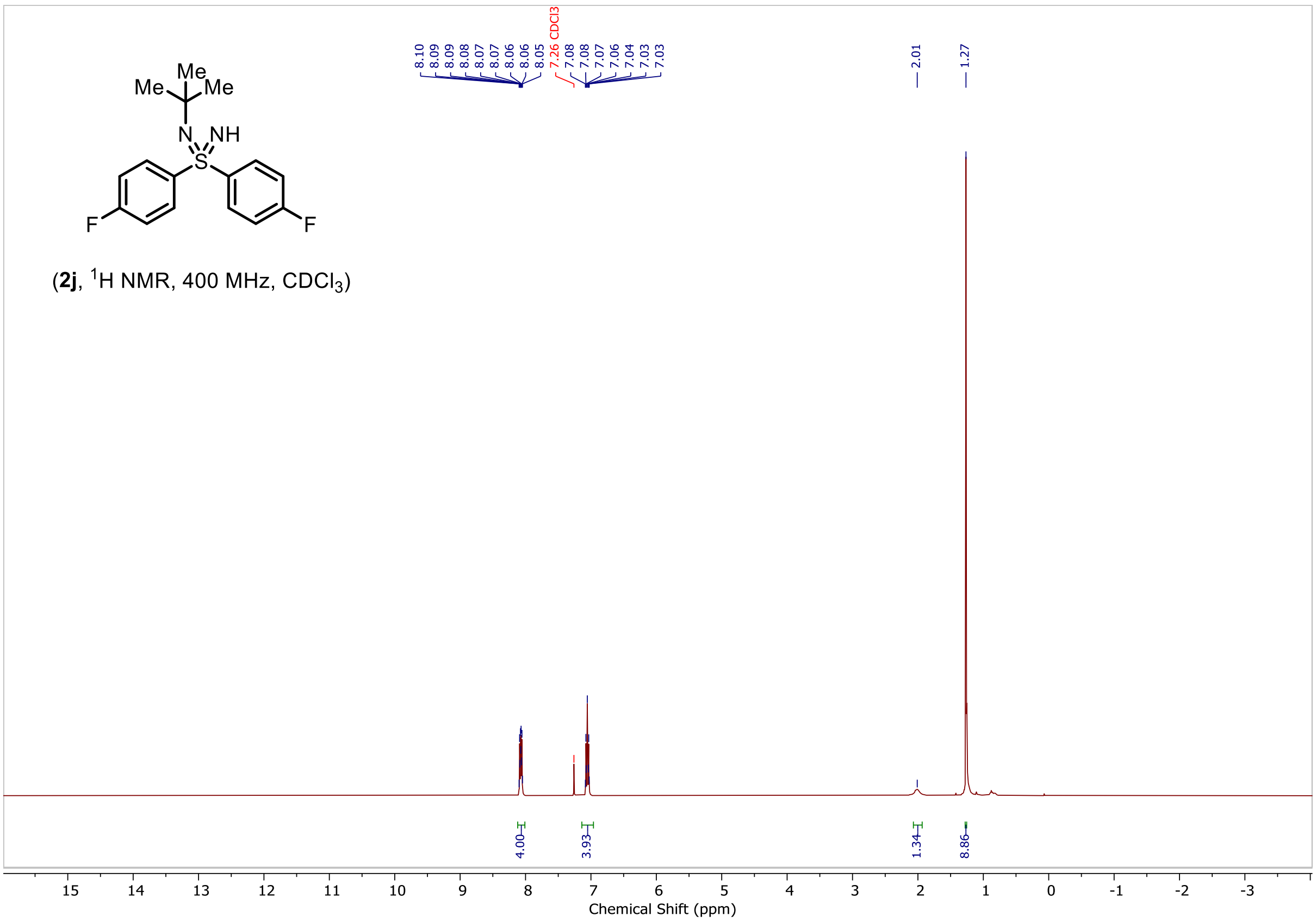
8.10
8.09
8.09
8.08
8.07
8.07
8.06
8.06
8.05
7.26 CDCl3
7.08
7.08
7.07
7.06
7.04
7.03
7.03
2.01
1.27
Me
Me
Me
N
NH
S
F
F
(2j, 1H NMR, 400 MHz, CDCl3)
4.00
3.93
1.34
8.86
Chemical Shift (ppm)

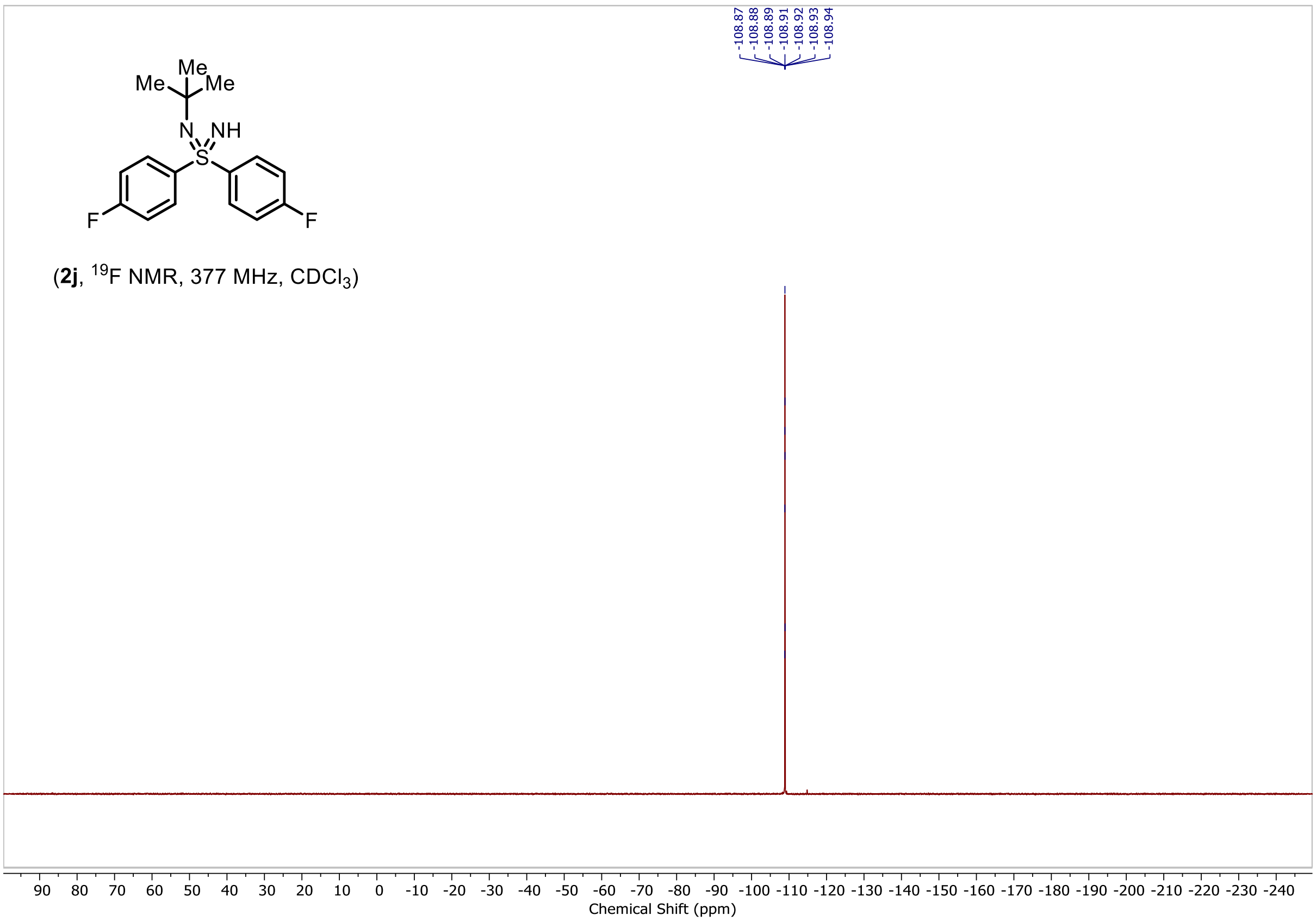
-108.87
-108.88
-108.89
-108.91
-108.92
-108.93
-108.94
Me
Me
Me
N
NH
S
F
F
(2j, 19F NMR, 377 MHz, CDCl3)
Chemical Shift (ppm)

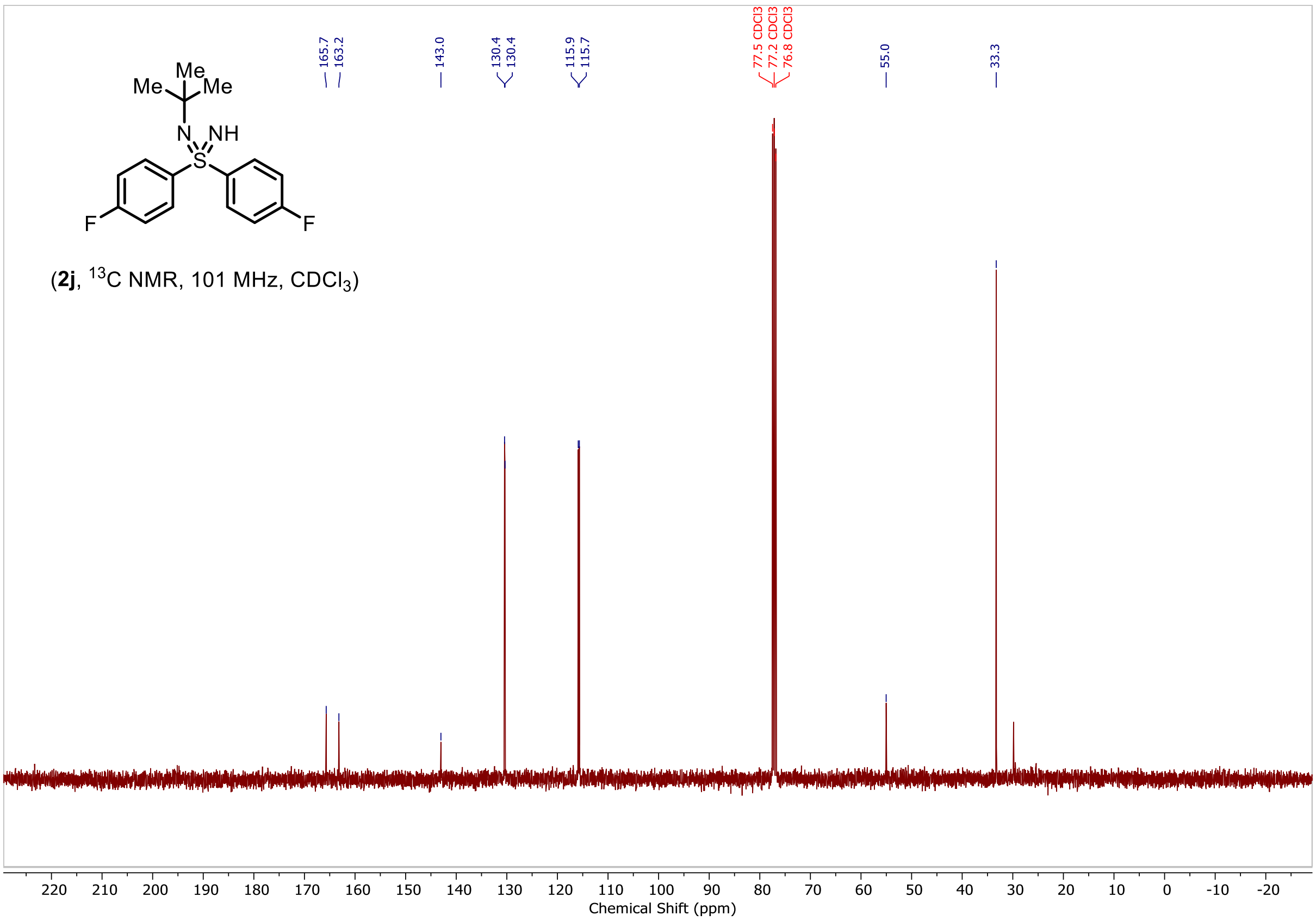

(**2j**, ^{13}C NMR, 101 MHz, $CDCl_3$)

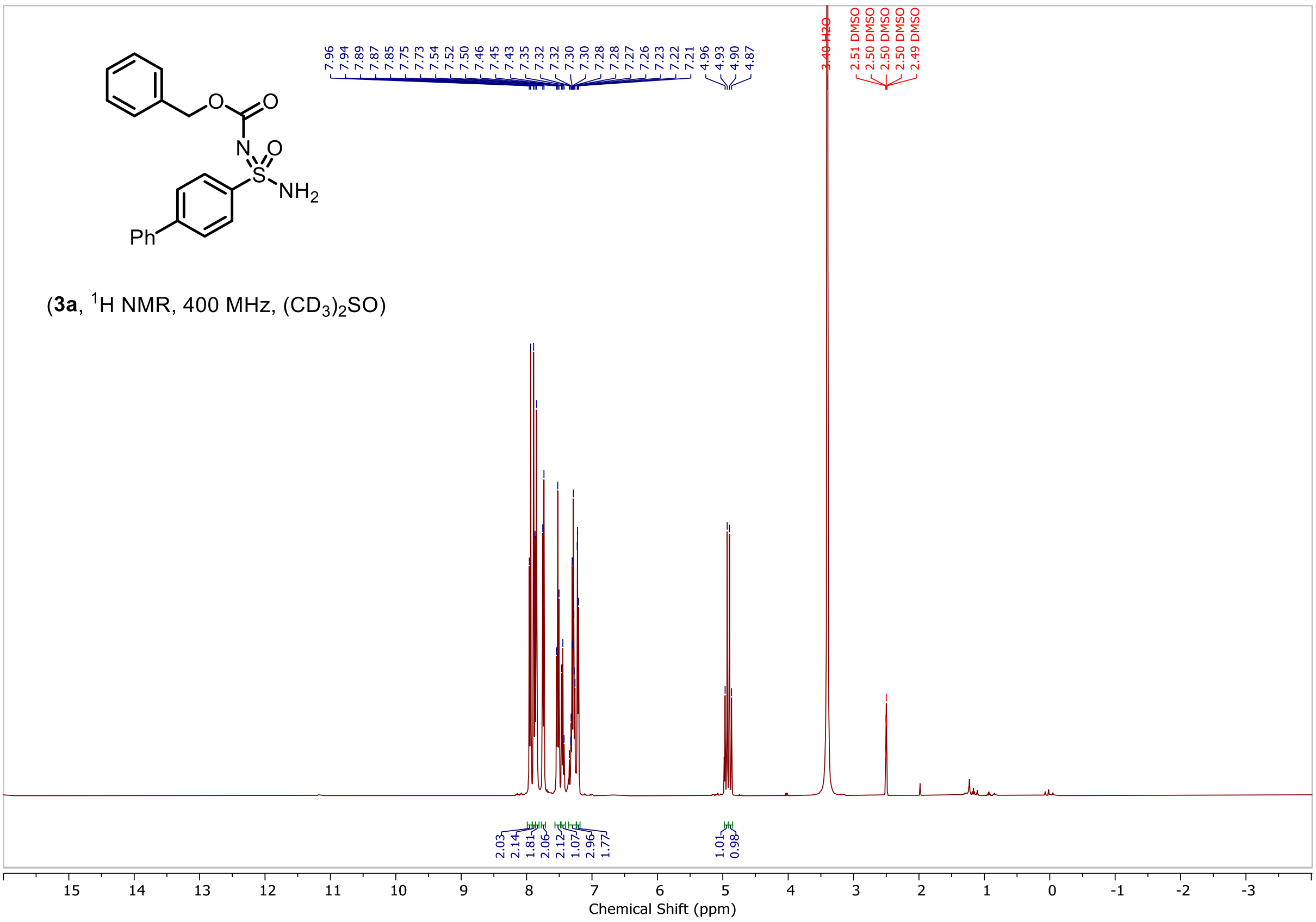

(**3a**, ^{1}H NMR, 400 MHz, $(CD_3)_2SO$)

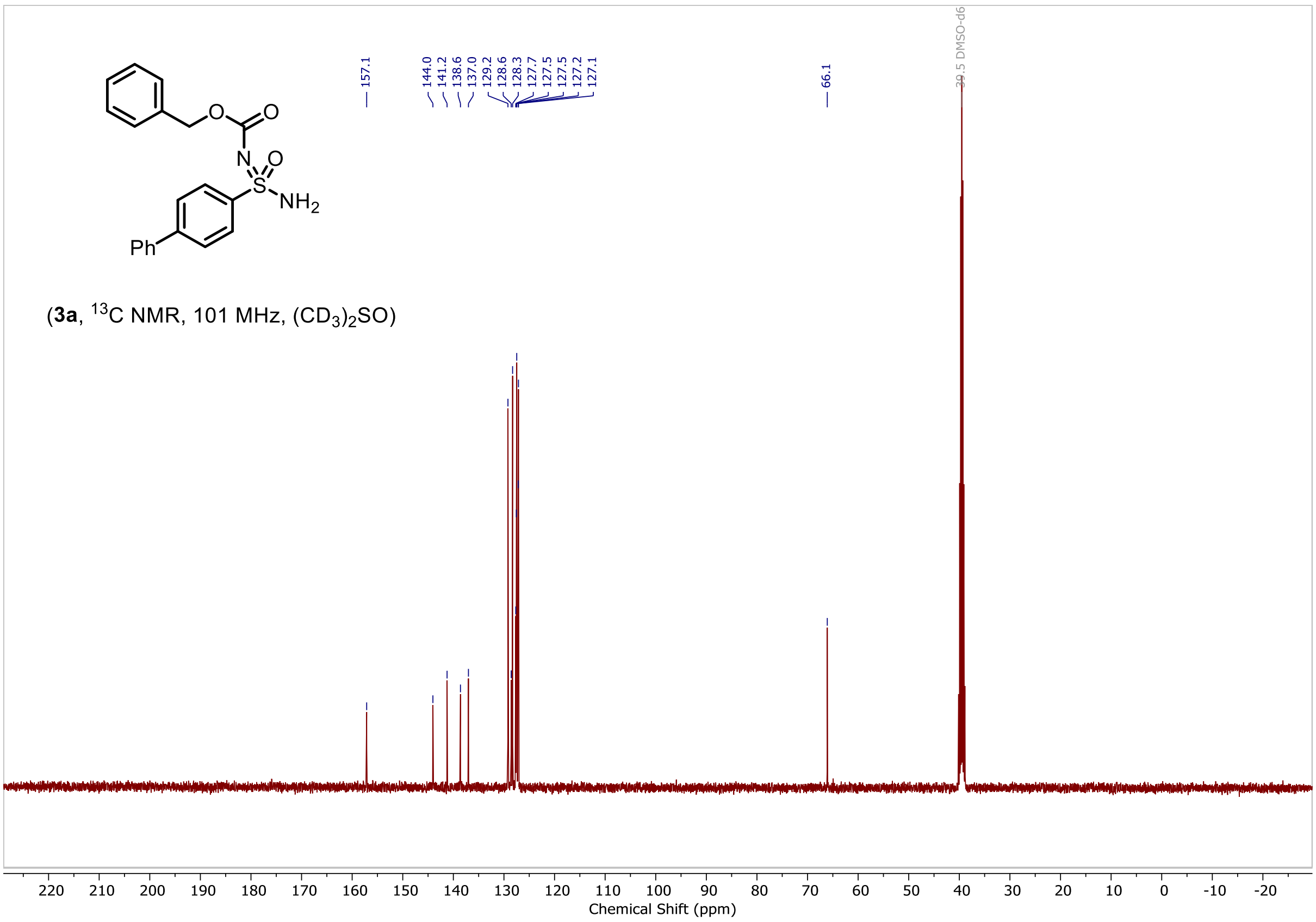
(3a, 13C NMR, 101 MHz, (CD3)2SO)
157.1
144.0
141.2
138.6
137.0
129.2
128.6
128.3
127.7
127.5
127.5
127.2
127.1
66.1
39.5 DMSO-d6
NH2
Ph
Chemical Shift (ppm)

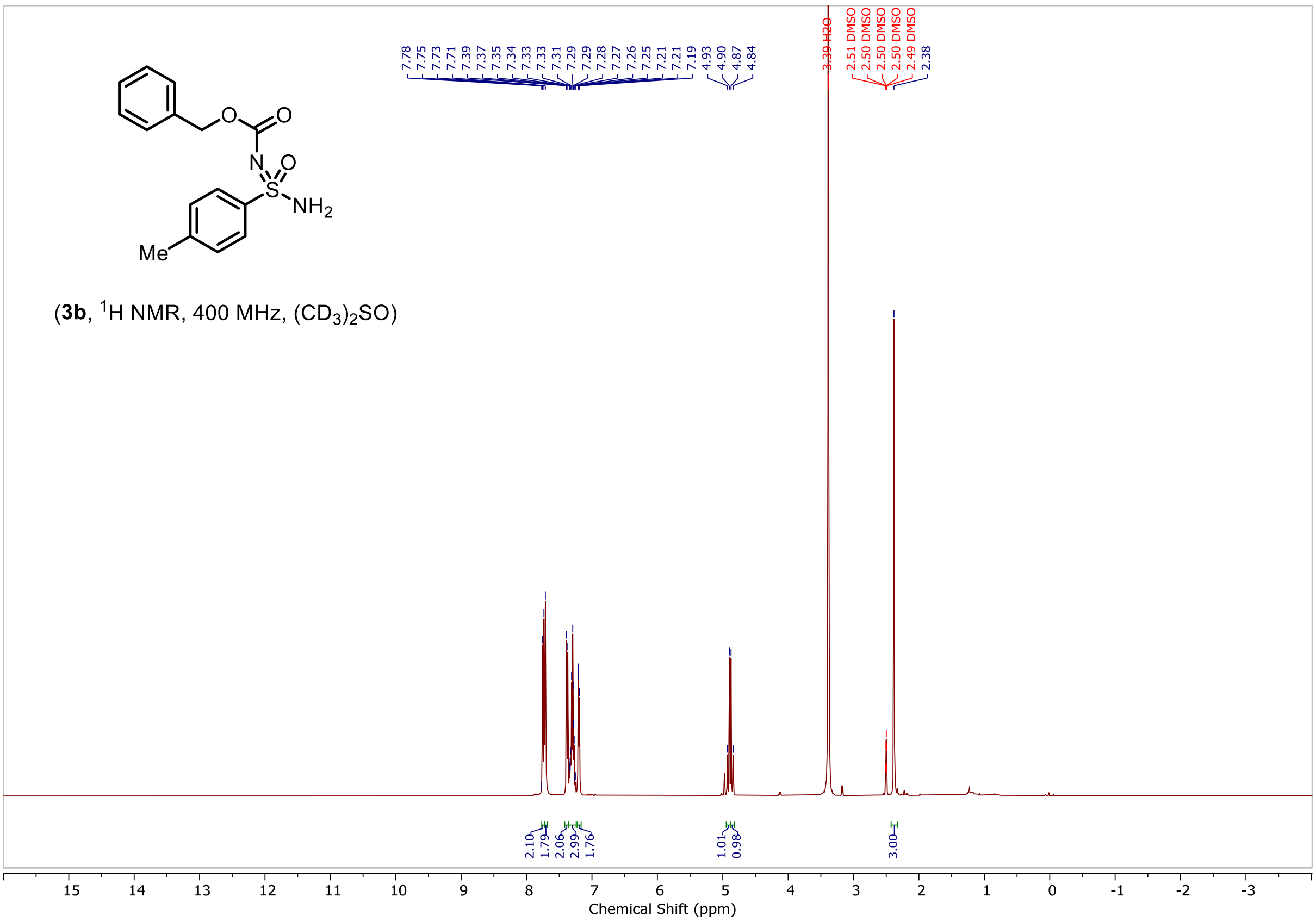
(3b, 1H NMR, 400 MHz, (CD3)2SO)
7.78
7.75
7.73
7.71
7.39
7.37
7.35
7.34
7.33
7.33
7.31
7.29
7.29
7.28
7.27
7.26
7.25
7.21
7.21
7.19
4.93
4.90
4.87
4.84
3.39 H2O
2.51 DMSO
2.50 DMSO
2.50 DMSO
2.50 DMSO
2.49 DMSO
2.38
2.10
1.79
2.06
2.99
1.76
1.01
0.98
3.00
NH2
Me
Chemical Shift (ppm)

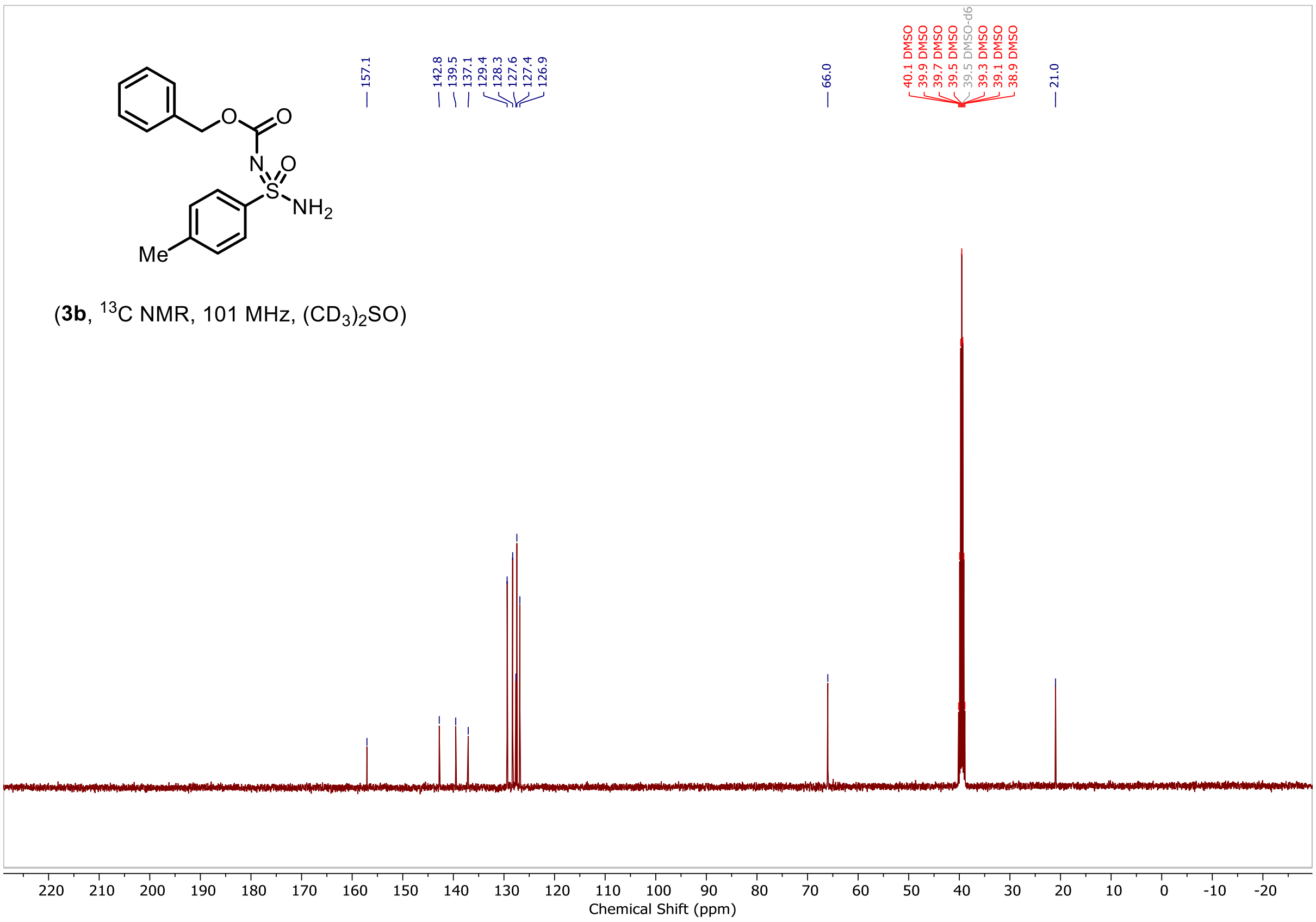

(**3b**, ^{13}C NMR, 101 MHz, $(CD_3)_2SO$)

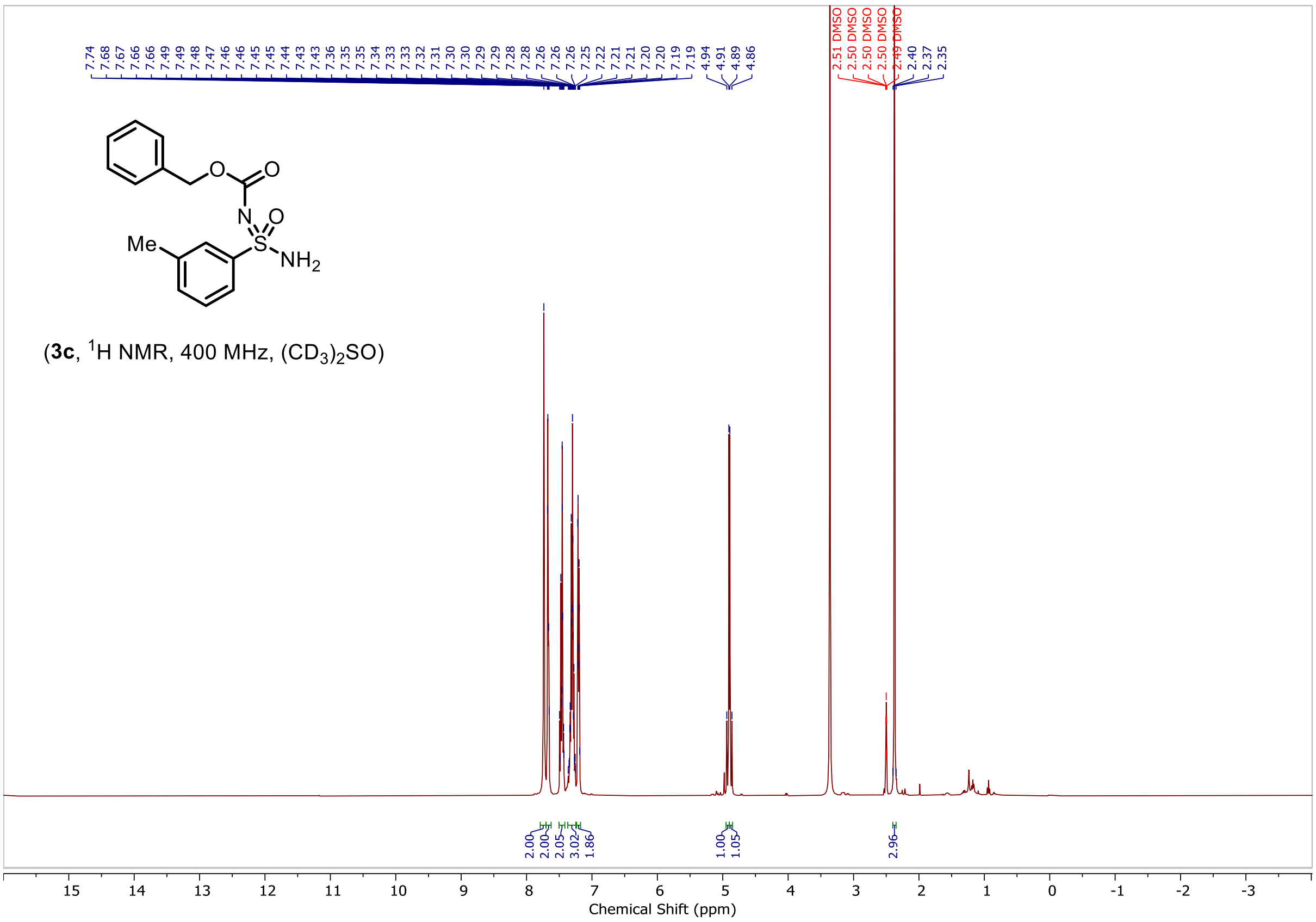

(**3c**, ^{1}H NMR, 400 MHz, $(CD_3)_2SO$)

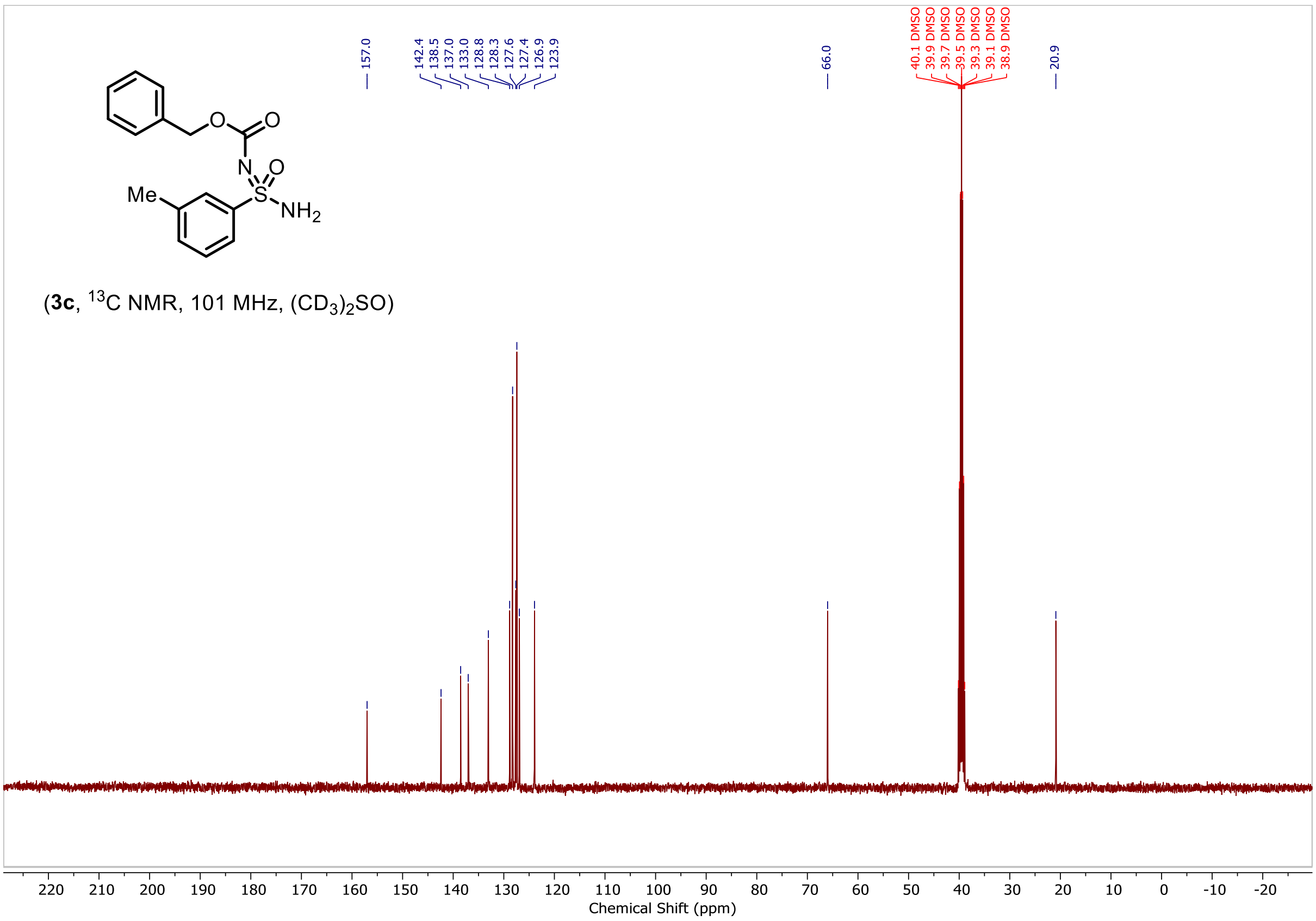

(**3c**, ^{13}C NMR, 101 MHz, $(CD_3)_2SO$)

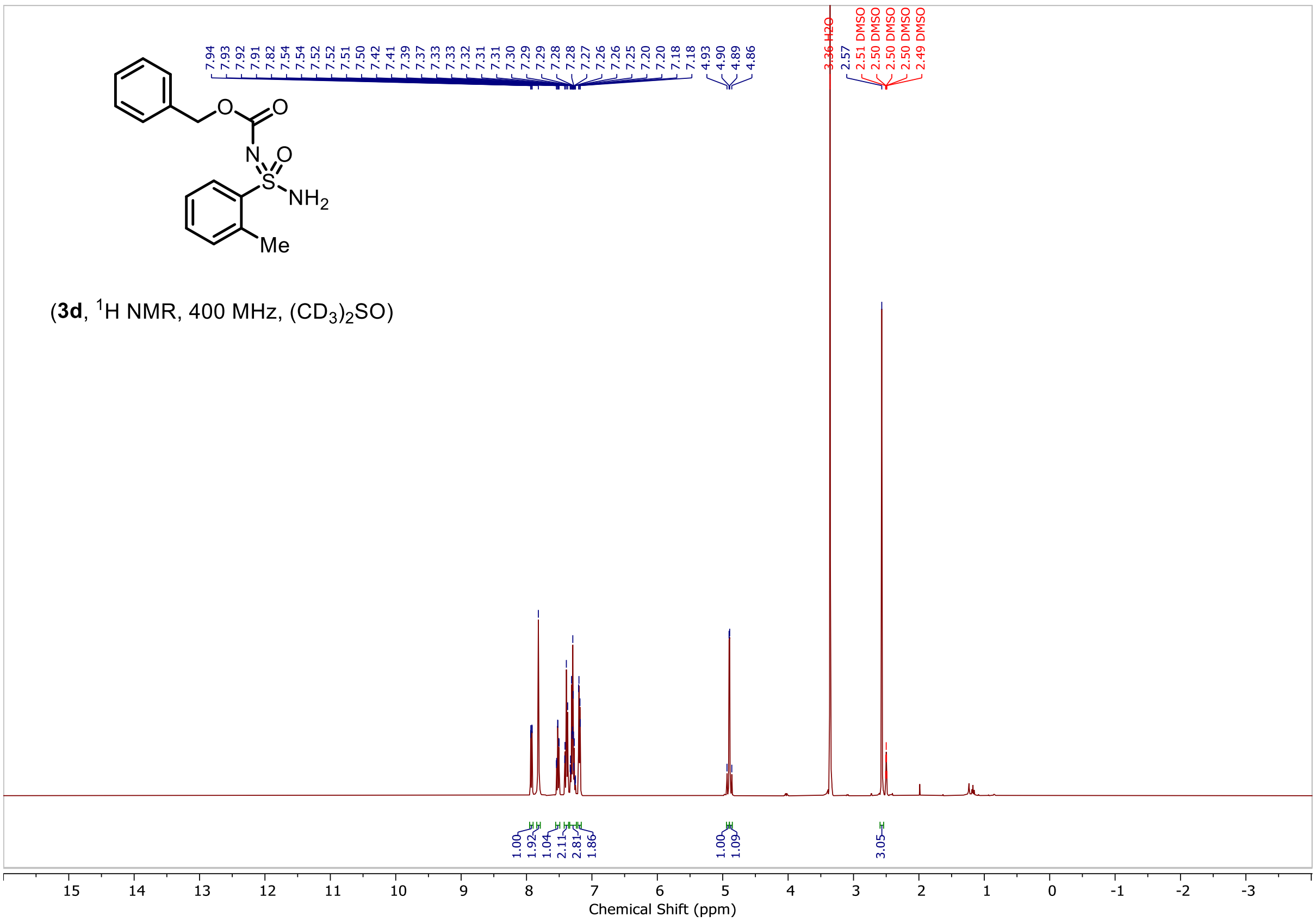

(**3d**, ^{1}H NMR, 400 MHz, $(CD_3)_2SO$)

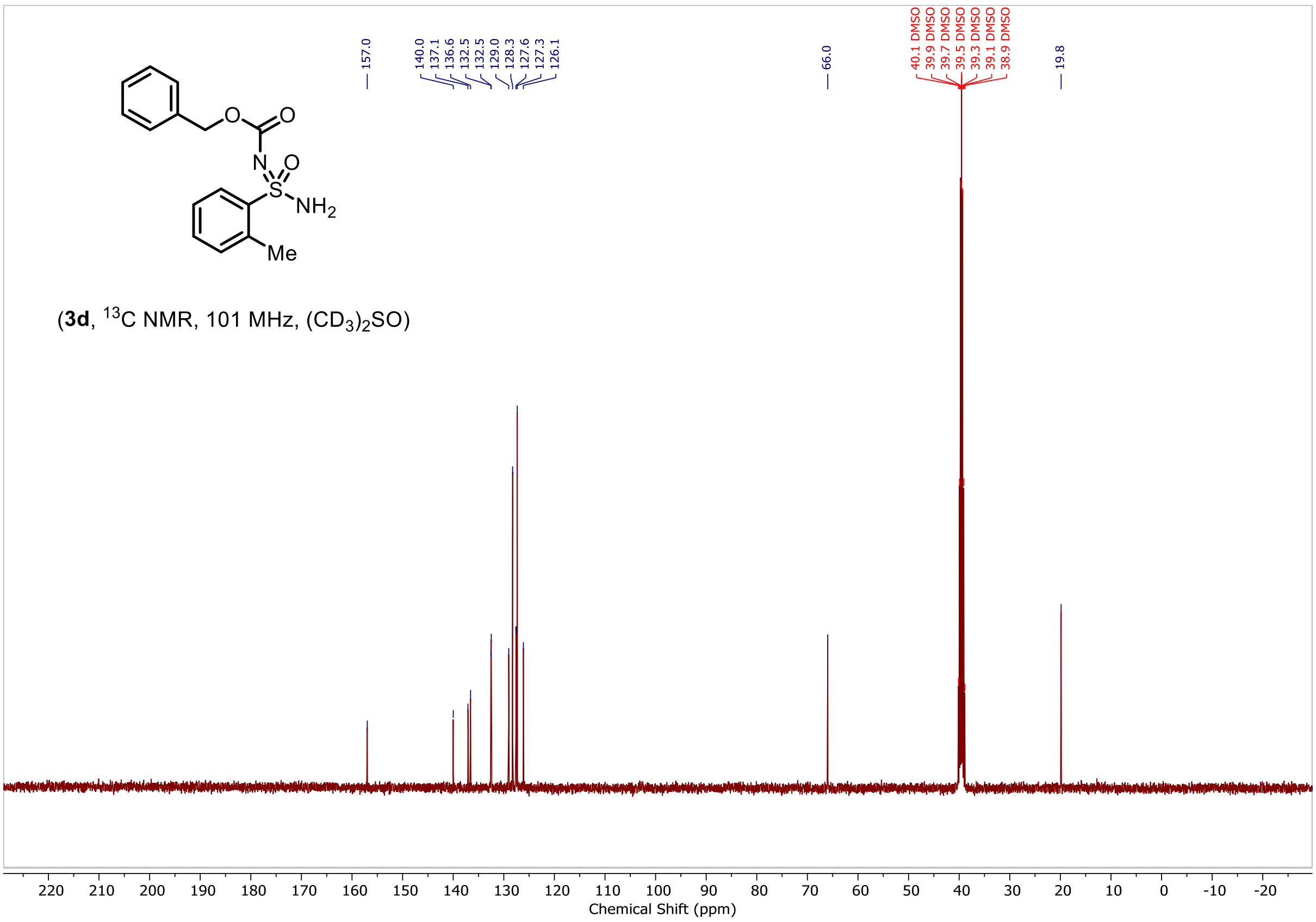

(**3d**, ^{13}C NMR, 101 MHz, $(CD_3)_2SO$)

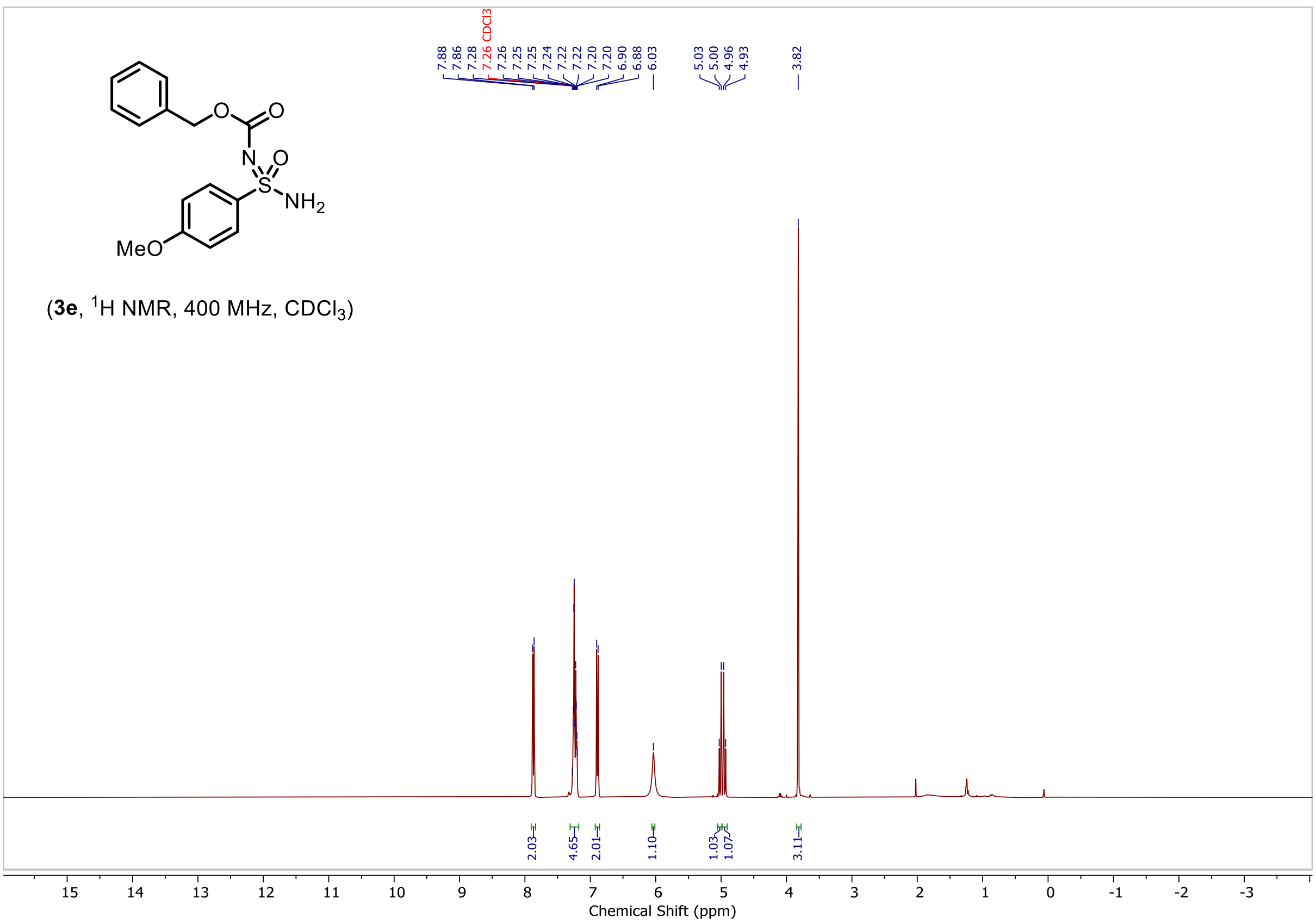

(**3e**, ^{1}H NMR, 400 MHz, $CDCl_3$)

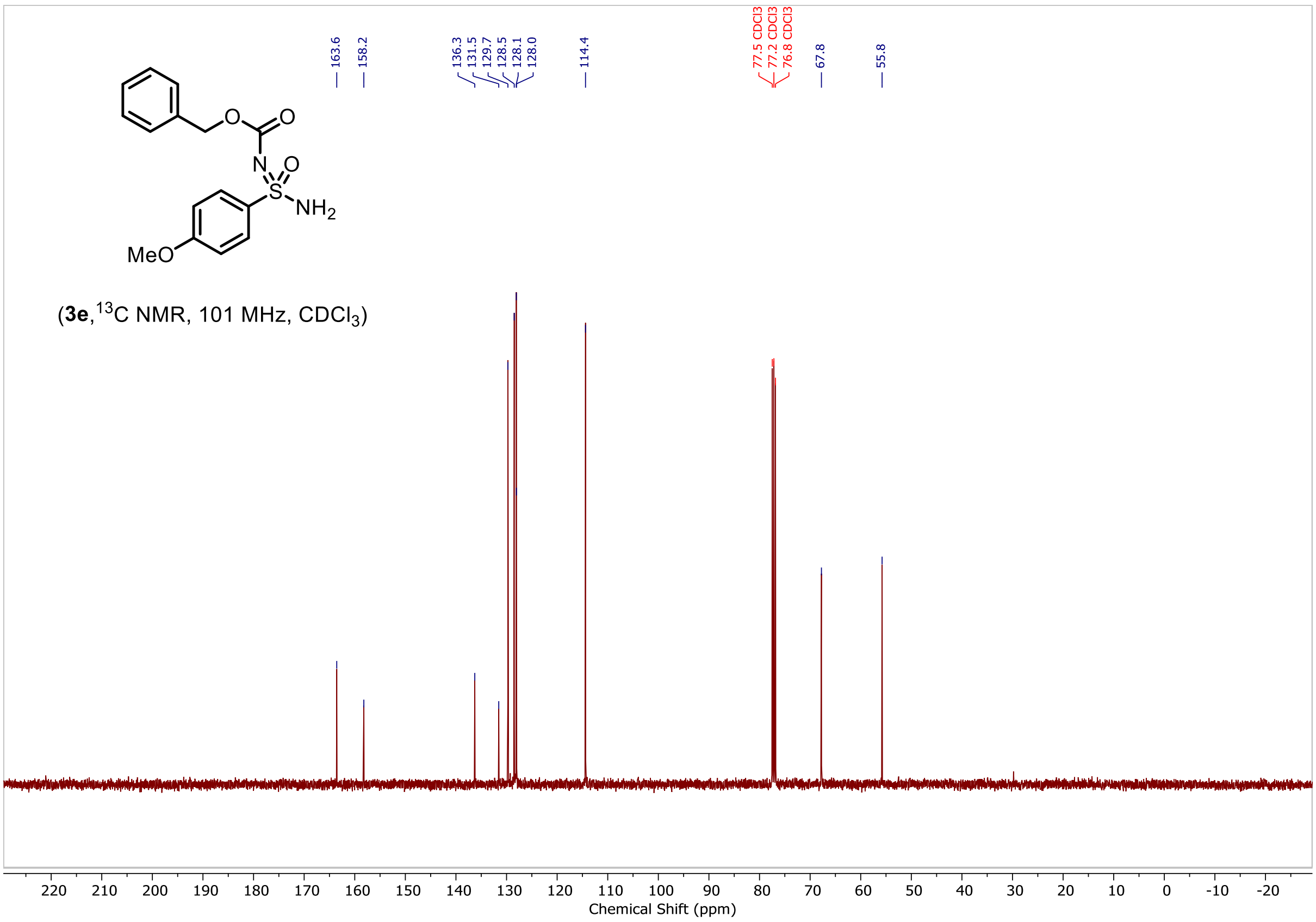

(**3e**, ^{13}C NMR, 101 MHz, $CDCl_3$)

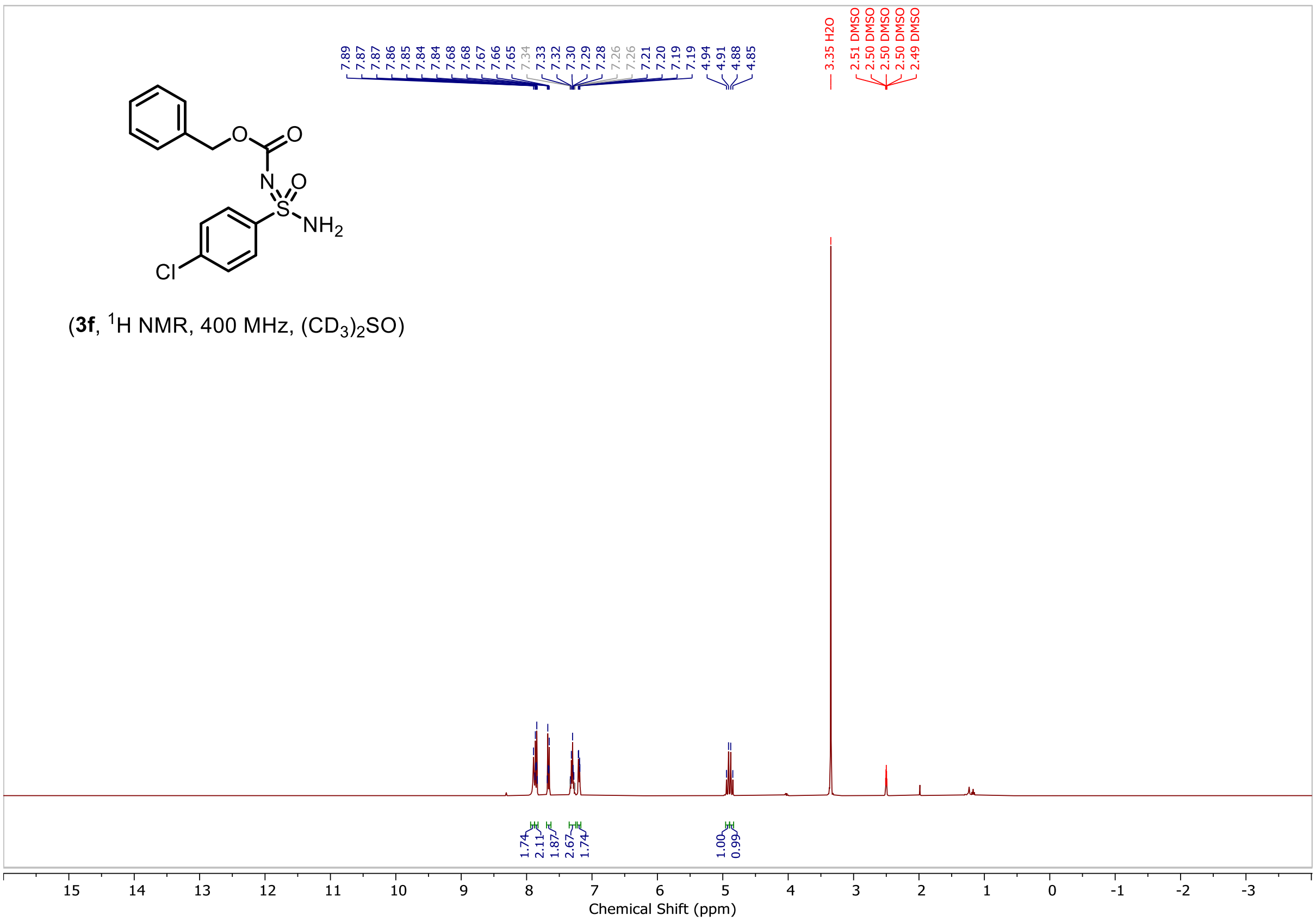

(**3f**, ^{1}H NMR, 400 MHz, $(CD_3)_2SO$)

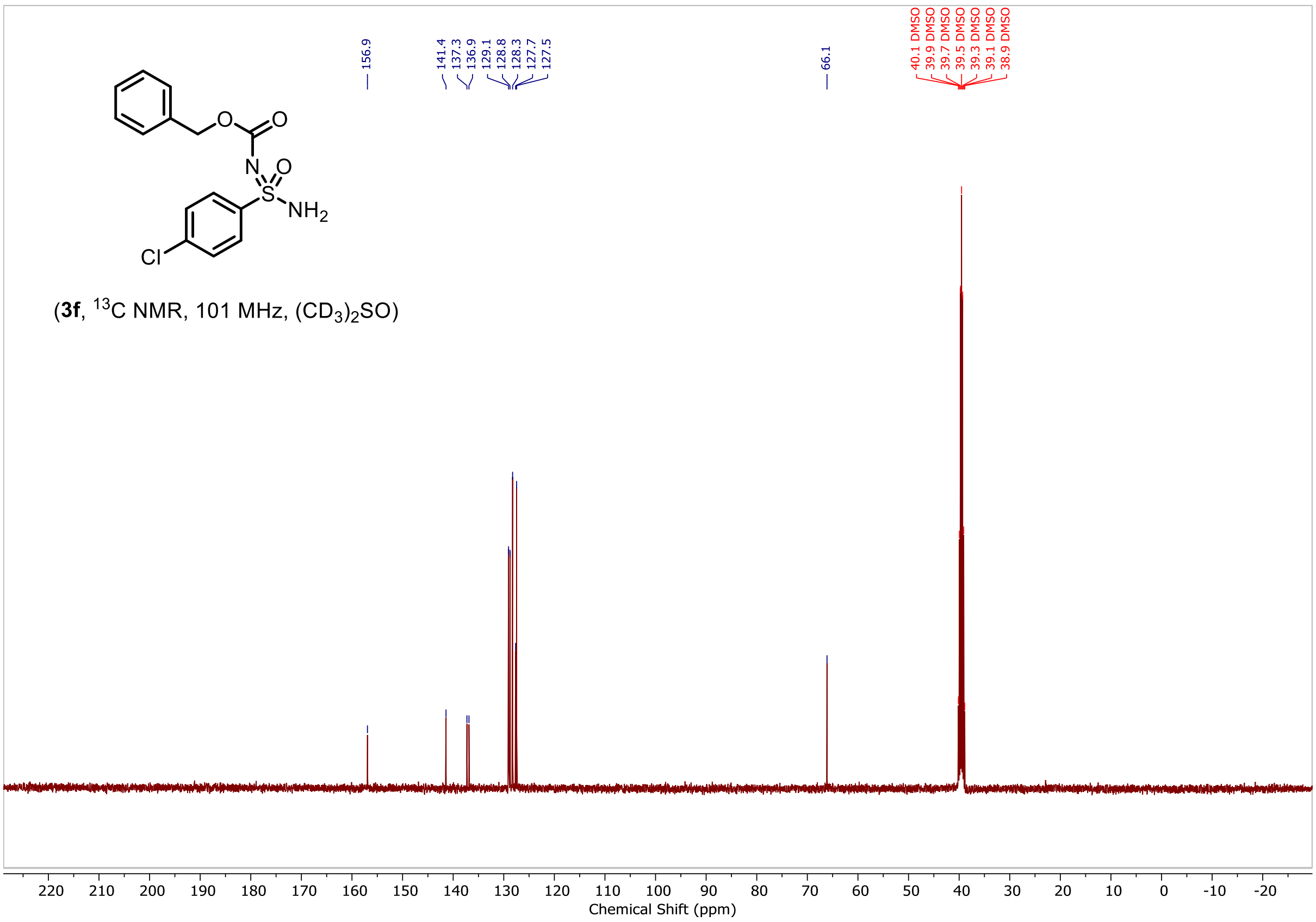

(3f, 13C NMR, 101 MHz, (CD3)2SO)
156.9
141.4
137.3
136.9
129.1
128.8
128.3
127.7
127.5
66.1
40.1 DMSO
39.9 DMSO
39.7 DMSO
39.5 DMSO
39.3 DMSO
39.1 DMSO
38.9 DMSO
Chemical Shift (ppm)

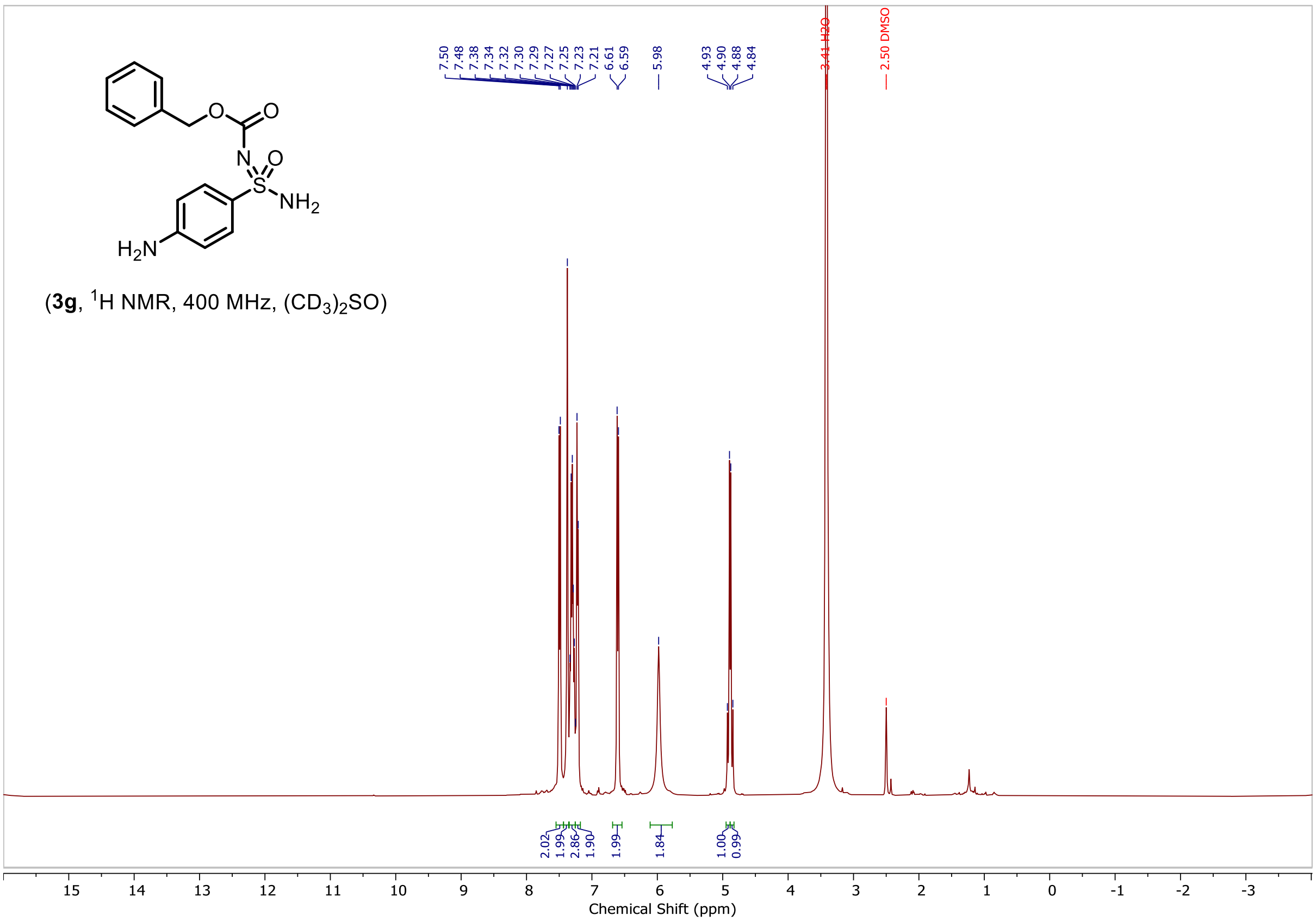

(3g, 1H NMR, 400 MHz, (CD3)2SO)
7.50
7.48
7.38
7.34
7.32
7.30
7.29
7.27
7.25
7.23
7.21
6.61
6.59
5.98
4.93
4.90
4.88
4.84
3.41 H2O
2.50 DMSO
2.02
1.99
2.86
1.90
1.99
1.84
1.00
0.99
Chemical Shift (ppm)

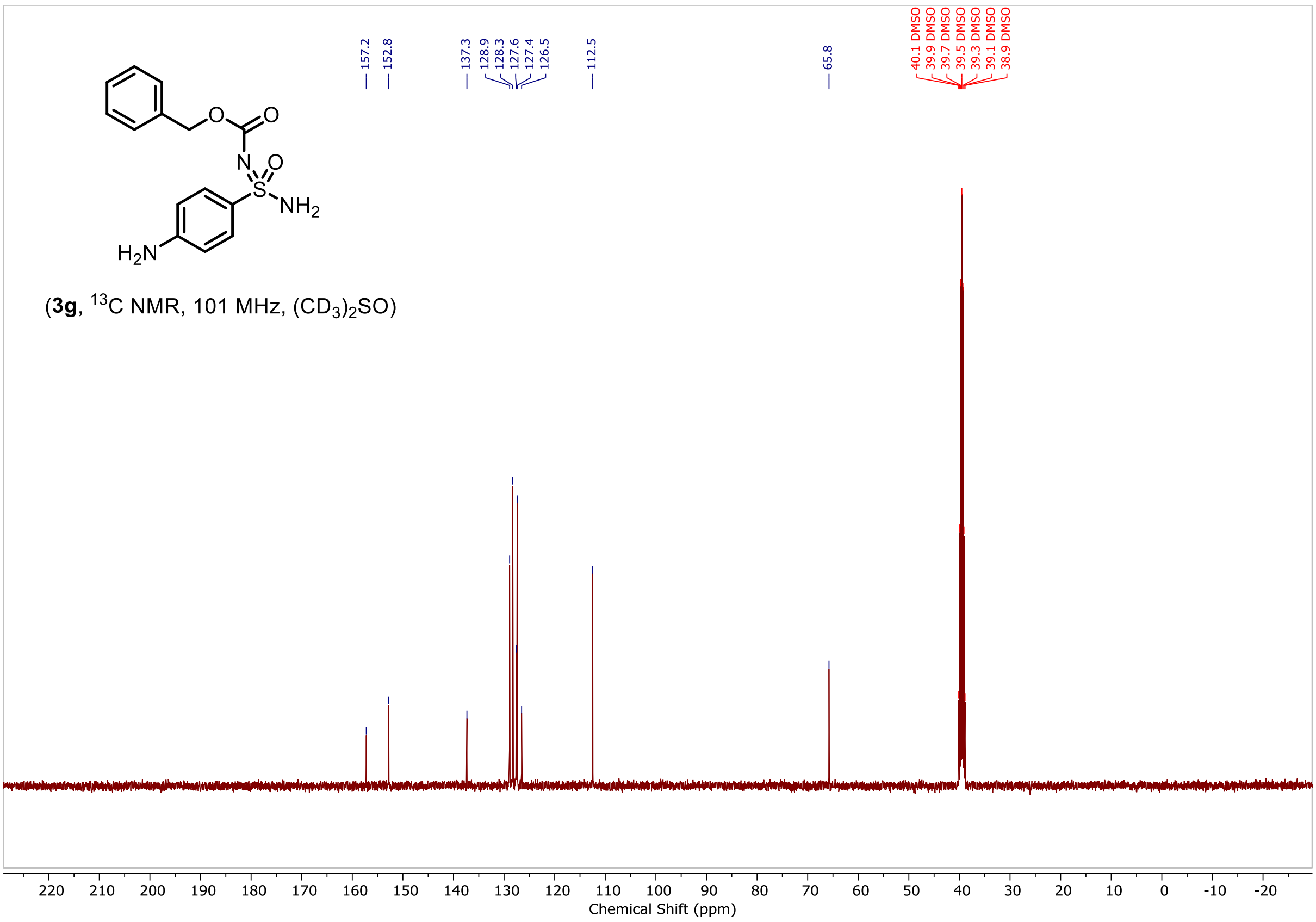

(**3g**, ^{13}C NMR, 101 MHz, $(CD_3)_2SO$)

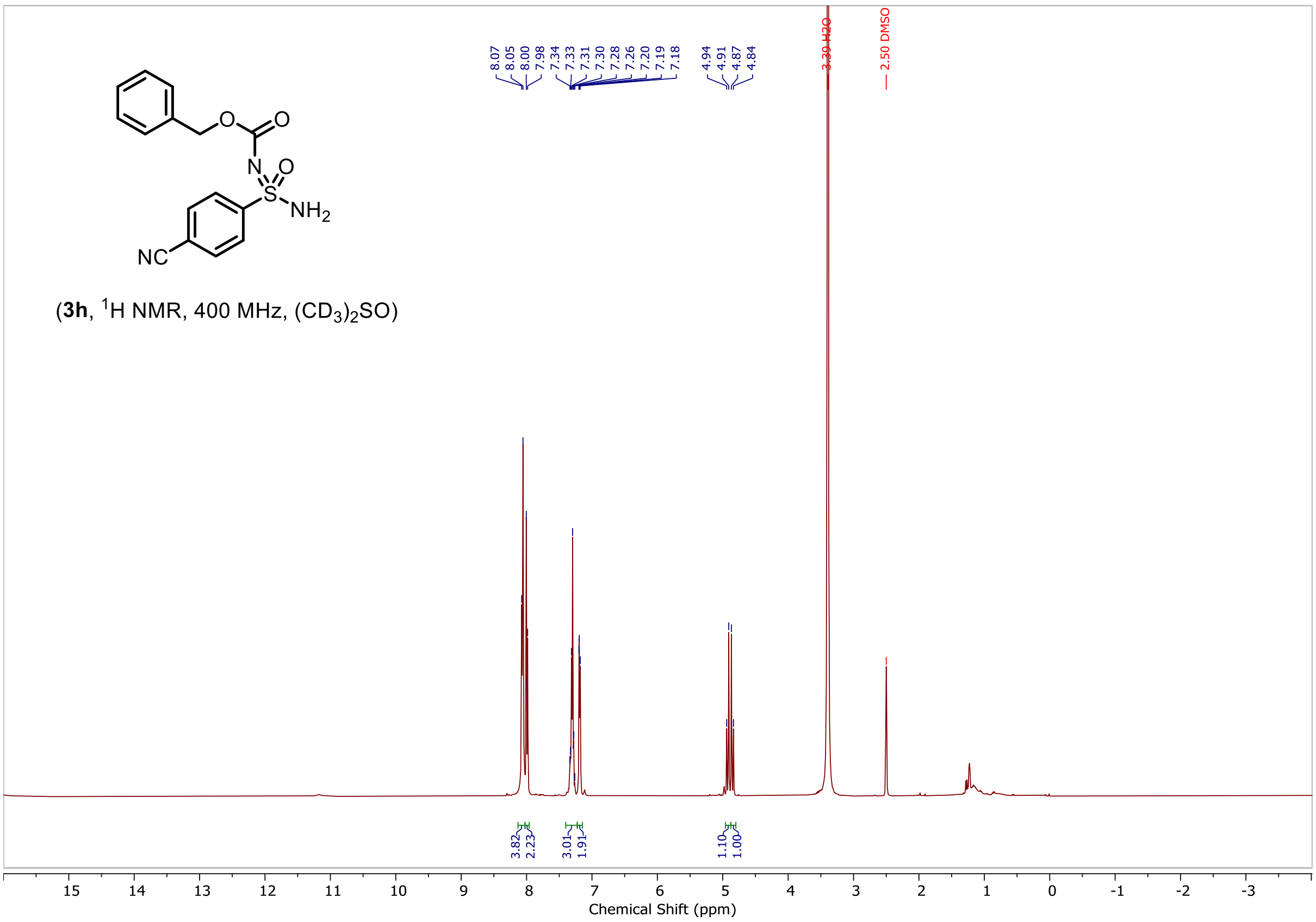

(**3h**, ^{1}H NMR, 400 MHz, $(CD_3)_2SO$)

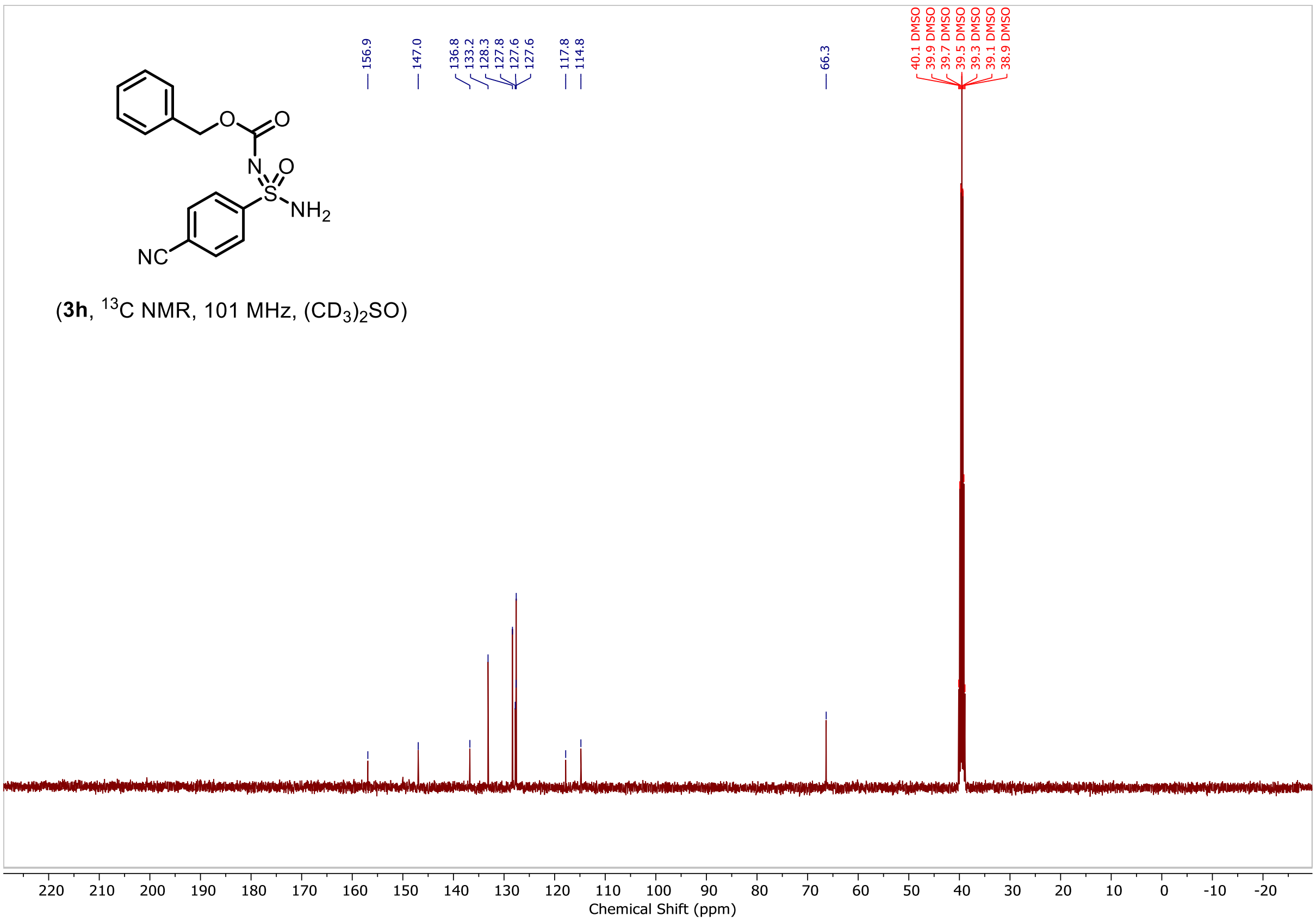

(**3h**, ^{13}C NMR, 101 MHz, $(CD_3)_2SO$)

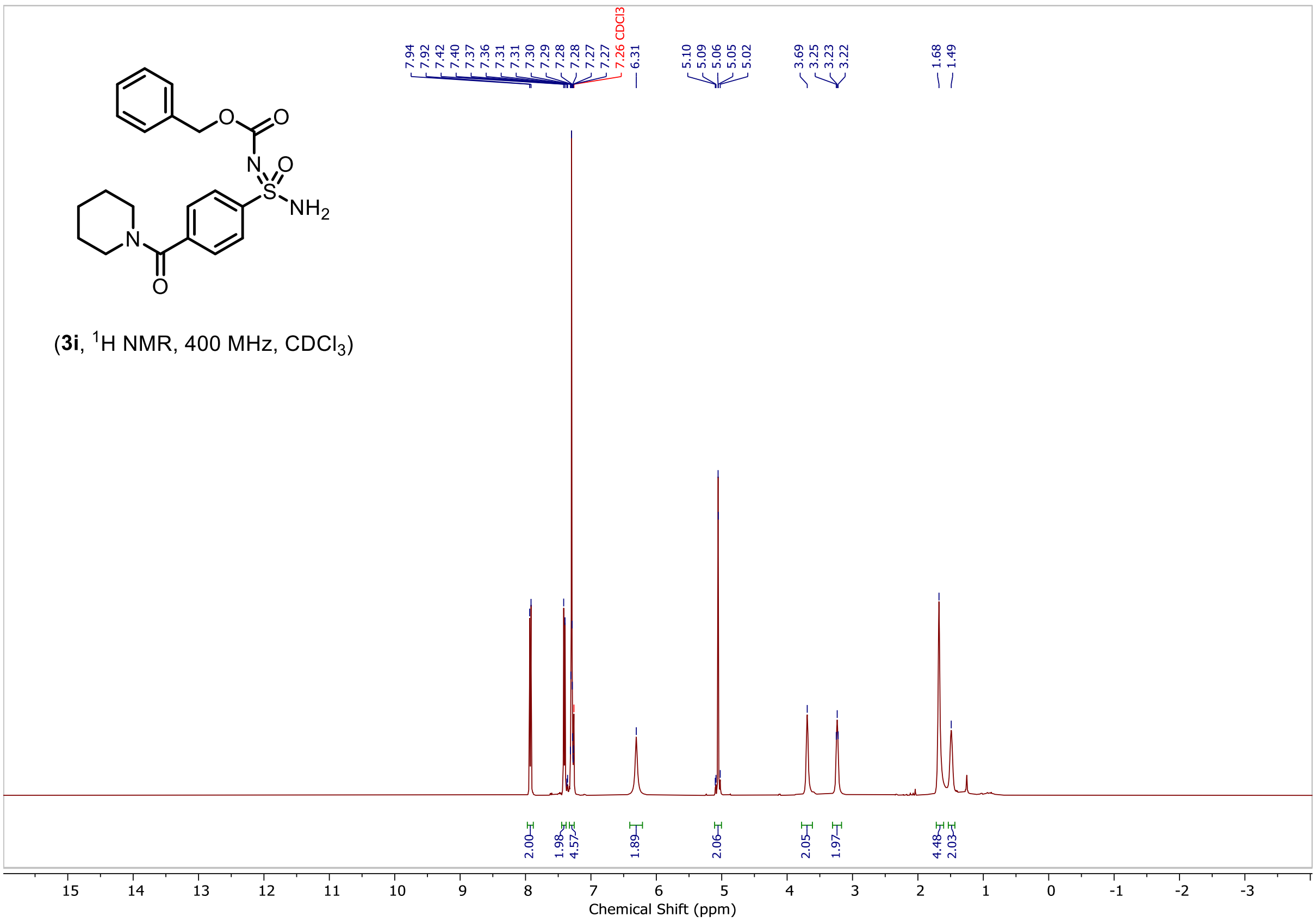

(**3i**, ^{1}H NMR, 400 MHz, $CDCl_3$)

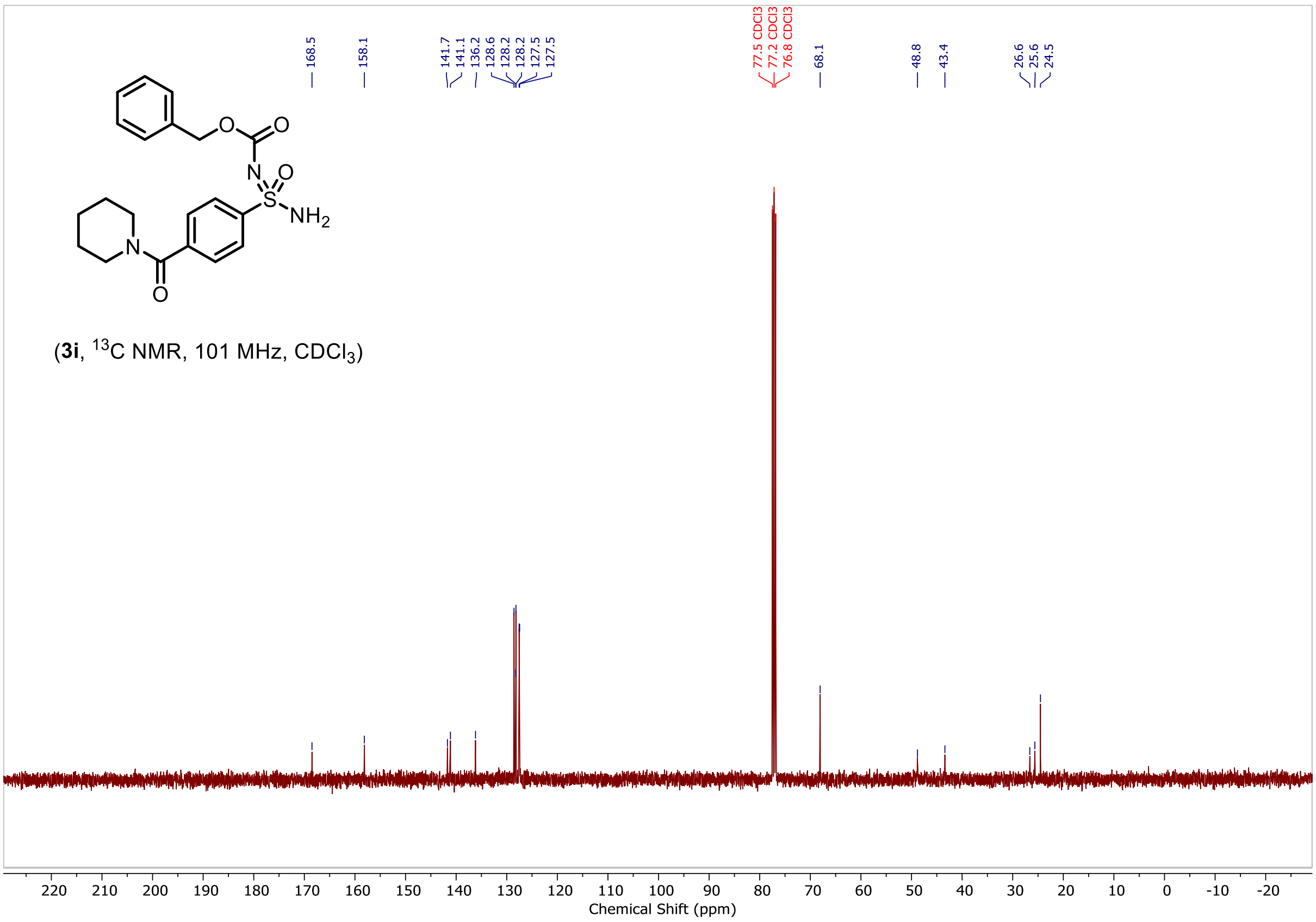
168.5
158.1
141.7
141.1
136.2
128.6
128.2
128.2
127.5
127.5
77.5 CDCl3
77.2 CDCl3
76.8 CDCl3
68.1
48.8
43.4
26.6
25.6
24.5
O
O
N
O
S
NH2
N
O
(3i, 13C NMR, 101 MHz, CDCl3)
Chemical Shift (ppm)

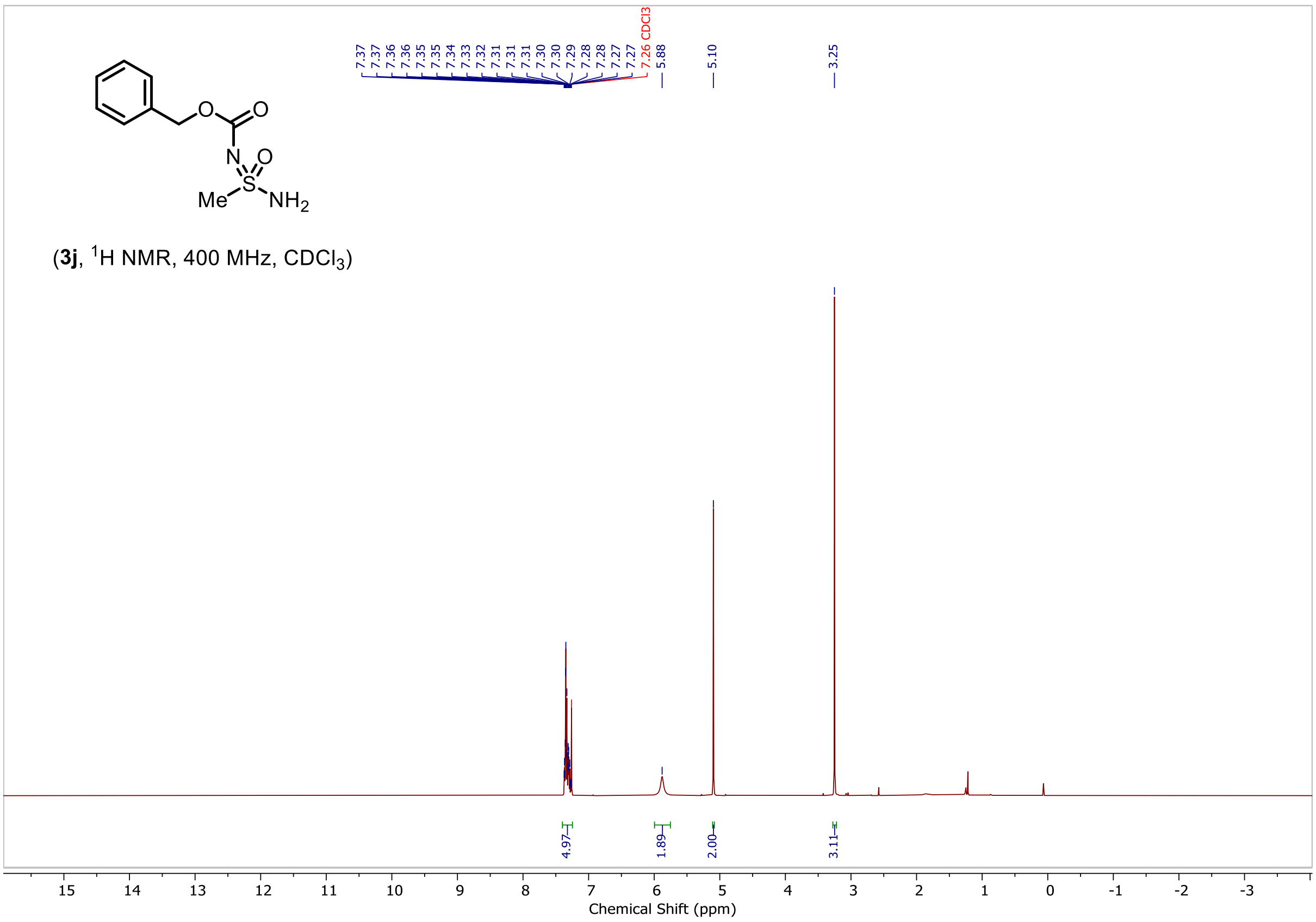
7.37
7.37
7.36
7.36
7.35
7.35
7.34
7.33
7.32
7.31
7.31
7.31
7.30
7.30
7.29
7.28
7.28
7.27
7.27
7.26 CDCl3
5.88
5.10
3.25
O
O
N
O
S
Me
NH2
(3j, 1H NMR, 400 MHz, CDCl3)
4.97
1.89
2.00
3.11
Chemical Shift (ppm)

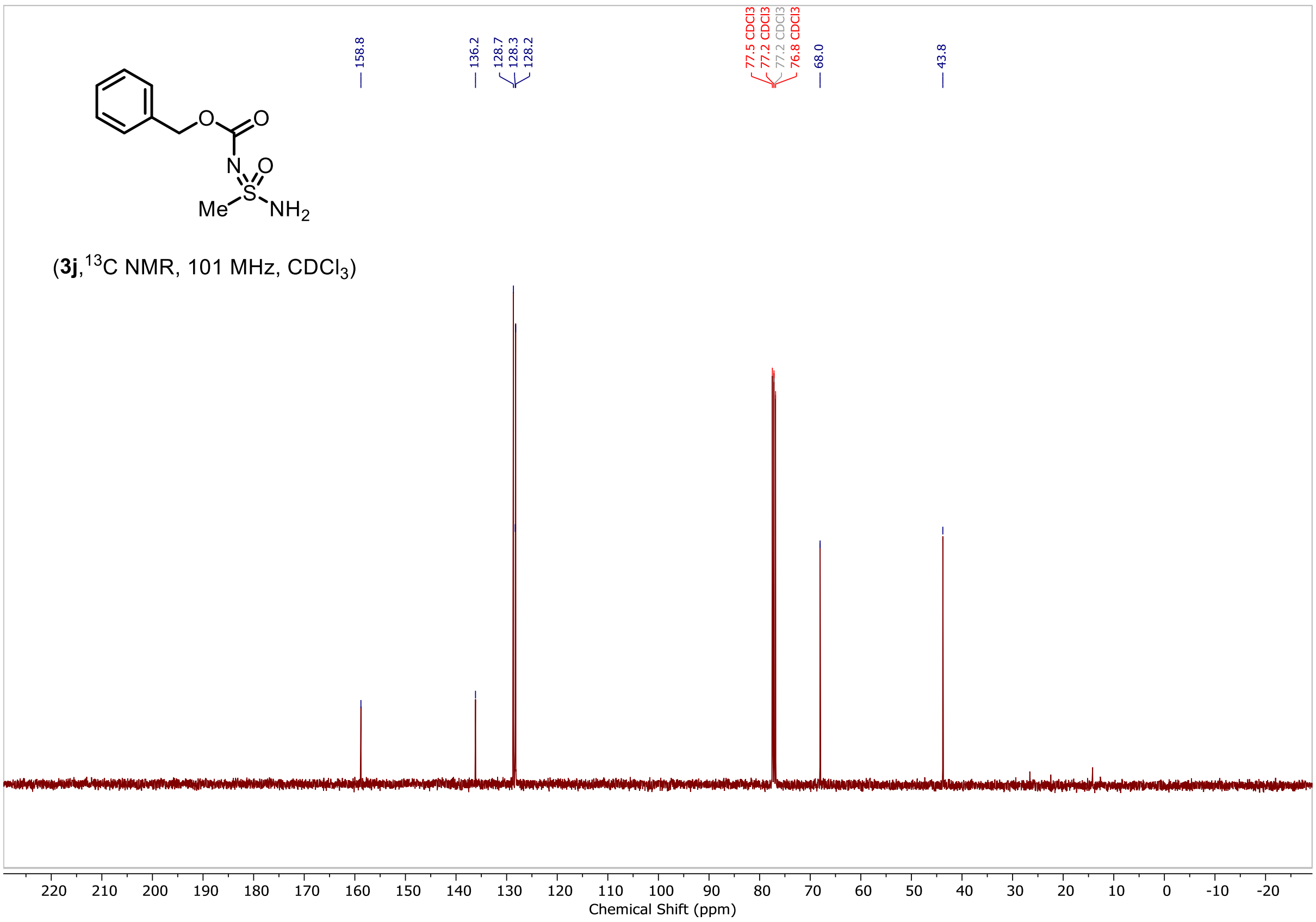

(**3j**, ^{13}C NMR, 101 MHz, $CDCl_3$)

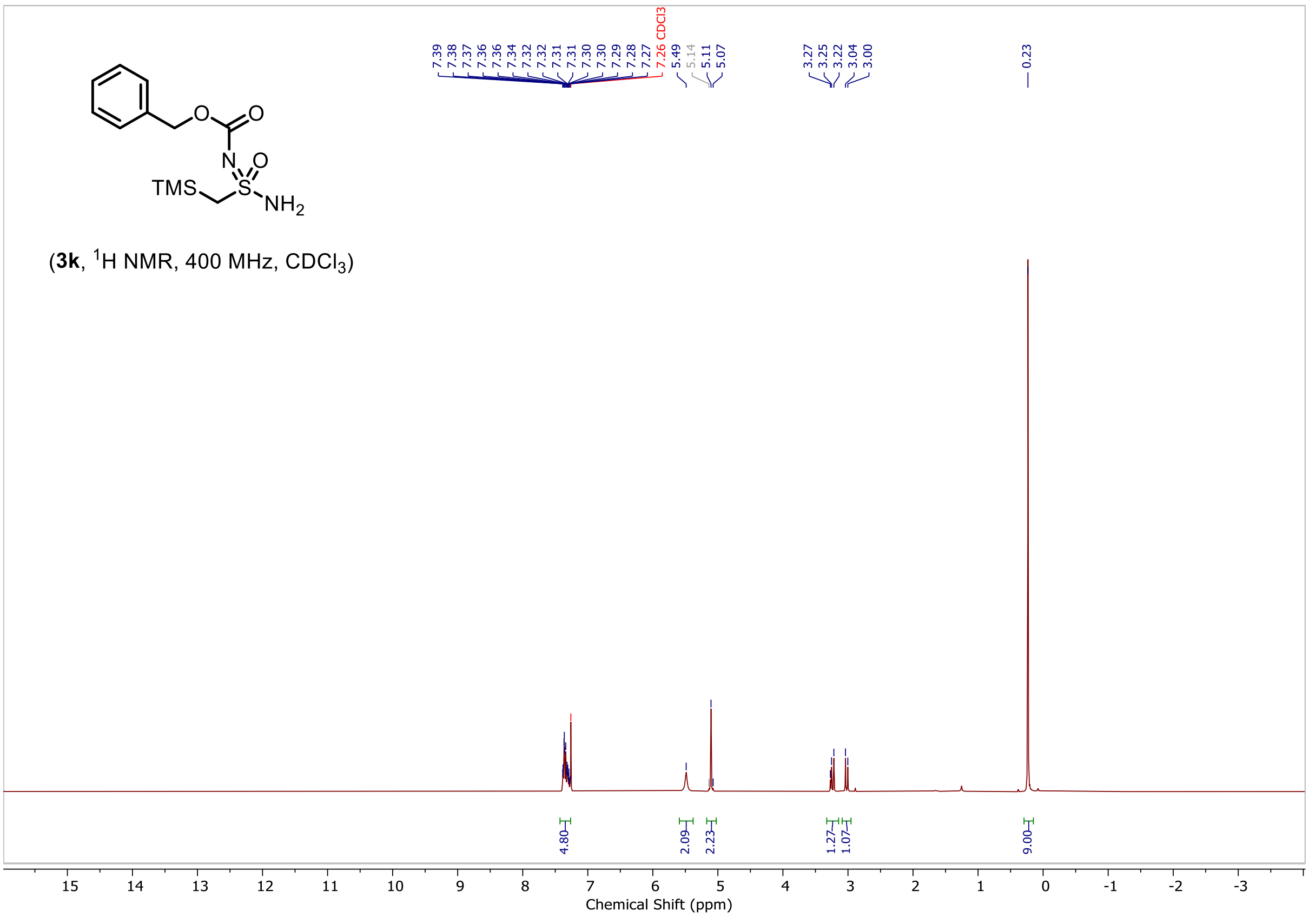

(**3k**, ^{1}H NMR, 400 MHz, $CDCl_3$)

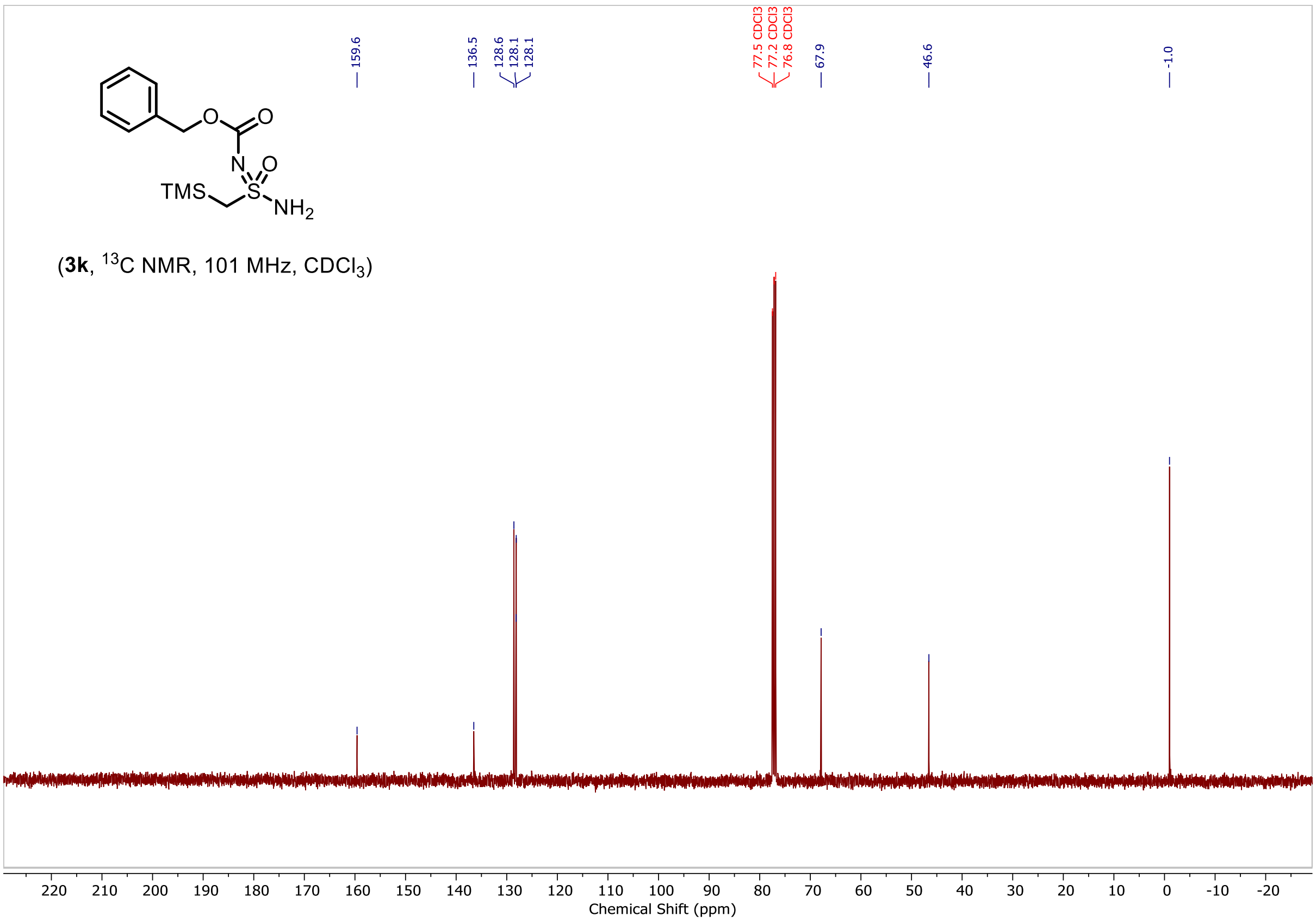
159.6
136.5
128.6
128.1
128.1
77.5 CDCl3
77.2 CDCl3
76.8 CDCl3
67.9
46.6
-1.0
TMS
N
O
O
O
S
NH2
(3k, 13C NMR, 101 MHz, CDCl3)
Chemical Shift (ppm)

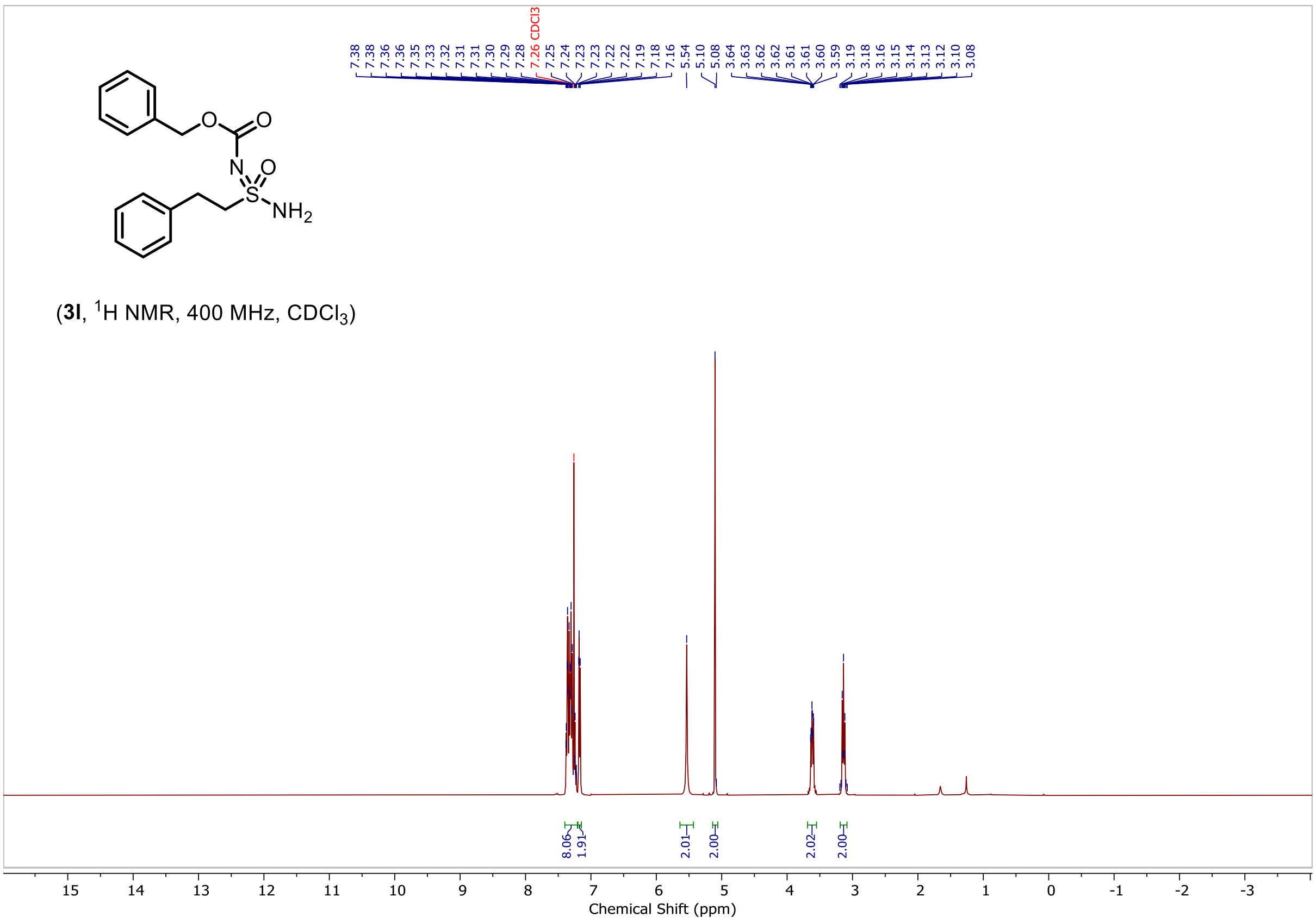
7.38
7.38
7.36
7.36
7.35
7.33
7.32
7.31
7.31
7.30
7.29
7.28
7.26 CDCl3
7.25
7.24
7.23
7.23
7.22
7.22
7.19
7.18
7.16
5.54
5.10
5.08
3.64
3.63
3.62
3.61
3.60
3.59
3.19
3.18
3.16
3.15
3.14
3.13
3.12
3.10
3.08
N
O
O
O
S
NH2
(3l, 1H NMR, 400 MHz, CDCl3)
8.06
1.91
2.01
2.00
2.02
2.00
Chemical Shift (ppm)

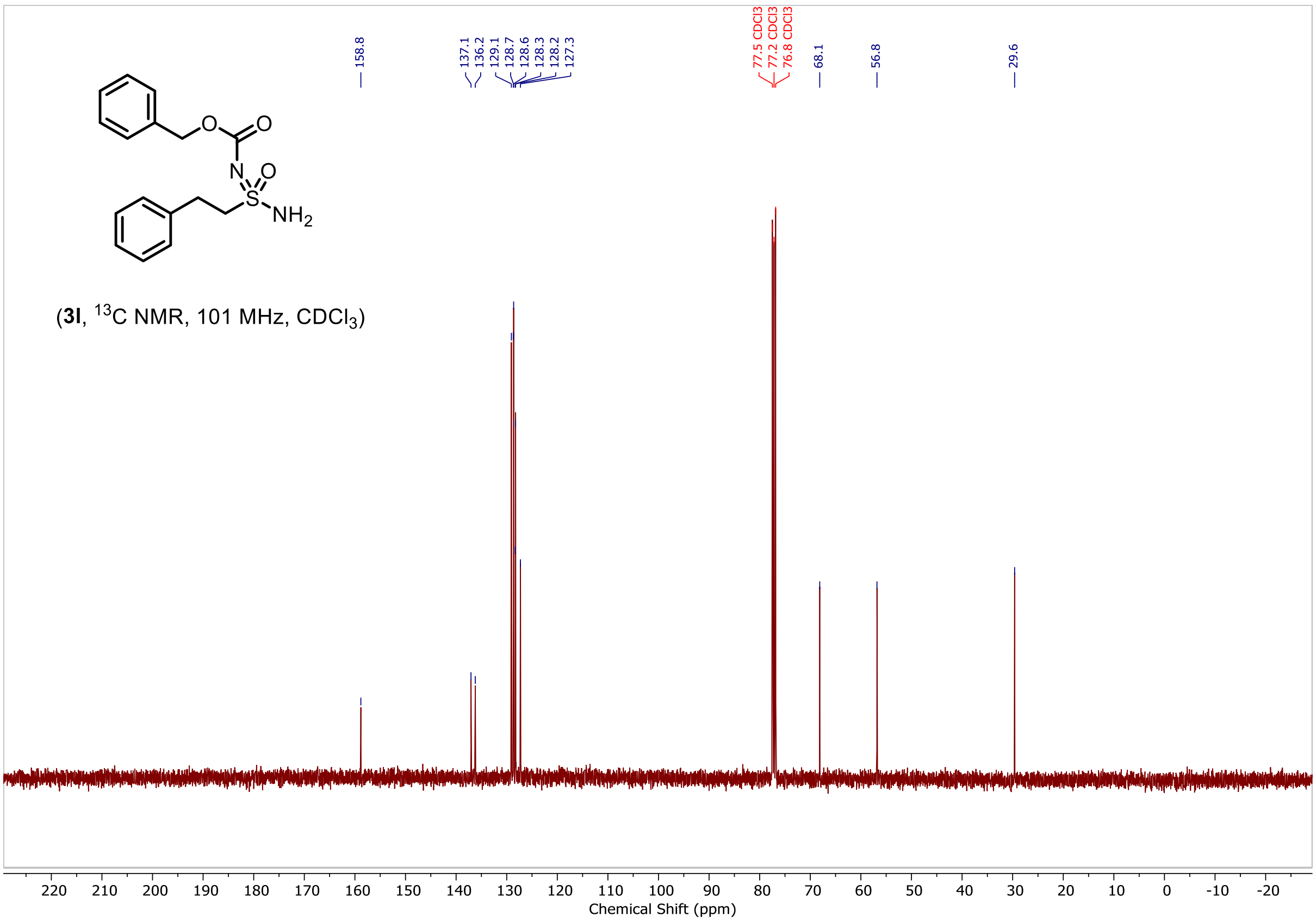

(**3l**, ^{13}C NMR, 101 MHz, $CDCl_3$)

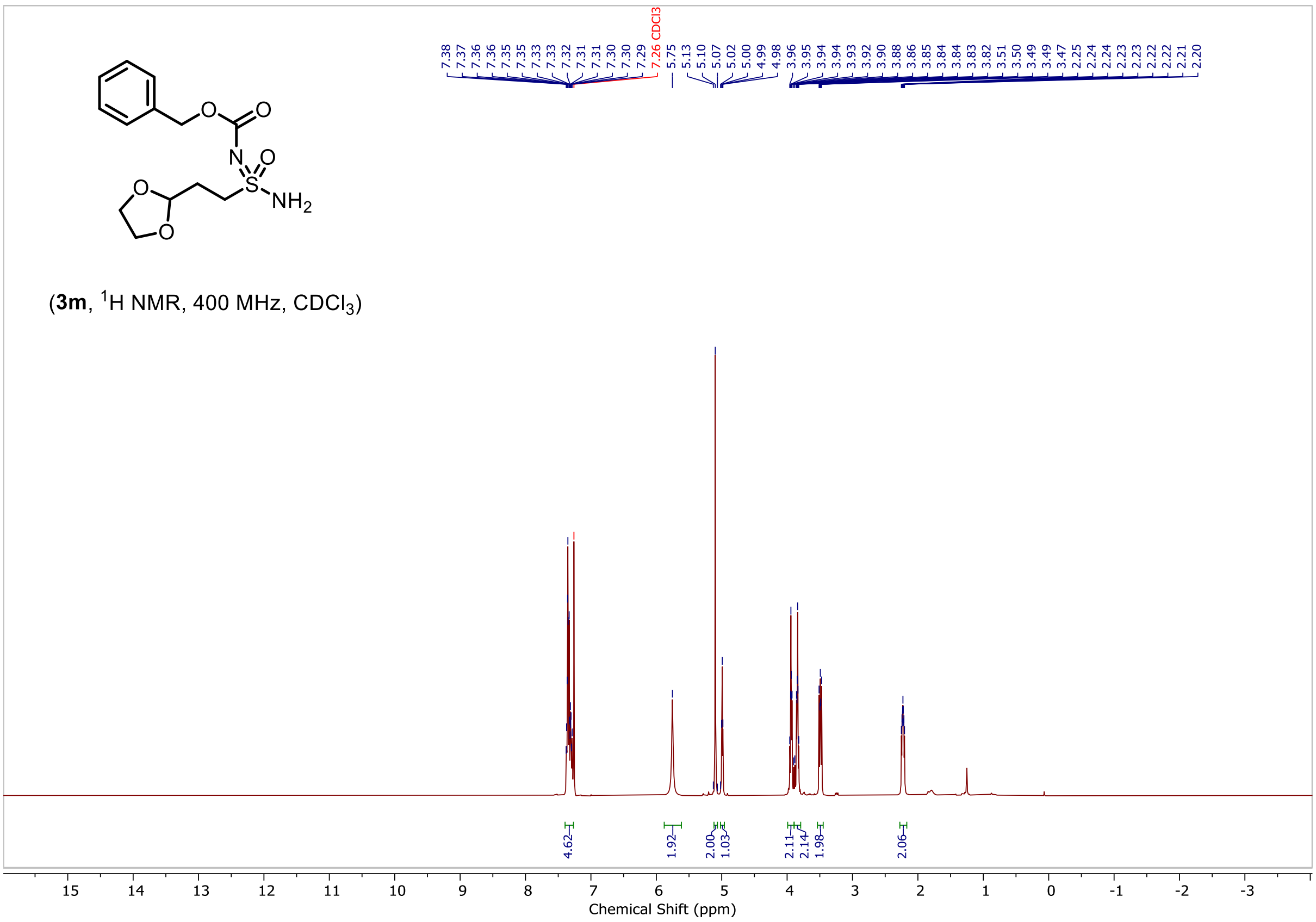

(**3m**, ^{1}H NMR, 400 MHz, $CDCl_3$)

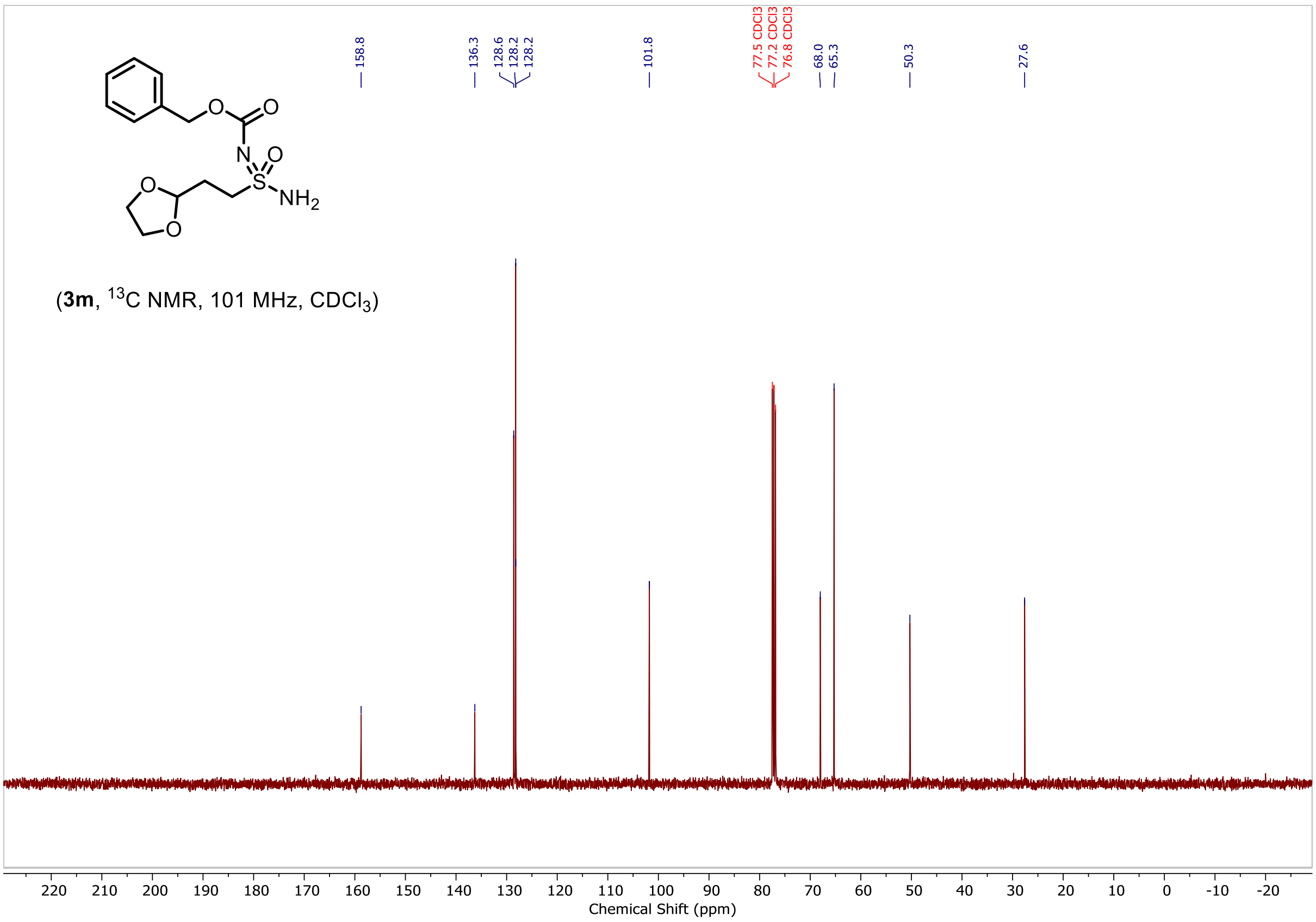

(3m, ^{13}C NMR, 101 MHz, CDCl$_3$)
158.8
136.3
128.6
128.2
128.2
101.8
77.5 CDCl3
77.2 CDCl3
76.8 CDCl3
68.0
65.3
50.3
27.6
Chemical Shift (ppm)

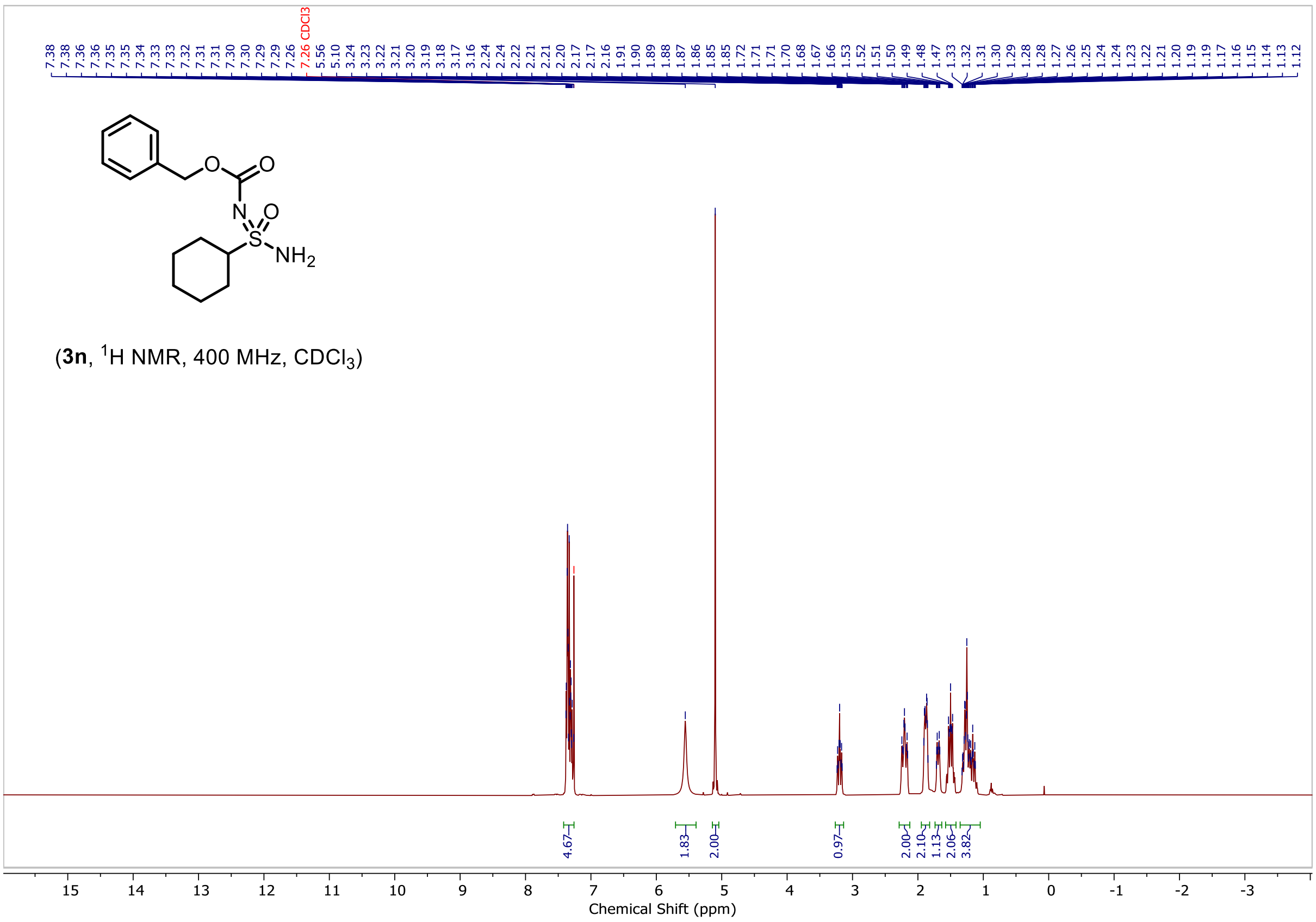

(3n, ^{1}H NMR, 400 MHz, CDCl$_3$)
7.38 7.38 7.36 7.35 7.35 7.34 7.33 7.33 7.32 7.31 7.31 7.30 7.30 7.29 7.29 7.26
7.26 CDCl3
5.56 5.10 3.24 3.23 3.22 3.21 3.20 3.19 3.18 3.17 3.16 2.24 2.24 2.22 2.21 2.21 2.20 2.17 2.17 2.16 1.91 1.90 1.89 1.88 1.87 1.86 1.85 1.85 1.72 1.71 1.71 1.70 1.68 1.67 1.66 1.53 1.52 1.51 1.50 1.49 1.48 1.47 1.33 1.32 1.31 1.30 1.29 1.28 1.28 1.27 1.26 1.25 1.24 1.24 1.23 1.22 1.21 1.20 1.19 1.19 1.17 1.16 1.15 1.14 1.13 1.12
4.67
1.83
2.00
0.97
2.00
2.10
1.13
2.06
3.82
Chemical Shift (ppm)

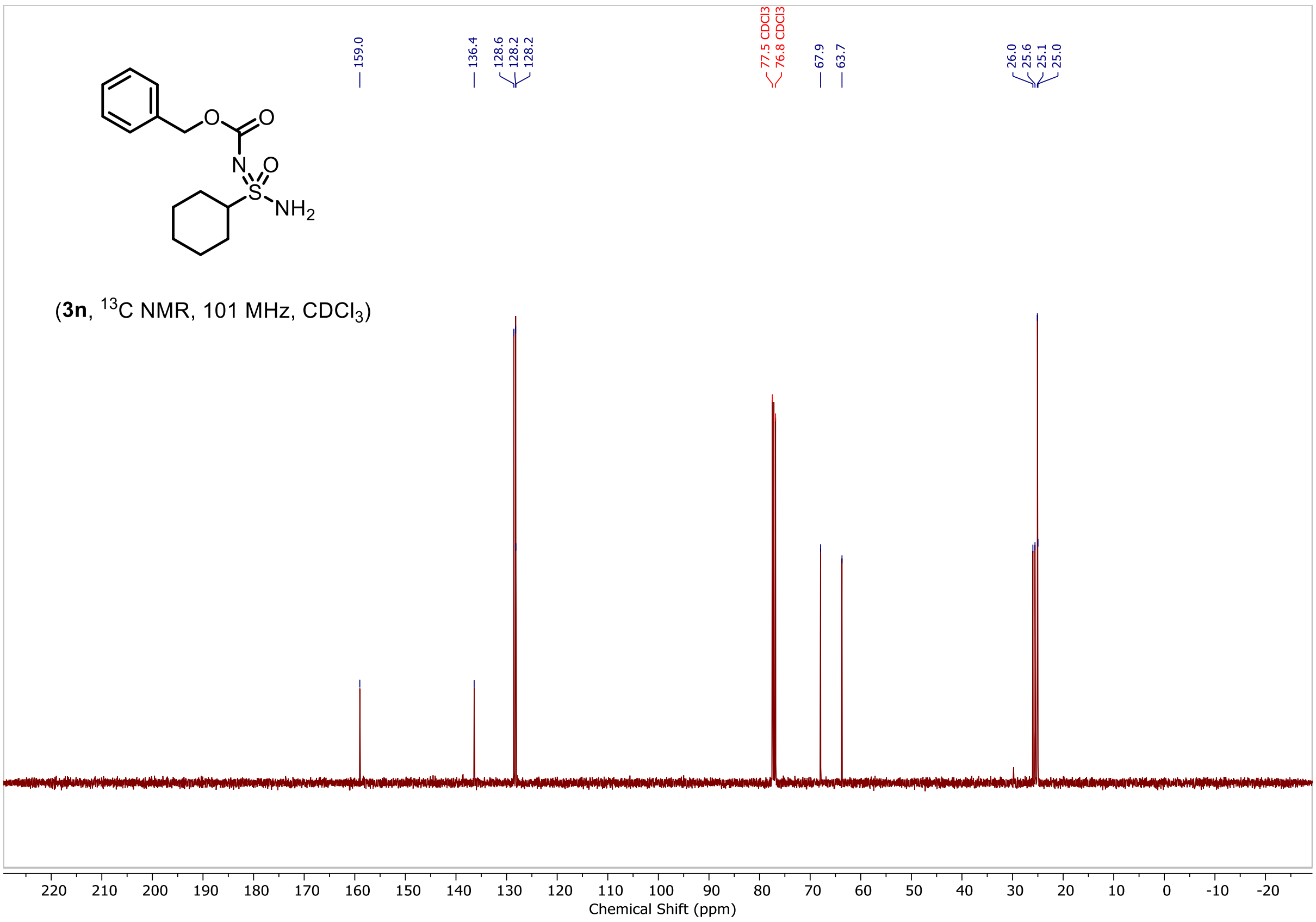

(**3n**, ^{13}C NMR, 101 MHz, $CDCl_3$)

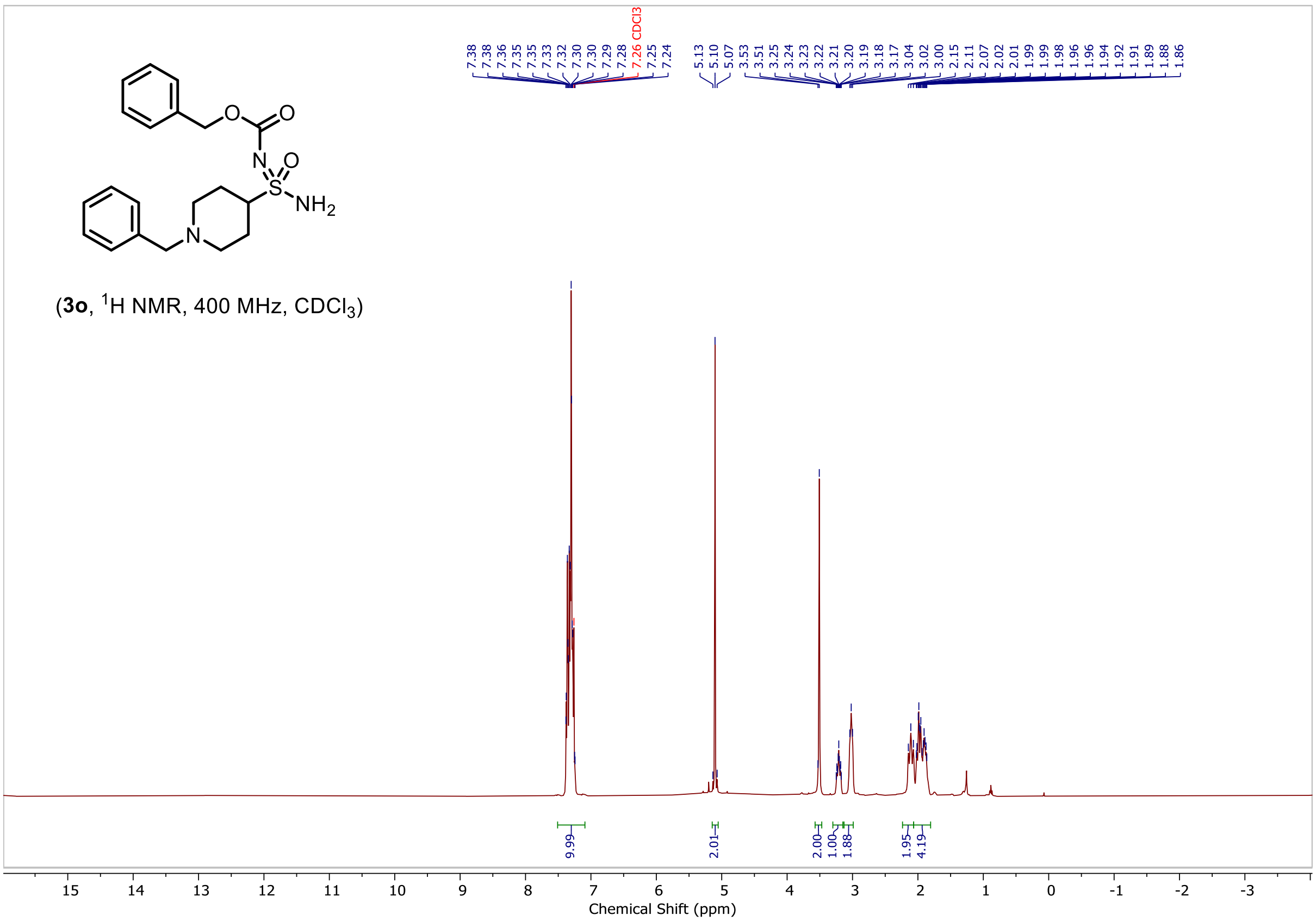

(**3o**, ^{1}H NMR, 400 MHz, $CDCl_3$)

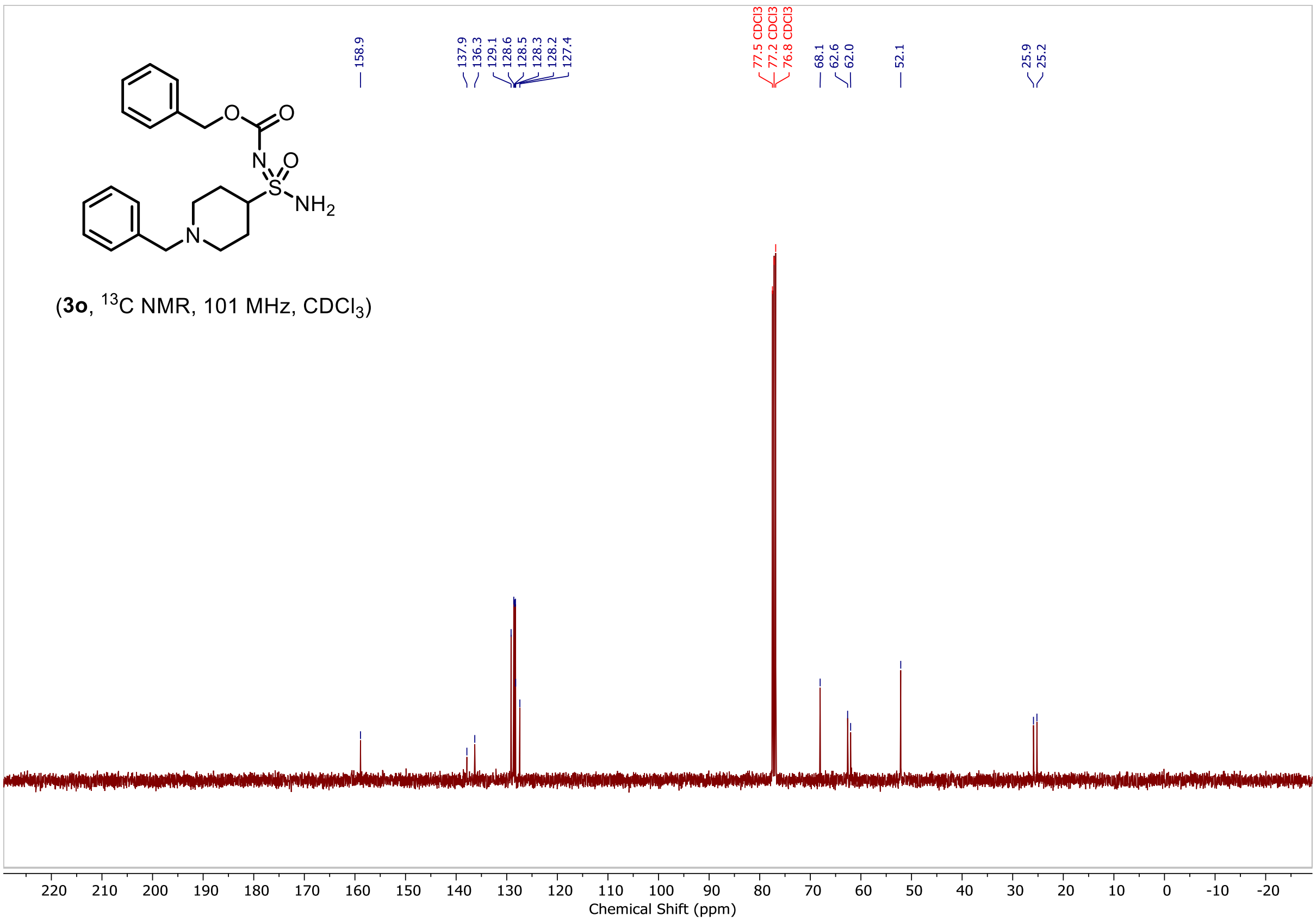

(**3o**, ^{13}C NMR, 101 MHz, $CDCl_3$)

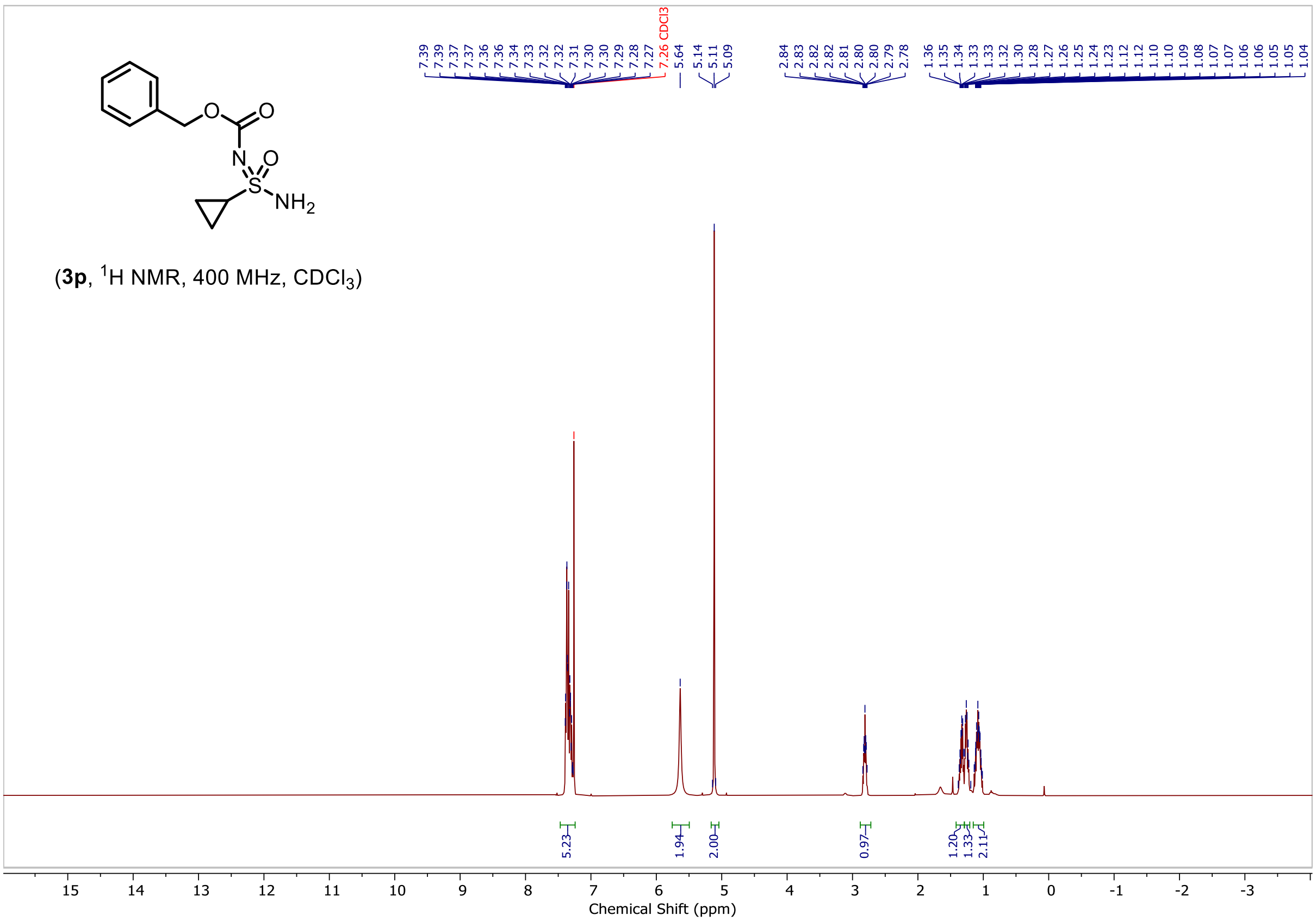

(**3p**, ^{1}H NMR, 400 MHz, $CDCl_3$)

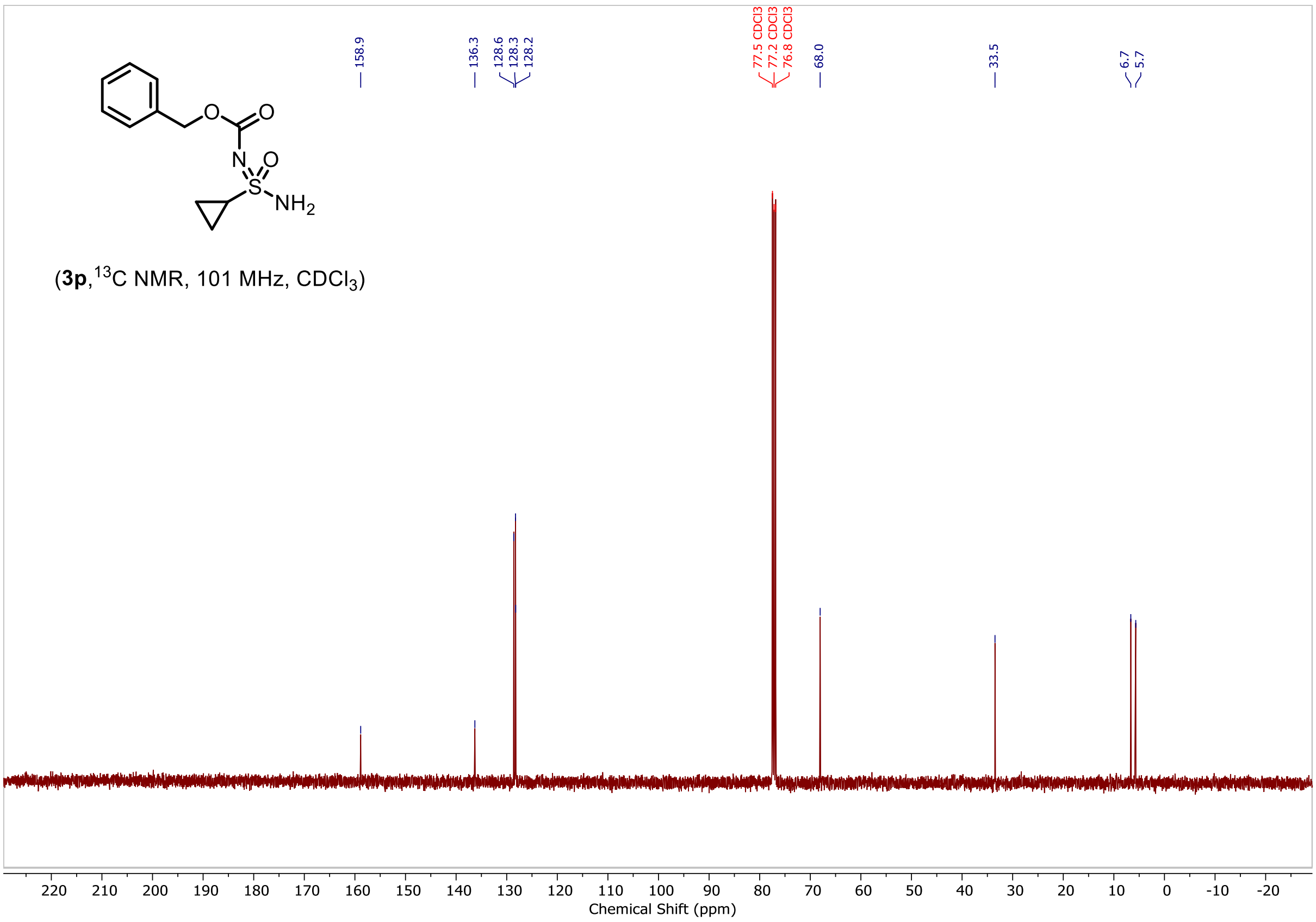

(3p, 13C NMR, 101 MHz, CDCl3)
158.9
136.3
128.6
128.3
128.2
77.5 CDCl3
77.2 CDCl3
76.8 CDCl3
68.0
33.5
6.7
5.7
Chemical Shift (ppm)

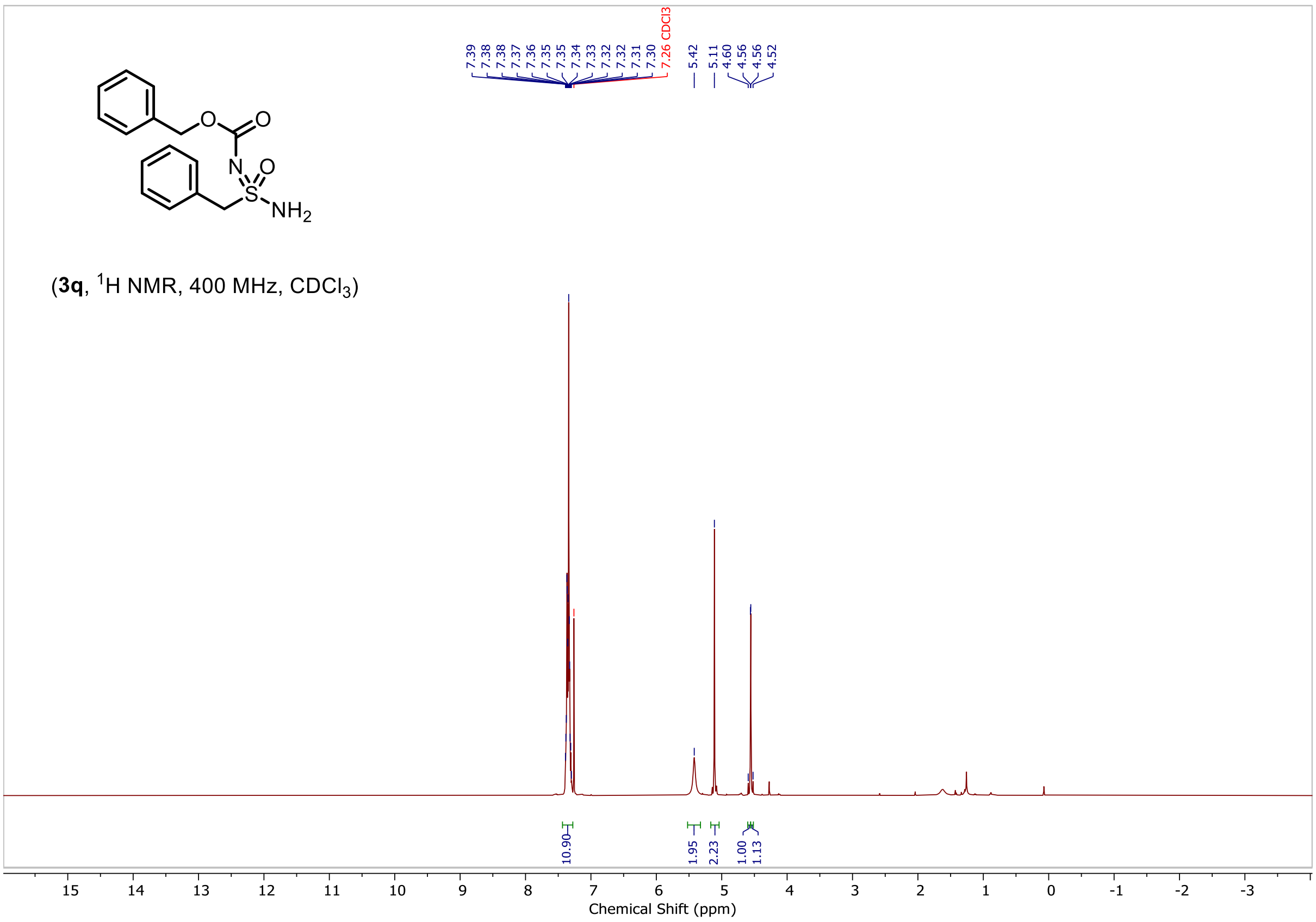

(3q, 1H NMR, 400 MHz, CDCl3)
7.39
7.38
7.38
7.37
7.36
7.35
7.35
7.34
7.33
7.32
7.32
7.31
7.30
7.26 CDCl3
5.42
5.11
4.60
4.56
4.56
4.52
10.90
1.95
2.23
1.00
1.13
Chemical Shift (ppm)

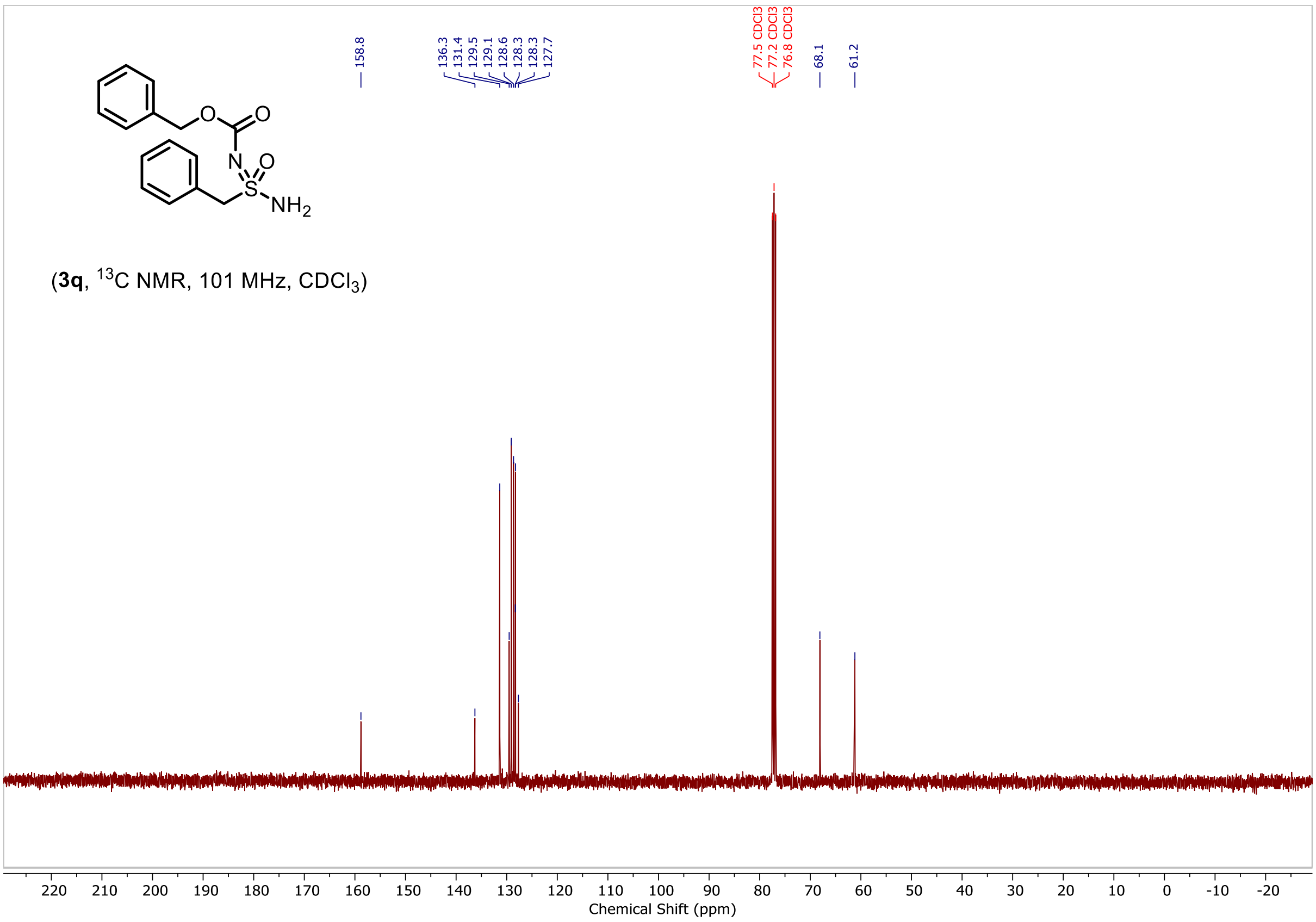

158.8
136.3
131.4
129.5
129.1
128.6
128.3
128.3
127.7
77.5 CDCl3
77.2 CDCl3
76.8 CDCl3
68.1
61.2
(3q, 13C NMR, 101 MHz, CDCl3)
Chemical Shift (ppm)

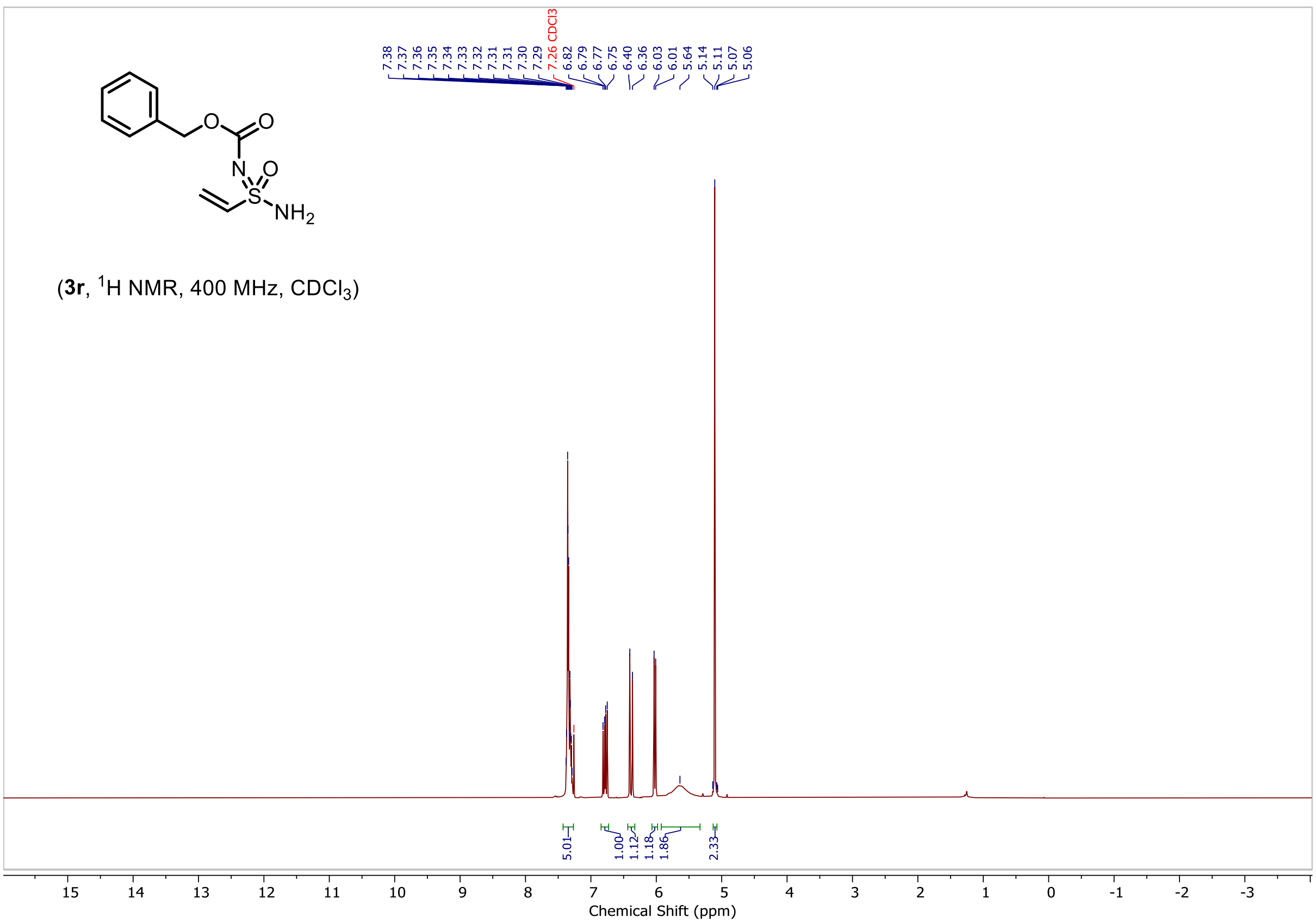

7.38
7.37
7.36
7.35
7.34
7.33
7.32
7.31
7.30
7.29
7.26 CDCl3
6.82
6.79
6.77
6.75
6.40
6.36
6.03
6.01
5.64
5.14
5.11
5.07
5.06
(3r, 1H NMR, 400 MHz, CDCl3)
5.01
1.00
1.12
1.18
1.86
2.33
Chemical Shift (ppm)

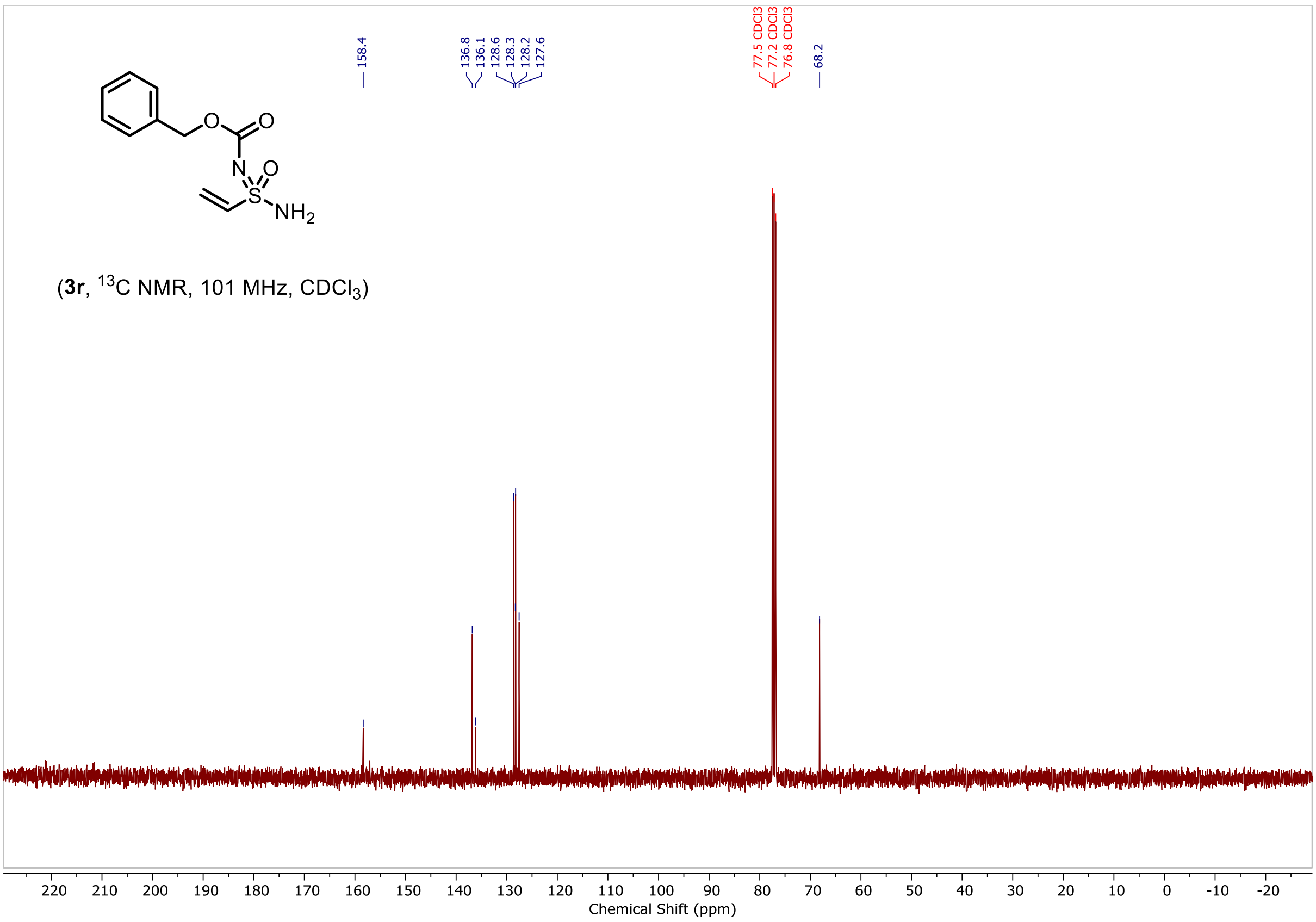

(**3r**, ^{13}C NMR, 101 MHz, $CDCl_3$)

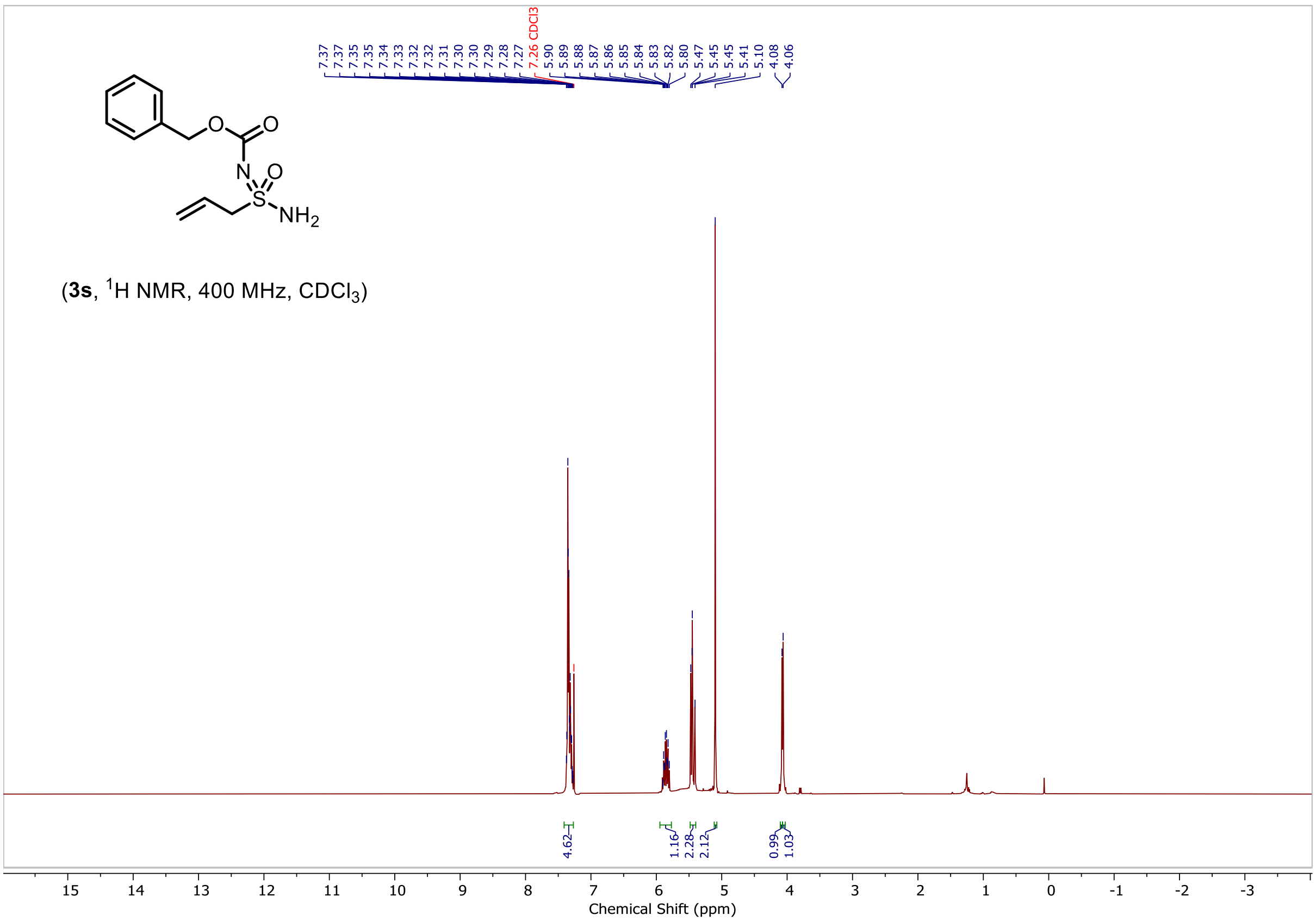

(**3s**, ^{1}H NMR, 400 MHz, $CDCl_3$)

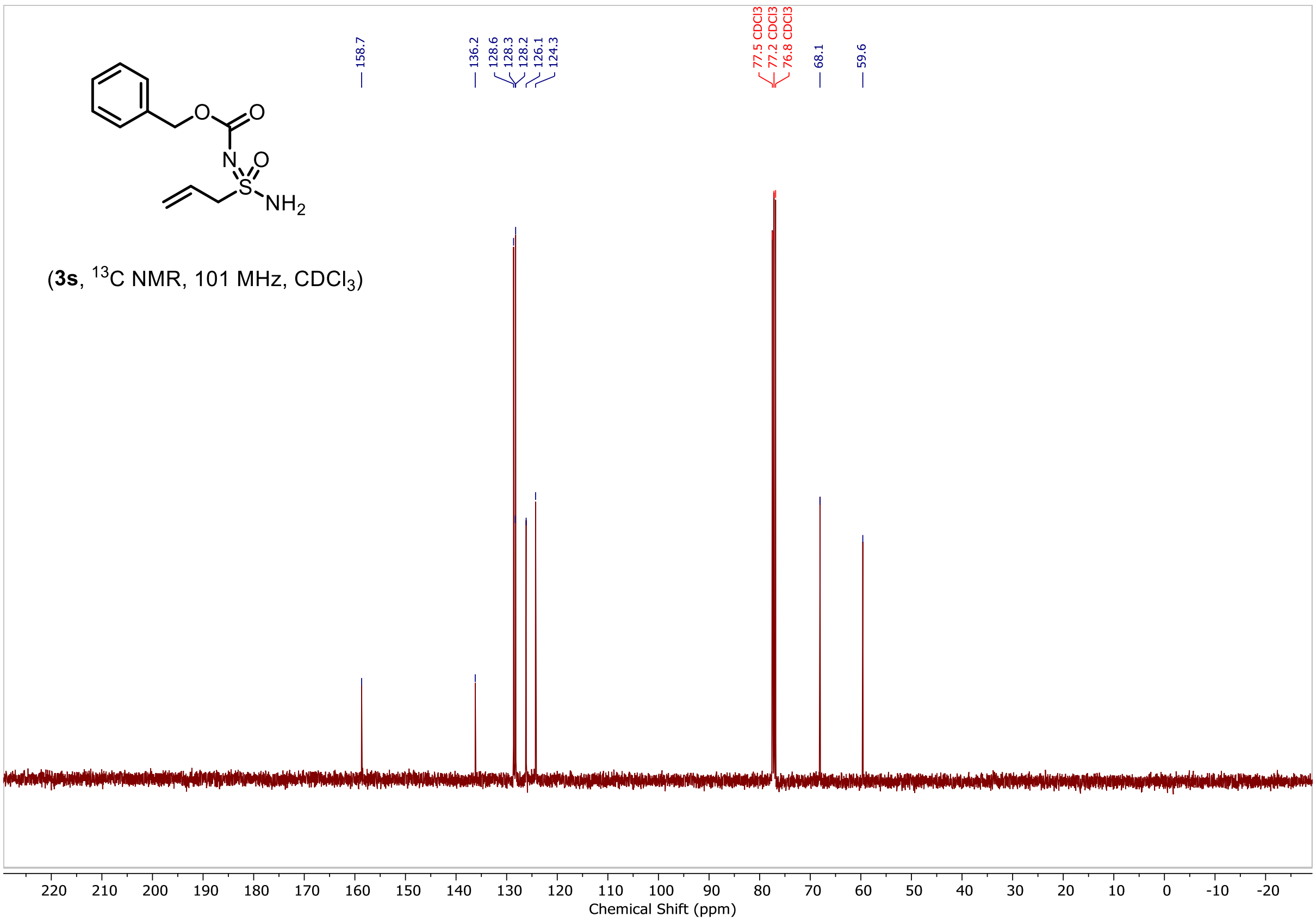
O
O
N
O
S
NH2
(3s, 13C NMR, 101 MHz, CDCl3)
158.7
136.2
128.6
128.3
128.2
126.1
124.3
77.5 CDCl3
77.2 CDCl3
76.8 CDCl3
68.1
59.6
220 210 200 190 180 170 160 150 140 130 120 110 100 90 80 70 60 50 40 30 20 10 0 -10 -20
Chemical Shift (ppm)

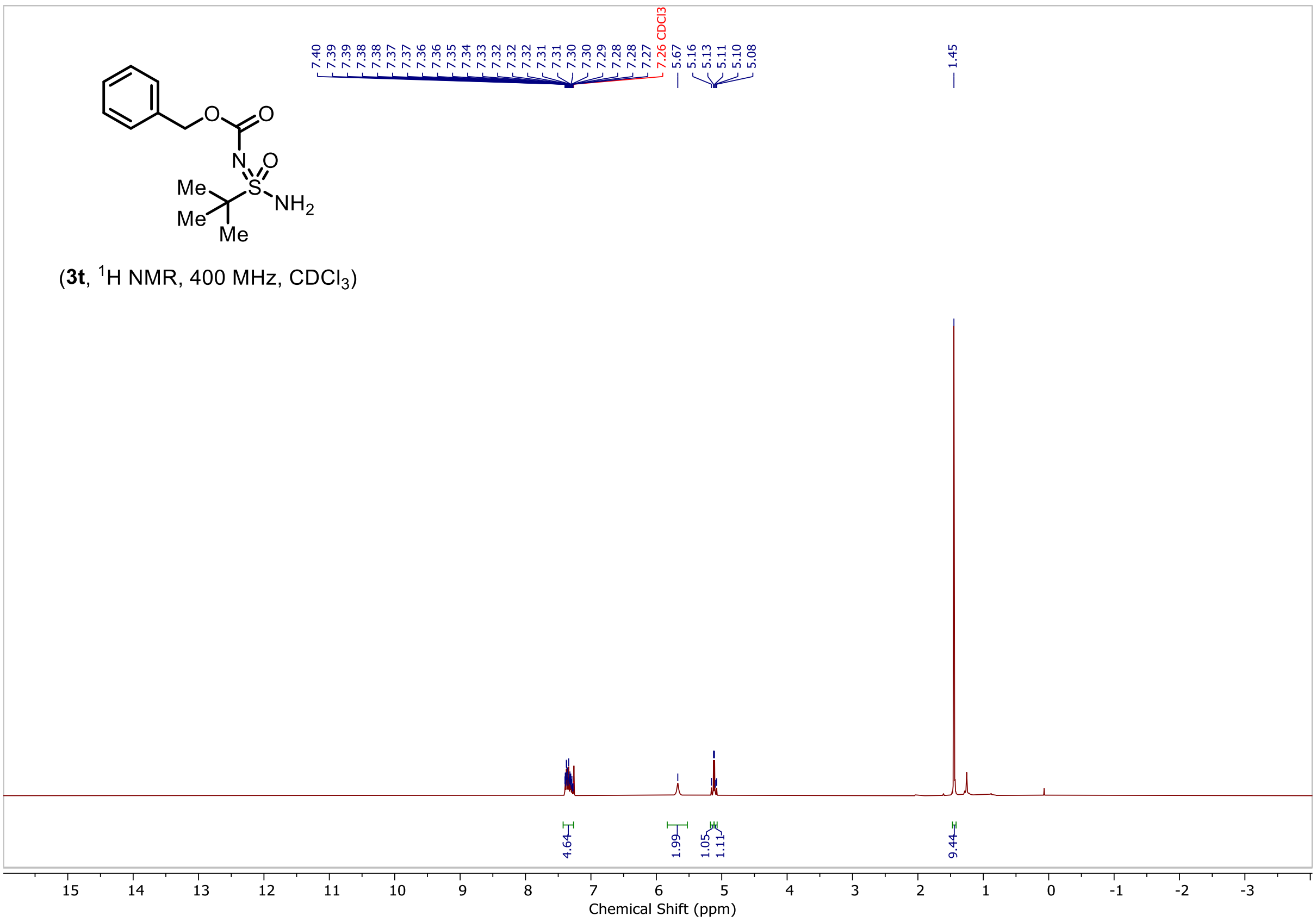
O
O
N
O
S
NH2
Me
Me
Me
(3t, 1H NMR, 400 MHz, CDCl3)
7.40
7.39
7.39
7.38
7.38
7.37
7.37
7.36
7.35
7.34
7.33
7.32
7.32
7.32
7.31
7.31
7.30
7.30
7.29
7.28
7.28
7.27
7.26 CDCl3
5.67
5.16
5.13
5.11
5.10
5.08
1.45
4.64
1.99
1.05
1.11
9.44
15 14 13 12 11 10 9 8 7 6 5 4 3 2 1 0 -1 -2 -3
Chemical Shift (ppm)

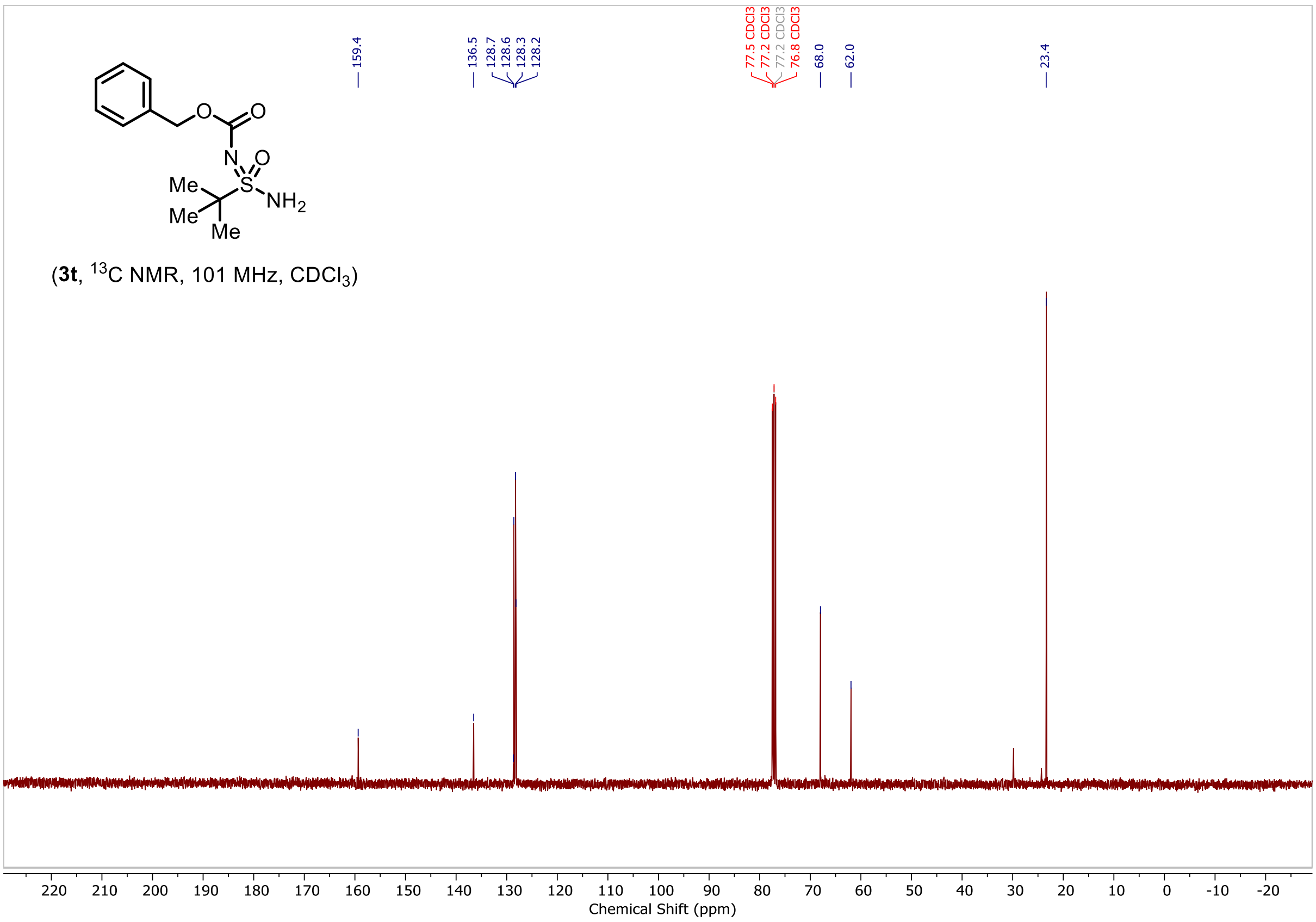

159.4
136.5
128.7
128.6
128.3
128.2
77.5 CDCl3
77.2 CDCl3
77.2 CDCl3
76.8 CDCl3
68.0
62.0
23.4
(3t, 13C NMR, 101 MHz, CDCl3)
Chemical Shift (ppm)

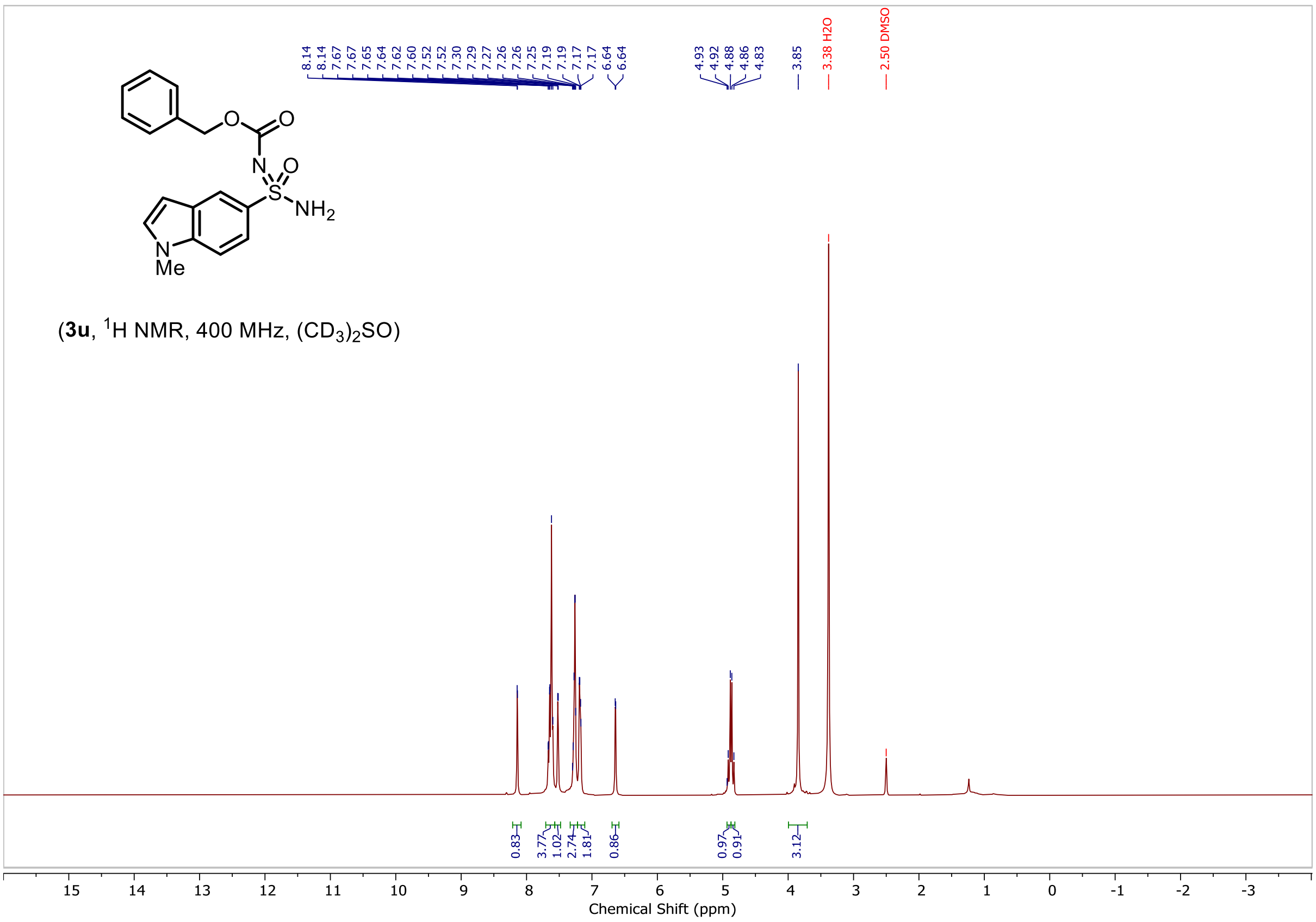

8.14
8.14
7.67
7.65
7.64
7.62
7.60
7.52
7.52
7.30
7.29
7.27
7.26
7.26
7.25
7.19
7.19
7.17
7.17
6.64
6.64
4.93
4.92
4.88
4.86
4.83
3.85
3.38 H2O
2.50 DMSO
(3u, 1H NMR, 400 MHz, (CD3)2SO)
0.83
3.77
1.02
2.74
1.81
0.86
0.97
0.91
3.12
Chemical Shift (ppm)

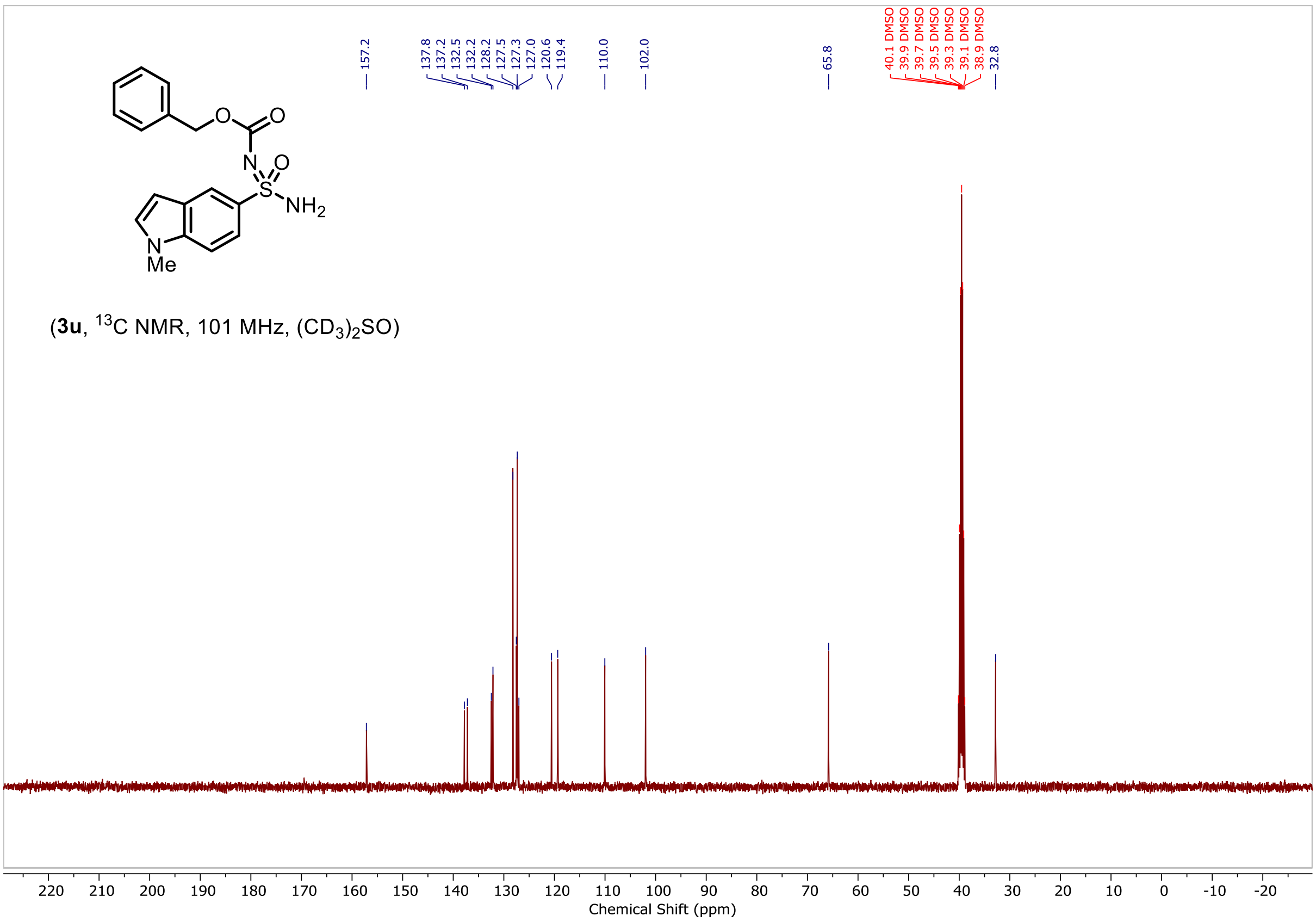

O
O
N
O
S
NH2
N
Me
(3u, 13C NMR, 101 MHz, (CD3)2SO)
157.2
137.8
137.2
132.5
132.2
128.2
127.5
127.3
127.0
120.6
119.4
110.0
102.0
65.8
40.1 DMSO
39.9 DMSO
39.7 DMSO
39.5 DMSO
39.3 DMSO
39.1 DMSO
38.9 DMSO
32.8
Chemical Shift (ppm)

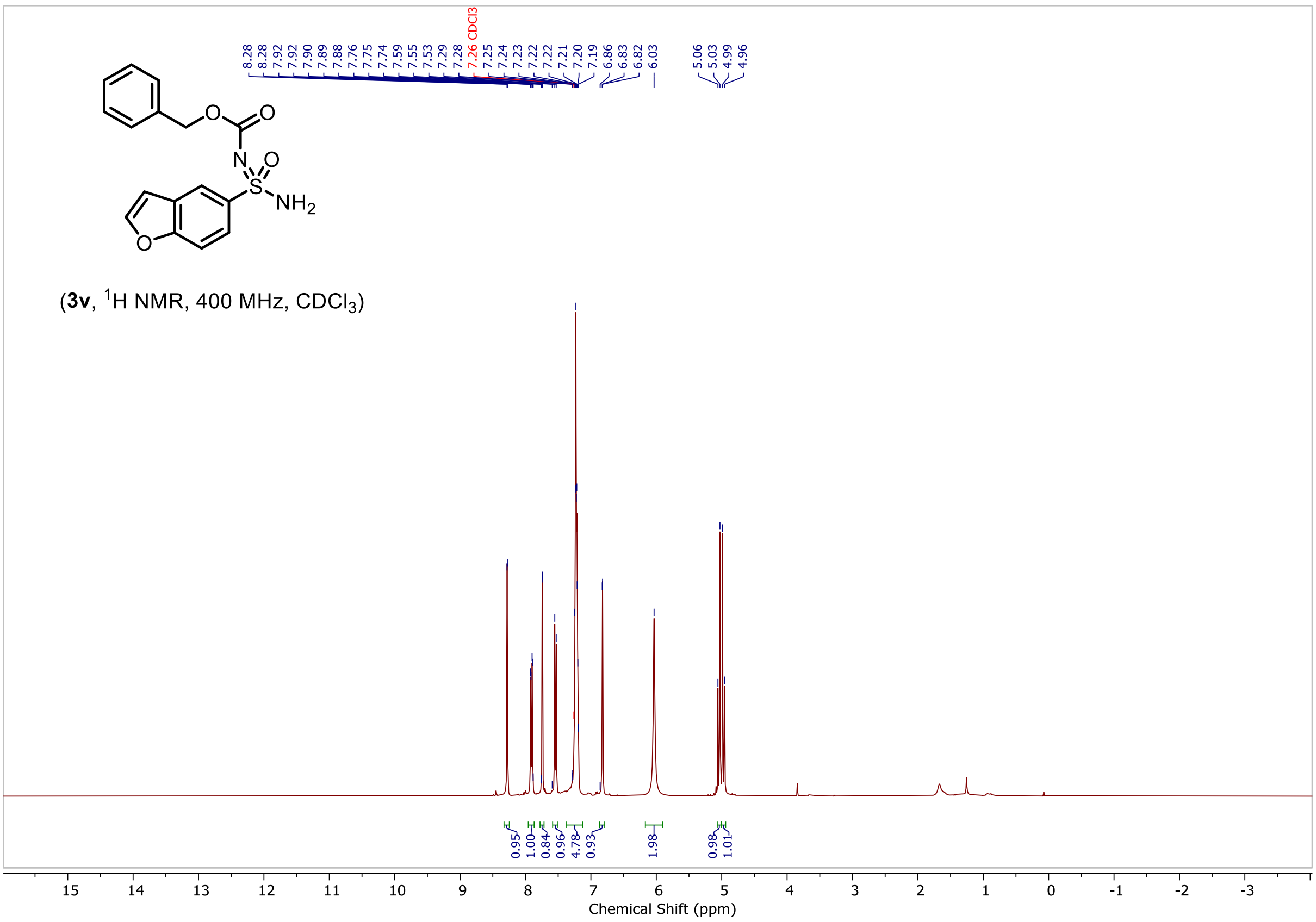

O
O
N
O
S
NH2
O
(3v, 1H NMR, 400 MHz, CDCl3)
8.28
8.28
7.92
7.92
7.90
7.89
7.88
7.76
7.75
7.74
7.59
7.55
7.53
7.29
7.28
7.26 CDCl3
7.25
7.24
7.23
7.22
7.22
7.21
7.20
7.19
6.86
6.83
6.82
6.03
5.06
5.03
4.99
4.96
0.95
1.00
0.84
0.96
4.78
0.93
1.98
0.98
1.01
Chemical Shift (ppm)

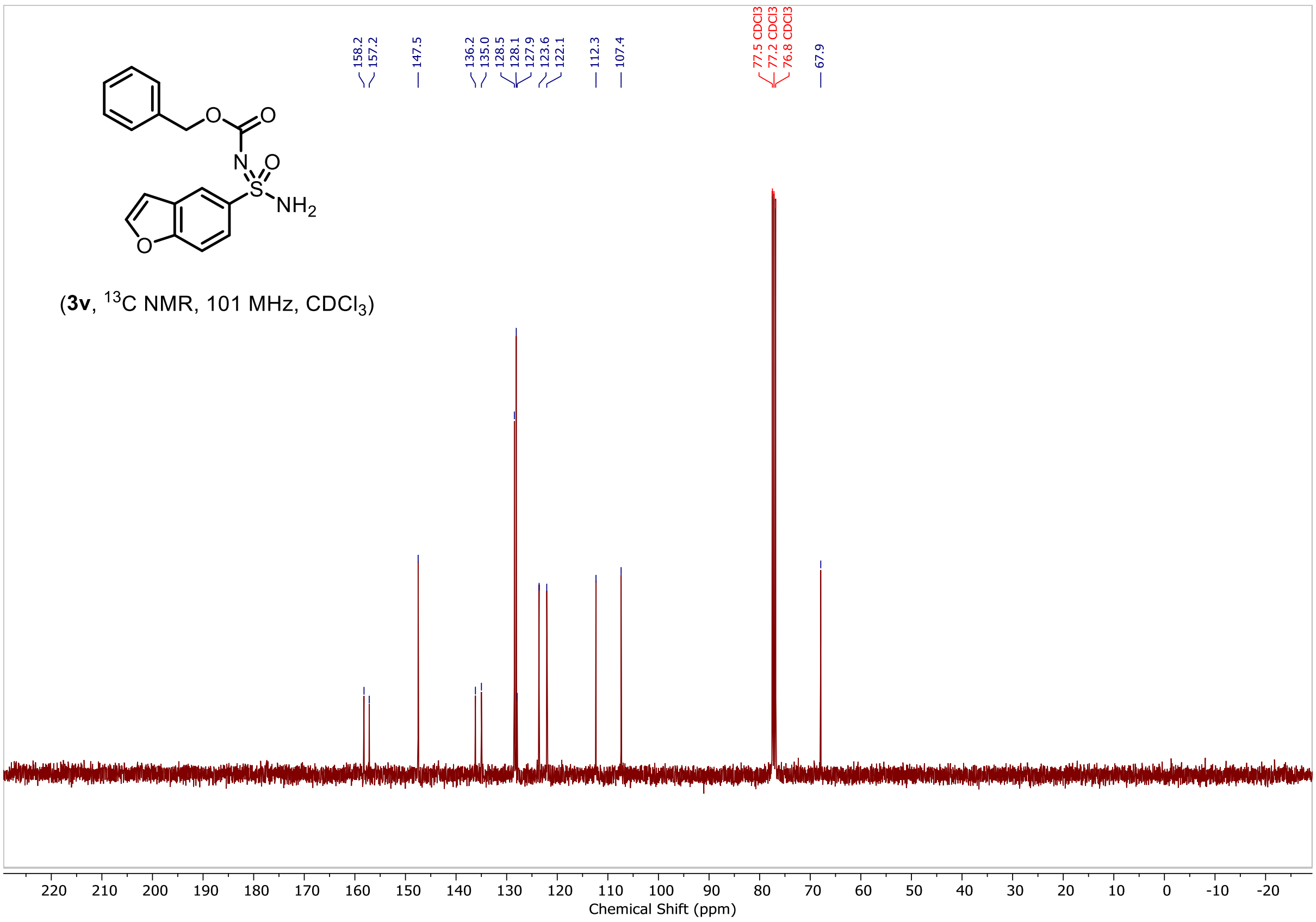

158.2
157.2
147.5
136.2
135.0
128.5
128.1
127.9
123.6
122.1
112.3
107.4
77.5 CDCl3
77.2 CDCl3
76.8 CDCl3
67.9
(3v, 13C NMR, 101 MHz, CDCl3)
Chemical Shift (ppm)

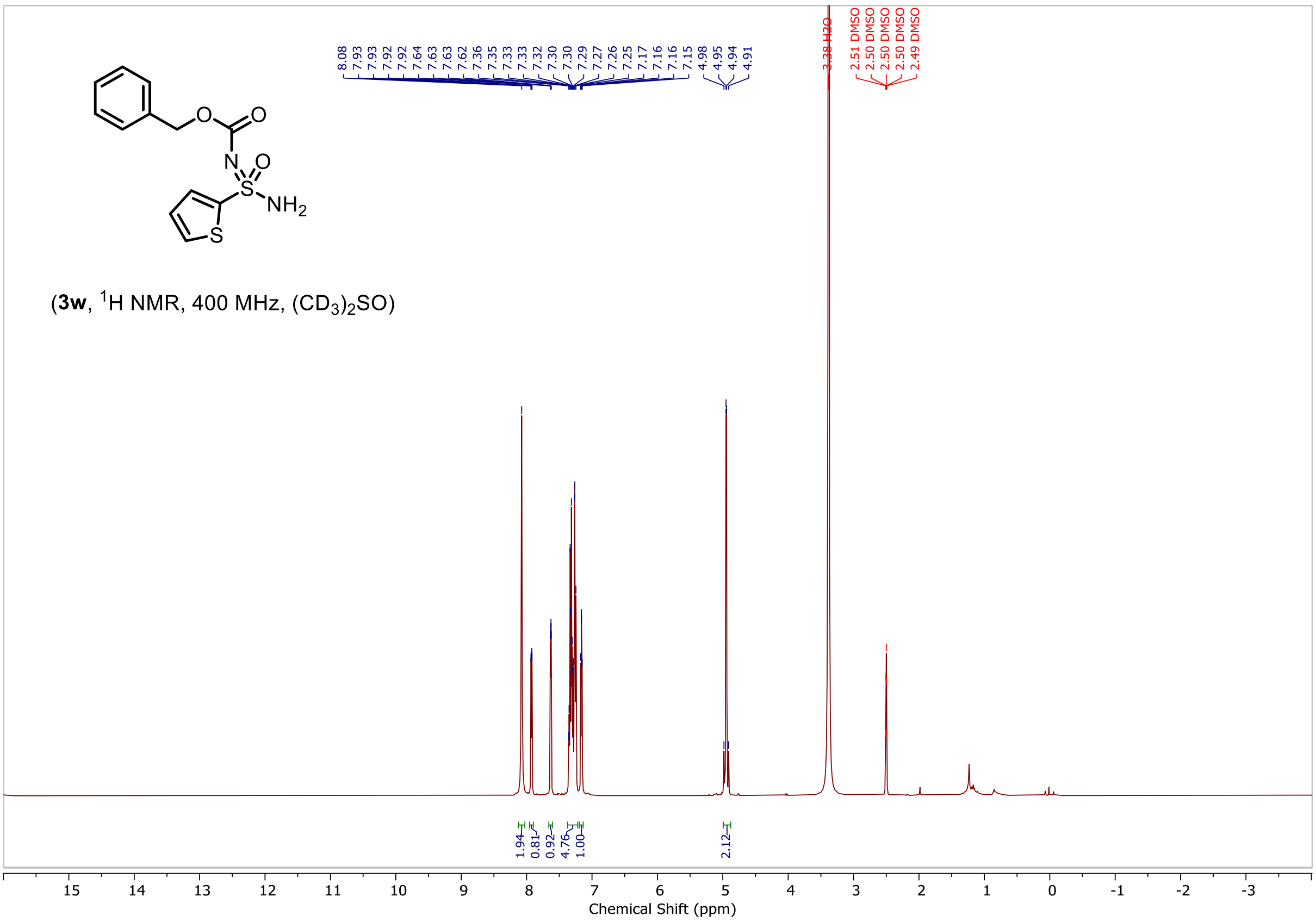

8.08
7.93
7.93
7.92
7.92
7.64
7.63
7.63
7.62
7.36
7.35
7.33
7.33
7.32
7.30
7.29
7.27
7.26
7.25
7.17
7.16
7.16
7.15
4.98
4.95
4.94
4.91
3.38 H2O
2.51 DMSO
2.50 DMSO
2.50 DMSO
2.50 DMSO
2.49 DMSO
(3w, 1H NMR, 400 MHz, (CD3)2SO)
1.94
0.81
0.92
4.76
1.00
2.12
Chemical Shift (ppm)

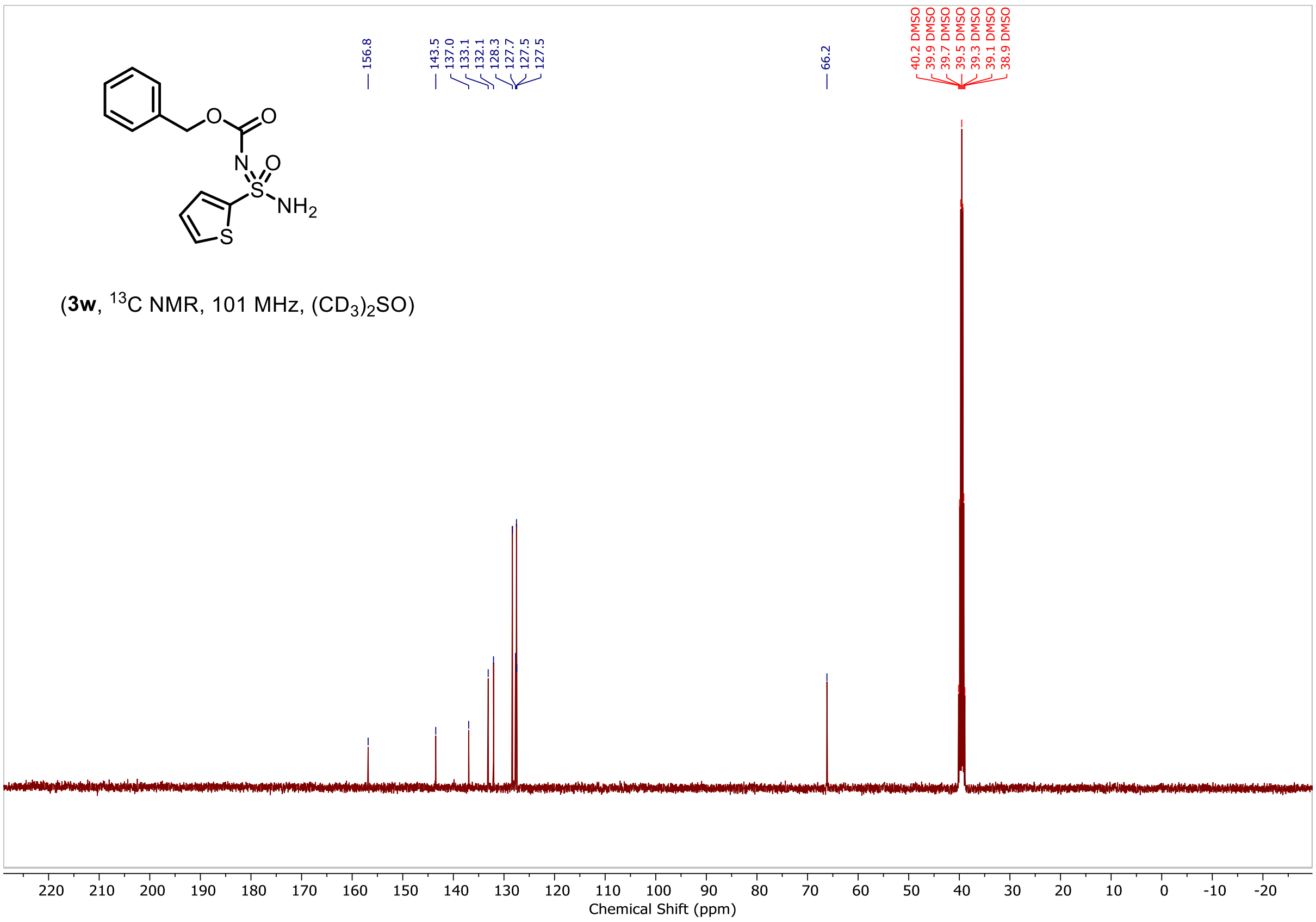

156.8
143.5
137.0
133.1
132.1
128.3
127.7
127.5
127.5
66.2
40.2 DMSO
39.9 DMSO
39.7 DMSO
39.5 DMSO
39.3 DMSO
39.1 DMSO
38.9 DMSO
O
O
N
O
S
NH2
S
(3w, 13C NMR, 101 MHz, (CD3)2SO)
220 210 200 190 180 170 160 150 140 130 120 110 100 90 80 70 60 50 40 30 20 10 0 -10 -20
Chemical Shift (ppm)

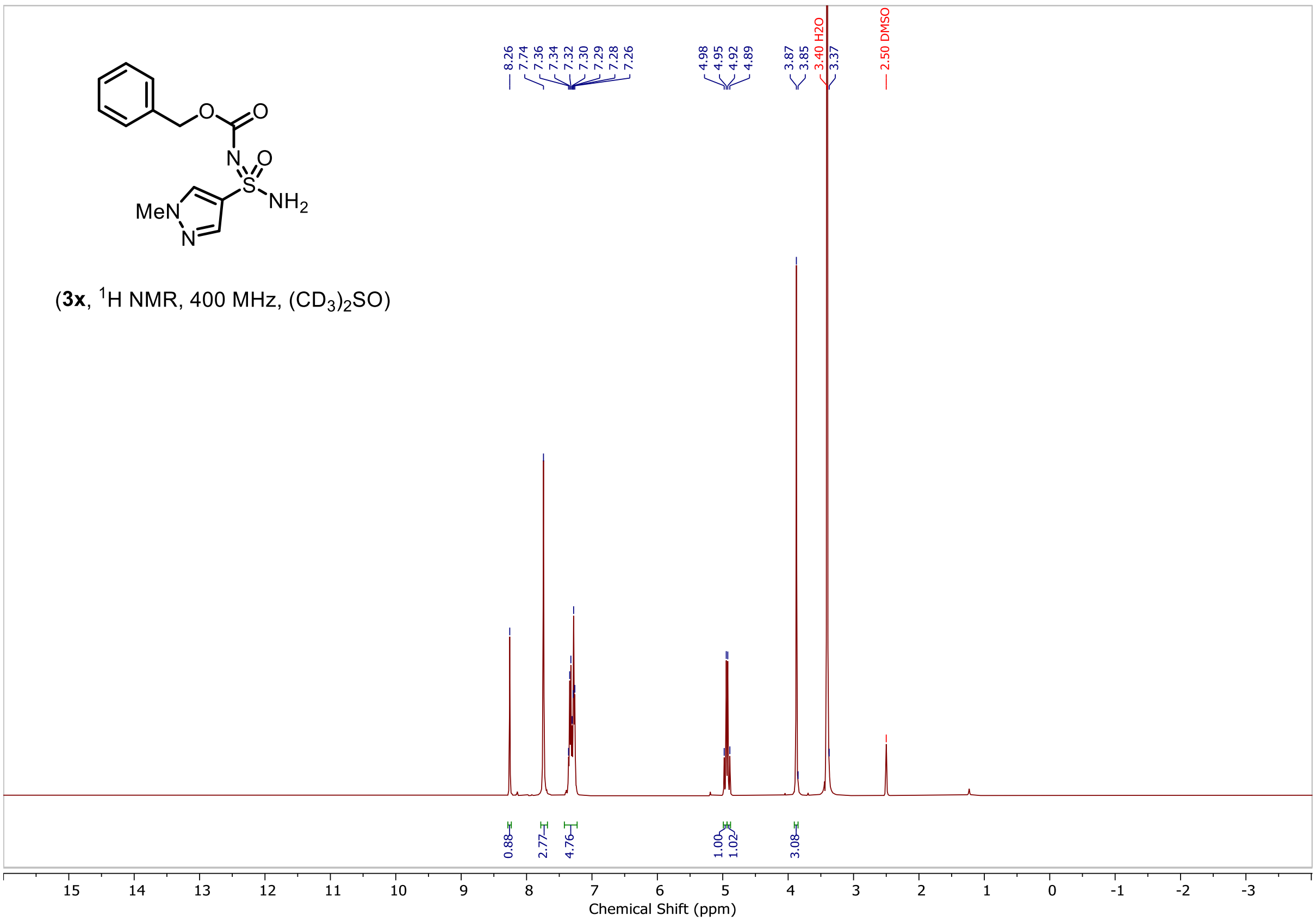

8.26
7.74
7.36
7.34
7.32
7.30
7.29
7.28
7.26
4.98
4.95
4.92
4.89
3.87
3.85
3.40 H2O
3.37
2.50 DMSO
O
O
N
O
S
NH2
MeN
N
(3x, 1H NMR, 400 MHz, (CD3)2SO)
0.88
2.77
4.76
1.00
1.02
3.08
15 14 13 12 11 10 9 8 7 6 5 4 3 2 1 0 -1 -2 -3
Chemical Shift (ppm)

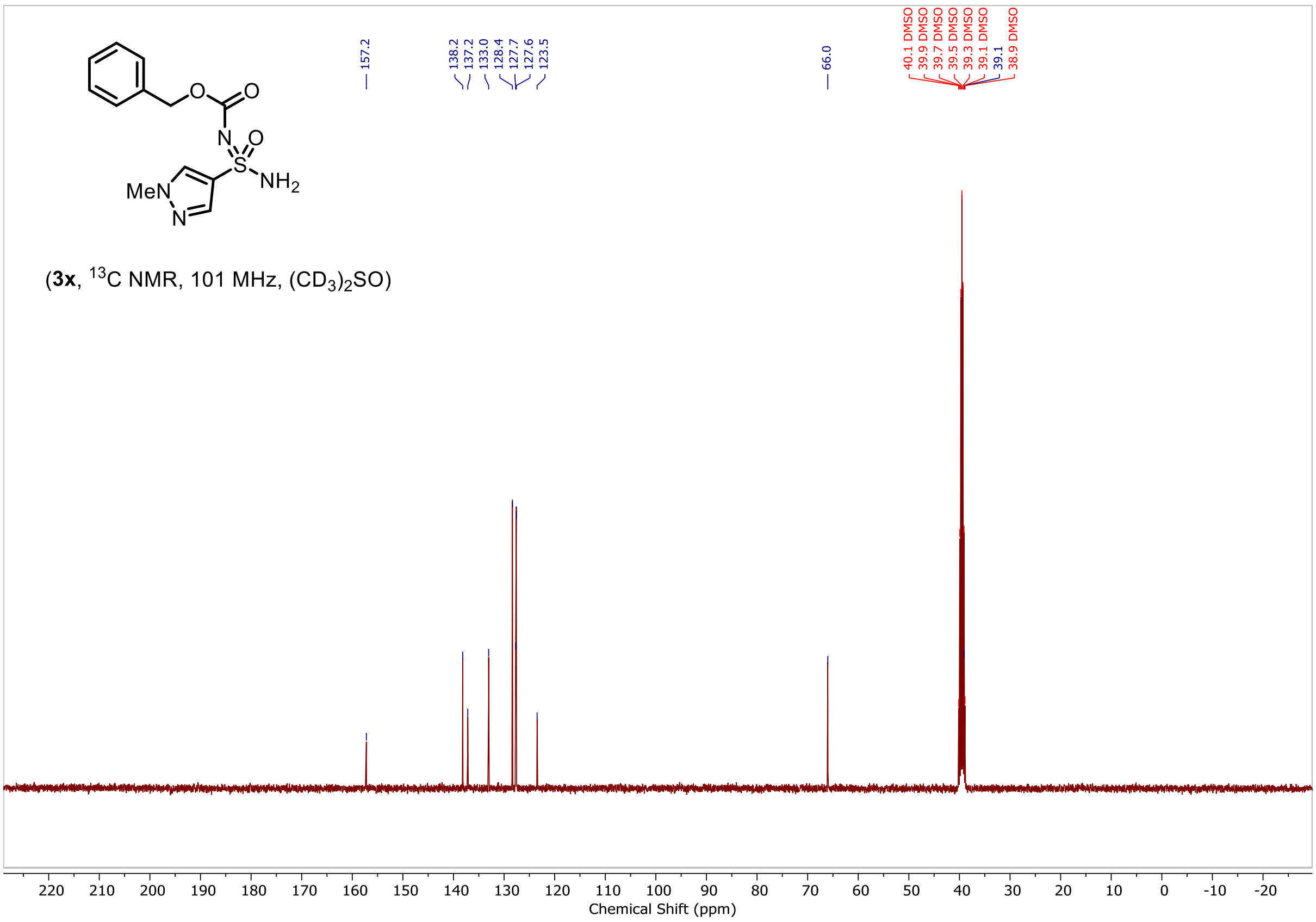

(**3x**, ^{13}C NMR, 101 MHz, $(CD_3)_2SO$)

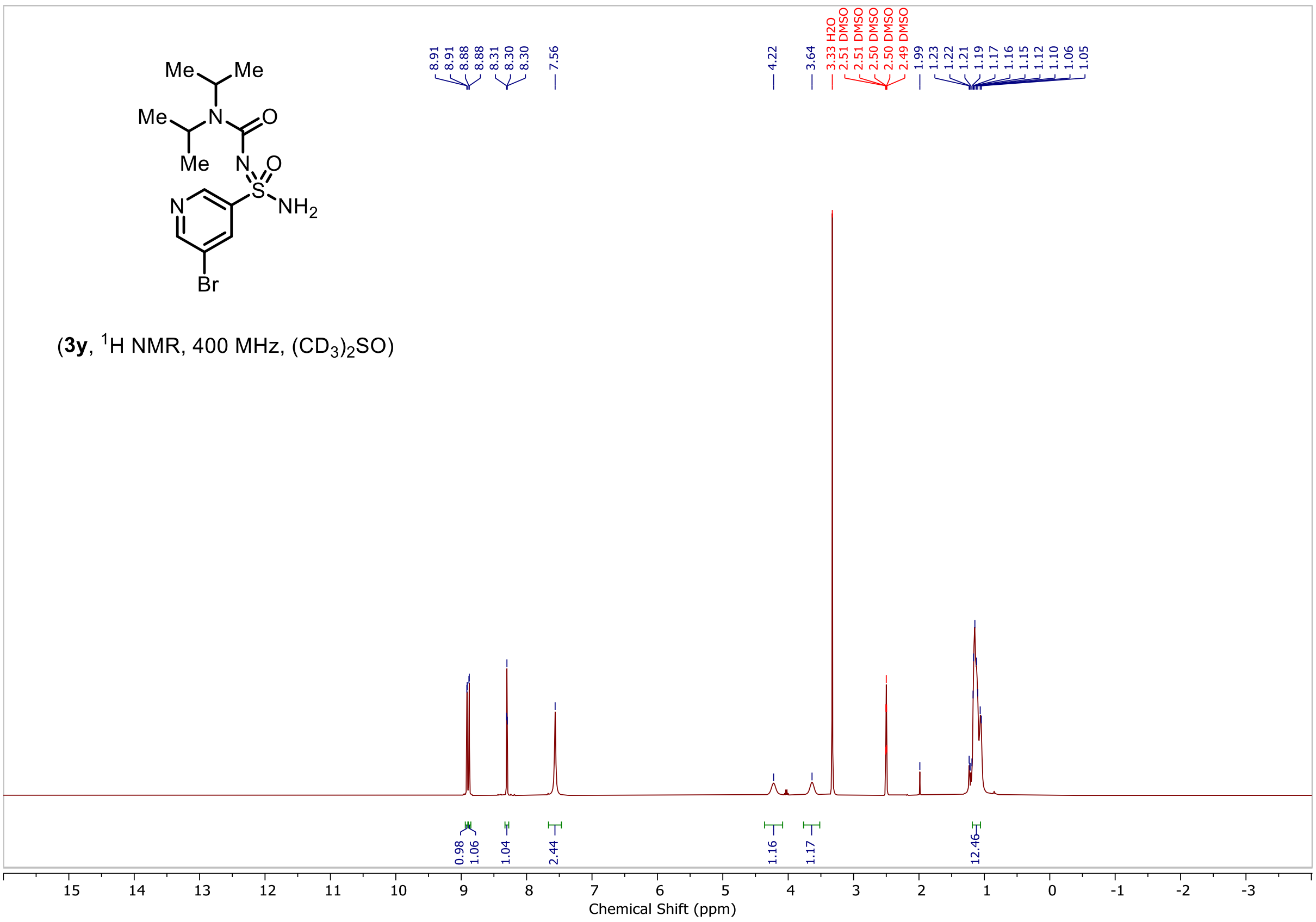

(**3y**, ^{1}H NMR, 400 MHz, $(CD_3)_2SO$)

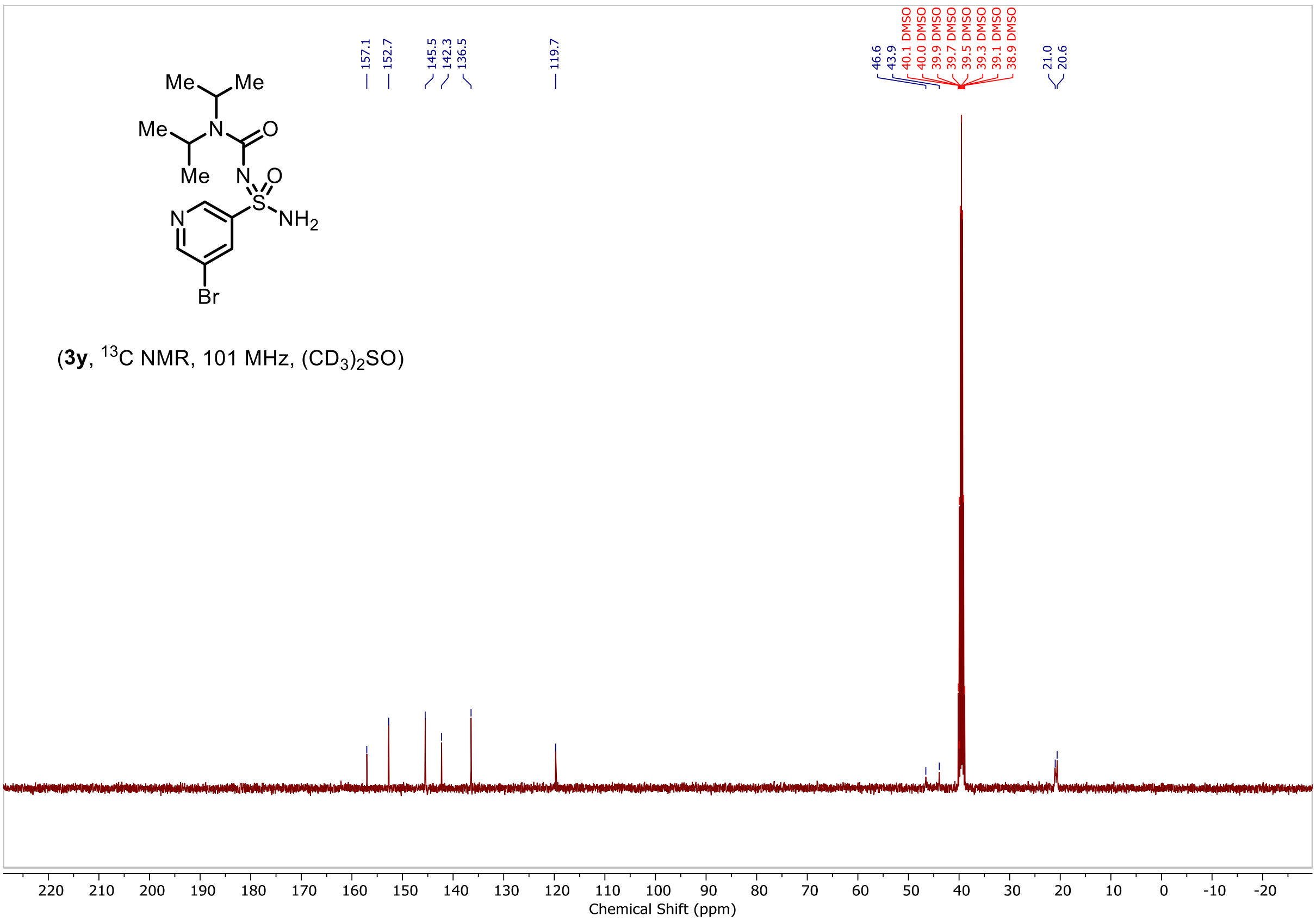
157.1
152.7
145.5
142.3
136.5
119.7
46.6
43.9
40.1 DMSO
40.0 DMSO
39.9 DMSO
39.7 DMSO
39.5 DMSO
39.3 DMSO
39.1 DMSO
38.9 DMSO
21.0
20.6
Me
Me
Me
Me
N
O
N
O
S
NH2
N
Br
(3y, 13C NMR, 101 MHz, (CD3)2SO)
220 210 200 190 180 170 160 150 140 130 120 110 100 90 80 70 60 50 40 30 20 10 0 -10 -20
Chemical Shift (ppm)

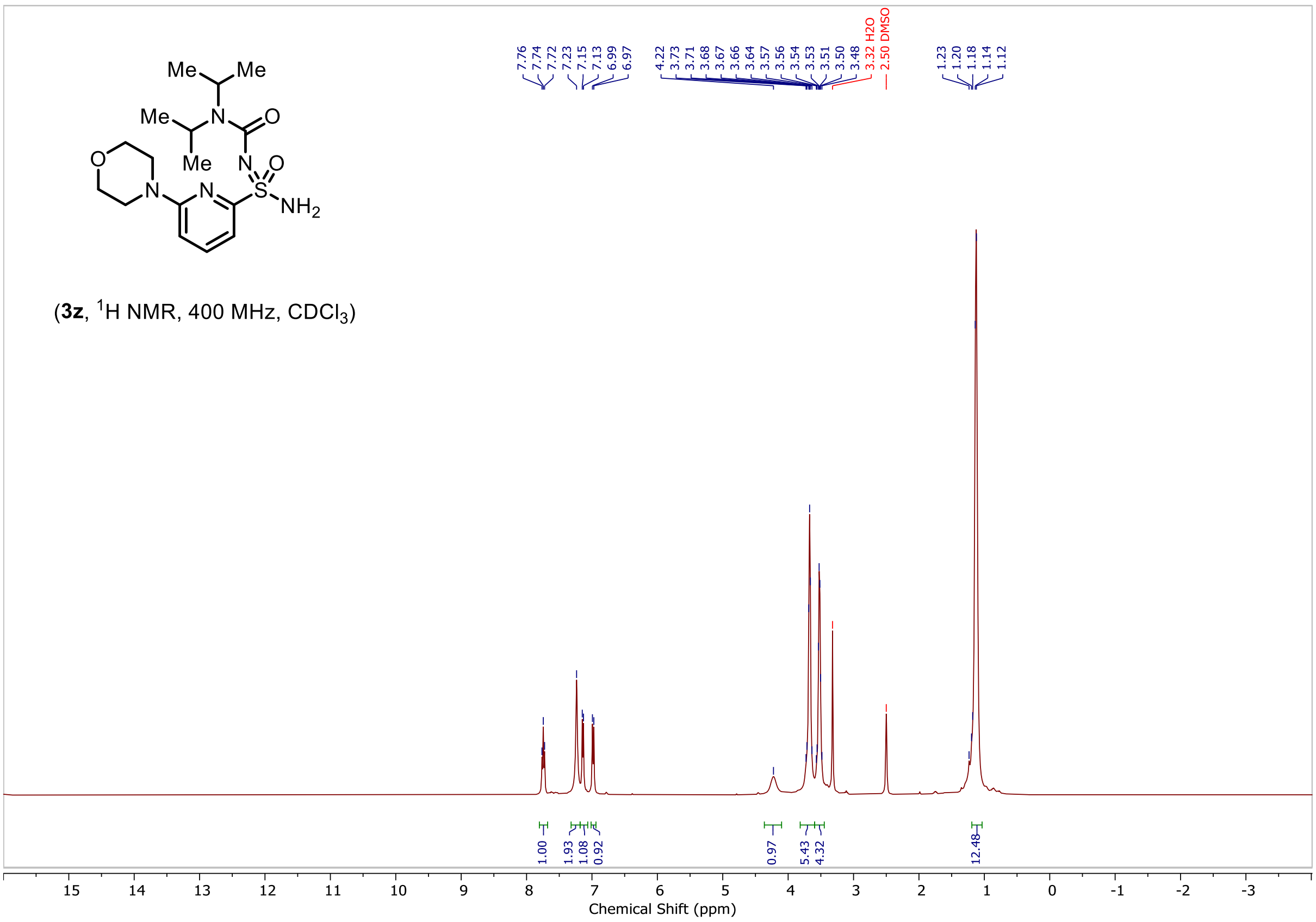
7.76
7.74
7.72
7.23
7.15
7.13
6.99
6.97
4.22
3.73
3.71
3.68
3.67
3.66
3.64
3.57
3.56
3.54
3.53
3.51
3.50
3.48
3.32 H2O
2.50 DMSO
1.23
1.20
1.18
1.14
1.12
Me
Me
Me
Me
N
O
O
N
N
N
O
S
NH2
(3z, 1H NMR, 400 MHz, CDCl3)
1.00
1.93
1.08
0.92
0.97
5.43
4.32
12.48
15 14 13 12 11 10 9 8 7 6 5 4 3 2 1 0 -1 -2 -3
Chemical Shift (ppm)

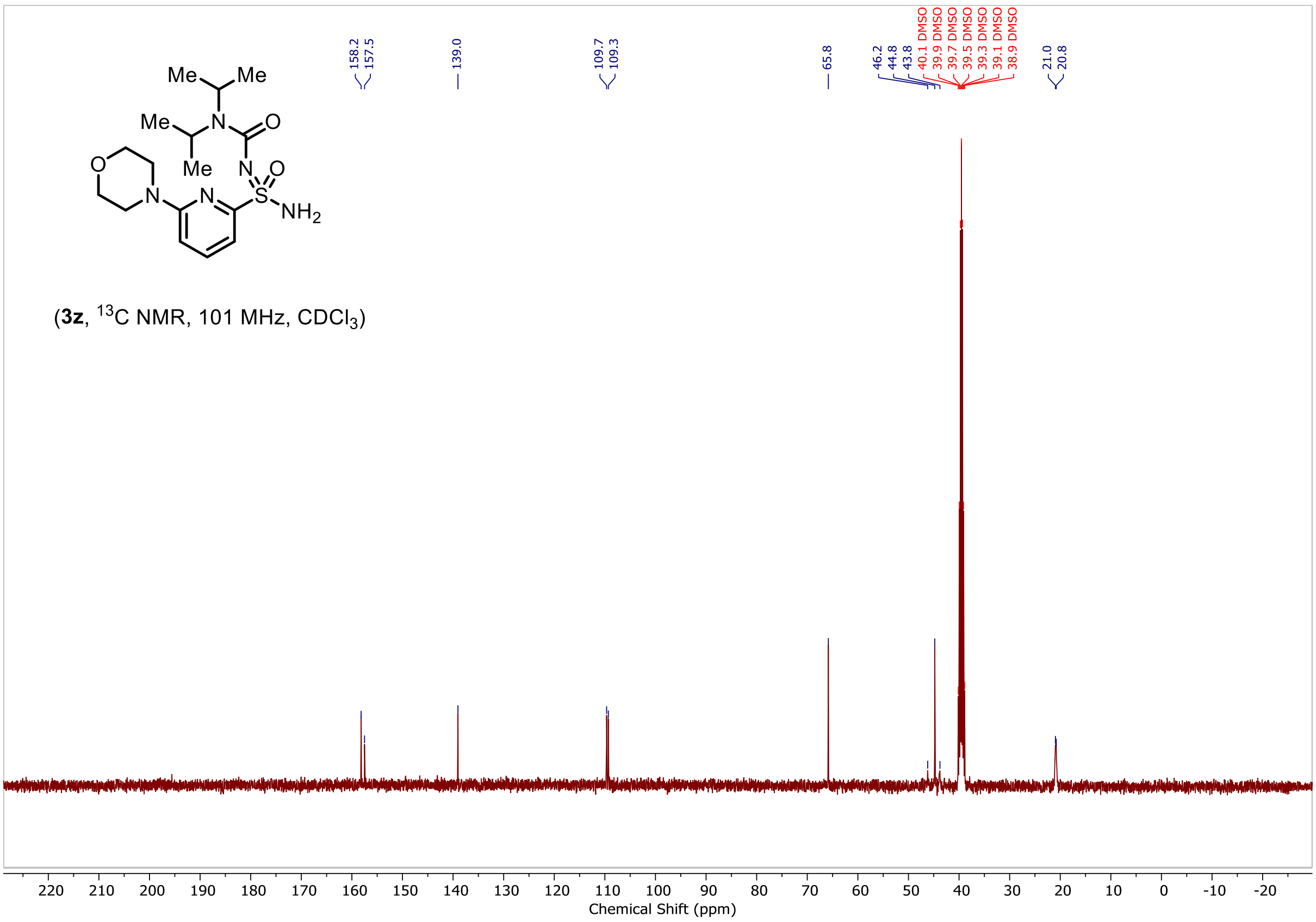

(**3z**, ^{13}C NMR, 101 MHz, $CDCl_3$)

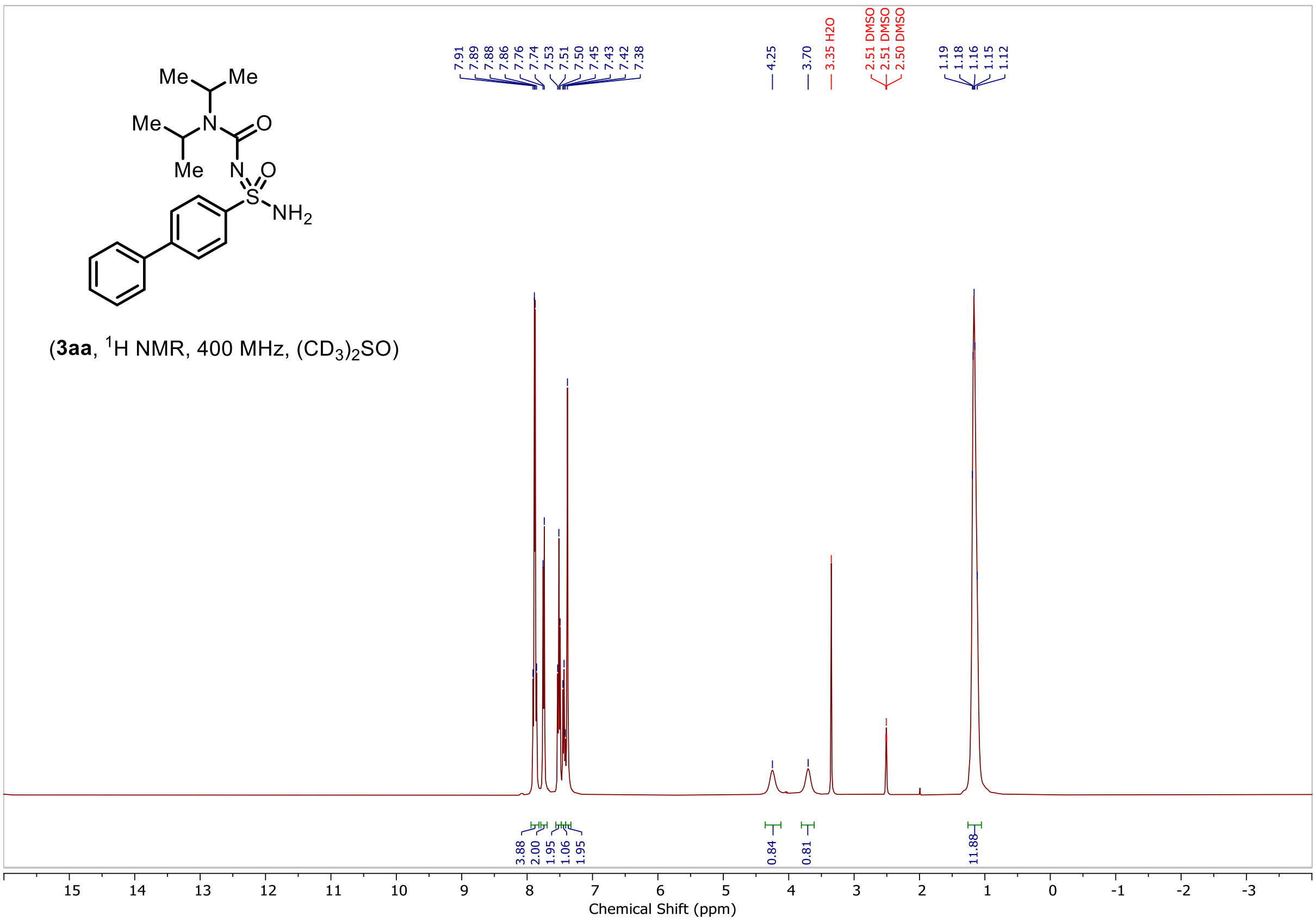

(**3aa**, ^{1}H NMR, 400 MHz, $(CD_3)_2SO$)

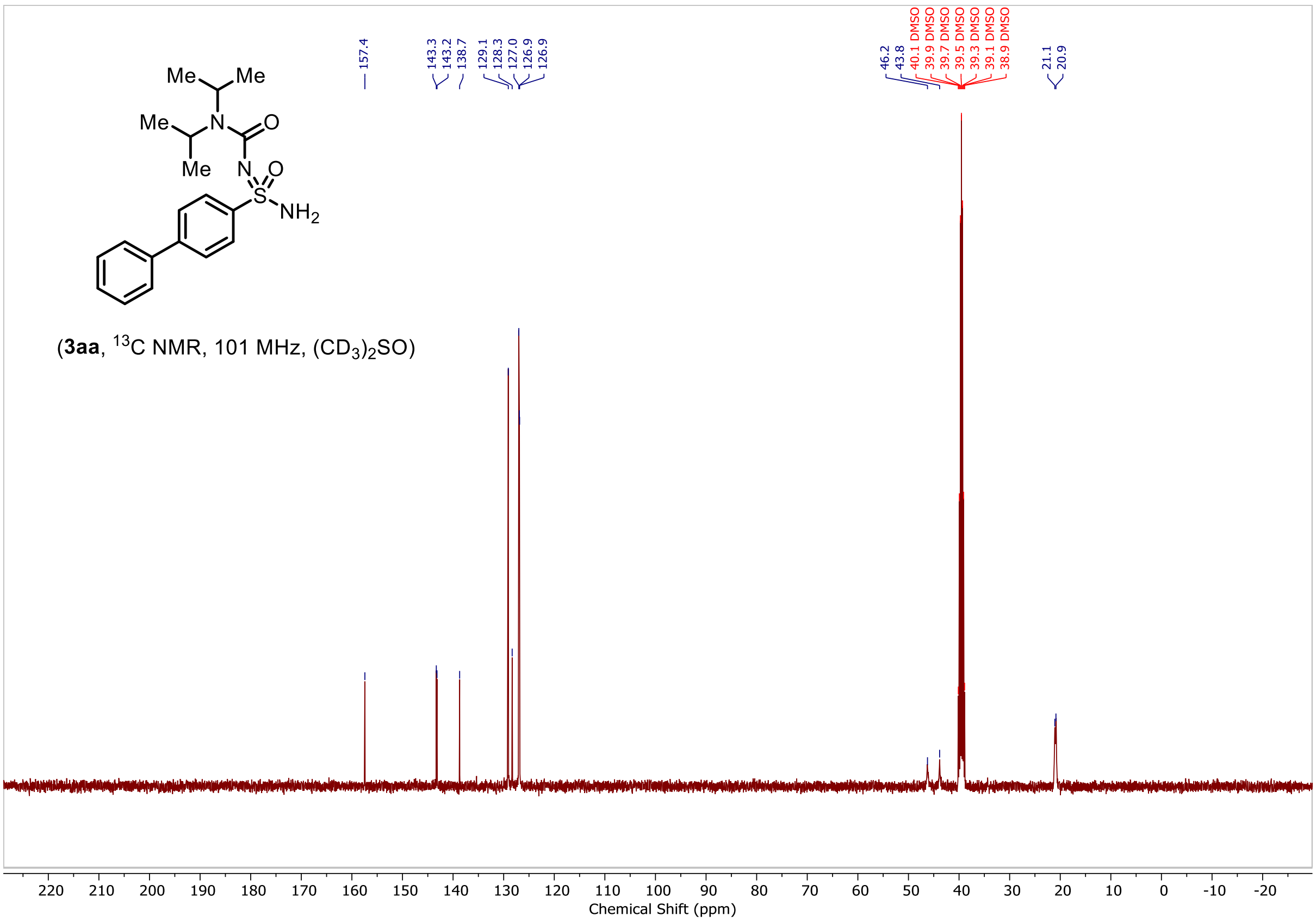

(**3aa**, ^{13}C NMR, 101 MHz, $(CD_3)_2SO$)

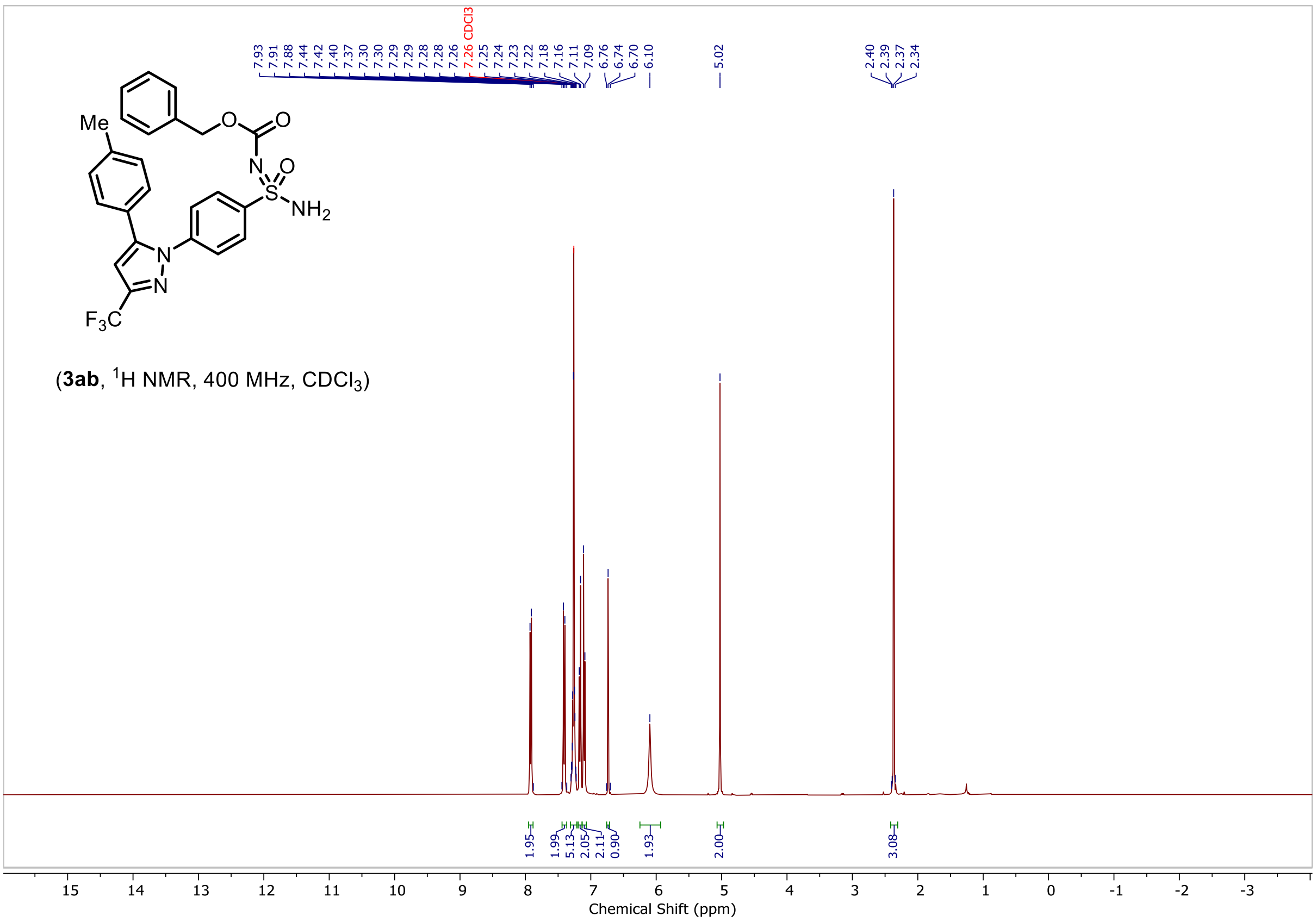

(**3ab**, ^{1}H NMR, 400 MHz, $CDCl_3$)

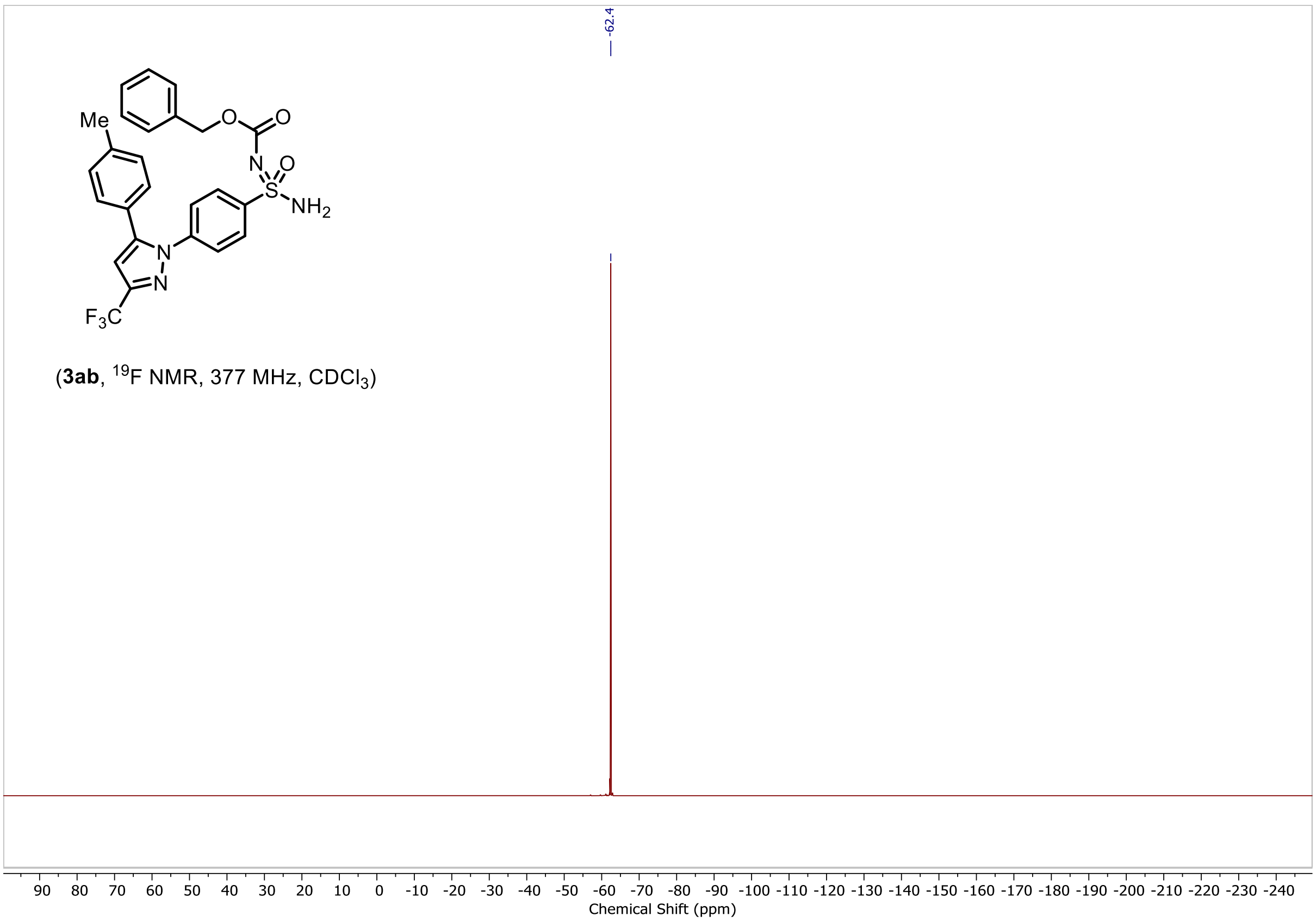

-62.4
Me
O
O
N
O
S
NH2
N
N
F3C
(3ab, 19F NMR, 377 MHz, CDCl3)
90 80 70 60 50 40 30 20 10 0 -10 -20 -30 -40 -50 -60 -70 -80 -90 -100 -110 -120 -130 -140 -150 -160 -170 -180 -190 -200 -210 -220 -230 -240
Chemical Shift (ppm)

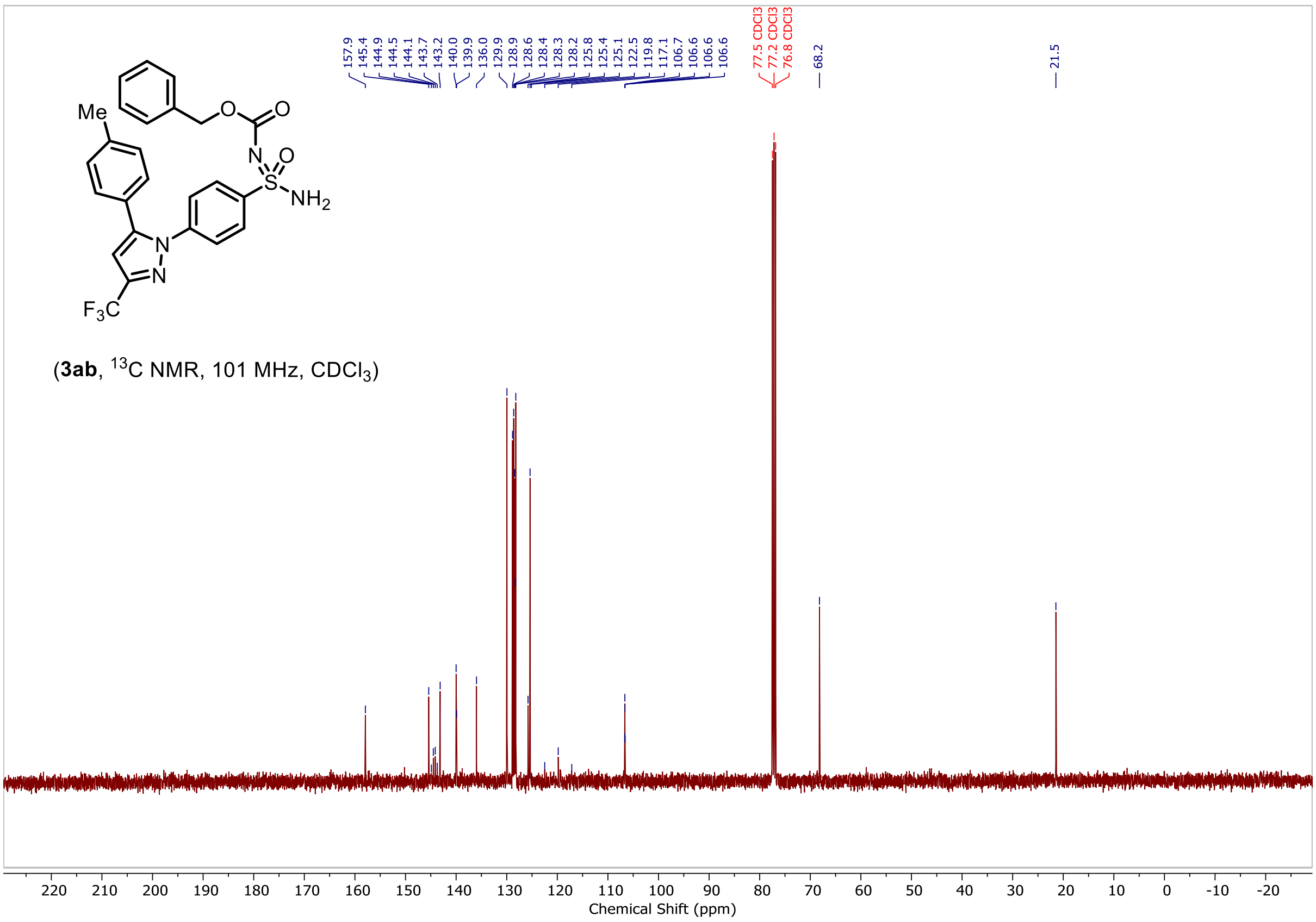

157.9
145.4
144.9
144.5
144.1
143.7
143.2
140.0
139.9
136.0
129.9
128.9
128.6
128.4
128.3
128.2
125.8
125.4
125.1
122.5
119.8
117.1
106.7
106.6
106.6
106.6
77.5 CDCl3
77.2 CDCl3
76.8 CDCl3
68.2
21.5
Me
O
O
N
O
S
NH2
N
N
F3C
(3ab, 13C NMR, 101 MHz, CDCl3)
220 210 200 190 180 170 160 150 140 130 120 110 100 90 80 70 60 50 40 30 20 10 0 -10 -20
Chemical Shift (ppm)

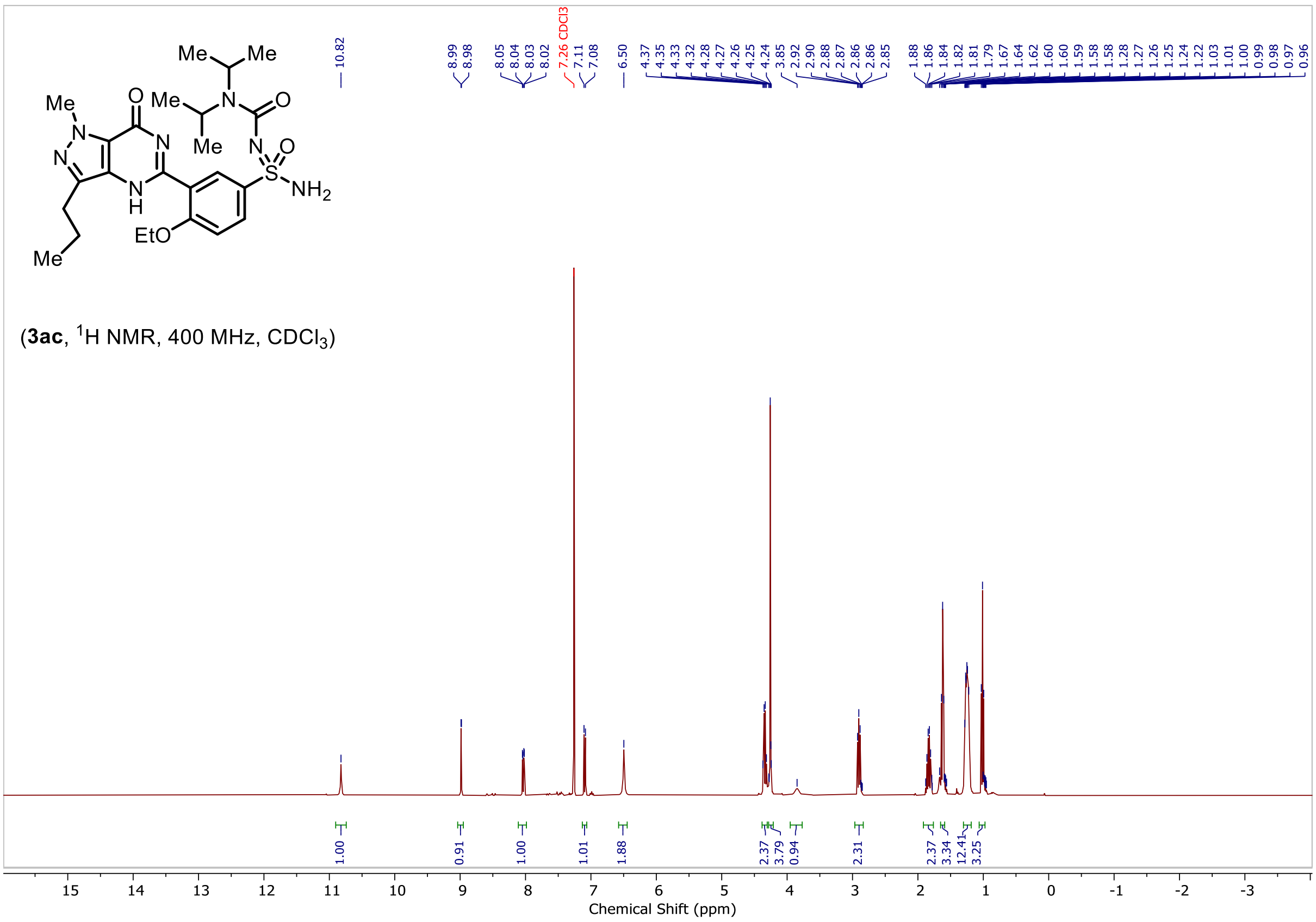
10.82
8.99 8.98
8.05 8.04 8.03 8.02
7.26 CDCl3
7.11 7.08
6.50
4.37 4.35 4.33 4.32 4.28 4.27 4.26 4.25 4.24
3.85
2.92 2.90 2.88 2.87 2.86 2.86 2.85
1.88 1.86 1.84 1.82 1.81 1.79 1.67 1.64 1.62 1.60 1.60 1.59 1.58 1.58 1.28 1.27 1.26 1.25 1.24 1.22 1.03 1.01 1.00 0.99 0.98 0.97 0.96
Me
N
N
O
N
H
N
EtO
Me
Me
Me
Me
Me
N
O
N
S
O
NH2
(3ac, 1H NMR, 400 MHz, CDCl3)
1.00
0.91
1.00
1.01
1.88
2.37
3.79
0.94
2.31
2.37
3.34
12.41
3.25
15 14 13 12 11 10 9 8 7 6 5 4 3 2 1 0 -1 -2 -3
Chemical Shift (ppm)

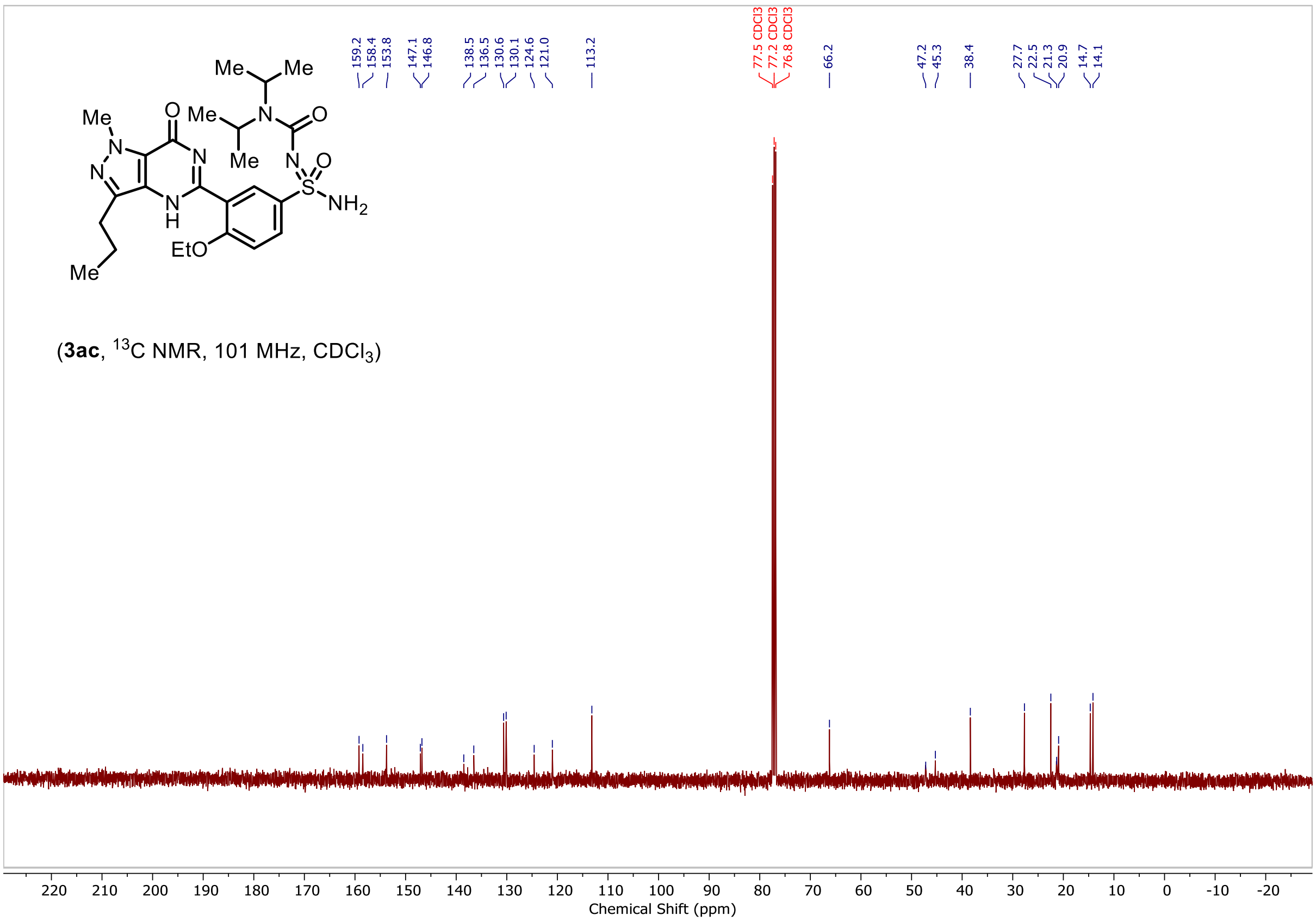
159.2 158.4 153.8 147.1 146.8 138.5 136.5 130.6 130.1 124.6 121.0
113.2
77.5 CDCl3 77.2 CDCl3 76.8 CDCl3
66.2
47.2 45.3
38.4
27.7 22.5 21.3 20.9 14.7 14.1
(3ac, 13C NMR, 101 MHz, CDCl3)
220 210 200 190 180 170 160 150 140 130 120 110 100 90 80 70 60 50 40 30 20 10 0 -10 -20
Chemical Shift (ppm)

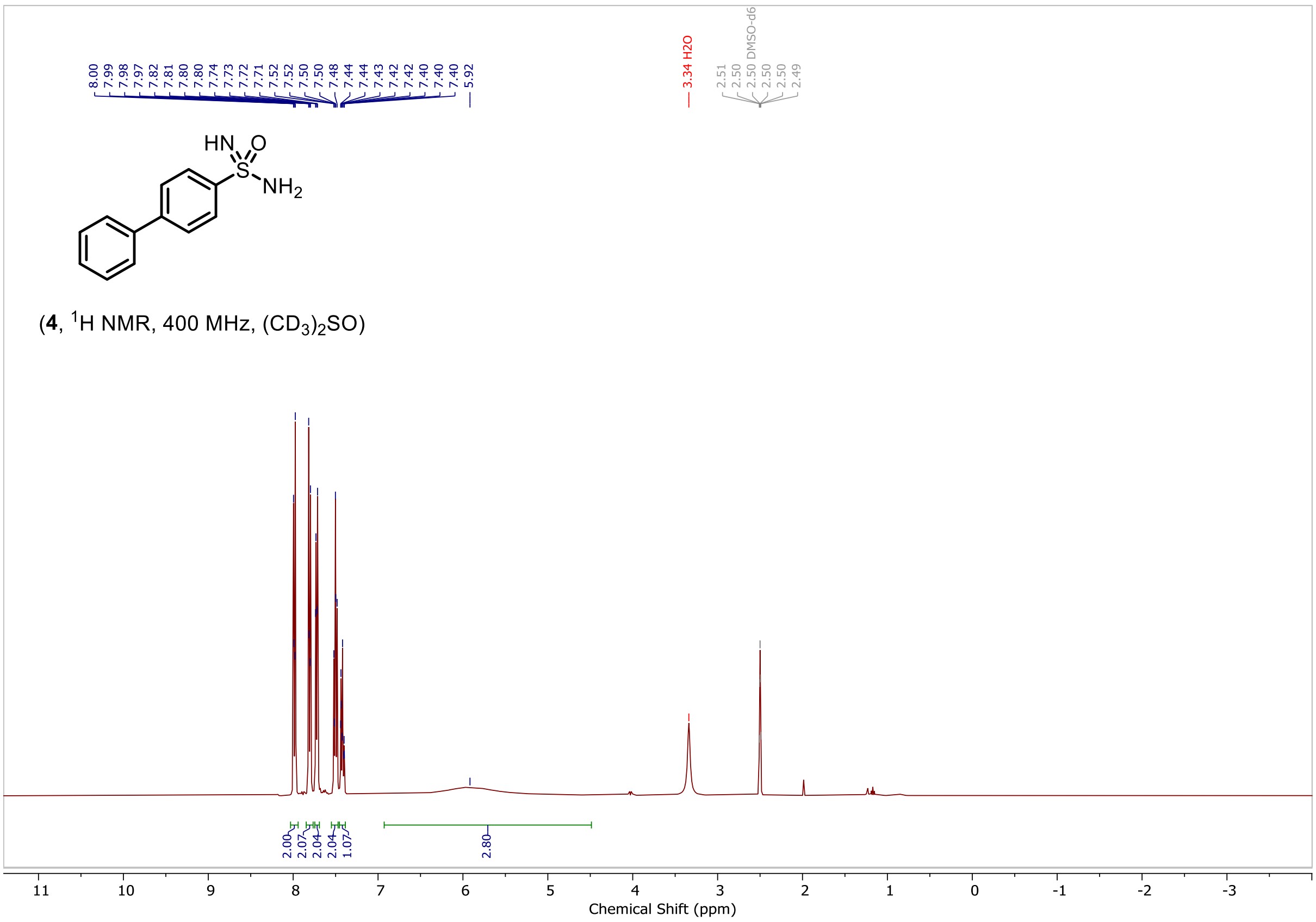

(**4**, ^{1}H NMR, 400 MHz, $(CD_3)_2SO$)

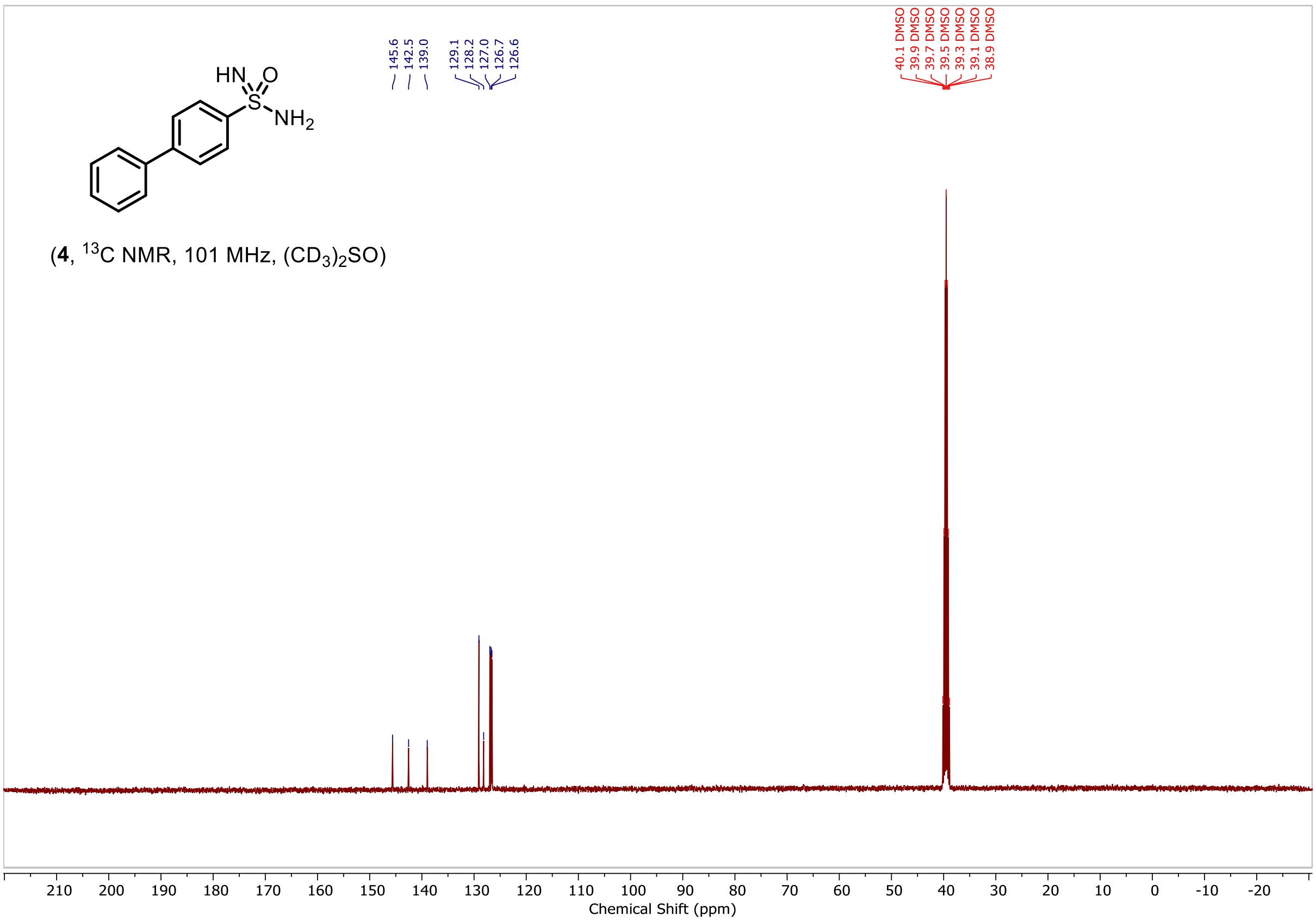

(**4**, ^{13}C NMR, 101 MHz, $(CD_3)_2SO$)
